# Non-enzymatic function of QSOX2 directly regulates the JUNB-ITGB4 axis and enhanced resistance to osimertinib in EGFR-mutation lung adenocarcinoma

**DOI:** 10.1038/s41420-026-02969-4

**Published:** 2026-04-01

**Authors:** Chaoxing Liu, Siya Wang, Rong Qi, Weiguo Gu, Chen Fang, Guohua Zhang, Jinyu Gan, Feng Yu, Ke Fang, Jianxiong Deng, Chao Shi, Feng Qiu

**Affiliations:** 1 Department of Oncology, Gaoxin Branch of The First Affiliated Hospital, Jiangxi Medical College, Nanchang University, Nanchang, PR China; 2 Department of Oncology, The First Affiliated Hospital, Jiangxi Medical College, Nanchang University, Nanchang, PR China; 3 Nanchang Key Laboratory of Tumor Gene Diagnosis and Innovative Treatment Research, Gaoxin Branch Of The First Affiliated Hospital, Jiangxi Medical College, Nanchang University, Nanchang, PR China

**Keywords:** Non-small-cell lung cancer, Preclinical research

## Abstract

Third-generation epidermal growth factor receptor tyrosine kinase inhibitors (EGFR-TKIs) represent a significant advancement in the targeted therapy of lung adenocarcinoma (LUAD), markedly prolonging patient overall survival. However, resistance remains a major barrier to sustained clinical benefit. Beyond the classical EGFR resistance pathways, EGFR-independent bypass mechanisms have emerged as a critical research focus. Quiescent sulfhydryl oxidase 2 (QSOX2), linked to poor outcomes in various cancers, remains poorly studied in LUAD, with its role in tumor progression mechanisms largely unknown. In this study, we demonstrated that the specific high expression of QSOX2 in LUAD induced osimertinib resistance (OR). Mechanistically, QSOX2 directly binds to and stabilizes the transcription factor JUNB via a non-enzymatic interaction, promoting JUNB phosphorylation and nuclear translocation through AKT pathway activation. This results in the transcriptional activation of ITGB4, which in turn initiates FAK/AKT signaling to establish a positive feedback loop that ultimately drives osimertinib bypass resistance. In conclusion, this study innovatively identified the non-enzymatic function of QSOX2 in regulating OR in EGFR-mutant LUAD through the JUNB-ITGB4-FAK/AKT pathway. The QSOX2/JUNB-ITGB4 signaling axis represents a potential therapeutic target for overcoming OR and offers a novel strategy to improve outcomes in LUAD patients.

## Background

Lung cancer remains the leading cause of cancer-related mortality worldwide [1], with lung adenocarcinoma (LUAD) accounting for over 65% of all cases. Driver gene mutations are a primary mechanism behind LUAD development [2], with epidermal growth factor receptor (EGFR) being one of the most common mutations, regulating pathways such as PI3K/AKT and MAPK involved in LUAD progression [3, 4]. In East Asian LUAD patients, the EGFR mutation rate is ~40–60% [5, 6], and EGFR-TKIs have revolutionized the treatment of EGFR-mutated LUAD [7]. Unlike first- and second-generation TKIs, third-generation EGFR-TKIs, such as osimertinib, form irreversible covalent bonds with EGFR and have shown remarkable efficacy in treating patients with EGFR-mutant LUAD, including those with resistance to first-generation TKIs due to the EGFR T790M mutation [8]. However, while the median progression-free survival (mPFS) reached 18.9 months, osimertinib resistance (OR) remains an inevitable challenge [9]. Emerging evidence has elucidated the complexity and heterogeneity of resistance mechanisms to EGFR-targeted therapies, which can be broadly categorized into EGFR-dependent and EGFR-independent pathways. EGFR-dependent mechanisms include the emergence of tertiary mutations such as EGFR C797S and EGFR L718Q, which impair drug binding to the kinase domain of third-generation EGFR-TKIs [10]. Concurrently, the scientific community has increasingly recognized the critical role of EGFR-independent resistance pathways, particularly those involving activation of alternative bypass signaling networks. Key mechanisms in this category encompass MET proto-oncogene amplification, HER2 receptor tyrosine kinase amplification, oncogenic PIK3CA mutations, and AXL kinase activation, which can initiate parallel survival signaling cascades that bypass EGFR inhibition [11–14]. Notably, nearly 50% of the resistance mechanisms are still unclear [15]. Therefore, identifying EGFR-independent bypass mechanisms and new therapeutic targets will deepen our understanding of resistance to third-generation EGFR-TKIs in LUAD and facilitate the development of effective clinical strategies.

Quiescin sulfhydryl oxidase (QSOX) is a flavoprotein belonging to the flavin adenine dinucleotide-dependent sulfhydryl oxidase family. It catalyzes the formation of disulfide bonds and reduces oxygen to hydrogen peroxide, functioning enzymatically both intra- and extracellularly [16–19]. The QSOX family consists of two proteins: QSOX1 and QSOX2 [18]. QSOX1 has been implicated in tumor proliferation, metastasis, and mediating drug resistance in various malignancies, including non-small cell lung cancer (NSCLC) [20–22]. In contrast, QSOX2 has been less studied in cancer, though it shares 41.2% sequence homology and key functional features with QSOX1 [23], suggesting similar biological functions. As a member of the oxidase family, previous studies have linked QSOX2 expression to poor prognosis and aggressive phenotypes in colorectal cancer [24] and triple-negative breast cancer [25]. In advanced NSCLC, our preliminary research revealed that QSOX2 induced poor prognosis [26]. However, the specific molecular mechanisms by which the QSOX2 promotes tumor remain unclear.

Integrin β4 (ITGB4) is a member of the integrin family and contains a large cytoplasmic domain of 1017 amino acids, which allows it to interact with both extracellular matrix (ECM) proteins and intracellular kinases, granting it strong signaling and regulatory capacities [27]. ITGB4 is significantly upregulated in multiple cancers, including pancreatic and renal clear cell carcinomas, and is associated with poor prognosis [28, 29]. Furthermore, ITGB4 plays a pivotal role in activating the FAK/PI3K/AKT signaling pathway, promoting cancer invasion and metastasis [30, 31]. Although ITGB4 has been reported as a potential marker of poor prognosis and cisplatin resistance in lung cancer [32, 33], its role in LUAD progression and third-generation TKI resistance remains unknown.

In this study, we found that QSOX2 is notably upregulated in LUAD and that its overexpression significantly increases ITGB4 expression and activates the FAK/AKT signaling pathway, as revealed by RNA sequencing (RNA-seq) analysis. Furthermore, we demonstrated that QSOX2 plays a critical role in OR. Mechanistically, by generating a non-enzymatic QSOX2 mutant, we demonstrated that QSOX2 directly binds to the transcription factor (TF) JUNB via a non-enzymatic mechanism. This interaction stabilizes JUNB by inhibiting its proteasomal degradation. Subsequently, the stabilized JUNB is phosphorylated and translocated into the nucleus under the regulation of the activated AKT pathway, leading to the transcriptional activation of ITGB4. The upregulated ITGB4 then activates the FAK/AKT signaling pathway. This establishes a positive feedback loop that ultimately drives the development of resistance to osimertinib. Understanding the non-enzymatic mechanisms mediated by QSOX2 could assist in the development of novel biomarkers and therapeutic strategies to overcome OR in EGFR-mutated LUAD.

## Results

### QSOX2 is characteristically upregulated in LUAD cells and tissues

**Fig. 1:**
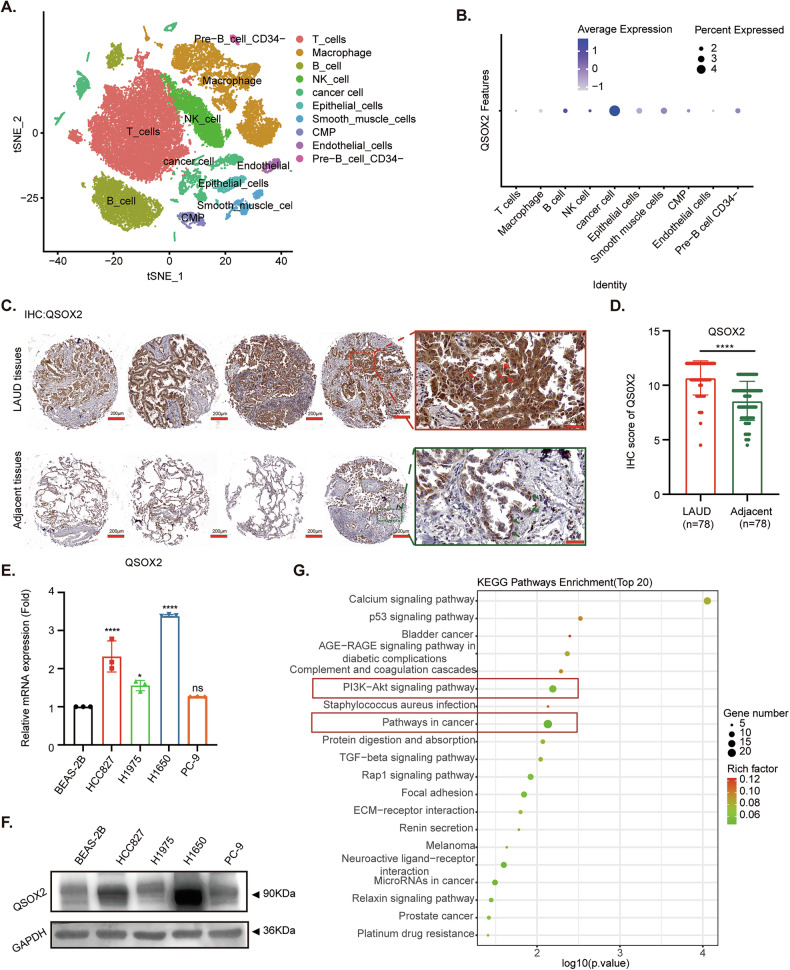
QSOX2 is characteristically upregulated in LUAD cells and tissues. **A** tSNE analysis of single-cell RNA-sequencing data showing the distribution of 10 cell types in LUAD. **B** Dot plot illustrating the expression of QSOX2 across different cell types in LUAD. **C**, **D** IHC staining of QSOX2 in LUAD tissues and adjacent normal tissues. **E** RT-qPCR analyses QSOX2 mRNA expression in BEAS-2B, HCC827, H1975, H1650 and PC-9 cell lines. Data are presented as fold change compared to the BEAS-2B group. **F** Western blot analyses of QSOX2 protein levels in BEAS-2B, HCC827, H1975, H1650 and PC-9 cell lines. GAPDH serves as a loading control. **G** KEGG-pathway enrichment of upregulated differentially expressed genes in H1975-QSOX2-flag and Vector-flag after RNA-sequencing (top 20). (*P < 0.05, **P < 0.01, ***P < 0.001, ****P < 0.0001). Statistical analysis was performed using one-way ANOVA. Data are represented as the mean ± SEM value from three biological replicates.

To investigate the specific cellular components in tumor tissues where QSOX2 is abnormally expressed and exerts tumor-promoting effects, we downloaded the LUAD dataset from the GEO database (GSE131907). After quality control, integration, and dimensionality reduction clustering, and strictly following Seurat’s standard protocol, we annotated the cell populations into 10 subtypes (Fig. 1A). Subsequently, we clarified the expression levels of QSOX2 in each cell population and found that QSOX2 is specifically highly expressed in cancer cells (Fig. 1B). Additionally, we verified the expression levels of QSOX2 on a tissue microarray, where QSOX2 is significantly overexpressed in tumor parenchymal (Fig. 1C, D). Subsequently, we selected EGFR-mutant LUAD cell lines (HCC827, H1975, H1650 and PC-9) and normal lung epithelial cells (BEAS-2B) for RT-qPCR and Western blot experiments. The RNA (Fig. 1E) and protein (Fig. 1F) expression levels of QSOX2 in LUAD cells (HCC827, H1975, H1650) were both higher than those in BEAS-2B, with the highest protein expression observed in H1650 cells, while H1975 cells showed a relatively lower expression. To further explore the biological function of QSOX2 in LUAD. We performed RNA-seq on the stable overexpression cell line H1975-QSOX2-flag and the control group H1975-Vector-flag cells. KEGG-pathway enrichment analysis of the upregulated differential genes indicated significant enrichment in the pathways related to cancer and the PI3K/AKT signaling pathway (Fig. 1G). In summary, our results confirmed that QSOX2 is specifically upregulated in LUAD cells and tissues. Overexpression of QSOX2 is associated with activation of the PI3K/AKT pathway, which is implicated in specific biological behaviors in patients with LUAD.

### QSOX2 mediates poor prognosis of LUAD patients treated with third-generation EGFR-TKIs and induces OR

**Fig. 2:**
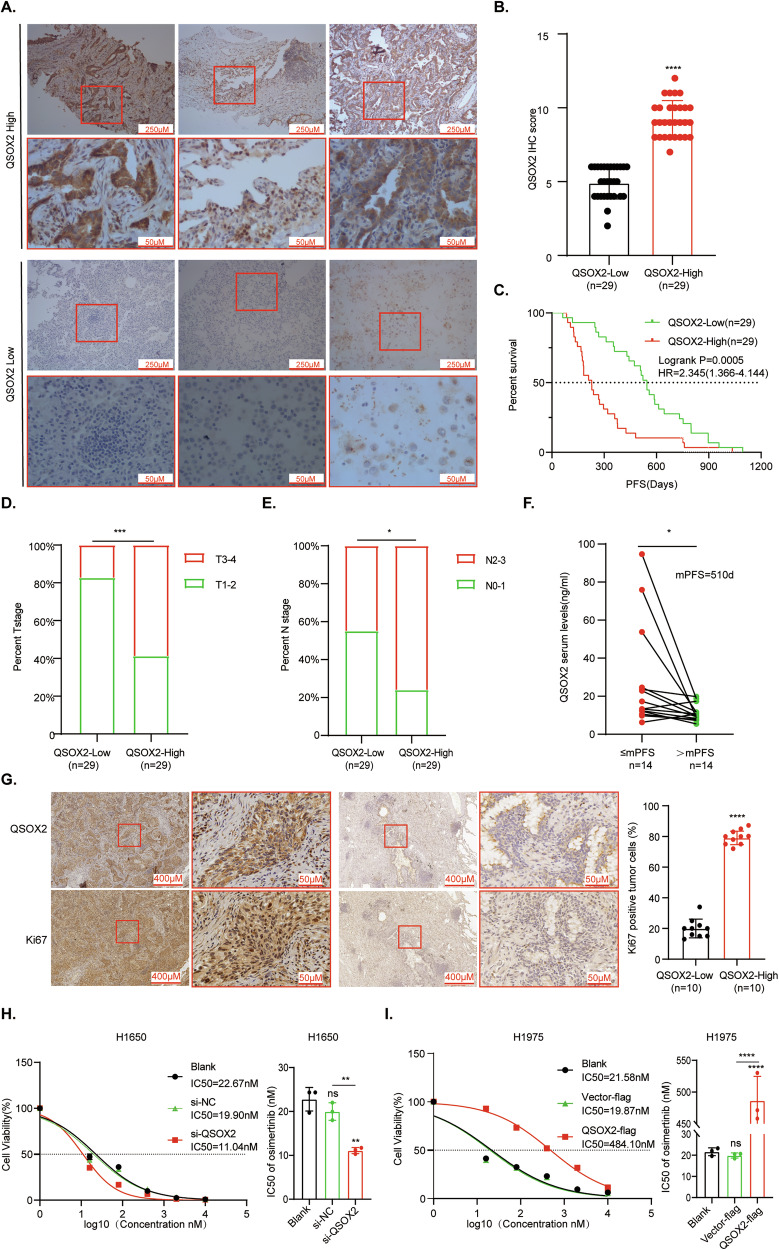
QSOX2 mediates the poor prognosis of LUAD patients treated with third-generation EGFR-TKIs and induces OR. **A** IHC staining for QSOX2 expression in LUAD tissues with QSOX2-high and QSOX2-low groups. The top row shows representative images of QSOX2-high expression, and the bottom row shows QSOX2-low tissues. Enlarged views in red boxes highlight QSOX2 staining intensities in specific regions (4X, 20X). **B** Quantification of QSOX2 IHC scores in QSOX2-high and QSOX2-low groups, by two-tailed t-test. **C** Kaplan-Meier survival analysis of PFS in LUAD patients stratified by QSOX2 expression levels. Patients with high QSOX2 expression have significantly shorter PFS compared to those with low QSOX2 expression (HR = 2.345(1.366–4.144, *P* = 0.0005). **D** Association between QSOX2 expression and clinical T stage or N stage (**E**). **F** Elevated serum QSOX2 levels are associated with shorter PFS. **G** Correlation analysis of QSOX2 expression and Ki-67 proliferation index, by two-tailed t-test. **H** Dose-response curves showing cell viability of H1650 cells treated with osimertinib in si-QSOX2 (11.04 nM)/si-NC (19.90 nM)/Blank group (22.67 nM). **I** Dose-response curves showing cell viability of H1975 cells treated with osimertinib in QSOX2-flag (484.10 nM)/Vector-flag (19.87 nM)/Blank (21.58 nM) (*P < 0.05, **P < 0.01, ***P < 0.001, ****P < 0.0001). Statistical analysis was performed using one-way ANOVA. Data are represented as the mean ± SEM value from three biological replicates.

Given the established role of PI3K/AKT signaling in third-generation EGFR-TKI resistance [3, 34], we investigated QSOX2’s potential involvement in this mechanism. Our retrospective analysis of 58 EGFR-mutant LUAD patients treated with third-generation EGFR-TKIs revealed distinct clinical outcomes based on QSOX2 expression levels. Compared to the QSOX2-low group (IHC score≤6, *n* = 29), patients with QSOX2-high expression (IHC score >6, *n* = 29) exhibited predominant cytoplasmic and nuclear localization (Fig. 2A, B). Notably, the QSOX2-high cohort demonstrated significantly shorter PFS following osimertinib treatment (HR = 2.345 (1.366–4.144, *P* = 0.0005) (Fig. 2C), establishing QSOX2 overexpression as an independent adverse prognostic marker. To further elucidate the clinical significance of QSOX2, we analyzed its correlation with clinicopathological characteristics (Supplementary Table 6). High QSOX2 expression was significantly associated with advanced T stage and N stage (Fig. 2D, E). We further assessed the potential of serum QSOX2 as a prognostic biomarker in 28 patients receiving third-generation EGFR-TKI therapy. Patients with shorter mPFS (PFS ≤ 510 days) exhibited significantly higher serum QSOX2 levels compared to those with longer PFS (>510 days) (Fig. 2F). Consistent with these findings, IHC analysis revealed a positive correlation between QSOX2 expression and the Ki-67 proliferation index in samples from high and low expression groups (*n* = 10 per group; Fig. 2G), supporting a role for QSOX2 in promoting tumor proliferation. Functional validation in EGFR-mutant cell lines confirmed QSOX2’s role in drug tolerance. In H1650 cells, the IC50 of the si-QSOX2 group was significantly reduced compared to the si-NC group (19.90 nM vs 11.04 nM) (Fig. 2H). Meanwhile, in H1975 cells, the IC50 of the QSOX2-flag group was significantly increased by 24.36-fold compared to the Vector-flag group (19.87 nM vs 484.10 nM) (Fig. 2I), suggesting that QSOX2 significantly enhances the tolerance of EGFR-mutant cells to osimertinib. Collectively, our preclinical analyses demonstrate that QSOX2 overexpression serves as both a prognostic biomarker and functional mediator of OR in EGFR-mutant LUAD.

### QSOX2 positively regulates ITGB4 and downstream FAK/AKT signaling

**Fig. 3:**
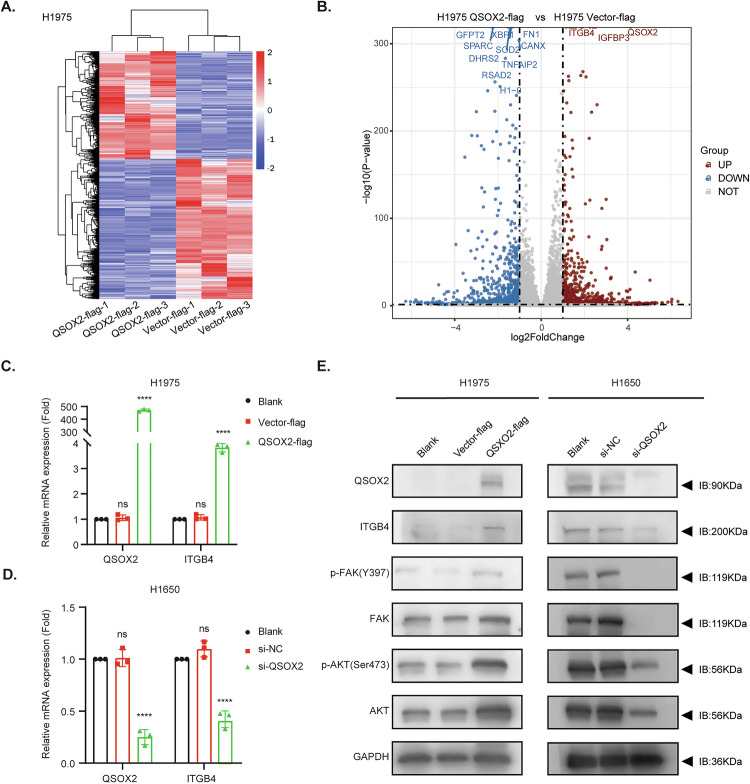
QSOX2 positively regulates ITGB4 and FAK/AKT signaling expression in LUAD cell lines. **A** The differentially expressed genes between H1975-QSOX2-flag and Vector-flag were significantly divided into 4 clusters after RNA sequencing. Red represents an increase, and blue represents a decrease. **B** The volcano plot shows that with the QSOX2-flag, genes such as ITGB4 are increased simultaneously. Red represents an increase, and blue represents a decrease. **C** RT-qPCR analysis of QSOX2, ITGB4 mRNA expression in H1975-QSOX2-flag and H1650-si-QSOX2 cell lines (**D**). **E** Western blot analysis of QSOX2, ITGB4, p-FAK, total FAK, p-AKT, and total AKT in H1975 and H1650 cells. GAPDH serves as a loading control. Data are presented as fold change compared to the blank group. (*P < 0.05, **P < 0.01, ***P < 0.001, ****P < 0.0001). Statistical analysis was performed using one-way ANOVA. Data are represented as the mean ± SEM value from three biological replicates.

To elucidate the mechanism of signal transduction by which QSOX2 induces OR, RNA-seq analysis revealed that stable overexpression of QSOX2 altered the expression patterns of downstream differentially expressed genes (Fig. 3A). Notably, ITGB4 and IGFBP3 expression was significantly upregulated with QSOX2-OE (Fig. 3B). Previous studies have indicated that ITGB4 participates in EGFR-related downstream pathways and contributes to stemness and cisplatin resistance in lung cancer cells [33, 35, 36]. However, it remains unclear whether ITGB4 and the FAK/AKT signaling pathway are involved in QSOX2-mediated OR. To investigate the changes in key molecular markers, we conducted experimental analyses at both the RNA and protein levels. RT-qPCR analysis confirmed that QSOX2 positively regulates ITGB4 expression (Fig. 3C, D). Compared with the blank and vector controls, ITGB4, p-FAK (phosphorylated FAK), and p-AKT (phosphorylated AKT) were significantly elevated in the QSOX2-flag group, whereas these markers were notably reduced in the si-QSOX2 group (Fig. 3E and Supplementary Fig. 1A, B). Furthermore, transcriptome sequencing revealed that IGFBP3 expression was significantly elevated following QSOX2-flag (Supplementary Fig. 1C). Given that IGFBP3 serves as a primary binding protein for IGF1, we further investigated the potential involvement of the growth hormone/IGF1 pathway in QSOX2-mediated OR. Western blot analysis of tumor cells and ELISA-based measurement of serum IGF1 levels in patients treated with third-generation EGFR-TKIs collectively demonstrated that IGF1 is not involved in the QSOX2-mediated resistance to third-generation EGFR-TKIs (Supplementary Fig. 1D–F). In summary, our results indicated that in EGFR-mutant LUAD cell lines, QSOX2 positively regulates the expression of ITGB4 and activates the downstream FAK/AKT signaling axis.

### QSOX2 enhances ITGB4 membrane expression and activates the FAK/AKT pathway to induce OR

**Fig. 4:**
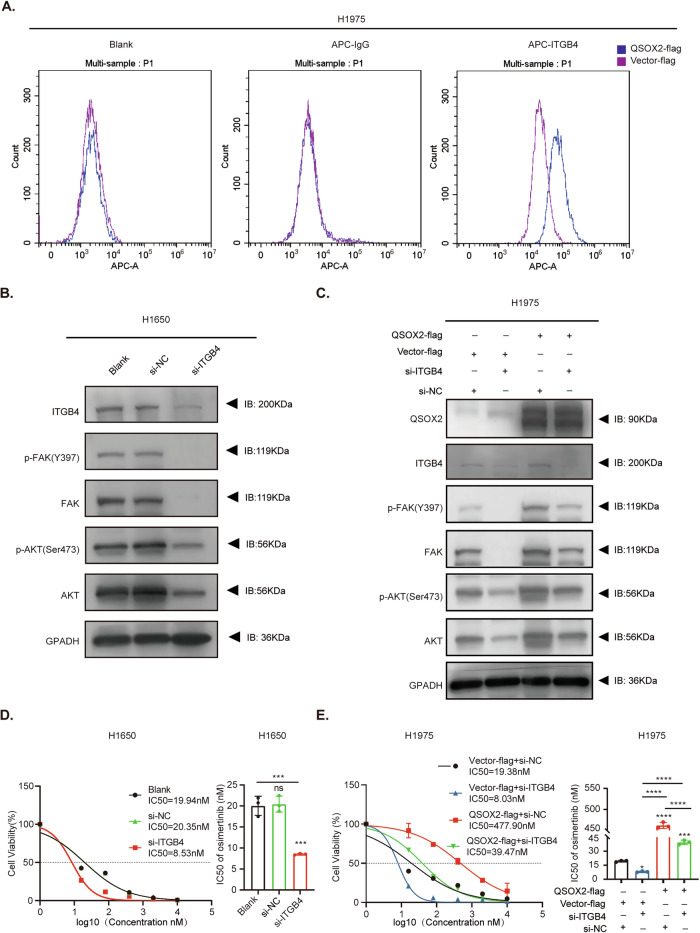
QSOX2 enhances ITGB4 membrane expression and mediates FAK-AKT axis-induced OR. **A** Flow cytometry analysis showing ITGB4 membrane expression in H1975-QSOX2-flag /Vector-flag. Cells were labeled with APC-conjugated anti-ITGB4 antibody. APC-IgG was used as a negative control. **B** Western blot analyses showing the effect of ITGB4 knockdown on the FAK/AKT signaling pathway in H1650 cells. Protein expression levels of ITGB4, p-FAK, total FAK, p-AKT, and total AKT were assessed across three groups: Blank, si-NC, and si-ITGB4. GAPDH serves as a loading control. **C** Western blot analyses the effects of QSOX2-OE and ITGB4 knockdown on the FAK/AKT signaling pathway in H1975 cells. Protein expression levels of ITGB4, QSOX2, p-FAK, total FAK, and p-AKT were evaluated under different conditions, including QSOX2-flag, Vector-flag, si-NC, and si-ITGB4. GAPDH serves as a loading control. **D** Dose-response curves showing cell viability of H1650 cells treated with osimertinib under different conditions. The IC50 values for each group are indicated: Blank (19.94 nM), si-NC (20.35 nM), and si-ITGB4 (8.53 nM). **E** Dose-response curves showing cell viability of H1975-QSOX2-flag/ H1975-Vector-flag cells treated with osimertinib under different conditions. IC50 values are indicated for each group: Vector-flag + si-NC (19.38 nM), Vector-flag+si-ITGB4 (8.03 nM), QSOX2-flag+si-NC (477.90 nM), and QSOX2-flag+si-ITGB4 (39.47 nM). Statistical analysis was performed using one-way ANOVA. Data are presented as mean ± SEM value from three biological replicates, *P < 0.05, **P < 0.01, ***P < 0.001, ****P < 0.0001, ns not significant.

To investigate the specific functional sites of ITGB4 regulated by QSOX2, we initially performed flow cytometry analysis. Results showed a significantly higher proportion of membrane-bound ITGB4-positive cells in the QSOX2-flag group compared to the Vector-flag group (Fig. 4A). Accordingly, to further investigate whether QSOX2 regulates the downstream FAK/AKT pathway through ITGB4-mediated mechanisms, we first performed ITGB4 knockdown in H1650 cells using siRNA-mediated gene silencing. Western blot analysis confirmed that ITGB4 depletion significantly suppressed the activation of FAK/AKT signaling pathway components (Fig. 4B and Supplementary Fig. 2A). Notably, in QSOX2-Flag cells, si-ITGB4 attenuated the effect of QSOX2 on the activation of the FAK/AKT pathway, highlighting the critical role of ITGB4 in QSOX2-mediated OR (Fig. 4C and Supplementary Fig. 2B). Next, to clarify the molecular effects of ITGB4 in QSOX2-mediated OR, we assessed osimertinib sensitivity. The IC50 of osimertinib in the si-ITGB4 group (8.53 nM) was markedly lower than in the blank (19.94 nM) and si-NC (20.35 nM) (Fig. 4D), supporting the role of ITGB4 in QSOX2-induced OR. Furthermore, to investigate the functional interaction between QSOX2 and ITGB4 in OR, we conducted CCK8 rescue assays in H1975-QSOX2-flag and Vector-flag cell lines. Following co-transfection with either si-ITGB4 or si-NC, osimertinib sensitivity was quantitatively assessed. The QSOX2-flag + si-NC group exhibited significantly elevated IC50 (477.90 nM) compared to control groups (Vector + si-ITGB4: 8.03 nM; Vector + si-NC: 19.38 nM). Notably, ITGB4 knockdown effectively restored drug sensitivity in QSOX2-overexpressing cells, reducing the IC50 to 39.47 nM (*P* < 0.001) (Fig. 4E). Taken together, these findings indicated that QSOX2 mediates OR in EGFR-mutant LUAD cells by increasing the expression of ITGB4, which activates downstream FAK/AKT signaling.

### QSOX2 transcriptionally activates ITGB4 expression via JUNB

**Fig. 5:**
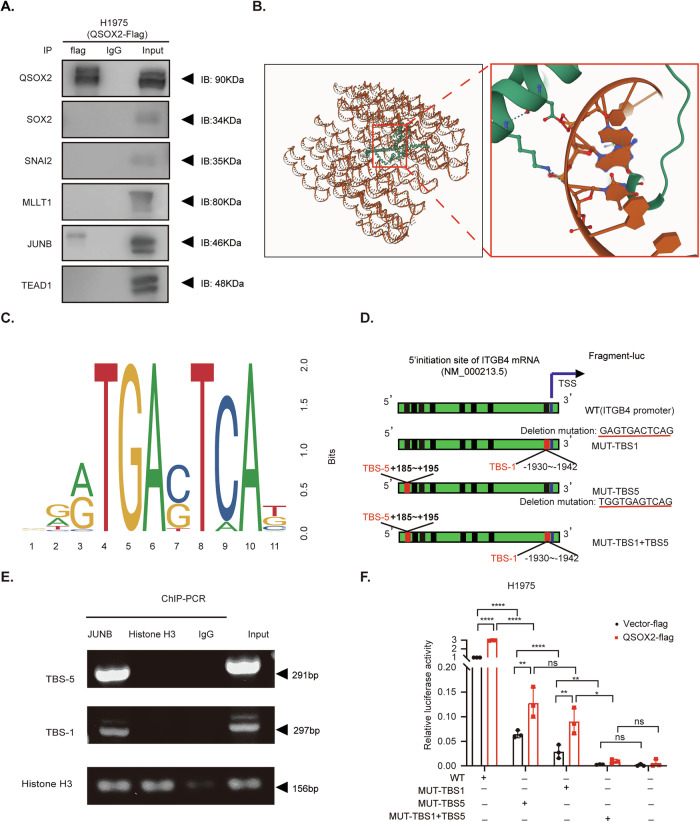
QSOX2 transcriptionally activates ITGB4 expression via JUNB. **A** Co-IP assay showing interactions between QSOX2 and potential transcription factors SOX2, SNAI2, MLLT1, JUNB, and TEAD1. **B** The binding region of JUNB (protein) and ITGB4 (DNA) predicted by AlphaFold3.0. The red box is an enlarged view of the binding domain. **C** Sequence logo of the DNA binding site, showing nucleotide preferences within the protein-binding region. Letter size reflects the frequency of each nucleotide at each position. **D** The diagram of the ITGB4 promoter reporter gene construction, including the wild-type (WT) and deletion mutant (MUT-TBS1, MUT-TBS5, and double mutant MUT-TBS1 + TBS5) fragments. Mutated binding sites are marked in red. **E** ChIP-PCR assay showing JUNB binding to TBS-5 and TBS-1 sites in the ITGB4 promoter. **F** Dual-luciferase reporter assays showing the effect of QSOX2-OE on ITGB4 promoter activity. Data are presented as mean ± SEM value from three biological replicates, with statistical significance indicated as follows: *P < 0.05, **P < 0.01, ***P < 0.001, ****P < 0.0001, ns for not significant.

To further elucidate the direct mechanism by which QSOX2 regulates ITGB4 expression, we examined the subcellular localization of QSOX2 in patients with high QSOX2 expression following third-generation EGFR-TKI treatment. As previously described, QSOX2 predominantly localizes to both the cytoplasm and nucleus, suggesting a potential role in transcriptional regulation via modulation of transcription factors. To identify potential transcription factors responsible for ITGB4 regulation, we queried the ChIP-Atlas database (https://chip-atlas.org/) and shortlisted the top five candidates (Supplementary Table 7). Co-IP experiments were then conducted to assess the interaction between QSOX2 and the five candidate transcription factors: SOX2, SNAI2, MLLT1, JUNB, and TEAD1. Among them, JUNB was considered to be a potential TF for ITGB4 (Fig. 5A). To further explore the interaction between JUNB and the ITGB4 promoter, we performed protein-DNA molecular docking using AlphaFold 3.0. The model revealed a high-confidence binding interface between JUNB (protein) and the ITGB4 promoter (DNA) (Fig. 5B). Additionally, bioinformatic analysis of the ITGB4 promoter region using UniProt identified eight putative transcription factor binding sites (TBS-1 to TBS-8). Two high-confidence JUNB-binding motifs were predicted at TBS-1 and TBS-5. Corresponding deletion mutants were constructed and are documented in Supplementary Table 8. Sequence analysis further characterized the nucleotide preferences within these binding regions (Fig. 5C). To functionally validate these predicted sites, we constructed wild-type and site-specific deletion mutant plasmids for TBS-1, TBS-5, and a double mutant (MUT-TBS1, MUT-TBS5, and MUT-TBS1 + TBS5, respectively) (Fig. 5D). ChIP-PCR assays confirmed direct binding of JUNB to the TBS-1 and TBS-5 regions in the ITGB4 promoter (Fig. 5E). We next performed dual-luciferase reporter assays to evaluate the functional relevance of these binding sites. Compared to the wild-type construct, luciferase activity was significantly reduced in the MUT-TBS1, MUT-TBS5, and especially in the MUT-TBS1 + TBS5 group (Fig. 5F), indicating that TBS-1 and TBS-5 are critical for JUNB-mediated transcriptional activation of ITGB4. Furthermore, the QSOX2-flag group significantly enhanced luciferase activity compared to the Vector-flag, suggesting that QSOX2 positively regulates ITGB4 transcription through JUNB. In summary, our findings demonstrate that through mapping QSOX2 expression patterns, we identified JUNB as a critical transcriptional regulator. Specifically, JUNB binds to two functionally essential regions (TBS-1 and TBS-5) within the ITGB4 promoter, thereby transcriptionally activating ITGB4 expression.

### QSOX2 interacts with JUNB to facilitate nuclear translocation through a non-enzymatic mechanism

**Fig. 6:**
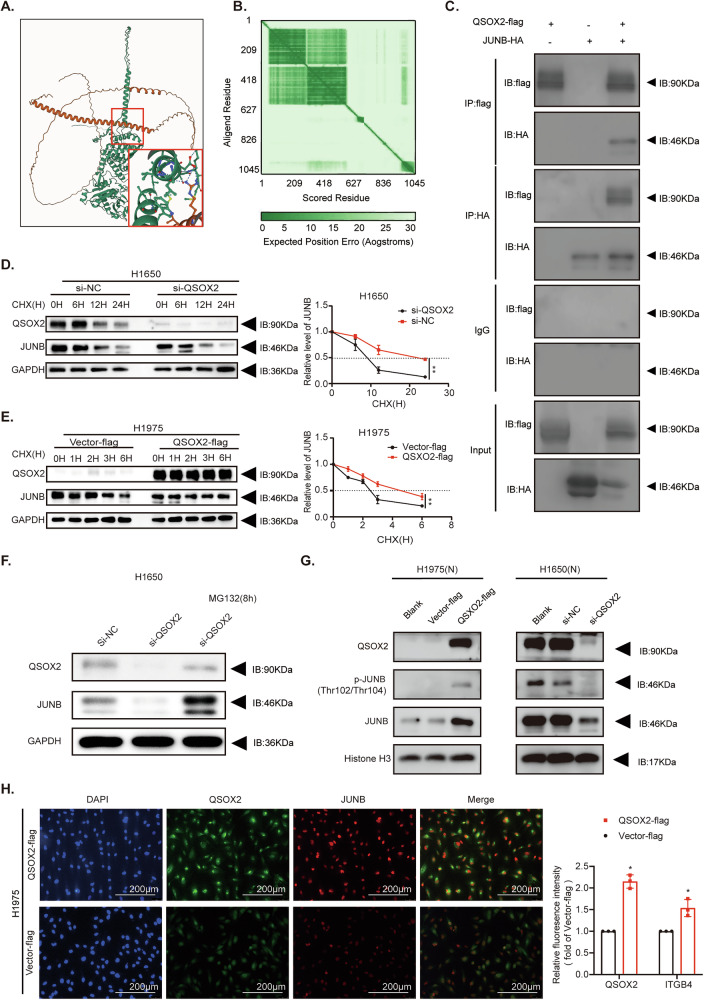
QSOX2 stabilizes JUNB protein and promotes its nuclear translocation via a proteasome-dependent mechanism. **A** The binding region of QSOX2 (protein) and JUNB (protein) predicted by AlphaFold3.0. The red box is an enlarged view of the binding domain. **B** Predicted alignment error heatmap for the QSOX2/JUNB protein, indicating the confidence in structural prediction across residues. Darker shades along the diagonal represent higher accuracy in alignment of residues, while lighter areas indicate regions with lower confidence. **C** Co-IP analyses of QSOX2 and JUNB interaction. In H1975 cells, QSOX2 and JUNB were tagged with flag and HA, respectively. Input was the positive control group, and IgG was the negative control. **D** In H1650 cells transfected with si-NC or si-QSOX2, protein degradation of JUNB was detected by Western Blot after treatment with CHX (50 μg/mL) for the indicated durations. **E** In H1975 cells transfected with Vector-flag or QSOX2-flag, changes in JUNB protein levels were similarly assessed following CHX (50 μg/mL) treatment for the indicated times. The line graph on the right shows quantitative analysis of the relative expression of JUNB over time under each condition (mean ± SD, *n* = 3; **p < 0.01). **F** H1650 cells were transfected with si-NC or si-QSOX2, and treated with or without the proteasome inhibitor MG132 (10 μM, 8 h). Western blot analysis was performed to detect the protein levels of QSOX2, JUNB, and GAPDH (loading control). **G** Western blot analysis of nuclear proteins from H1975 (left) and H1650 (right) cells under indicated transfection conditions, probing for QSOX2, p-JUNB (Thr102/Thr104), total JUNB, and Histone H3 (loading control). **H** IF assay showing the colocalization of QSOX2 (green) and JUNB (red) in H1975 cells. Nuclear staining was performed using DAPI (blue). The top row represents the QSOX2-flag group, and the bottom row represents the Vector-flag control group. Fluorescence intensity quantification is shown on the right. Statistical analysis was performed using one-way ANOVA. Data are represented as the mean ± SEM value from three biological replicates. Statistical significance indicated as follows: *P < 0.05, **P < 0.01, ***P < 0.001, ****P < 0.0001, ns for not significant.

To elucidate the putative regulatory mechanism of QSOX2 on JUNB, we performed structural interaction prediction between QSOX2 and JUNB using AlphaFold 3.0 modeling. The results suggested a potential direct protein-protein interaction (Fig. 6A, B). To experimentally confirm this interaction, we performed Co-IP assays, following exogenous transfection with an HA-JUNB fusion protein. IP using anti-Flag or anti-HA antibodies, followed by Western blot analyses, confirmed the interaction between QSOX2 and JUNB in LUAD cells (Fig. 6C). To evaluate the impact of QSOX2 on JUNB protein stability, we performed CHX chase assays. The results demonstrated that QSOX2 knockdown significantly accelerated the degradation rate of JUNB in H1650 cells, whereas QSOX2 overexpression markedly prolonged JUNB half-life (Fig. 6D, E). Notably, the proteasome inhibitor MG132 rescued the promoted degradation of JUNB induced by QSOX2 knockdown (Fig. 6F), indicating proteasome-dependent stabilization. Nuclear fractionation analysis revealed that QSOX2 overexpression significantly increased the nuclear levels of the QSOX2/JUNB complex and phosphorylated JUNB (p-JUNB), while QSOX2 knockdown reduced their nuclear accumulation compared to controls (Fig. 6G and Supplementary Fig. 2C, D). Additionally, IF demonstrated nuclear co-localization of QSOX2 and JUNB. The nuclear fluorescence intensity of QSOX2/JUNB co-localization was significantly higher in the QSOX2-flag group than in the Vector-flag group (Fig. 6H).

**Fig. 7:**
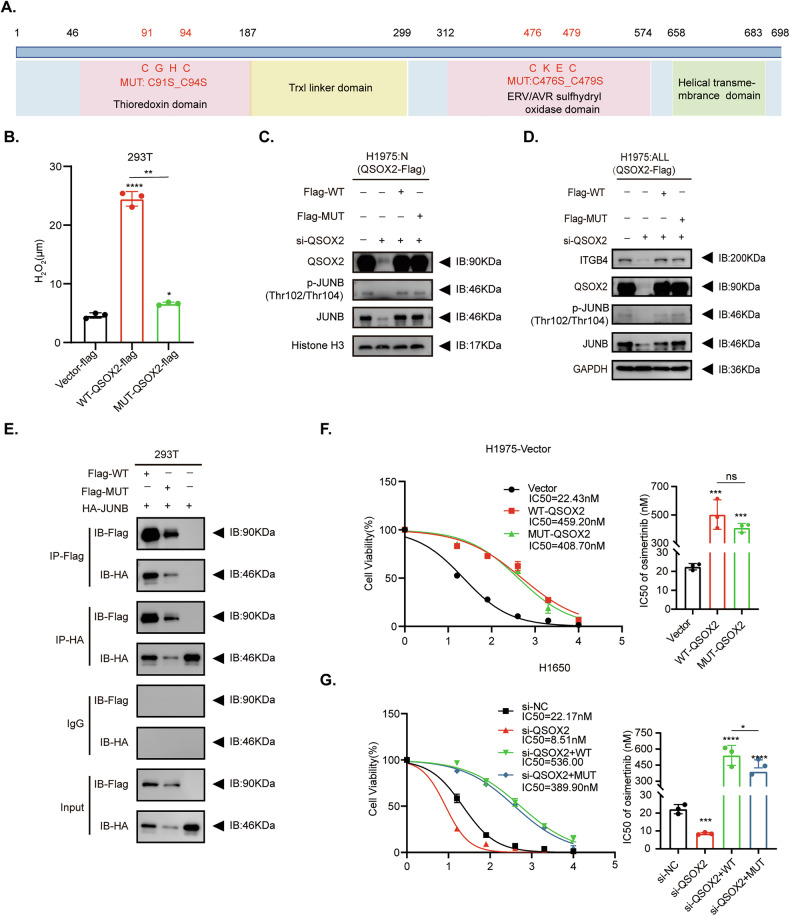
QSOX2 mediates OR independent of its enzymatic activity. **A** Schematic diagram of the domain architecture and catalytic site mutations (C91S_C94S in the Thioredoxin domain and C476S_C479S in the ERV/AVR sulfhydryl oxidase domain) of QSOX2. **B** Intracellular H₂O₂ levels measured in H1975 cells expressing Vector, WT-QSOX2, or MUT-QSOX2. **C** Nuclear protein levels of QSOX2, JUNB and p-JUNB(Thr102/Thr104) in H1975 cells expressing Vector, WT-QSOX2, or MUT-QSOX2. Histone H3 were used as a nuclear marker. **D** Western blot analysis of protein expression in H1975 cells expressing Vector, WT-QSOX2, or MUT-QSOX2. Whole-cell lysates were probed for ITGB4, QSOX2, p-JUNB (Thr102/Thr104), total JUNB. GAPDH served as a loading control. **E** Co-IP assays showing the interaction between JUNB and either WT- or MUT-QSOX2 in 293 T cells. **F** Dose-response curves of osimertinib in H1975-Vector cells expressing empty vector (Vector, IC50 = 22.43 nM), wild-type QSOX2 (WT-QSOX2, IC50 = 459.20 nM), or catalytically inactive QSOX2 (MUT-QSOX2, IC50 = 408.70 nM). Data points represent mean ± SD from three independent experiments. **G** Dose-response curves of osimertinib in H1650 cells under four conditions: non-targeting control (si-NC, IC50 = 22.17 nM), QSOX2 knockdown (si-QSOX2, IC50 = 8.51 nM), and rescue with either wild-type (si-QSOX2 + WT, IC50 = 536.00 nM) or catalytically inactive mutant QSOX2 (si-QSOX2 + MUT, IC50 = 389.90 nM). Data represent mean ± SD from three independent experiments. Statistical analysis was performed using one-way ANOVA. Statistical significance indicated as follows: *P < 0.05, **P < 0.01, ***P < 0.001, ****P < 0.0001, ns for not significant.

To determine whether QSOX2’s function requires enzymatic activity, we generated a catalytically inactive mutant (MUT-QSOX2) by mutating cysteine residues to serine in the CGHC (Thioredoxin domain) and CEKC (EVR/AVR sulfhydryl oxidase domain) motifs (Fig. 7A) [37–39]. H₂O₂ levels were significantly lower in MUT-QSOX2-expressing cells than in WT-QSOX2-expressing cells (Fig. 7B), confirming loss of enzymatic activity. Notably, both WT- and MUT-QSOX2 similarly increased nuclear JUNB and p-JUNB levels (Fig. 7C and Supplementary Fig. 2E) and upregulated ITGB4 expression (Fig. 7D and Supplementary Fig. 2F). Co-IP assays in 293 T cells confirmed that both WT- and MUT-QSOX2 interact with JUNB (Fig. 7E). Functionally, CCK-8 assays in H1975 cells showed significantly increased osimertinib IC50 upon overexpression of either WT- or MUT-QSOX2 (Fig. 7F). Conversely, QSOX2 knockdown in H1650 cells reduced IC50, which was rescued by re-expression of either WT- or MUT-QSOX2 (Fig. 7G). Collectively, these multimodal findings demonstrate that QSOX2 facilitates JUNB nuclear translocation and transcriptional activation of ITGB4 through a novel non-enzymatic mechanism, independently of its sulfhydryl oxidase activity, thereby promoting OR in LUAD.

### QSOX2/JUNB-ITGB4 signaling axis contributes to OR by mediating the FAK/AKT signaling pathway

**Fig. 8:**
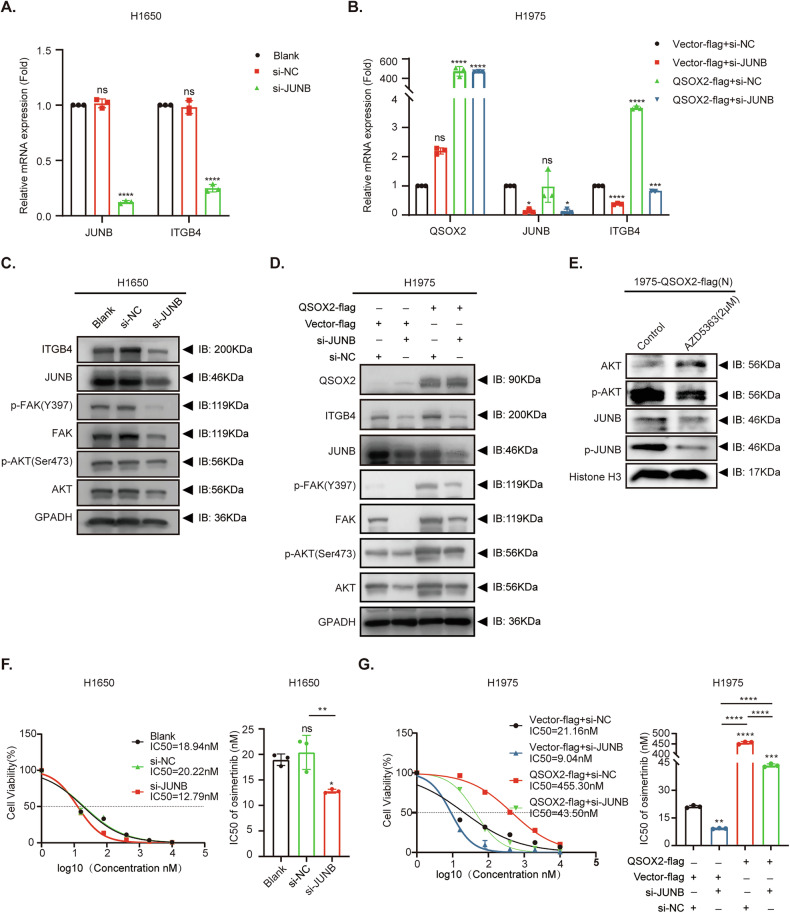
QSOX2/JUNB-ITGB4-FAK/AKT signaling axis contributes to OR. **A** RT-qPCR analyses of JUNB and ITGB4 in H1650 cells following treatment with siRNA-JUNB or negative si-NC. Data are presented as fold change compared to the blank group. **B** RT-qPCR analyses of QSOX2, JUNB, and ITGB4 in H1975 cells following transfection with QSOX2-flag or Vector-flag and si-JUNB or si-NC. QSOX2-OE significantly increased ITGB4 mRNA expression, while JUNB knockdown reversed this effect. Data are presented as fold change compared to the blank group. **C** Western blot analyses of ITGB4, JUNB, p-FAK, total FAK, p-AKT, and total AKT in H1650 cells after JUNB knockdown. GAPDH was used as a control internal reference. **D** Western blot analyses of ITGB4, QSOX2, JUNB, p-FAK, total FAK, p-AKT, and total AKT were analyzed by Western blot. The results show that QSOX2 enhances ITGB4 expression and activates FAK/AKT signaling, while JUNB knockdown reversed these effects. GAPDH was used as a control internal reference. **E** Western blot analysis of nuclear extracts from H1975-QSOX2-Flag cells treated with Control (DMSO) or the AKT inhibitor (AZD5363 2 μM). The levels of total AKT, p-AKT, total JUNB, p-JUNB, and the nuclear loading control Histone H3 are shown. **F** Dose-response curves showing cell viability of H1650 cells treated with osimertinib under different conditions. Cells were either untreated (Blank) or treated with si-NC (negative control) or si-JUNB. The IC50 values for each group are indicated: Blank (18.94 nM), si-NC (20.22 nM), and si-JUNB (12.79 nM). **G** Dose-response curves showing cell viability of H1975 cells treated with osimertinib under different conditions. Cells were transfected with either Vector-flag or QSOX2-flag constructs, and treated with either si-NC (negative control) or si-JUNB. IC50 values are indicated for each group: Vector-flag + si-NC (21.16 nM), Vector-flag + si-JUNB (9.04 nM), QSOX2-flag+si-NC (455.30 nM), and QSOX2-flag+ si-JUNB (43.50 nM).Statistical analysis was performed using one-way ANOVA. Data are represented as the mean ± SEM value from three biological replicates. ****P < 0.0001, ***P < 0.001, **P < 0.01, *P < 0.05, ns for not significant.

To elucidate the role of JUNB in promoting OR via the QSOX2/JUNB-ITGB4-FAK-AKT signaling axis, we performed JUNB knockdown in H1650 cells. RNA analyses revealed that silencing JUNB reduced ITGB4 expression (Fig. 8A). Rescue experiments in QSOX2-flag and Vector-flag cells demonstrated that ITGB4 expression was significantly elevated in the QSOX2-flag group, whereas JUNB knockdown in this group suppressed ITGB4 levels (Fig. 8B). Western blot analyses in H1650 cells showed reduced expression of ITGB4, p-FAK, and p-AKT in the JUNB knockdown group compared to si-NC and blank controls (Fig. 8C and Supplementary Fig. 2G). Rescue experiments in QSOX2-flag/Vector-flag cells confirmed that QSOX2 overexpression enhanced ITGB4 expression and activated downstream FAK-AKT signaling, while JUNB knockdown reversed these effects (Fig. 8D and Supplementary Fig. 2H). To further identify the upstream kinase responsible for JUNB phosphorylation, we performed western blot analyses in H1975-QSOX2-Flag cells. The results demonstrated that AKT inhibitor treatment significantly reduced nuclear p-JUNB levels (Fig. 8E and Supplementary Fig. 2I). In contrast, the MEK inhibitor U0126 not only failed to suppress p-JUNB but also increased nuclear p-AKT levels (Supplementary Figs. 2J and 3A). These data support the specific role of AKT in promoting JUNB phosphorylation and nuclear translocation within the QSOX2-mediated resistance pathway. To further verify whether JUNB is involved in QSOX2-mediated OR, we assessed the IC50 of osimertinib in JUNB-silenced H1650 cells using the CCK8 assay. The results showed that the IC50 value was significantly reduced in the si-JUNB group (12.79 nM) compared with the si-NC (20.22 nM) and blank control (18.94 nM) groups, indicating that JUNB plays a critical role in the development of resistance (Fig. 8F). Subsequently, we conducted rescue experiments in QSOX2-flag/Vector-flag cells with JUNB knockdown. CCK8 assay results revealed that the IC50 value in the QSOX2-flag + si-JUNB group (43.50 nM) was markedly lower than that in the QSOX2-flag + si-NC group (455.30 nM), suggesting that silencing JUNB can reverse QSOX2-mediated resistance (Fig. 8G).

To investigate whether FAK activation is sufficient to promote QSOX2-mediated OR, we first confirmed a direct protein interaction between ITGB4 and FAK by Co-IP (Supplementary Fig. 3B). In H1650 cells with ITGB4 knockdown, administration of the FAK agonist ZINC40099027 significantly restored phosphorylation of FAK and its downstream target AKT. Consistent with these findings, ZINC40099027 treatment partially reversed the decreased osimertinib IC50 caused by ITGB4 knockdown (Supplementary Fig. 3C–E). We next evaluated whether pharmacological inhibition of FAK or AKT could counteract QSOX2-driven OR. In H1975 cells, the FAK inhibitor defactinib (VS-6063) markedly reduced levels of p-FAK and p-AKT (Supplementary Fig. 4A), while the AKT inhibitor capivasertib (AZD5363) effectively suppressed p-AKT signaling (Supplementary Fig. 4B). CCK-8 assays demonstrated that both inhibitors significantly reduced the osimertinib IC50 and restored drug sensitivity in H1975-Vector-flag and H1975-QSOX2-flag cells (Supplementary Fig. 4C, D). Similar resensitization effects were observed in QSOX2-OE LUADOs following treatment with FAK or AKT inhibitors (Supplementary Fig. 4E–G). Furthermore, dual knockdown of QSOX2 and ITGB4 resulted in greater enhancement of OS compared to single knockdown of either gene alone (Supplementary Fig. 4H, I), indicating that simultaneous targeting of both QSOX2 and ITGB4/FAK/AKT signaling may provide superior therapeutic efficacy. Collectively, these results demonstrate that the QSOX2/JUNB-ITGB4 axis drives OR through FAK-AKT pathway activation, which is further sustained by a p-AKT/p-JUNB-mediated positive feedback loop.

### High expression of the QSOX2/JUNB-ITGB4 signaling axis in OR PDXs, LUADOs

**Fig. 9:**
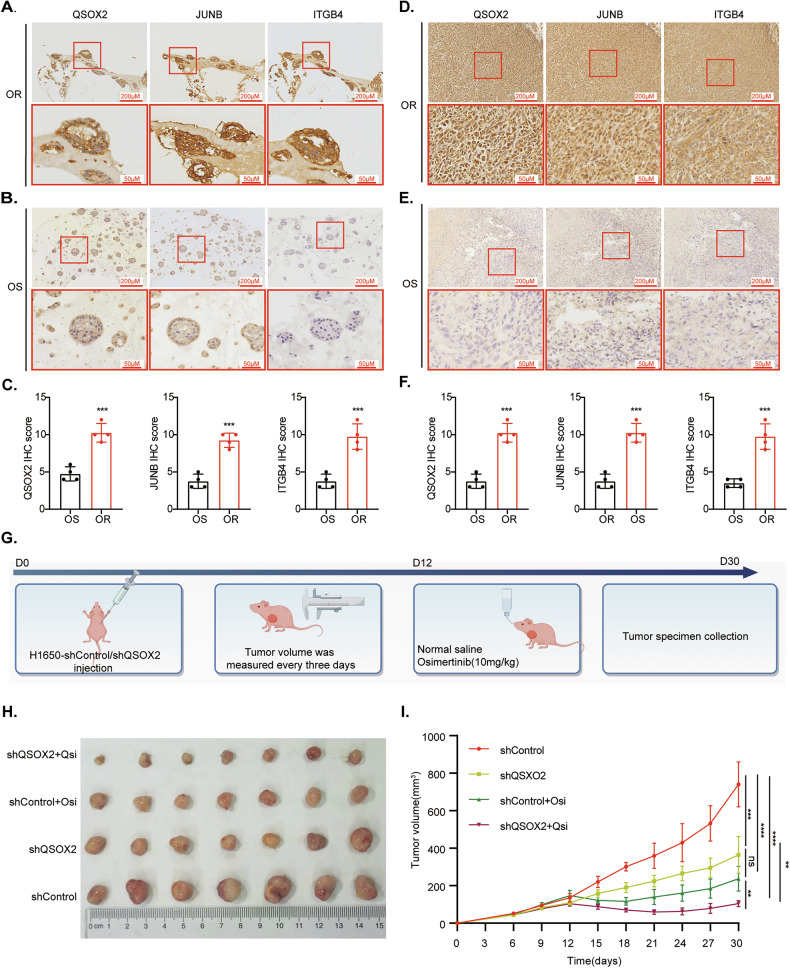
QSOX2/JUNB-ITGB4 signaling axis is highly expressed in drug-resistant LUADOs, PDXs. **A** IHC validation of QSOX2/JUNB-ITGB4 expression in OR and OS group (**B**) LUADOs. Enlarged views in red boxes highlight QSOX2/JUNB-ITGB4 staining intensities in specific regions (10X, 40X). **C** Quantitative statistical graph of IHC between organoids in the OS and OR groups. **D** IHC verifies the expression of QSOX2/JUNB-ITGB4 signaling axis in the OR and OS (**E**) group of PDXs. Enlarged views in red boxes highlight QSOX2/JUNB-ITGB4 staining intensities in specific regions (10X, 40X). **F** Quantitative statistical graph of IHC between PDXs in the OS and OR groups, by two-tailed t-test. **G** Schematic timeline of the xenograft experiment. **H** Representative images of excised tumors from the four experimental groups: shControl, shQSOX2, shControl + osimertinib, and shQSOX2 + osimertinib. On the 12th day, osimertinib 10 mg/kg and normal saline were administered orally to the treatment group and the control group, respectively, for 5 consecutive times. **I** Tumor growth curves showing volume changes over time in the four experimental groups. Data represent mean ± SD (*n* = 7 mice per group).; two-way ANOVA with Bonferroni post-hoc test. Data are represented as the mean ± SEM value from three biological replicates. ****P < 0.0001***P < 0.001, **P < 0.01, *P < 0.05.

In this study, we collected samples from 12 LUAD patients who were treated with third-generation EGFR-TKIs at the First Affiliated Hospital of Nanchang University between 2023.1 to 2024.1, including 9 pleural effusion samples and 3 biopsy specimens. Among these cases, EGFR mutations included exon 19 deletion (19del) in 6 patients, exon 21 L858R mutation in 4 patients, and EGFR T790M mutation in 2 patients (Supplementary Table 9). All 12 cases had been treated with third-generation EGFR-TKIs, of which 4 were OR patients. Supplementary Fig. 5A shows the growth process of LUADOs. Once the LUADOs reached a diameter of over 200 µm, samples were collected for passaging and further histological validation of the LUADOs was performed (Supplementary Fig. 5B). To further confirm that the cultured organoids were derived from LUAD, IHC for LUAD diagnostic markers (Napsin A, TTF1, CK7) was conducted (Supplementary Fig. 5C). HE staining was performed on both the OR and OS PDXs models (Supplementary Fig. 5D). IHC staining of LUADOs models from the OR (Fig. 9A) and OS group (Fig. 9B) was also performed to validate the expression of the QSOX2/JUNB-ITGB4 signaling axis. Compared with the OS group, the expression of the QSOX2/JUNB-ITGB4 signaling axis was significantly elevated in the OR group (Fig. 9C). In PDX models, EGFR mutations included 19del in 4 cases, L858R in 3 cases, and T790M in 2 cases (Supplementary Table 10). IHC staining was performed to evaluate the expression of the QSOX2/JUNB-ITGB4 signaling axis in both OR (Fig. 9D) and OS (Fig. 9E) PDX models. In the OR group, expression of the QSOX2/JUNB-ITGB4 signaling axis was markedly elevated compared to the OS group (Fig. 9F). To evaluate the translational potential of QSOX2 targeting, we established stable QSOX2-knockdown cell lines (H1650-shQSOX2) with corresponding controls (H1650-shControl) (Supplementary Fig. 5E). In a nude mouse xenograft model, QSOX2 knockdown significantly suppressed tumor growth compared to the shControl group. Furthermore, shQSOX2 demonstrated a synergistic antitumor effect when combined with osimertinib treatment (Fig. 9G–I). Collectively, these findings demonstrate that the QSOX2/JUNB-ITGB4 signaling axis mechanistically drives OR in both patient-derived LUADOs and PDX models, highlighting its potential as a druggable target for overcoming EGFR-TKI resistance in clinical oncology.

## Discussion

Despite osimertinib offering significant survival benefits for patients with EGFR-sensitive mutations, resistance inevitably develops, and the mechanisms of resistance remain unclear in approximately half of the patients [40, 41]. Therefore, it is crucial to understand novel drivers of OR, which may reveal potential new therapeutic targets to improve the prognosis of resistant patients. In this study, we discovered that QSOX2 was specifically upregulated in LUAD parenchyma. Subsequent stable overexpression of QSOX2 significantly enriched the classical resistance pathway PI3K/AKT [3, 42]. Our results revealed that the IC50 value of osimertinib increased 24.36-fold following QSOX2-OE, and a significantly shortened PFS was observed in patients with QSOX2-OE. For the first time, our findings clearly defined the adverse prognostic role of QSOX2 in OR LUADs, contributing to a deeper and more comprehensive understanding of the tumor-promoting mechanism of QSOX2.

Integrin family proteins not only mediate multiple transmembrane signal transductions but also play a key role in the development and progression of EGFR-TKI resistance [43]. In our study, we observed significant upregulation of ITGB4 in QSOX2-OE stable cell lines through RNA-seq. As a transmembrane glycoprotein, ITGB4 mediates signaling between cells and the ECM [30, 44]. The extracellular domain forms complexes with surrounding cells and ECM components, providing adhesion points, while the intracellular domain interacts with FAK, activating downstream signaling pathways that regulate various cellular behaviors [31]. In this study, we demonstrated for the first time that ITGB4 promotes OR, which is positively regulated by QSOX2. Consistent with previous reports, we also found that ITGB4 activates FAK, which in turn induced AKT phosphorylation, ultimately leading to OR [43]. Notably, our study revealed concomitant coregulation of total FAK and p-FAK by upstream signaling alterations. This phenomenon likely stems from constitutive activation of the ITGB4-FAK axis during OR, which drives ECM remodeling to establish adhesion-dependent survival niches. Within these niches, ITGB4-FAK complexes maintain signaling fidelity through FAK auto-amplification, which is a feedforward mechanism sustaining pathway activation under therapeutic pressure [30].

Moreover, the activation of various bypass pathways, including the upregulation of the expression and activity of multiple kinases and proteases, also plays a significant role in OR through both enzymatic and non-enzymatic mechanisms [45–47]. QSOX2 plays a critical enzymatic role in disulfide bond formation, and an excess of disulfide bonds in specific contexts can contribute to cancer cell survival and poor prognosis [25, 48]. Similar to the role of protein disulfide isomerase in cancer [49, 50], QSOX2 may promote cancer cell proliferation, adhesion, and invasion through enzymatic regulation of disulfide bond formation or by modulating ECM proteins [24, 25]. In human growth regulation research, QSOX2 has been found to promote the nuclear translocation of STAT5B and regulate downstream genes, suggesting broader physiological functions of QSOX2 [37]. Recent studies further demonstrated that QSOX2 catalyzes TSC2 disulfide bond formation to drive esophageal cancer malignancy [38]. However, the non-enzymatic functions of QSOX2 have been largely overlooked. In this study, we innovatively uncovered a novel non-enzymatic mechanism by which QSOX2 induces OR through site-directed mutagenesis of critical catalytic residues in its Thioredoxin and EVR/AVR sulfhydryl oxidase domains. We observed significant upregulation of nuclear QSOX2 in patients with rapid disease progression following third-generation EGFR-TKI therapy, suggesting its potential involvement in transcriptional regulation. Mechanistically, QSOX2 directly binds to JUNB, forming a protein complex that stabilizes JUNB by inhibiting its proteasomal degradation. In contrast, cytosolic JUNB proteins unable to bind QSOX2 may undergo ubiquitin-mediated degradation [51]. Notably, OR mediated by QSOX2 persisted even after disruption of its enzymatic activity via catalytic site mutations. Furthermore, previous studies have shown that the MAPK/ERK pathway is involved in OR and can also mediate nuclear translocation [52, 53]. In this study, we found that the MEK inhibitor U0126 could not inhibit the level of p-JUNB in the nucleus, but instead led to abnormal activation of p-AKT, which may be related to the parallel crosstalk between the AKT and MAPK pathways [54, 55]. Conversely, AKT inhibition markedly reduced nuclear p-JUNB. Thus, QSOX2-mediated AKT hyperactivation promotes p-JUNB nuclear accumulation, establishing a self-reinforcing QSOX2/JUNB-ITGB4/FAK/AKT positive feedback loop that drives OR.

Organoids have emerged as powerful tools in cancer research due to their ability to mimic tumor complexity and heterogeneity more accurately than traditional 2D cell cultures [56]. In this study, we successfully established OS and OR LUADOs, which provided a relevant model to validate the clinical significance of the QSOX2/JUNB-ITGB4 axis. IHC analysis confirmed elevated expression of this axis in OR LUADOs. Furthermore, in QSOX2-OE organoid models, combined treatment with FAK and AKT inhibitors significantly restored OS, underscoring the clinical potential of targeting this pathway.

However, the lack of specific QSOX2 inhibitors limits our ability to fully explore its role in vivo and assess its potential for reversing or delaying EGFR-TKIs resistance. Future studies should focus on clarifying the pharmacokinetics of this pathway in vivo and exploring the development of QSOX2-targeted inhibitors as a feasible strategy to mitigate OR. Additionally, while the non-enzymatic functions of QSOX2 in regulating the QSOX2/JUNB-ITGB4 axis are central to OR, its enzymatic role in disulfide bond formation and protein folding may also contribute to maintaining cellular integrity and promoting survival under drug pressure. Understanding how these two functions collaborate could open new avenues for targeted therapeutic strategies. Additionally, it is crucial to explore whether QSOX2 contributes to third-generation EGFR-TKI resistance via IGFBP3-mediated mechanisms, as well as the potential involvement of cancer-associated fibroblasts in promoting drug resistance. Finally, although PDX models better recapitulate the in vivo microenvironment, they remain insufficient for fully elucidating the critical role of the tumor microenvironment in OR. Future studies should focus on testing novel inhibitors targeting this pathway in more advanced organoid models, which may accelerate clinical translation and improve therapeutic outcomes for patients with OR. The complexity of QSOX2-mediated resistance through JUNB and ITGB4 not only expands our understanding of OR in LUAD but also underscores the urgent need for therapeutic strategies targeting this pathway to improve treatment outcomes and patient prognosis.

**Fig. 10:**
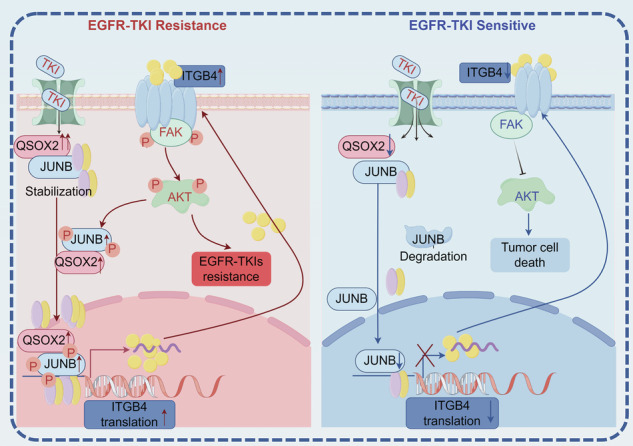
Mechanism of action of QSOX2/JUNB-ITGB4-FAK/AKT signaling axis in OR.

In conclusion, our findings demonstrated that QSOX2 is characteristically upregulated in LUAD and plays a critical role in the resistance to osimertinib. Mechanistically, QSOX2 engages in a non-enzymatic mechanism that interacts with JUNB, promoting its nuclear translocation, while JUNB acts as a TF to increase the expression of ITGB4, thereby facilitating OR through the FAK/AKT pathway (Fig. 10). Although we have elucidated and validated the biological significance of the QSOX2/JUNB-ITGB4-FAF/AKT signaling axis in contributing to OR, further large-scale, multicenter clinical studies are required to confirm the utility of QSOX2 as a biomarker for monitoring third-generation EGFR-TKI resistance in LUAD patients and as a novel therapeutic target.

## Methods

### Single-cell data acquisition, processing, and analysis

The single-cell LUAD dataset (GSE131907) [57] was obtained from the Gene Expression Omnibus (GEO; https://www.ncbi.nlm.nih.gov/geo/). The analyses were conducted using the “Seurat” package in R (version 4.3.0). The number of detected genes per single cell and the total molecule count were calculated. Due to the large size of the dataset, exclusion sampling analysis was performed (with each sample representing one-fifth of the remaining data, repeated for five iterations). Dimensionality reduction was conducted using t-distributed stochastic neighbor embedding (t-SNE). Initial manual annotation of each cluster was performed using “SingleR”. After initial annotation, copy number variation analysis was carried out to further distinguish normal cells from cancer cells (immune cells were used as references, and epithelial cells were analyzed; a high variant score indicated cancer cells, while a low variant score indicated normal epithelial cells). For a detailed analysis of the single-cell sequencing data, refer to previous reports [58].

### Cell culture, transfection and RNA sequencing (RNA-seq)

The human LUAD cell lines HCC827, NCI-H1975(H1975), PC9, and the normal lung epithelial cell line BEAS-2B were obtained from the Cell Bank of the Chinese Academy of Sciences (China). The NCI-H1650 (H1650) cell line was a kind gift from Procell (Wuhan, China). The human embryonic kidney HEK-293T (293T) cell line was acquired from the American Type Culture Collection (ATCC, Manassas, VA, USA). The human LUAD cell lines (HCC827, H1975, H1650, and PC9), the normal human lung epithelial cell line BEAS-2B, and the HEK-293T cell line were cultured as previously described [26]. The stable overexpression cell lines H1975-QSOX2-OE-flag (QSOX2-flag) and Vector-flag, cell were obtained from Zhonghong Boyuan Biotechnology Co., Ltd. (Nanchang, China), and RNA-seq was performed on two stable cell lines by Shanghai Applied Protein Technology Co., Ltd (Shanghai, China). All cell lines were cultured in a humidified incubator at 37 °C with 5% CO₂, using their recommended medium supplemented with 10% fetal bovine serum. Each cell line was authenticated by short tandem repeat (STR) profiling. The sequencing data have been deposited in the NCBI Sequence Read Archive (SRA) database under the accession code PRJNA1204999. Subsequent analyses focused on differentially expressed genes upregulated following QSOX2-flag, with KEGG-pathway enrichment analysis performed on these upregulated genes. Small interfering RNAs (siRNAs) targeting QSOX2, JUNB, and ITGB4, along with a nonspecific siRNA negative control, and the plasmid constructs for wild-type (WT) and mutant (MUT) QSOX2 were synthesized by Gene Pharma (Suzhou, China). All siRNA transfections were performed using jetPRIM transfection reagent (Polyplus, LOT#0000003107). The sequences of the siRNAs are listed in Supplementary Table 1. Transfection efficiency was evaluated using inverted fluorescence microscopy (Leica DMi8, Heidelberg, Germany), and the results were further validated by Western blot analyses.

### Enzyme-linked immunosorbent assay (ELISA)

Serum concentrations of QSOX2 and IGF1 were measured using commercial ELISA kits (QSOX2: BP05093; IGF1: BP01871) from Shanghai Baipeng Biotechnology Co., Ltd, according to the manufacturer’s instructions. Briefly, patient serum samples and standards were added to the antibody-precoated 96-well plates and incubated at 37 °C for 60 min. After washing, biotin-labeled detection antibody was added and incubated for 60 min, followed by incubation with enzyme conjugate for 30 min. The plates were then incubated with tetramethylbenzidine substrate for 10–20 min for color development. The reaction was stopped by the addition of the stop solution, and the absorbance was measured immediately. The concentrations of QSOX2 and IGF1 in the samples were calculated based on the standard curve.

### Measurement of hydrogen peroxide (H₂O₂) concentration

The concentration of H₂O₂ in cell samples was measured using a commercial Hydrogen Peroxide Assay Kit (Beyotime, Cat#S0038) following the manufacturer’s protocol. Briefly, cell lysates were prepared and incubated with the working solution. A standard curve was generated concurrently using the provided H₂O₂ standards. The absorbance was measured, and the H₂O₂ concentration in each sample was calculated based on the standard curve.

### Western blot analyses, RNA extraction, and RT-qPCR

Western blot, RNA extraction, and RT-qPCR were carried out as previously described [26]. The primers used in this study were designed (Supplementary Table 2) and synthesized by Shenggong Biotechnology Co., Ltd. (Shanghai, China). The antibody manufacturers, product numbers and dilution ratios used are shown in Supplementary Table 3. The FAK inhibitor Defactinib (Cat. No.VS-6063; HY-12289), the FAK agonist ZINC40099027 (Cat. No. HY-134570), the AKT inhibitor Capivasertib (AZD5363; Cat. No. HY-15431), and the MEK inhibitor U0126 (Cat. No. HY-12031A) were purchased from MedChemExpress (MCE). All compounds were dissolved in dimethyl sulfoxide (DMSO) to prepare stock solutions, which were aliquoted and stored at –20 °C until use. Working concentrations were diluted in cell culture medium immediately before experiments, with the final DMSO concentration maintained below 0.1% to avoid solvent toxicity.

### Flow cytometry

H1975-QSOX2-flag/Vector-flag cells were stained using APC-conjugated ITGB4 antibody (Immunoway; Catalog #YT2372) and APC-IgG antibody (Proteintech; Catalog #APC-65124). The expression of ITGB4 on the surface of the cells was detected by flow cytometry (DxFLEX, Beckman Coulter, Suzhou, China). Fluorescently labeled ITGB4 expression levels were measured, and at least 10,000 single cells were collected for each sample. All experiments were repeated at least three times.

### CCK-8 assay for determining the IC50 value of osimertinib

Cells were digested and resuspended, and then seeded into 96-well plates at a concentration of 5 × 10^3^ cells/mL, with 100 μL of culture medium added to each well. The cells were treated with osimertinib at varying concentrations (0 nM, 16 nM, 80 nM, 400 nM, 2000nM, 10000 nM) for 72 h. After treatment, 10% CCK-8 solution (APExBIO; Cat. No. K1018) was added to each well, followed by incubation for 1.5 h in a cell culture incubator. The absorbance was then measured at 450 nm using a microplate reader. The experiment was repeated three times, and the average value was calculated to determine the inhibition rate of osimertinib on tumor cells. Osimertinib (AZD9291) was purchased from Selleck (S729708).

### Establishment of stable organoid lines and osimertinib sensitivity assay

To generate stable QSOX2 overexpression (QSOX2-OE) organoids, pre-digested organoids were resuspended in a mixture of QSOX2-OE lentiviral particles (Genema, Suzhou, China), complete medium, and 6 μg/mL Polybrene. The organoid-virus mixture was aliquoted into 24-well plates and centrifuged at 300 × *g* for 1 h using a horizontal plate centrifuge to enhance viral infection. After centrifugation, the organoids were embedded in matrix and cultured in complete medium. Puromycin was added for selection, and the medium was refreshed every 2–3 days. Organoid growth and morphology were monitored daily.

Organoids were dissociated and seeded into 96-well plates at a density of 1500–2000 cells/well, ensuring uniformity in size and viability across replicates. Following plating, organoids were treated with the same gradient concentrations of osimertinib as applied to tumor cells for 72 h. Cell viability was subsequently measured using the CellTiter-Glo® 3D Cell Viability Assay (Promega, Cat#G9683) according to the manufacturer’s protocol. Luminescence signals were recorded to evaluate drug sensitivity.

### TF screening, ChIP assay

TFs regulating ITGB4 were screened using the Enrichment Analysis module of the ChIP-Atlas database (https://chip-atlas.org). The screening parameters were set as follows: Experiment type: “ChIP: TF and other”; Cell type Class: “Lung”; Threshold for Significance: 50 (peaks with a *Q* < 1 × 10⁻⁵ were used to assess overlap with the dataset). TFs with a fold enrichment greater than 100 were considered high-confidence candidates.

Cells were cultured in 15 cm culture dishes, and 540 μL of 37% formaldehyde was added, followed by gentle vortex mixing. The cells were incubated at room temperature for 10 min with 20 mL of culture medium in the dish. Subsequently, 2 mL of 10× glycine was added, and the mixture was incubated at room temperature for an additional 5 min. After discarding the culture medium and washing the cells twice with PBS, 2 mL of PBS containing protease inhibitor cocktail was added. The cells were then scraped and transferred to a 15 mL centrifuge tube. The suspension was centrifuged at 2000 × *g* for 5 min at 4 °C to pellet the cells. The procedure was performed according to the manufacturer’s instructions (Cell Signaling). The predicted binding sequences for the ITGB4 promoter and the primers used are listed in Supplementary Table 4. The specific steps are as described previously [26].

### Immunoprecipitation (IP) and Co-IP assays

To investigate the interaction between QSOX2 and ITGB4, H1975-QSOX2-flag cells were cultured in 10 cm dishes until approximately 80% confluence. Proteins were then extracted using IP lysis buffer containing protease inhibitors. To examine the interaction between QSOX2 and JUNB, the HA-JUNB plasmid was transfected into the H1975-QSOX2-flag cells. After 72 h, IP lysis buffer was added, and the lysates were incubated overnight at 4 °C with anti-Flag (Beyotime, P2215), anti-HA magnetic beads (Beyotime, P2121), and anti-IgG magnetic beads (Beyotime, P2108). The samples were subsequently washed three times with TBS. The IP and whole-cell lysates were analyzed by Western blot using the corresponding antibodies. A list of antibodies used in this study is provided in Supplementary Table 5.

### Dual-luciferase assay

The JUNB transcription binding sites in the ITGB4 promoter region were predicted using the Uniprot website (https://www.uniprot.org/). Based on the H352 pQL4.10 reporter vector, this study successfully constructed a series of plasmids containing the wild-type (WT) and targeted deletion mutants at the TBS1/TBS5 sites (MUT-TBS1, MUT-TBS5, and MUT-TBS1 + TBS5). Cells were then transfected with these luciferase reporter plasmids, and after 48 h, the cells were harvested. Luciferase activity was assessed using the Dual-Glo Luciferase system (Promega). All experiments were performed in triplicate.

### Cycloheximide (CHX) chase assay and MG132 proteasome inhibition assay

For the CHX (MCE, Cat. No. HY-12320) chase assay, cells were transfected according to the experimental design. At 48 h post-transfection, the culture medium was replaced with complete medium containing 50 μg/mL CHX. H1650 cell lysates were collected at 0, 6, 12, and 24 h after CHX treatment, while H1975-QSOX2-flag and Vector-flag control cell lysates were collected at 0, 1, 2, 3, and 6 h. JUNB protein half-life was analyzed by Western blot. For the proteasome inhibition assay, transfected H1650 cells were treated with 10 μM MG132 (MCE, Cat. No. HY-13259) in complete medium for 8 h at 48 h post-transfection. Cells were then harvested, and JUNB protein levels were assessed by Western blot analysis.

### Ethics statement and clinical specimens

The experimental protocol for the subcutaneous xenograft tumor model was approved by the Institutional Animal Care and Use Committee of Nanchang Leyo Biotechnology Co., Ltd. (Approval No. REY2024032501). All procedures were performed in strict accordance with the committee’s guidelines. The ethics approval number for the patient-derived xenograft (PDX) model is RYE2023011901.

Human LUAD tissues and serum samples used in this study were collected from patients treated with third-generation EGFR-TKIs at The First Affiliated Hospital of Nanchang University. Informed consent was obtained from all participants. The study protocol was approved by the Medical Ethics Review Committee of The First Affiliated Hospital of Nanchang University (Approval No. (2024) CDYFYYLK (10-005)), and all experiments were conducted in compliance with the committee’s ethical guidelines. Serum samples were additionally collected from 28 out of the 58 patients treated with third-generation EGFR-TKIs.

### Establishment of LUAD organoids (LUADOs) and PDX

LUAD tissue was thoroughly washed and cut into approximately 1 mm³ pieces using a scalpel. The tissue fragments were transferred to a centrifuge tube and treated with a digestion solution, with the extent of digestion monitored under a microscope. Digestion was stopped once cell clusters formed, as complete dissociation into single cells may compromise cell viability. After stopping the digestion, the sample was mixed thoroughly, filtered through a 100-mesh filter to obtain a suspension of cell clusters, and washed with Advanced/DEMEM/F12. Primary cells were then resuspended in Matrigel for plating. For malignant pleural effusion samples, tumor cells were collected after red blood cell lysis and then plated. LUADOs were passaged when they reached a diameter of 200 µm, as described in [59]. All LUADOs culture reagents were obtained from BioGenous Biotechnology Co., Ltd. (Suzhou, China). For the PDX models, 9 cases were sourced from the LeYou Biological PDX Biobank (Nanchang, China), including three OR and six osimertinib-sensitive (OS) models.

### Subcutaneous xenograft tumor model

A total of 28 female BALB/c nude mice (4 weeks old) were purchased from Nanchang LeYou Biotechnology Co., Ltd. and housed under specific pathogen-free conditions. To establish the xenograft model, H1650 cells were infected with lentivirus to generate stable QSOX2-knockdown (shQSOX2) and control (shControl) cell lines. After shaving the scapular region, each mouse was inoculated subcutaneously with H1650-shControl or shQSOX2 cells (2 × 10⁶ cells per mouse). When tumor volumes reached 30–50 mm³, mice with well-established and uniform tumors were randomly divided into two groups (*n* = 7 per group). Tumor volume was measured every three days using the formula: Volume = 0.5 × Length × Width². Starting from day 12 post-inoculation, the treatment group received oral gavage of osimertinib (10 mg/kg) daily for five days, while the control group received an equivalent volume of saline. After 30 days, mice were euthanized via cervical dislocation under anesthesia and subcutaneous tumors were harvested and measured.

### Hematoxylin and eosin (HE), immunohistochemistry (IHC) and immunofluorescence (IF) staining

LUADOs, PDX and tissue slices were fixed in 4% paraformaldehyde, followed by routine dehydration and paraffin embedding to prepare 3 μm thick sections. After routine dewaxing, the sections were stained with HE and sealed with neutral gum. The IHC antibody manufacturers, product numbers and dilution ratios used are shown in SupplementaryTable 3. The IHC slides were independently evaluated by two pathologists, who were blinded to the samples. The intensity and percentage of positive staining for QSOX2, JUNB, and ITGB4 were recorded. IHC scoring was performed using the semi-quantitative scoring method described by Germany [60], as previously outlined [26]. The slides were examined under an optical microscope (Leica DM2000, Heidelberg, Germany). IF staining was performed by incubating cells overnight at 4 °C with antibodies against QSOX2, JUNB, and ITGB4. The cells were then incubated for 1 h with secondary antibodies, donkey anti-rabbit IgG (H + L) Alexa Fluor 555 and goat anti-mouse IgG (H + L) Alexa Fluor 488. The nuclei were counterstained with 4′,6-diamidino-2-phenylindole (DAPI). All stained cells were imaged using an inverted fluorescence microscope (Leica DMi8, Heidelberg, Germany). At least three independent experiments were performed.

### Statistical analysis

All statistical analyses were conducted using GraphPad Prism v.8.0 software. Normality tests were performed on all quantitative data, which are presented as the mean ± standard deviation (mean ± SD). One-way analysis of variance (ANOVA) was used for comparisons among multiple groups, followed by LSD post hoc tests for pairwise comparisons. For comparisons between two groups, a t-test was applied. A *P*-value of less than 0.05 was considered statistically significant.

## Supplementary information

Supplementary material

Raw data

## Data Availability

The sequencing data have been deposited in the NCBI Sequence Read Archive (SRA) database under the accession code PRJNA1204999. The relevant data can be obtained by contacting the corresponding author.
