# Supplementary material for: Non-enzymatic function of QSOX2 directly regulates the JUNB-ITGB4 axis and enhanced resistance to osimertinib in EGFR-mutation lung adenocarcinoma
Source: Cell Death Discov. 2026 Apr 1;12:215. doi: 10.1038/s41420-026-02969-4 (PMC13168330; doi:10.1038/s41420-026-02969-4)
Supplement: Supplementary file 1 — Supplementary material [file 41420_2026_2969_MOESM1_ESM.docx]

**Supplementary Material**

**Supplementary Figures**

**
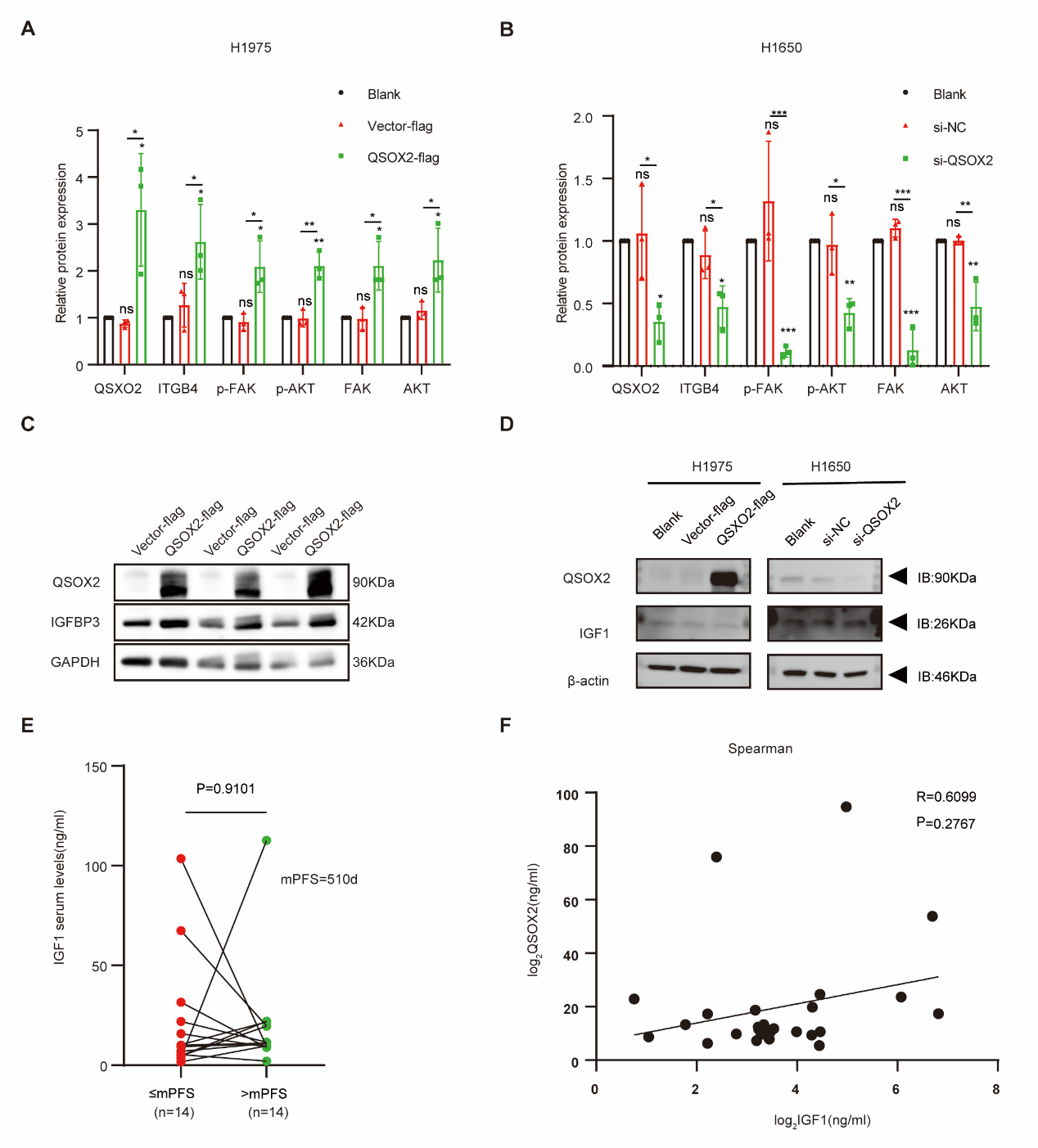
Supplemental Figure 1. The growth hormone/IGF1 pathway is not involved in QSOX2-mediated resistance to third-generation EGFR-TKIs. ​A.** **Quantification of protein levels in H1975 cells from the experiment shown in Figure 3E. Cells were either untransfected (Blank), transfected with a control vector (Vector-flag), or a QSOX2 overexpression vector (QSOX2-flag). B. Quantification of protein levels in H1650 cells from the experiment shown in Figure 3E. Cells were treated as follows: Blank, si-NC, or si-QSOX2. For both panels, expression levels of QSOX2, ITGB4, p-FAK, FAK, p-AKT, and AKT were analyzed. Protein levels were normalized to GAPDH and are presented as relative expression. Data are shown as mean ± SD from three independent experiments. Statistical significance was determined by one-way ANOVA followed by Tukey's post hoc test. **P* < 0.05, ***P* < 0.01, ****P* < 0.001, *****P* < 0.0001; ns, not significant. C. Western blot analysis of QSOX2 and IGFBP3 expression in H1975-Vector/QSOX2-flag cells. GAPDH was used as a loading control. D. Western blot analysis of QSOX2 and IGF1 expression in H1975/H1650 cells. β-actin was used as a loading control. E. Kaplan–Meier survival analysis of progression-free survival based on serum IGF1 levels** (*P* = 0.9101)**. F. Correlation analysis between QSOX2 and IGF1 serum expression (*P* = 0.2767, *R* = 0.6099).** Statistical analysis was performed using **two-tailed t-test.**

**
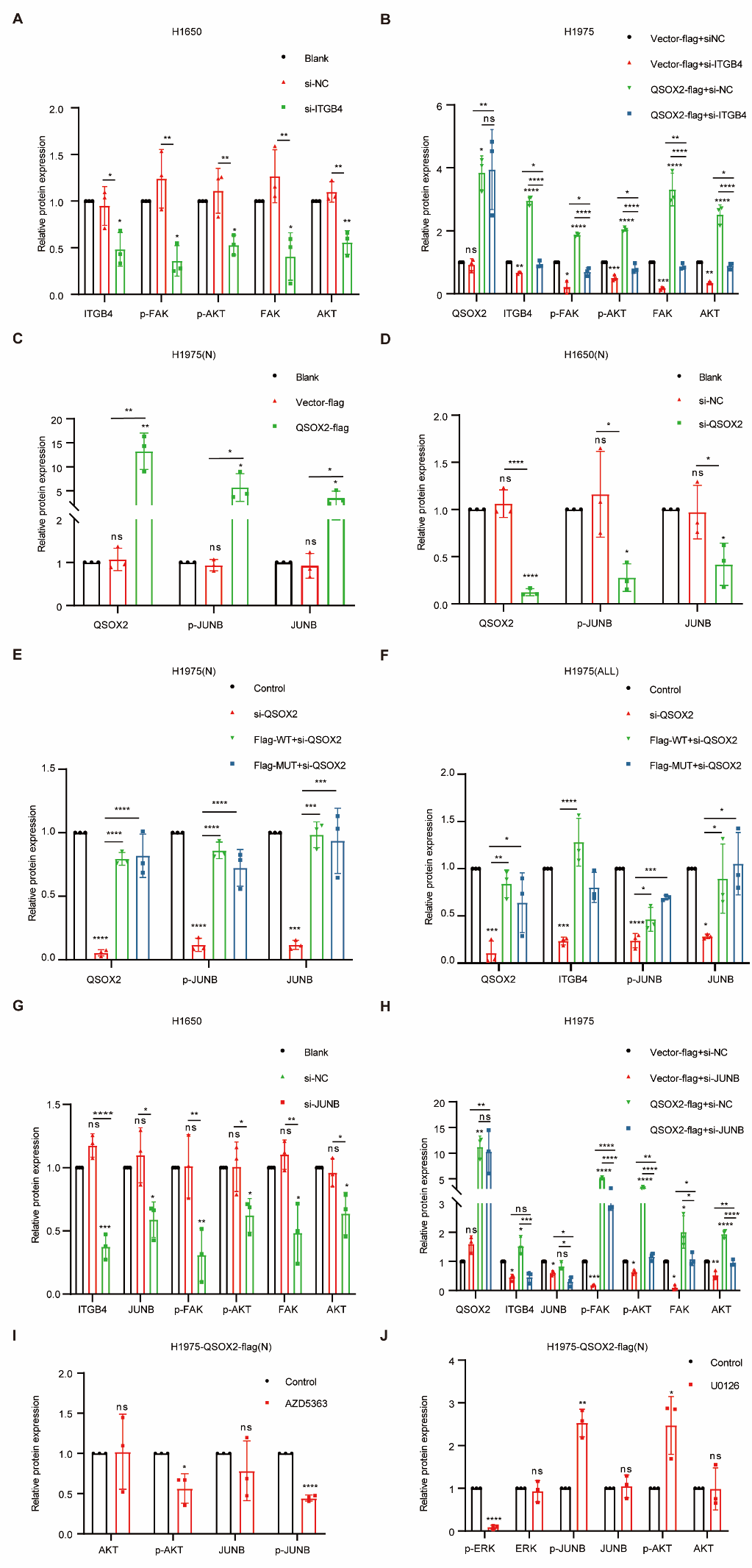
**

**Supplementary Figure 2. Densitometric quantification of Western blot results presented in Figures 4B, 4C, 6G, 7C, 7D, 8C, 8D,** **8E, and S3A. A.​****Quantification of protein levels in H1650 cells from the experiment shown in Figure 4B. Cells were treated as follows: untransfected (Blank), transfected with a non-targeting control siRNA (si-NC), or a targeting siRNA against ITGB4 (si-ITGB4). Expression levels of ITGB4, p-FAK, FAK, p-AKT, and AKT were analyzed. B. Quantification of protein levels in H1975 cells from the experiment shown in Figure 4C. Cells transfected with either a control vector (Vector-flag) or a QSOX2 overexpression vector (QSOX2-flag) were co-transfected with si-NC) or si-ITGB4. Expression levels of QSOX2, ITGB4, p-FAK, FAK, p-AKT, and AKT were analyzed. C. Quantification of nuclear​ protein levels in H1975 cells from the experiment shown in Figure 6G. Cells were treated as follows: Blank, Vector-flag, or QSOX2-flag. Expression levels of QSOX2, phosphorylated JUNB (p-JUNB), and total JUNB were analyzed. D. Quantification of nuclear​ protein levels in H1650 cells from the experiment shown in Figure 6G. Cells were treated as follows: Blank, si-NC, or si-QSOX2. Expression levels of QSOX2, p-JUNB, and JUNB were analyzed. E. Quantification of nuclear protein levels in H1975 cells from the experiment shown in Figure 7C.​Cells were transfected as follows: si-NC, si-QSOX2, or co-transfected with si-QSOX2 and a flag-tagged wild-type (Flag-WT) or mutant (Flag-MUT) QSOX2 overexpression plasmid. Expression levels of QSOX2, p-JUNB, and JUNB were analyzed. F. Quantification of total protein levels in H1975 cells from the experiment shown in Figure 7D.​ Cells were treated as described for panel (E). Expression levels of QSOX2, ITGB4, p-JUNB, and JUNB were analyzed. G. Quantification of protein levels in H1650 cells from the experiment shown in Figure 8C. Cells were treated as follows: Blank, si-NC, or si-JUNB. Expression levels of ITGB4,** **JUNB, p-FAK, FAK, p-AKT, and AKT were analyzed. H. Quantification of protein levels in H1975 cells from the experiment shown in Figure 8D. Cells transfected with Vector-flag or QSOX2-flag were co-transfected with si-NC or si-JUNB. Expression levels of QSOX2,** **ITGB4, JUNB, p-FAK, FAK, p-AKT, and AKT were analyzed.** **I. Quantification of nuclear protein levels in H1975-QSOX2-flag cells from the experiment shown in Figure 8E.​ Cells were treated with Control (DMSO) or the AKT inhibitor (AZD5363 2μM). Expression levels of AKT, p-AKT, JUNB, and p-JUNB were analyzed. J. Quantification of nuclear protein levels in H1975-QSOX2-flag cells from the experiment shown in Figure S3A. Cells were treated with Control (DMSO) or the MEK inhibitor (U0126 10μM). Expression levels of ERK, p-ERK, JUNB, p-JUNB, AKT, and p-AKT were analyzed. Data are presented as the mean ± SD; Statistical analysis was performed using one-way ANOVA (for multiple groups) or Student's t-test (for two groups). **P* < 0.05, ***P* < 0.01, ****P* < 0.001, *****P* < 0.0001; ns, not significant.**

**
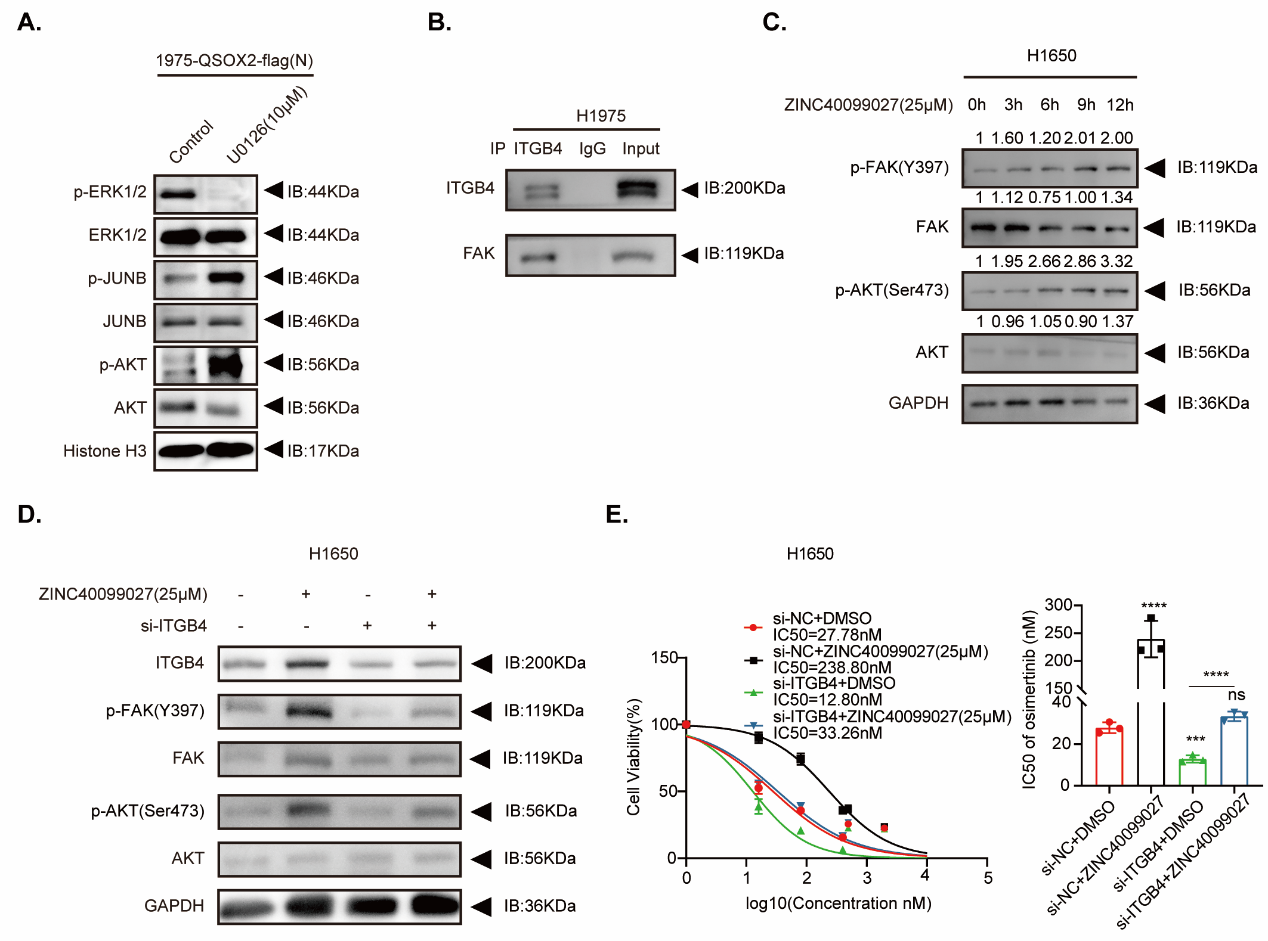
**

**Supplemental Figure 3. FAK agonist ZINC40099027 activates FAK/AKT signaling and partially reverses ITGB4 knockdown-induced OS. A.** Western blot analysis of nuclear extracts from H1975-QSOX2-Flag cells treated with Control (DMSO) or the MEK inhibitor (U0126 10μM). The levels of total ERK, p‑ERK, total JUNB, p‑JUNB, AKT, p‑AKT, and the nuclear loading control Histone H3 are shown. **B.** Co-IP analysis of ITGB4-FAK interaction in H1975 cells. Whole cell lysates were immunoprecipitated with ITGB4 antibody, followed by immunoblotting for FAK.**C.** Time-course analysis of FAK/AKT pathway activation in H1650 cells treated with ZINC40099027 (25μM). Phosphorylation levels of FAK (Tyr397) and AKT (Ser473) were assessed by Western blot. **D.** Western blot analysis of ITGB4, phospho-FAK (Tyr397), phospho-AKT (Ser473), and GAPDH in H1650 cells under indicated treatment conditions. ZINC40099027 (25μM) treatment enhanced phosphorylation of FAK and AKT. GAPDH served as loading control. **E.** Dose-response curves of osimertinib in H1650 cells under four conditions: si-NC + DMSO (IC50 = 27.78nM), si-NC + ZINC40099027 (IC50 = 238.80nM), si-ITGB4 + DMSO (IC50 = 12.80nM), and si-ITGB4 + ZINC40099027 (IC50 = 33.26nM). Cell viability was measured after 72 hours of treatment. Data represent mean ± SD from three independent experiments; *P < 0.05, **P < 0.01, ****P* < 0.001; ns, not significant (one-way ANOVA with Tukey's post-hoc test).

**
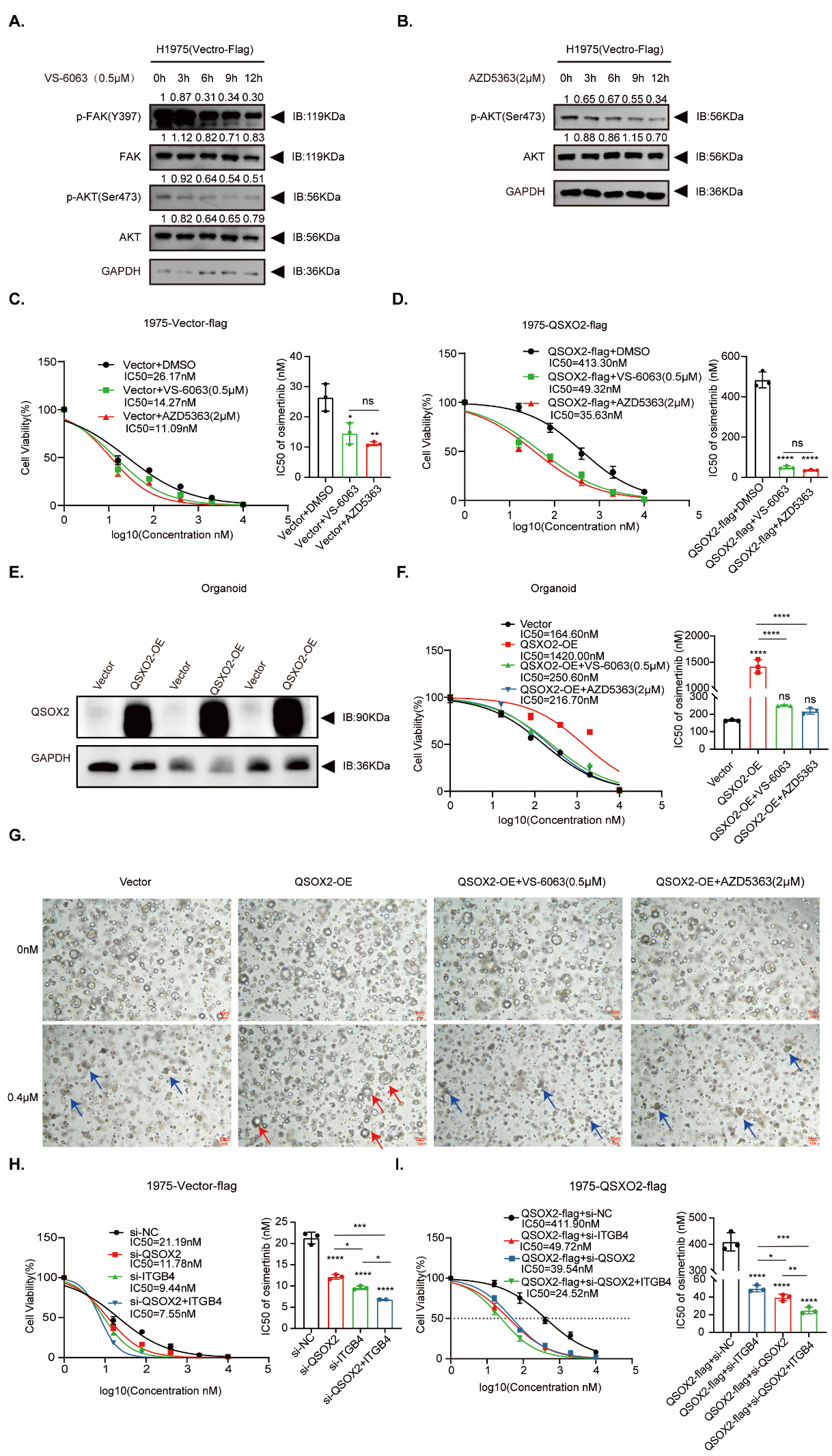
**

**Supplemental Figure 4. FAK/AKT signaling is essential for QSOX2-mediated OR. A.** Western blot analysis of p-FAK (Tyr397), total FAK, p-AKT (Ser473), and total AKT in H1975 cells treated with the FAK inhibitor VS-6063 (0.5μM) for indicated time periods. GAPDH serves as loading control. **B.** Western blot analysis of p-AKT (Ser473) and total AKT in H1975 cells treated with the AKT inhibitor AZD5363 (2μM) for indicated time periods. GAPDH serves as loading control. **C.** Dose-response curves of osimertinib in H1975-Vector cells treated with DMSO (IC50 = 26.17nM), VS-6063 (IC50 = 14.27nM), or AZD5363 (IC50 = 11.09nM). Cell viability was measured after 72 hours of treatment. **D.** Dose-response curves of osimertinib in H1975-QSOX2-flag cells pretreated with DMSO (IC50 = 413.30nM), VS-6063 (IC50 = 49.32nM), or AZD5363 (IC50 = 35.63nM). Cell viability was measured after 72 hours of treatment. Data points represent mean ± SD from three independent experiments. **E.** Western blot analysis confirming QSOX2 overexpression in LUADOs. GAPDH serves as loading control. **F.** Dose-response curves of osimertinib in LUADOs under four conditions: Vector control (IC50= 164.60nM), QSOX2-OE (IC50 = 1420.00nM), QSOX2-OE + VS-6063 (IC50 = 250.60nM), and QSOX2-OE + AZD5363 (IC50 = 216.70nM). Cell viability was measured after 72 hours of treatment. **G.** Representative bright-field images of LUAD organoids treated with osimertinib at 0nM and 400nM under indicated conditions. Red arrows indicate that organoids maintain activity and normal morphology; blue arrows indicate that organoids shrink and undergo apoptosis. Scale bar: 50μm. **H.** Dose-response curves of osimertinib in H1975-Vector-flag cells under five treatment conditions: non-targeting control (si-NC, IC50 = 21.19nM), QSOX2 knockdown (si-QSOX2, IC50 = 11.78nM), ITGB4 knockdown (si-ITGB4, IC50 = 9.44nM), and combined QSOX2/ITGB4 knockdown (si-QSOX2+ITGB4, IC50 = 7.55nM). Cell viability was measured after 72 hours of treatment. **I.** Dose-response curves of osimertinib in H1975-QSOX2-flag cells under four treatment conditions: non-targeting control (si-NC, IC50 = 411.90nM), ITGB4 knockdown (si-ITGB4, IC50 = 49.72nM), QSOX2 knockdown (si-QSOX2, IC50 = 39.54nM), and combined QSOX2/ITGB4 knockdown (si-QSOX2+ITGB4, IC50 = 24.52nM). Cell viability was measured after 72 hours of treatment. Data represent mean ± SD from three independent experiments; *P < 0.05, **P < 0.01, ****P* < 0.001; ns, not significant (one-way ANOVA with Tukey's post-hoc test).

**
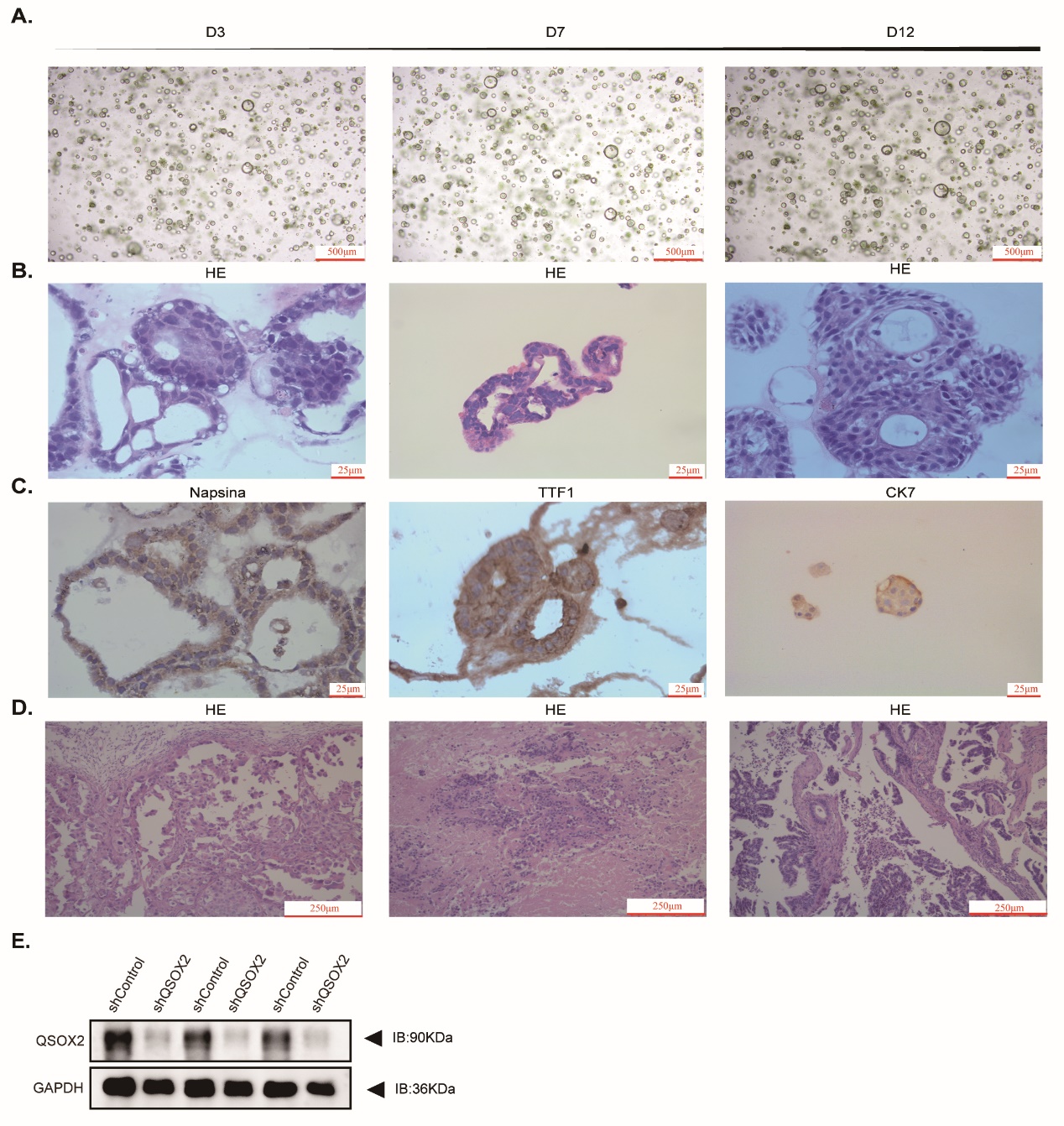
**

**Supplemental Figure 5. Growth and histological validation of LUADOs and PDX. A. Bright field image of LUADOs growth, 5X. B. HE-stained sections of organoids, 40X. C. Histological validation of characteristic markers of LUAD, Napsina, TTF1, and CK7, 40X. D.** HE staining of LUAD PDX, 4X. **E.** Western blot analysis confirming QSOX2 protein expression in H1650 cells stably transduced with control shRNA (shControl) or QSOX2-targeting shRNA (shQSOX2). GAPDH serves as a loading control.

**Supplementary Tables**

**Supplementary Table 1.** The sequences of the siRNA (QSOX2, JUNB, ITGB4, NC)

| **Gene** | **Sense**（**5'-3'**） | **Antisense**（**5'-3'**） |
| --- | --- | --- |
| siRNA-QSOX2-1 | GCAGCCAUUACGUGGCUAUTT | AUAGCCACGUAAUGGCUGCTT |
| siRNA-QSOX2-2 | GGUACGUUCACACCUUCUUTT | AAGAAGGUGUGAACGUACCTT |
| siRNA-QSOX2-3 | GCUGUGGAAGAAGCAUAAUTT | AUUAUGCUUCUUCCACAGCTT |
| siRNA-JUNB-1 | ACCAACCUCAGCAGCUACUTT | AGUAGCUGCUGAGGUUGGUTT |
| siRNA-JUNB-2 | ACAAGGUGAAGACGCUCAATT | UUGAGCGUCUUCACCUUGUTT |
| siRNA-JUNB-3 | UCAUGACCCACGUCAGCAATT | UUGCUGACGUGGGUCAUGATT |
| siRNA-ITGB4-1 | CCAGGAAGAUCCAUUUCAATT | UUGAAAUGGAUCUUCCUGGTT |
| siRNA-ITGB4-2 | GCACGUGUGAGGAAUGCAATT | UUGCAUUCCUCACACGUGCTT |
| siRNA-ITGB4-3 | GGACUACGACAGCUUCCUUTT | AAGGAAGCUGUCGUAGUCCTT |
| siRNA-NC | UUCUCCGAACGUGUCACGUTT | ACGUGACACGUUCGGAGAATT |

**Supplementary Table 2.** The primer of QSOX2, JUNB, ITGB4, GAPDH

| **Gene** | **Forward** | **Reverse** |
| --- | --- | --- |
| JUNB | CTTCTACCACGACGACTCATACAC | GGCTCGGTTTCAGGAGTTTGTAG |
| GAPDH | CACCAGGGCTGCTTTTAACTCTG | GATTTTGGAGGGATCTCGCTCCTG |
| QSOX2 | ATGAGTCTCTGTGTCGTGCTGTAC | GGTGGTGGTGCTTGACCTTCC |
| ITGB4 | ATCGTGGAGCTGCTGGAGGAG | CGTCTTCTGGAACATCTTGGAGGTG |

**Supplementary Table 3.** Antibody manufacturers and product number involved in WB and IHC/IF

| **Antibody** | **Manufacturer** | **Product Number** | **WB Dilutions** | **IHC/IF Dilutions** |
| --- | --- | --- | --- | --- |
| QSOX2 | Abcam | Catalog#ab121376 | 1:1,000 | 1:200 |
| JUNB | Proteintech | Catalog#10486-1-AP | 1:1,000 | 1:200 |
| p-JUNB | ABMART | Catalog#TA2354 | 1:500 | / |
| ITGB4 | Boster Biological Technology | Catalog#A01015-2 | 1:1,000 | 1:200 |
| FAK | Huabio | Catalog# ET1602-25 | 1:5,000 | / |
| p-FAK | Huabio | Catalog# ET1610-34 | 1:500 | / |
| AKT | Huabio | Catalog# JE75-09 | 1:5,000 | / |
| p-AKT | Huabio | Catalog# ET1607-73 | 1:2,000 | / |
| ERK1/2 | Abways | Catalog No.: CY5487 | 1:1,000 |  |
| p-ERK1/2 | Abways | Catalog No.: CY5277 | 1:1,000 |  |
| TTF1 | Immunoway | Catalog No.：YM7238 | / | 1:200 |
| NapsinA | Immunoway | Catalog No：YM4509 | / | 1:200 |
| CK7 | Immunoway | Catalog No：YM3054 | / | 1:200 |

**Supplementary Table 4.** The predicted binding sequences for the ITGB4 promoter and the primers

| **ITGB4 primers/Promoter** | **Forward** | **Reverse** | **Promoter binding sequence** |
| --- | --- | --- | --- |
| +185~+196 | aagcctcagcccccaccc | ttctgcacagagaaagtgttcag | AAGCCTCAGCCCCCACCCACAGTGGGGTGTATATGACCCCCTAGTTCTCTAATGGGTGCTGCTGGCAGACCCAGGCCCCTCAGGCAAGCTGTTTTTGGCCGTTCTCTGAGCTCCTGCTCCATGTTTCTGTTTTTTGGGGTGGATAGGTACCTGCTGGAGTGACTCAGGTCCACAGGGCACTTGTGCTGGCCTGACACACACAGATCTGAGTGCTTCAACCAAACTGGGACTCCTTCCACATTGTTCCCAAATCCAGGAGCAAGCCTCTCTGAACACTTTCTCTGTGCAGAA |
| -1932~-1942 | tagccgatcggggcgctg | ggcggactcgctccctc | TAGCCGATCGGGGCGCTGGGCGGGCGCCGCGGGAGCCGCAGCCCTTTCCGGGGGGCGGACCCGGCTCCGGCGGCGGCACCCAGCTCCTGCCCCGACAGGTGCGCGCCGCGCGAAGGAATGCAGCCGGTCTGACTCACCAGCGCCTCCTTCCTACCTGCGCGCCCGCCCCATAAAGCGCTGCCCGCCTCGTCCCCACCCCCCCAACCCCCGCGCCCGCCCTCGGACAGTCCCTGCTCGCCCGCGCGCTGCAGCCCCATCTCCTAGCGGCAGCCCAGGCGCGGAGGGAGCGAGTCCGCC |

**Supplementary Table 5.** Antibody manufacturers and part number involved in IP and Co-IP

| **Antibody** | **Manufacturer** | **Part Number** |
| --- | --- | --- |
| Flag | Proteintech | Catalog#20543-1-AP |
| HA | Proteintech | Catalog#51064-2-AP |
| QSOX2 | Abcam | Catalog#ab121376 |
| SOX2 | Huabio | Catalog#HA721155 |
| SNAI2 | Huabio | Catalog#EM1706-65 |
| MLLT1 | Proteintech | Catalog#31029-1-AP |
| JUNB | Proteintech | Catalog#10486-1-AP |
| TEAD1 | Huabio | Catalog#HA722144 |

**Supplementary Table 6. Association between QSOX2 expression and clinicopathological characteristics in patients treated with third-generation EGFR-TKIs (n=58).​​**

| Characteristic | | QSOX2(n=58) | | *P value* |
| --- | --- | --- | --- | --- |
|  |  | Low  Expression (%) | High  Expression (%) |  |
| **Gender** | Male | 14(24.14) | 13(22.41) |  |
|  | Female | 15(25.86) | 16(27.59) | 1.000 |
| **Age(years)** | ≤60 | 16(27.59) | 17(29.31) |  |
|  | ＞60 | 13(22.41) | 12(20.90) | 1.000 |
| **T stage** | T1-2 | 24(41.379) | 12(20.69) |  |
|  | T3-4 | 5(8.62) | 17(29.31) | **0.003** |
| **N stage** | N0-1 | 16(27.59) | 7(12.07) |  |
|  | N2-3 | 13(22.41) | 22(37.93) | **0.031** |
| **M stage** | M0 | 2(3.45) | 4(6.90) |  |
|  | M1a-c | 27(46.55) | 25(43.10) | 0.67 |
| **Smoking history** | Yes | 2(3.45) | 7(12.07) |  |
|  | No | 27(46.55) | 22(37.93) | 0.144 |
| **Drinking history** | Yes | 1(1.72) | 6(10.34) |  |
|  | No | 28(48.28) | 23(39.66) | 0.764 |

**Supplementary Table 7.** ITGB4 potential transcription factor

| **ID** | **Antigen class** | **Antigen** | **Cell class** | **Cell** | **Num of peaks** | **Overlaps/Control** | **Fold Enrichment** | **FE >1?** |
| --- | --- | --- | --- | --- | --- | --- | --- | --- |
| SRX277137 | TFs | SOX2 | Lung | HCC95 | 1208 | 46/18862 | 410.04 | TRUE |
| SRX5887616 | TFs | SNAI2 | Lung | PC-9 | 952 | 79/18862 | 238.76 | TRUE |
| SRX10131668 | TFs | MLLT1 | Lung | Bronchial tracheal epithelial cells | 1000 | 185/18862 | 101.96 | TRUE |
| **SRX8616845** | **TFs** | **JUNB** | **Lung** | **A549** | **2062** | **187/18862** | **100.87** | **TRUE** |
| SRX9163074 | TFs | TEAD1 | Lung | Bronchial epithelial cells | 2479 | 187/18862 | 100.87 | TRUE |

**Supplementary Table 8.** Deletion mutation site sequence and prediction score

| **Presumptive ITGB4 binding site** | **Position** | **Deletion mutation sequenceSequence** | **Score** |
| --- | --- | --- | --- |
| **TBS-1** | **-1932~-1942** | **tggtgagtcag** | **14.746123** |
| TBS-2 | -418~-427 | atgctgtcat | 9.234296 |
| TBS-3 | -344~-355 | tgtgagtcgcc | 7.10289 |
| **TBS-4** | **-185~-196** | **cctgagtcactc** | **11.047782** |
| **TBS-5** | **+185~+195** | **gagtgactcag** | **15.223728** |
| TBS-6 | +218~+227 | cctgacacac | 6.8806086 |
| TBS-7 | +344~+356 | tggcgactcacac | 7.7727857 |
| TBS-8 | +417~+428 | gatgacagcatg | 8.2902355 |

**Supplementary Table 9.** Clinicopathological characteristics and genetic background of patients in the LUADOs model

| **ID** | **Sex** | **Year** | **Smoking history** | **Pathological type** | **T** | **N** | **M** | **Ki-67** | **Specimen source** | **Mutation background** | **Sensitivity of the third generation EGFR-TKIs** | **Treatment status** |
| --- | --- | --- | --- | --- | --- | --- | --- | --- | --- | --- | --- | --- |
| LUADO-001 | Male | 83 | None | Adenocarcinoma | T2 | N2 | M1 | 40% | Pleural effusion | EGFR19del | OS | Osimertinib |
| LUADO-002 | Female | 47 | None | Microinvasive adenocarcinoma | T4 | N2 | M1 | 5% | Pleural effusion | EGFR19del | OS | Ametinib |
| LUADO-003 | Female | 54 | None | Invasive adenocarcinoma | T4 | N2 | M1 | 2% | Pleural effusion | EGFR19del | OS | Ametinib |
| LUADO-004 | Female | 73 | None | Invasive adenocarcinoma | T4 | N3 | M1 | 40% | Pleural effusion | EGFR T790M | OR | Bevacizumab + Osimertinib |
| LUADO-005 | Male | 76 | None | Invasive adenocarcinoma | T1 | N2 | M1 | 35% | Pleural effusion | EGFR 21 L858R | OR | Cisplatin + Osimertinib |
| LUADO-006 | Female | 64 | None | Invasive adenocarcinoma | T2 | N3 | M1 | 5% | Puncture | EGFR T790M | OS | Osimertinib |
| LUADO-007 | Female | 84 | None | Adenocarcinoma | T1 | N2 | M1 | 25% | Pleural effusion | EGFR19del | OR | Bevacizumab + cisplatin + Ametinib |
| LUADO-008 | Male | 62 | None | Adenocarcinoma | T4 | N3 | M1 | 2% | Pleural effusion | EGFR19del | OR | Bevacizumab + cisplatin + Ametinib |
| LUADO-009 | Female | 52 | None | Invasive adenocarcinoma | T2 | N3 | M1 | 40% | Pleural effusion | EGFR 21 L858R | OS | Furmonertinib |
| LUADO-010 | Female | 59 | None | Adenocarcinoma | T4 | N2 | M1 | 60% | Puncture | EGFR 21 L858R | OS | Furmonertinib |
| LUADO-011 | Female | 58 | None | Adenocarcinoma | T2 | N2 | M1 | 60% | Pleural effusion | EGFR19del | OS | Furmonertinib |
| LUADO-012 | Male | 60 | None | Adenocarcinoma | T2 | N1 | M1 | — | Puncture | EGFR 21 L858R | OS | Ametinib |

LUADOs:lung adenocarcinoma organoids, OR:osimertinib resistance, OS:osimertinib Sensitive

**Supplementary Table 10.** Clinicopathological characteristics and genetic background of patients in the PDX model

| **ID** | **Sex** | **Year** | **Pathological type** | **T** | **N** | **M** | **Ki-67** | **Specimen source** | **Mutation background** | **Sensitivity of the third generation EGFR-TKIs** | **Treatment status** |
| --- | --- | --- | --- | --- | --- | --- | --- | --- | --- | --- | --- |
| PDX-01 | Female | 78 | Adenocarcinoma | T2 | N3 | M1 | 40% | Pleural effusion | EGFR19del | OS | Osimertinib |
| PDX-02 | Female | 44 | Adenocarcinoma | T1 | N2 | M1 | 65% | Pleural effusion | EGFR19del | OR | Bevacizumab + Ametinib |
| PDX-03 | Male | 61 | Adenocarcinoma | T4 | N3 | M1 | 40% | Puncture | EGFR19del | OS | Osimertinib |
| PDX-04 | Female | 59 | Invasive adenocarcinoma | T2 | N3 | M1 | 40% | Pleural effusion | EGFR 21 L858R | OS | Furmonertinib |
| PDX-05 | Male | 66 | Invasive adenocarcinoma | T3 | N2 | M1 | 10% | Pleural effusion | EGFR 21 L858R | OR | Bevacizumab + Osimertinib |
| PDX-06 | Male | 53 | Invasive adenocarcinoma | T3 | N2 | M1 | 15% | Puncture | EGFR T790M | OS | Osimertinib |
| PDX-07 | Male | 52 | Adenocarcinoma | T4 | N2 | M1 | 50% | Pleural effusion | EGFR19del | OS | Ametinib |
| PDX-08 | Female | 67 | Invasive adenocarcinoma | T4 | N3 | M1 | 20% | Pleural effusion | EGFR T790M | OR | Bevacizumab+ Ametinib |
| PDX-09 | Male | 51 | Invasive adenocarcinoma | T4 | N3 | M1 | 40% | Pleural effusion | EGFR 21 L858R | OR | Bevacizumab + Osimertinib |

PDX: Patient-Derived Tumor Xenograft, OR: osimertinib resistance, OS: osimertinib Sensitive
