# Supplementary material for: Non-enzymatic function of QSOX2 directly regulates the JUNB-ITGB4 axis and enhanced resistance to osimertinib in EGFR-mutation lung adenocarcinoma
Source: Cell Death Discov. 2026 Apr 1;12:215. doi: 10.1038/s41420-026-02969-4 (PMC13168330; doi:10.1038/s41420-026-02969-4)
Supplement: Supplementary file 2 — Raw data [file 41420_2026_2969_MOESM2_ESM.pdf]

figure1F-WB

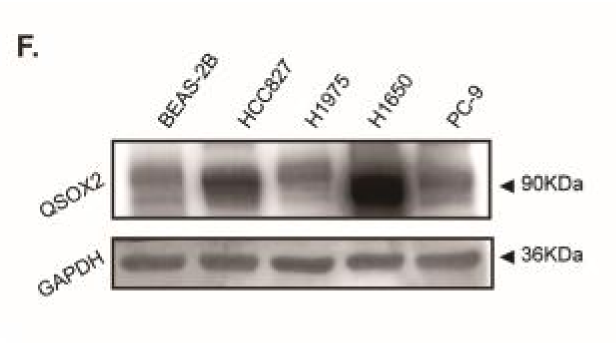

Marker

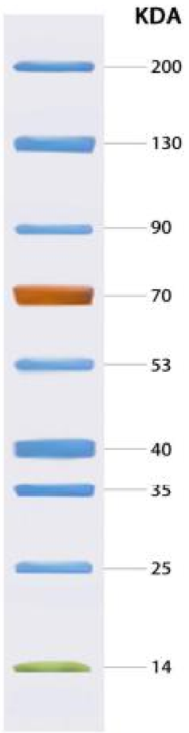

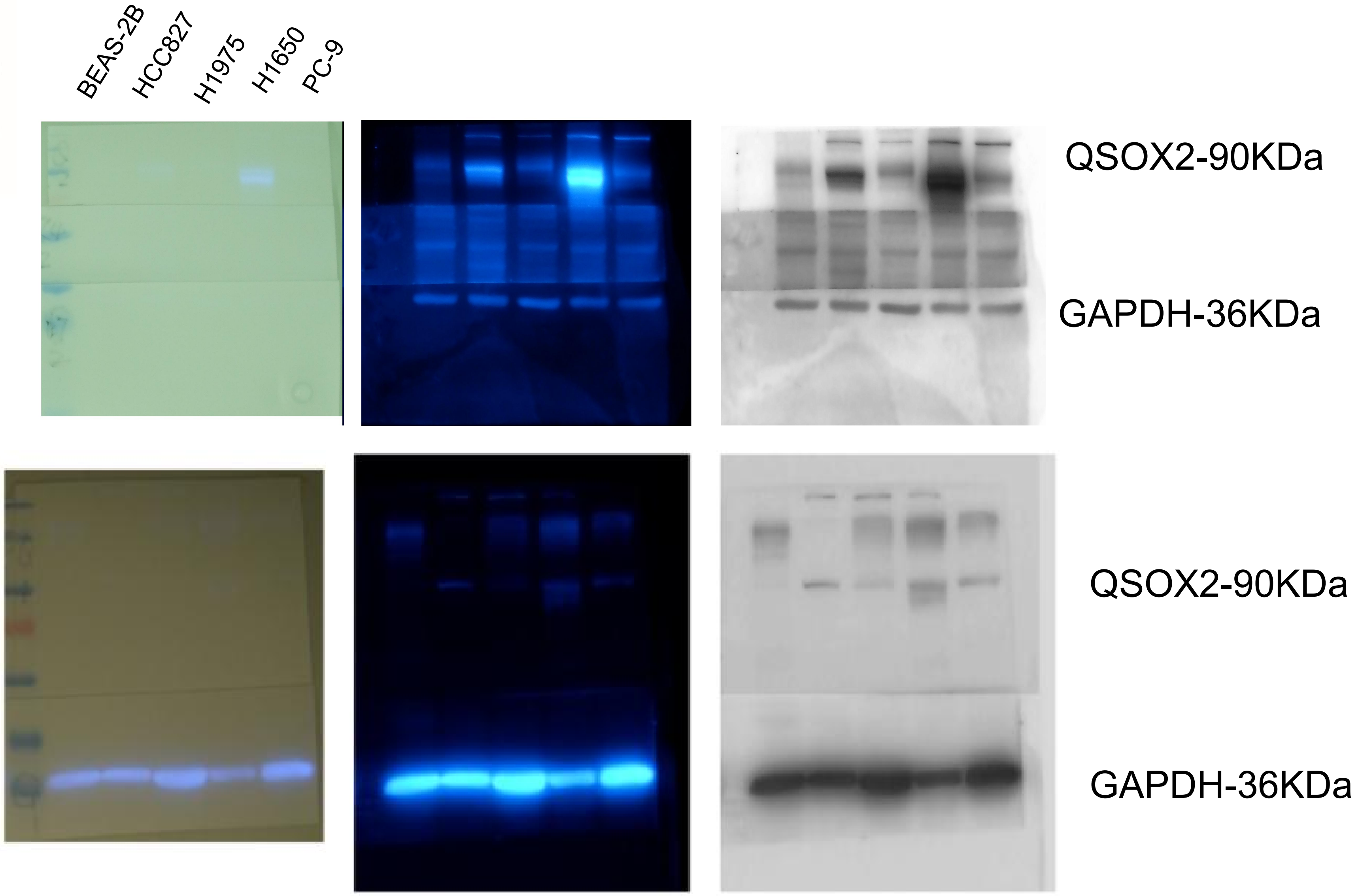

figure1F-WB

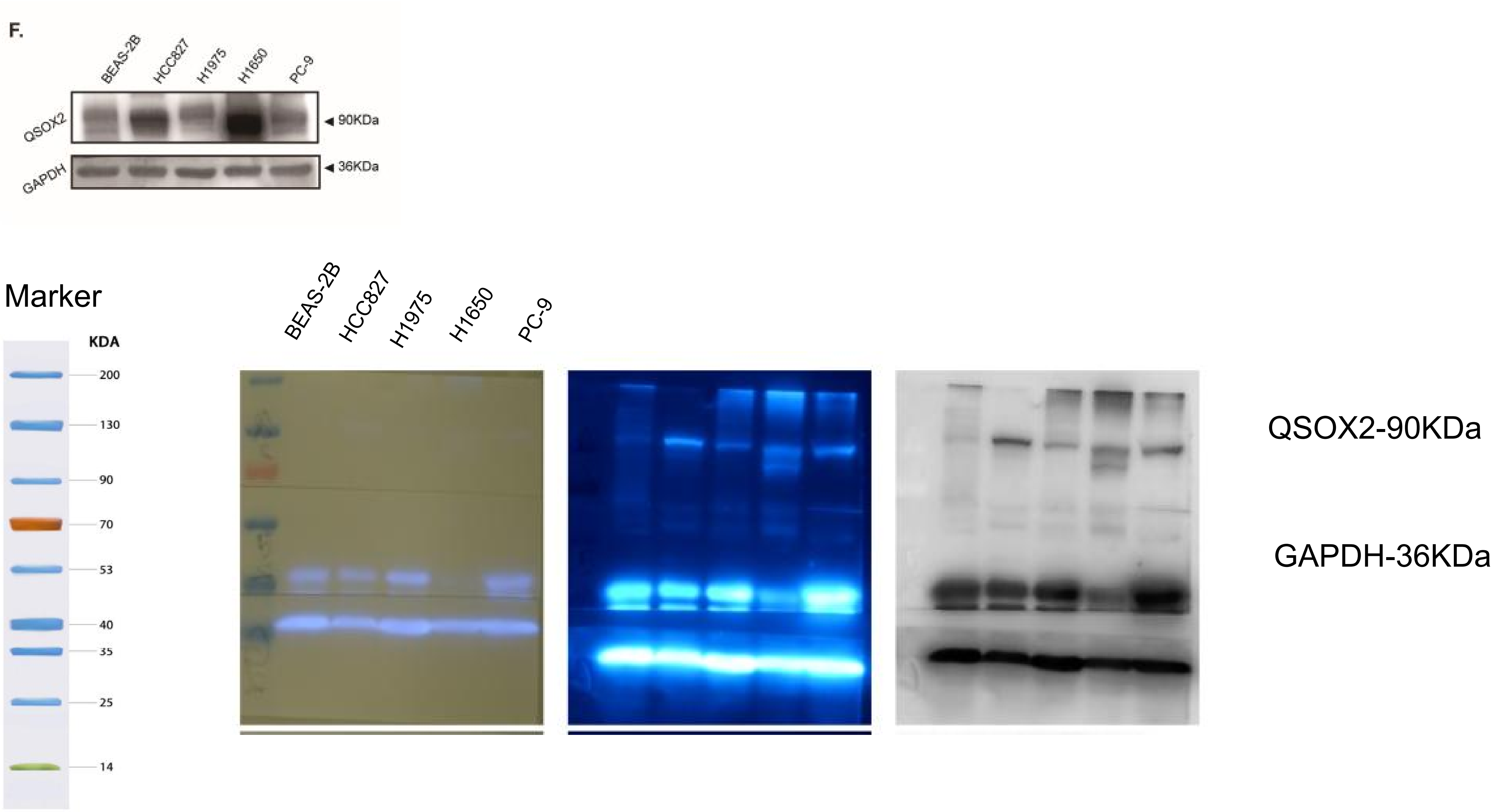
F.
BEAS-2B
HCC827
H1975
H1650
PC-9
QSOX2
GAPDH
◀ 90KDa
◀ 36KDa
Marker
KDA
200
130
90
70
53
40
35
25
14
BEAS-2B
HCC827
H1975
H1650
PC-9
QSOX2-90KDa
GAPDH-36KDa

figure3E-WB

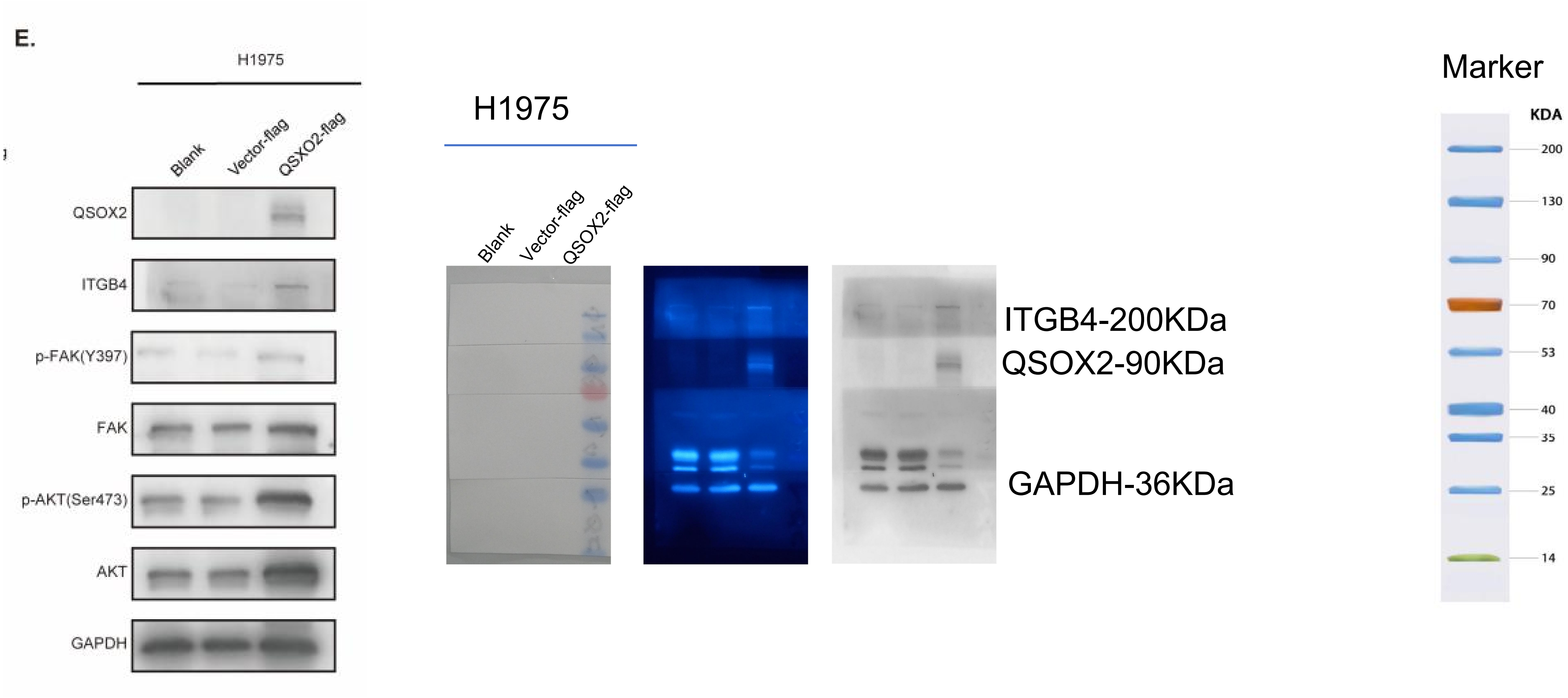
E.
H1975
Blank
Vector-flag
QSXO2-flag
QSOX2
ITGB4
p-FAK(Y397)
FAK
p-AKT(Ser473)
AKT
GAPDH
H1975
Blank
Vector-flag
QSOX2-flag
ITGB4-200KDa
QSOX2-90KDa
GAPDH-36KDa
Marker
KDA
200
130
90
70
53
40
35
25
14

figure3E-WB

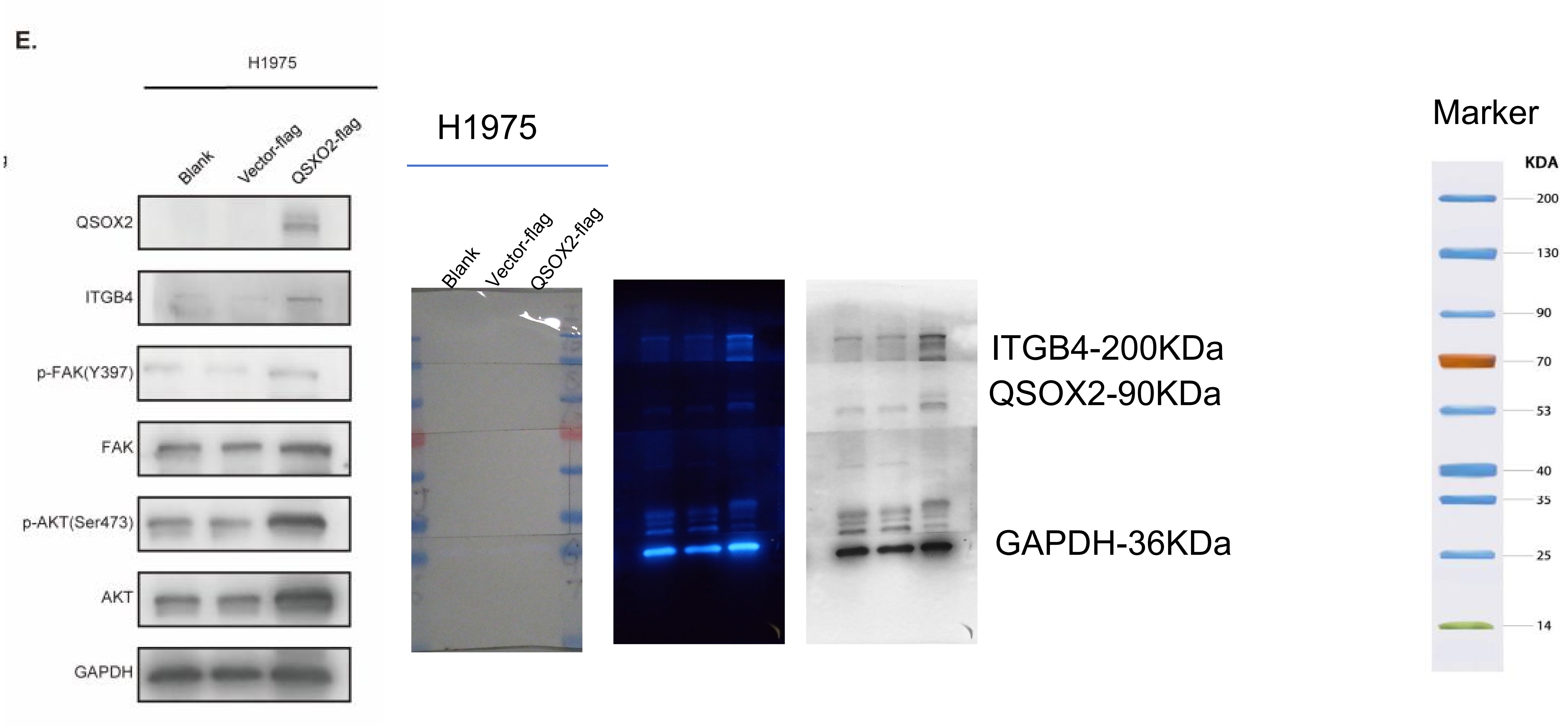

E.
H1975
Blank
Vector-flag
QSXO2-flag
QSOX2
ITGB4
p-FAK(Y397)
FAK
p-AKT(Ser473)
AKT
GAPDH
H1975
Blank
Vector-flag
QSOX2-flag
ITGB4-200KDa
QSOX2-90KDa
GAPDH-36KDa
Marker
KDA
200
130
90
70
53
40
35
25
14

figure3E-WB

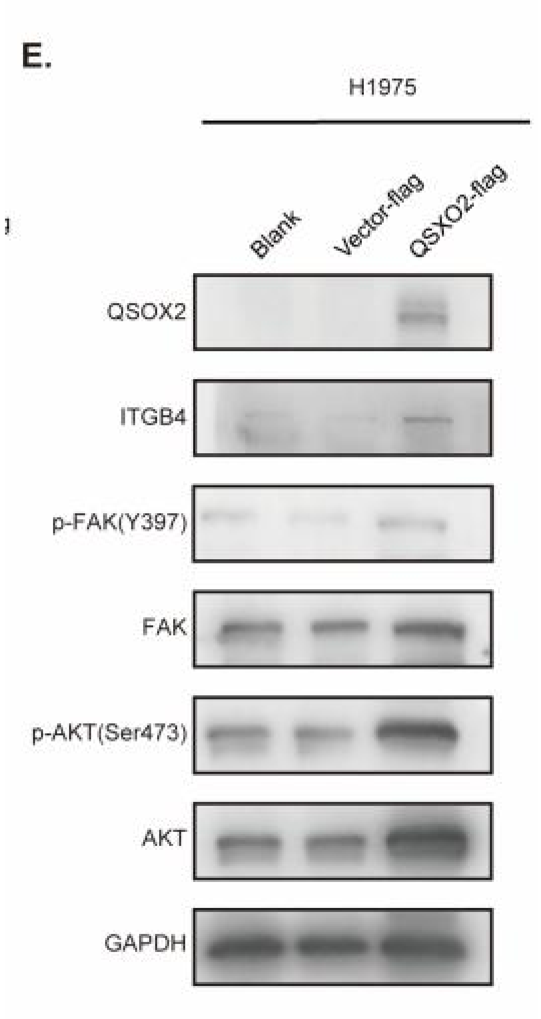
E.
H1975
Blank
Vector-flag
QSXO2-flag
QSOX2
ITGB4
p-FAK(Y397)
FAK
p-AKT(Ser473)
AKT
GAPDH

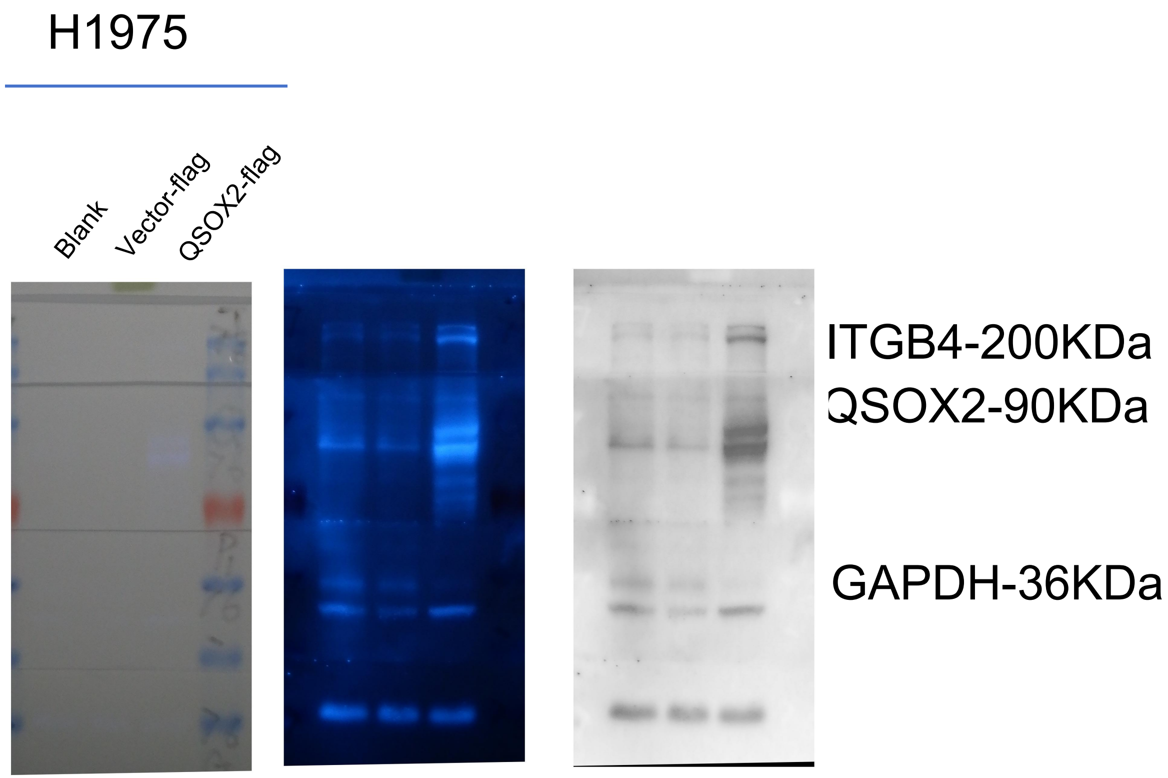
H1975
Blank
Vector-flag
QSOX2-flag
ITGB4-200KDa
QSOX2-90KDa
GAPDH-36KDa

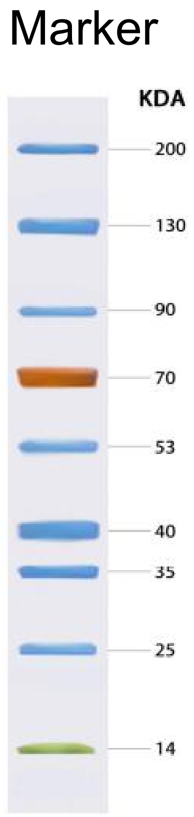
Marker
KDA
200
130
90
70
53
40
35
25
14

figure3E-WB

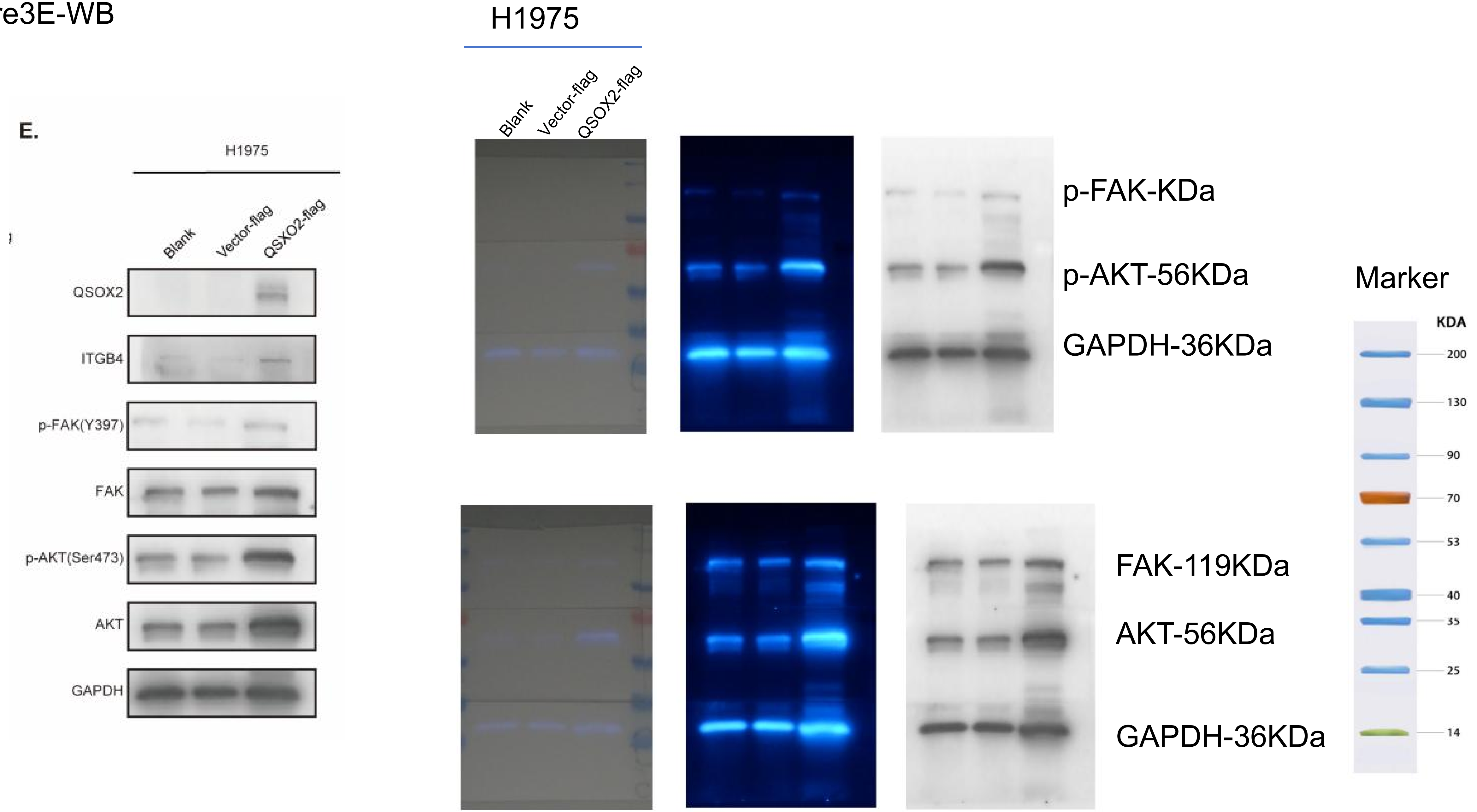
E.
H1975
Blank
Vector-flag
QSXO2-flag
QSOX2
ITGB4
p-FAK(Y397)
FAK
p-AKT(Ser473)
AKT
GAPDH
H1975
Blank
Vector-flag
QSOX2-flag
p-FAK-KDa
p-AKT-56KDa
GAPDH-36KDa
FAK-119KDa
AKT-56KDa
GAPDH-36KDa
Marker
KDA
200
130
90
70
53
40
35
25
14

figure3E-WB

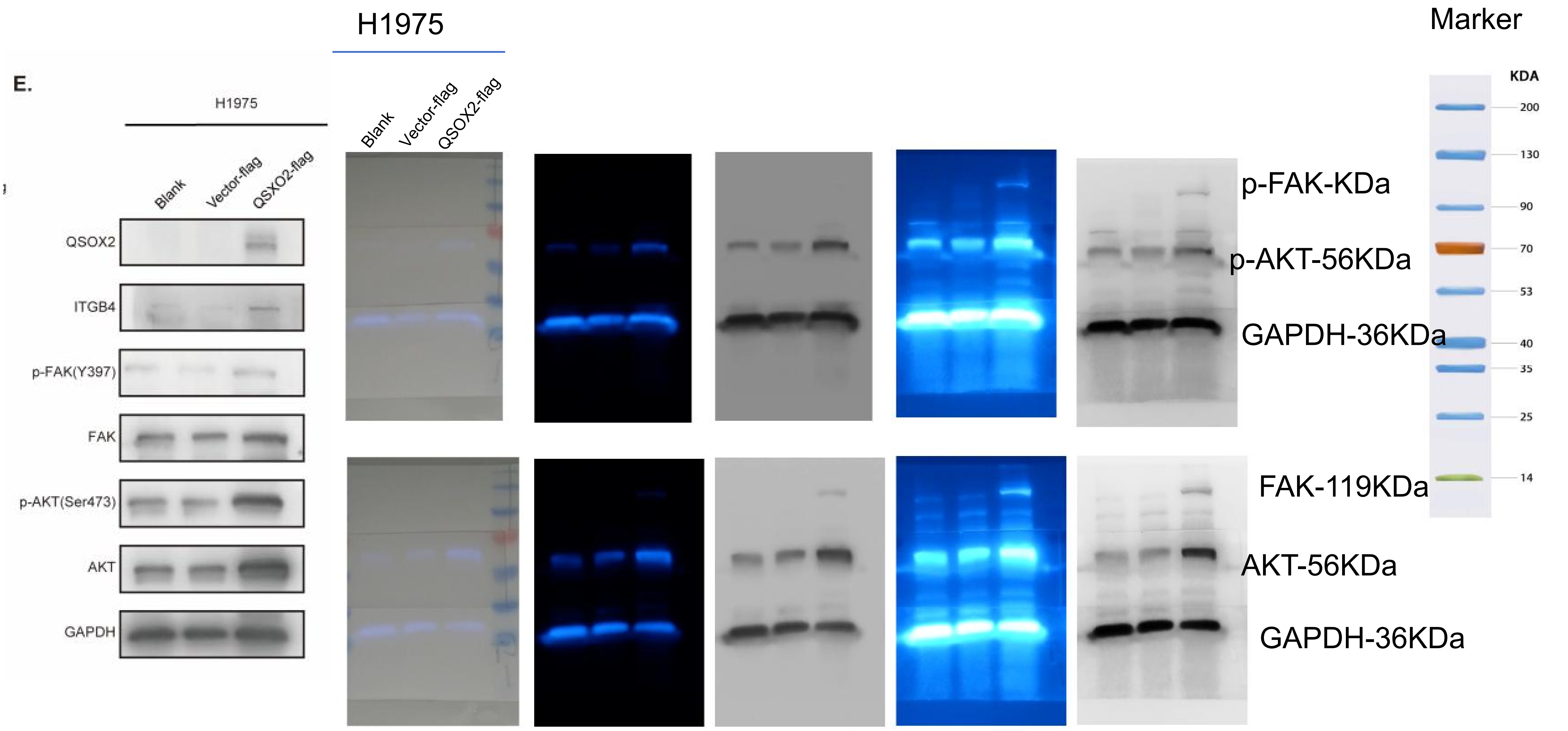
H1975
Marker
E.
H1975
Blank
Vector-flag
QSXO2-flag
QSOX2
ITGB4
p-FAK(Y397)
FAK
p-AKT(Ser473)
AKT
GAPDH
Blank
Vector-flag
QSOX2-flag
p-FAK-KDa
p-AKT-56KDa
GAPDH-36KDa
FAK-119KDa
AKT-56KDa
GAPDH-36KDa
KDA
200
130
90
70
53
40
35
25
14

figure3E-WB

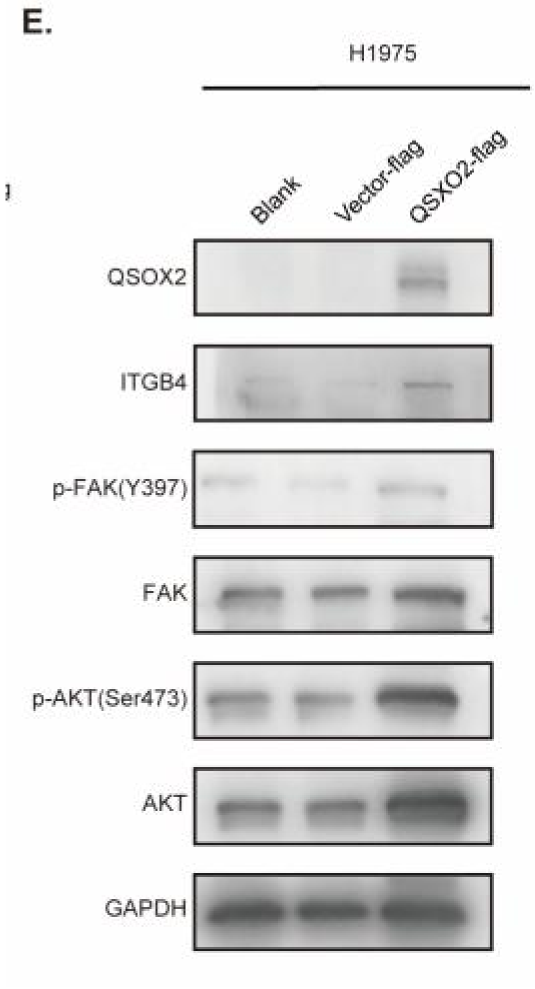
E.
H1975
Blank
Vector-flag
QSXO2-flag
QSOX2
ITGB4
p-FAK(Y397)
FAK
p-AKT(Ser473)
AKT
GAPDH

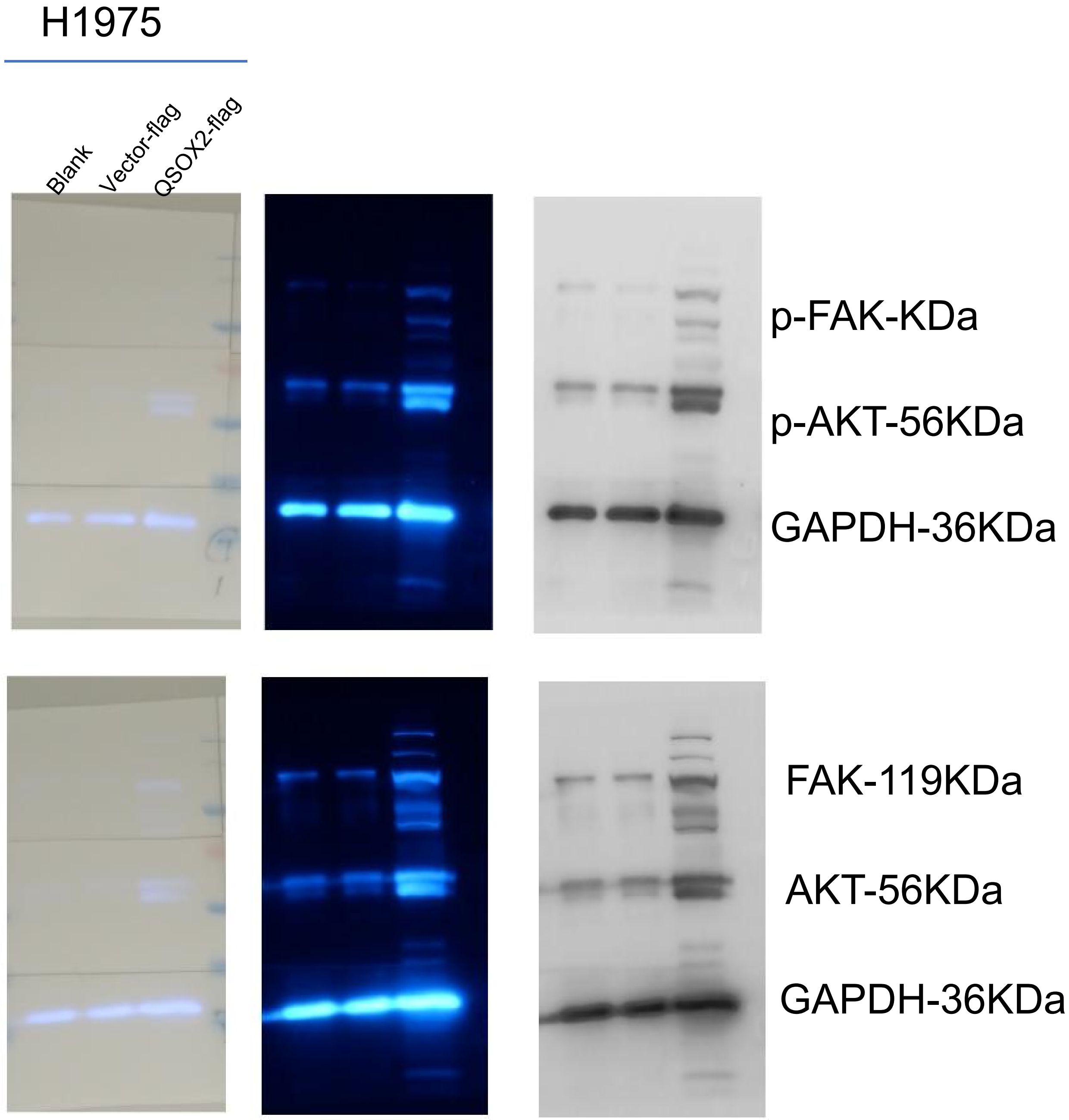
H1975
Blank
Vector-flag
QSOX2-flag
p-FAK-KDa
p-AKT-56KDa
GAPDH-36KDa
FAK-119KDa
AKT-56KDa
GAPDH-36KDa

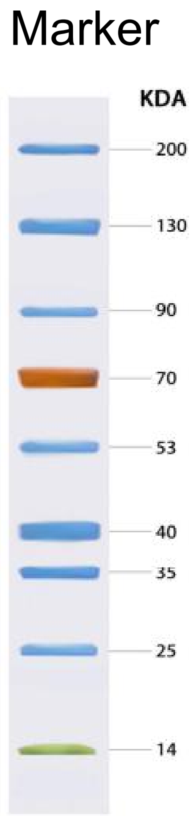
Marker
KDA
200
130
90
70
53
40
35
25
14

figure3E-WB

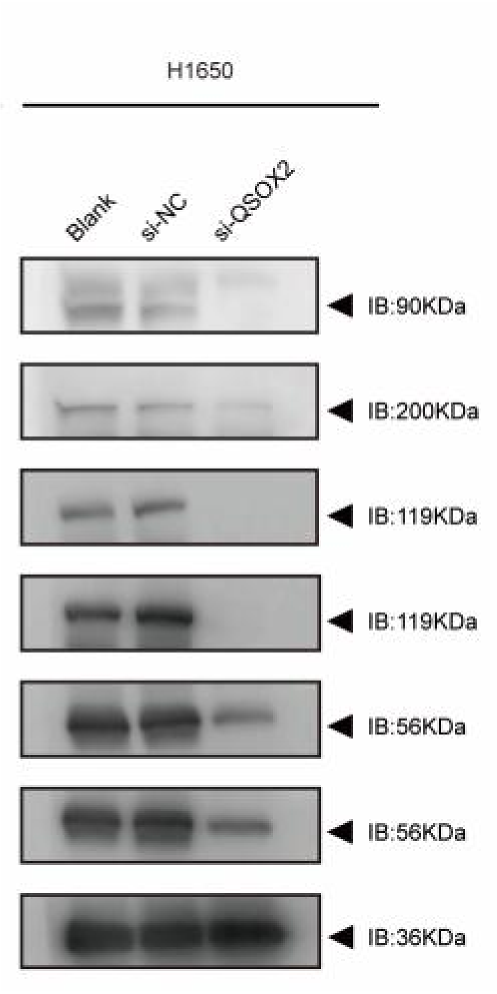
H1650
Blank
si-NC
si-QSOX2
IB:90KDa
IB:200KDa
IB:119KDa
IB:119KDa
IB:56KDa
IB:56KDa
IB:36KDa

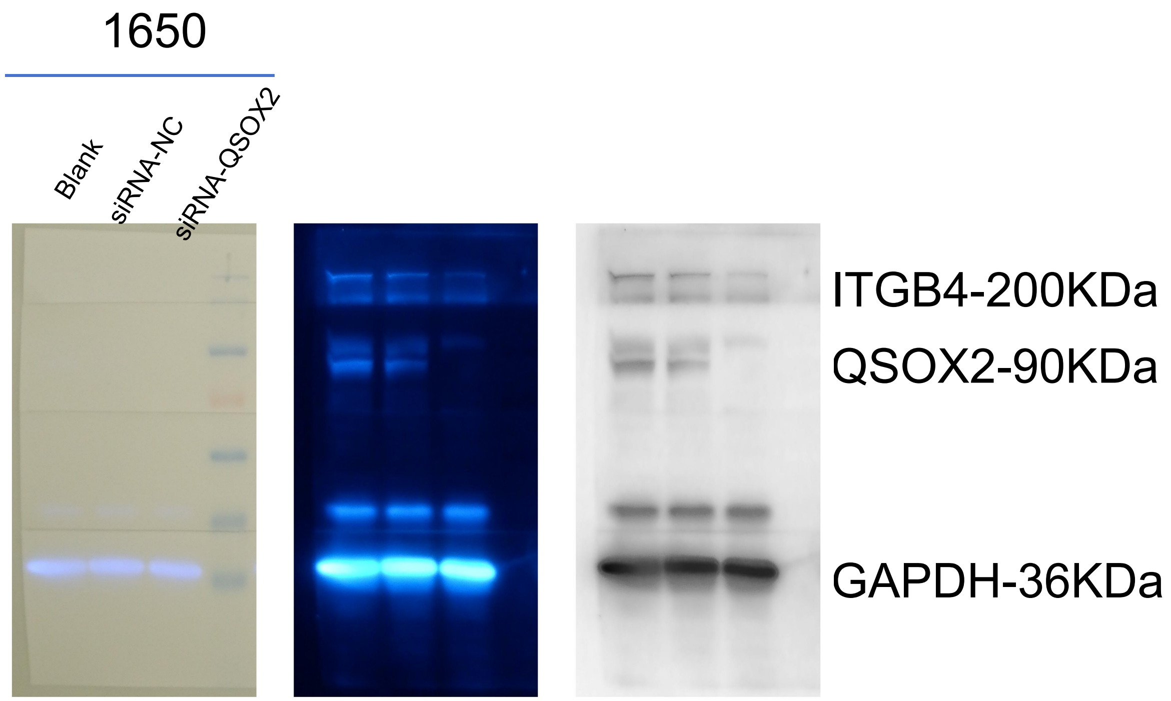
1650
Blank
siRNA-NC
siRNA-QSOX2
ITGB4-200KDa
QSOX2-90KDa
GAPDH-36KDa

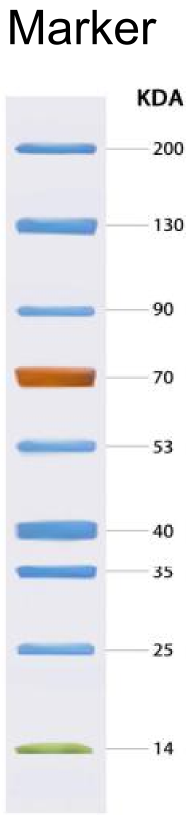
Marker
KDA
200
130
90
70
53
40
35
25
14

figure3E-WB

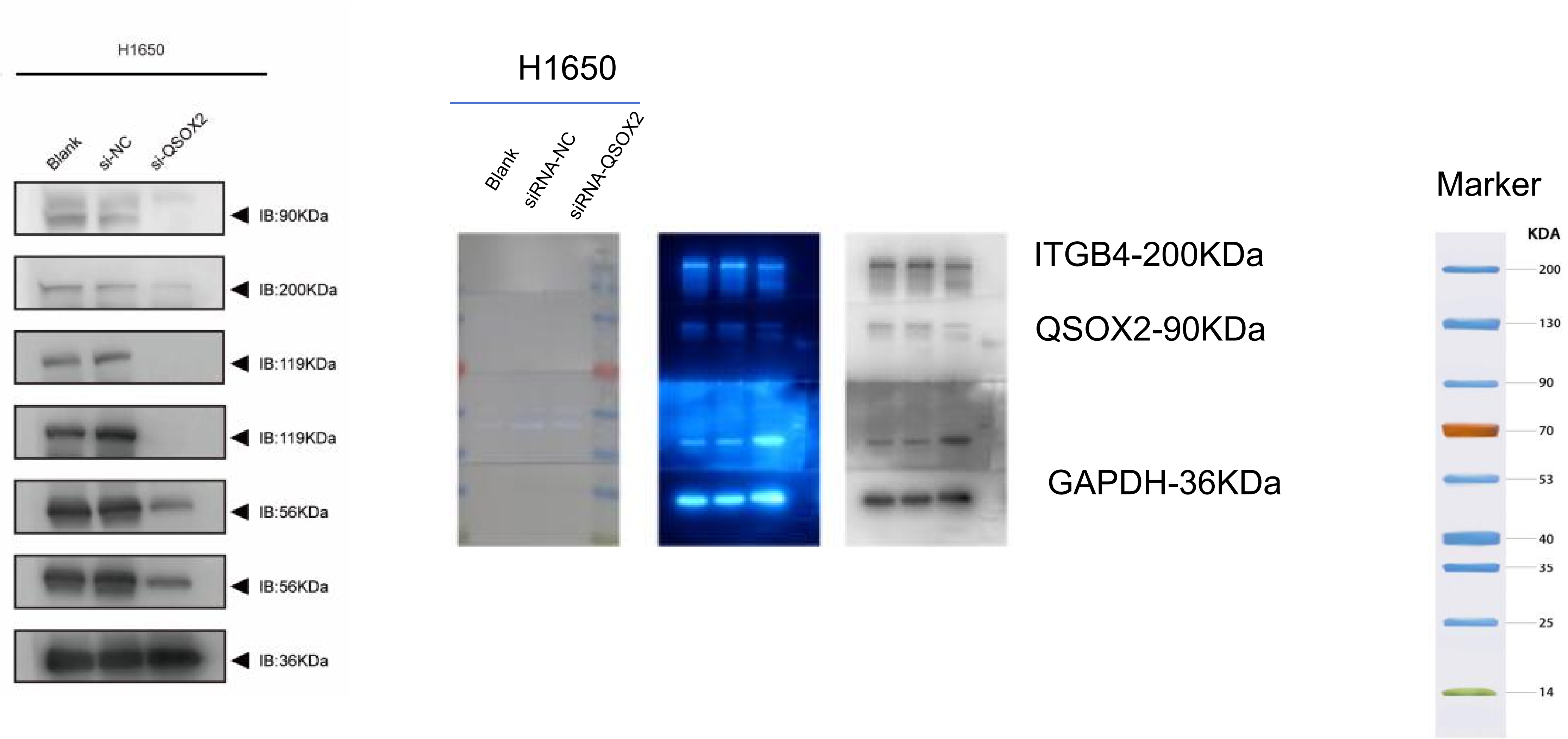
H1650
Blank
si-NC
si-QSOX2
IB:90KDa
IB:200KDa
IB:119KDa
IB:119KDa
IB:56KDa
IB:56KDa
IB:36KDa
H1650
Blank
siRNA-NC
siRNA-QSOX2
ITGB4-200KDa
QSOX2-90KDa
GAPDH-36KDa
Marker
KDA
200
130
90
70
53
40
35
25
14

figure3E-WB

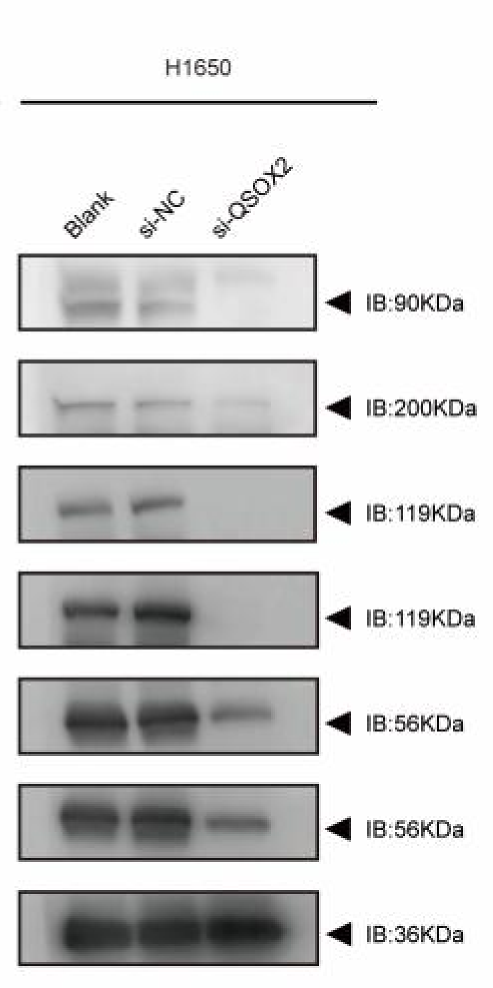
H1650
Blank
si-NC
si-QSOX2
IB:90KDa
IB:200KDa
IB:119KDa
IB:119KDa
IB:56KDa
IB:56KDa
IB:36KDa

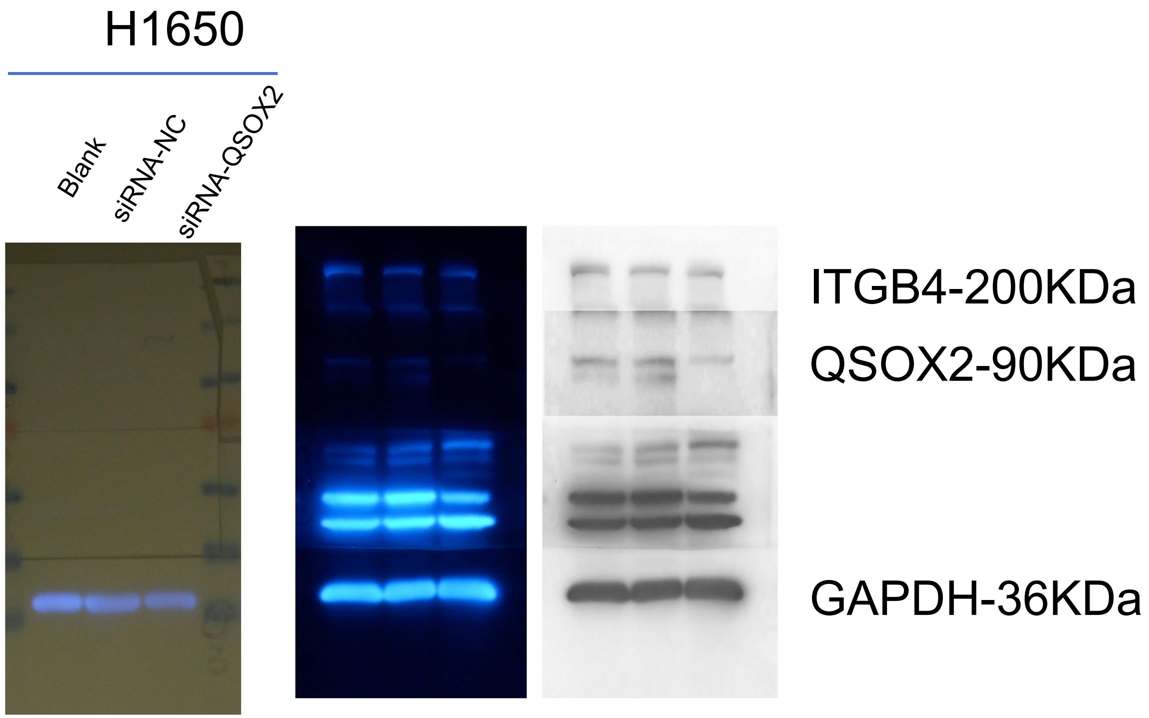
H1650
Blank
siRNA-NC
siRNA-QSOX2
ITGB4-200KDa
QSOX2-90KDa
GAPDH-36KDa

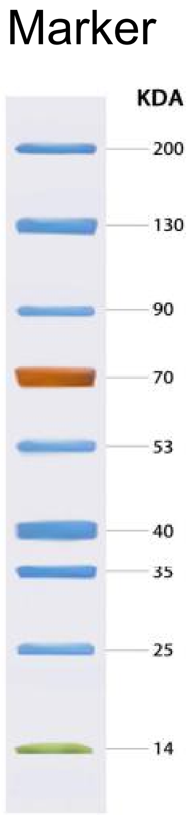
Marker
KDA
200
130
90
70
53
40
35
25
14

figure3E-WB

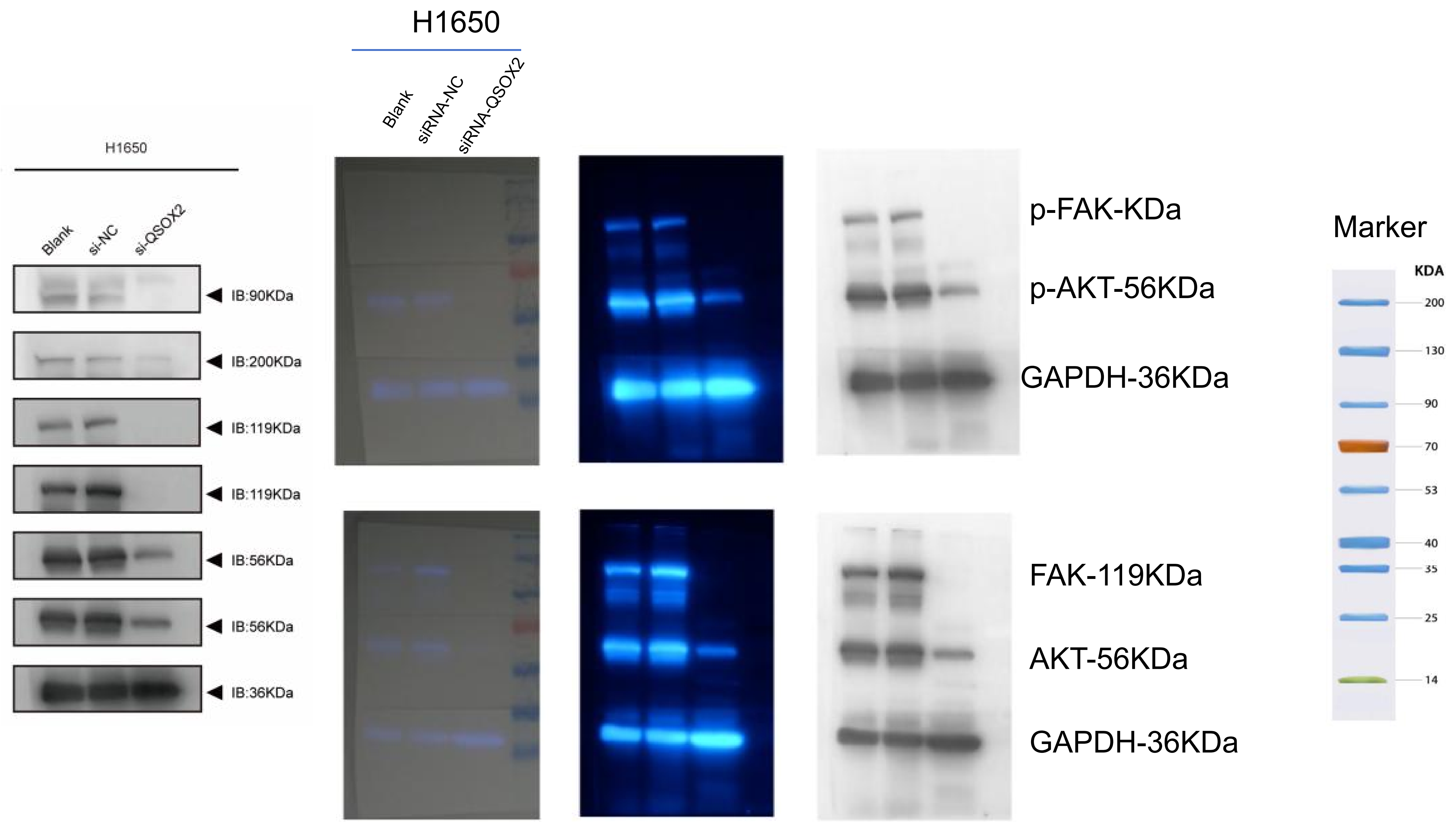
H1650
Blank
siRNA-NC
siRNA-QSOX2
H1650
Blank
si-NC
si-QSOX2
IB:90KDa
IB:200KDa
IB:119KDa
IB:119KDa
IB:56KDa
IB:56KDa
IB:36KDa
p-FAK-KDa
p-AKT-56KDa
GAPDH-36KDa
FAK-119KDa
AKT-56KDa
GAPDH-36KDa
Marker
KDA
200
130
90
70
53
40
35
25
14

figure3E-WB

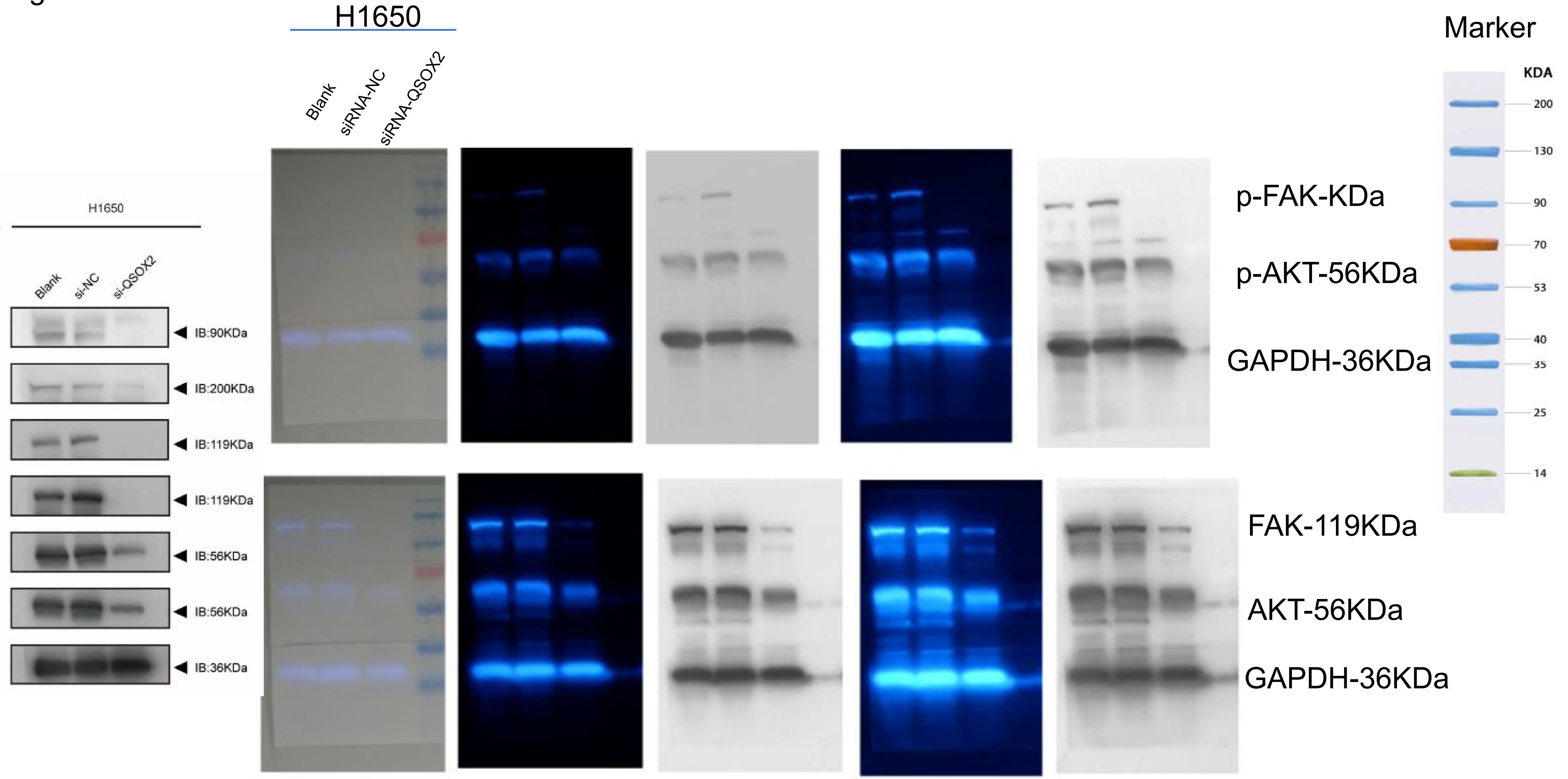

H1650
Blank
siRNA-NC
siRNA-QSOX2
Marker
KDA
200
130
90
70
53
40
35
25
14
p-FAK-KDa
p-AKT-56KDa
GAPDH-36KDa
FAK-119KDa
AKT-56KDa
GAPDH-36KDa
H1650
Blank
si-NC
si-QSOX2
IB:90KDa
IB:200KDa
IB:119KDa
IB:119KDa
IB:56KDa
IB:56KDa
IB:36KDa

figure3E-WB

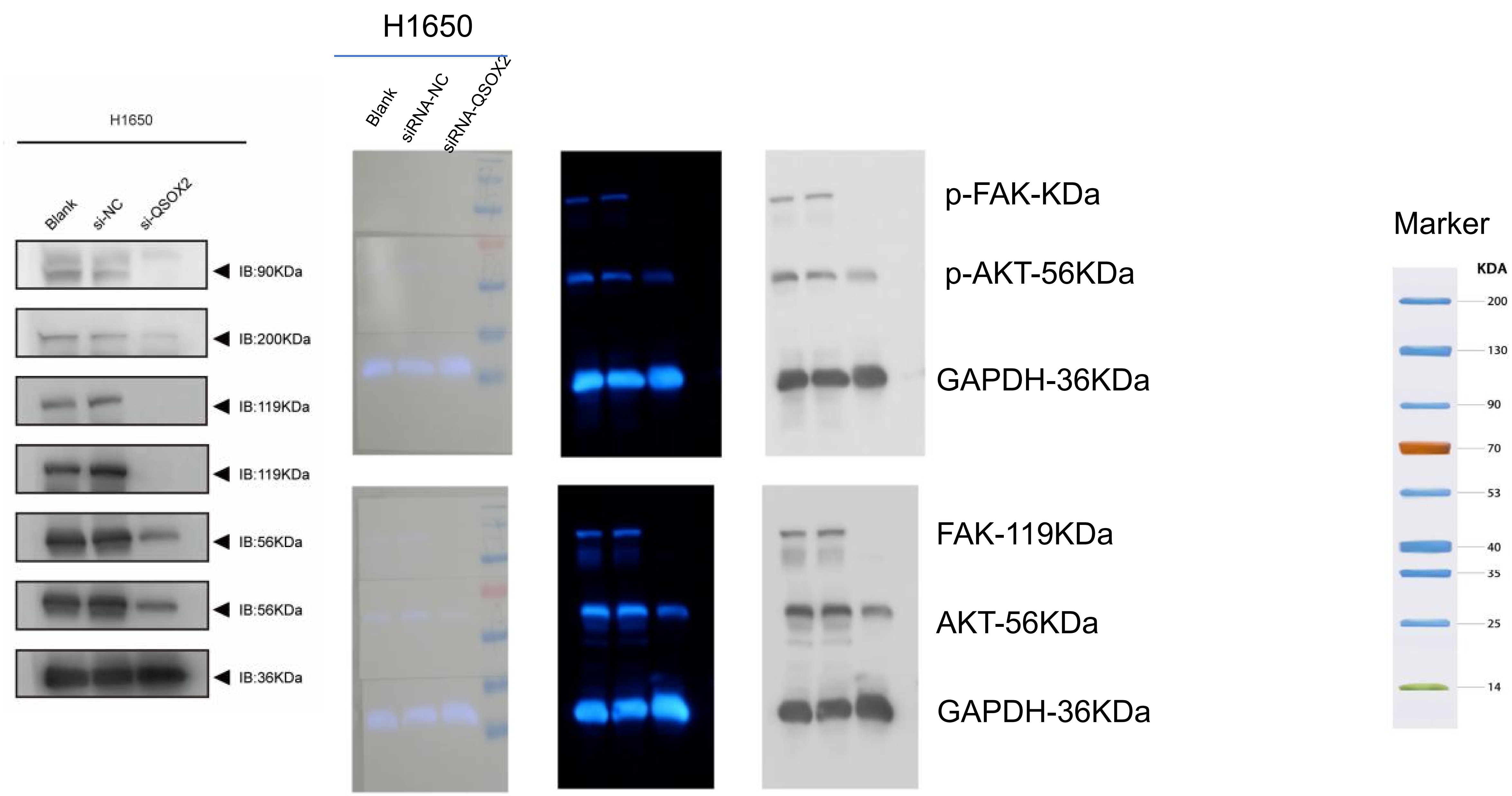
H1650
Blank
si-NC
si-QSOX2
IB:90KDa
IB:200KDa
IB:119KDa
IB:119KDa
IB:56KDa
IB:56KDa
IB:36KDa
H1650
Blank
siRNA-NC
siRNA-QSOX2
p-FAK-KDa
p-AKT-56KDa
GAPDH-36KDa
FAK-119KDa
AKT-56KDa
GAPDH-36KDa
Marker
KDA
200
130
90
70
53
40
35
25
14

figure4B-WB

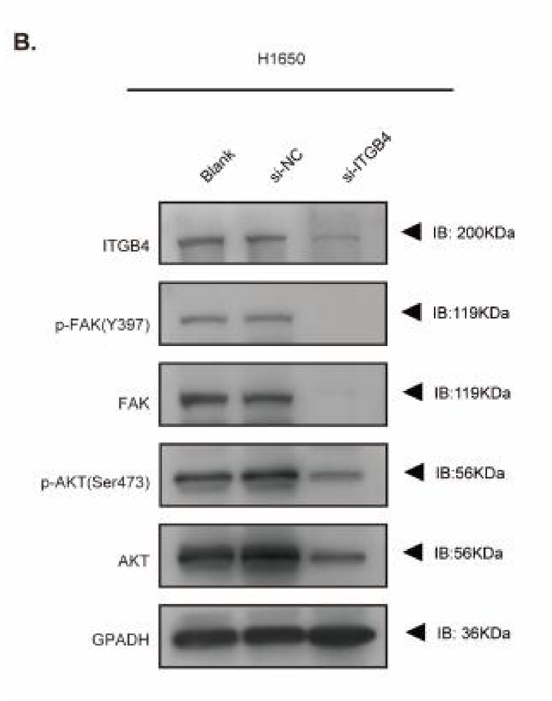
B.
H1650
Blank
si-NC
si-ITGB4
ITGB4
p-FAK(Y397)
FAK
p-AKT(Ser473)
AKT
GPADH
IB: 200KDa
IB:119KDa
IB:119KDa
IB:56KDa
IB:56KDa
IB: 36KDa

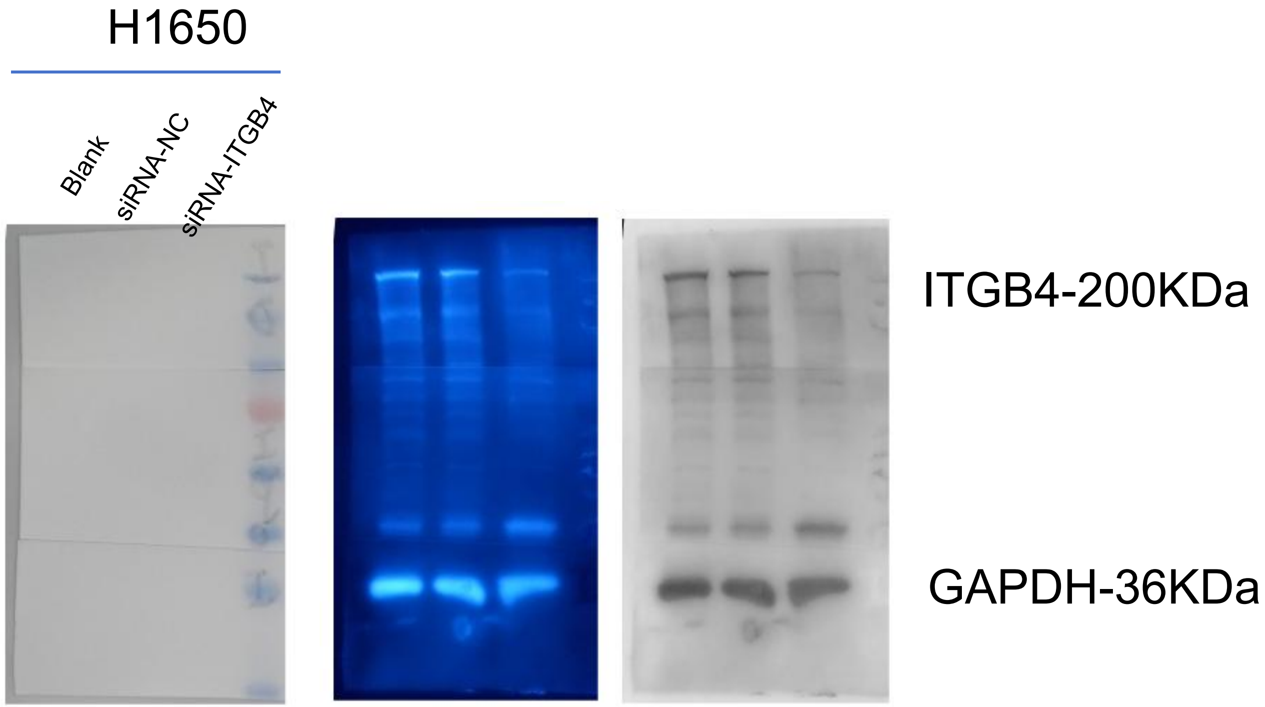
H1650
Blank
siRNA-NC
siRNA-ITGB4
ITGB4-200KDa
GAPDH-36KDa

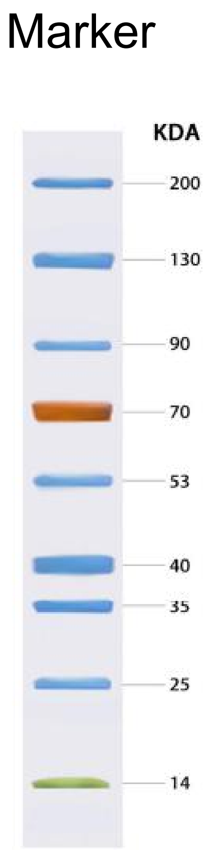
Marker
KDA
200
130
90
70
53
40
35
25
14

figure4B-WB

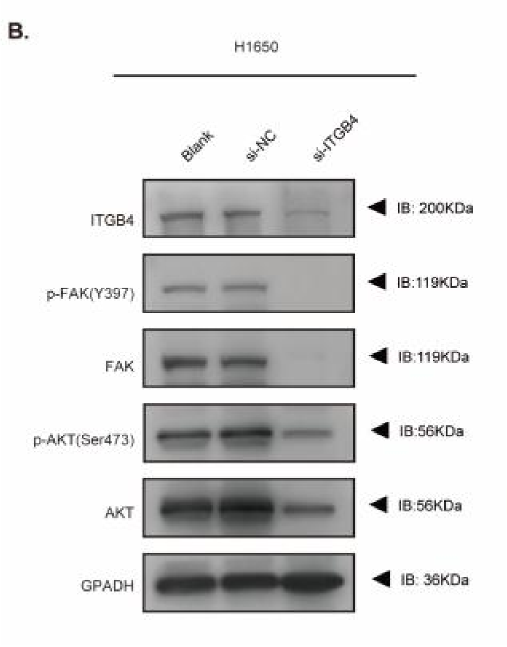

H1650

Blank
siRNA-NC
siRNA-ITGB4

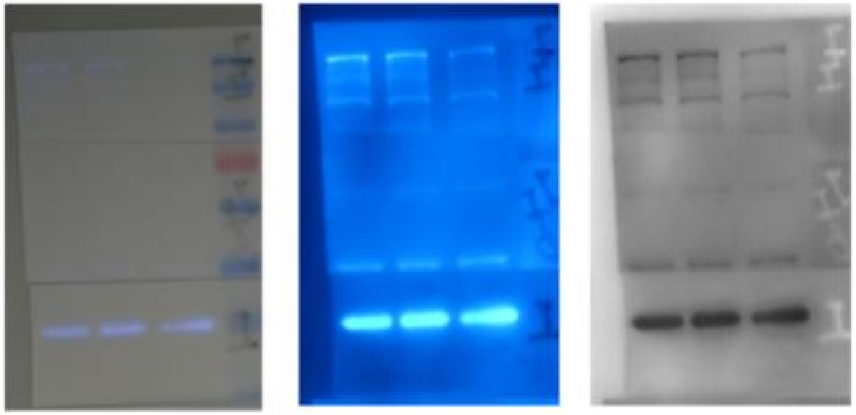

ITGB4-200KDa

GAPDH-36KDa

Marker

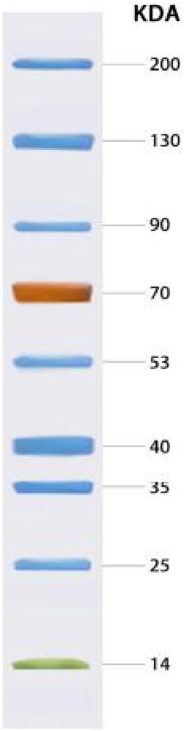

figure4B-WB

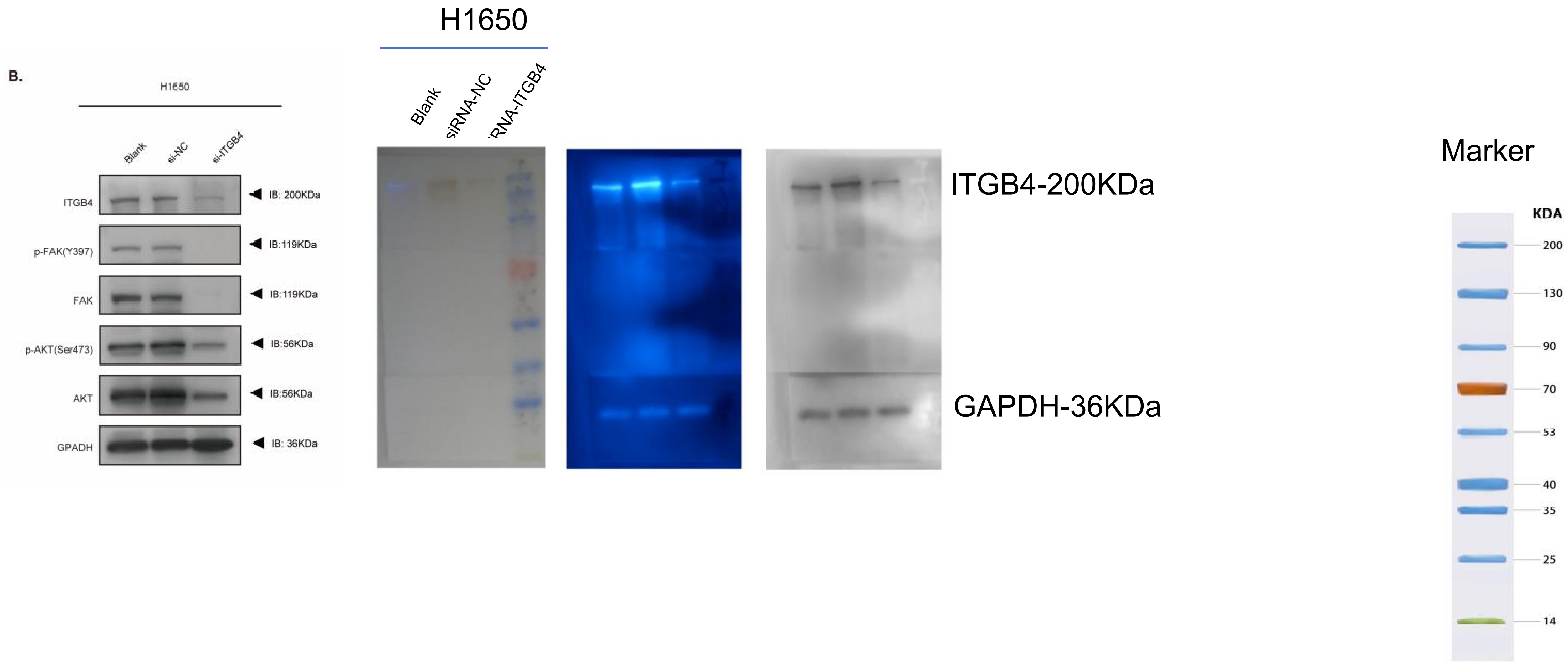
B.
H1650
Blank
si-NC
si-ITGB4
ITGB4
p-FAK(Y397)
FAK
p-AKT(Ser473)
AKT
GPADH
IB: 200KDa
IB:119KDa
IB:119KDa
IB:56KDa
IB:56KDa
IB: 36KDa
H1650
Blank
siRNA-NC
siRNA-ITGB4
ITGB4-200KDa
GAPDH-36KDa
Marker
KDA
200
130
90
70
53
40
35
25
14

figure4B-WB

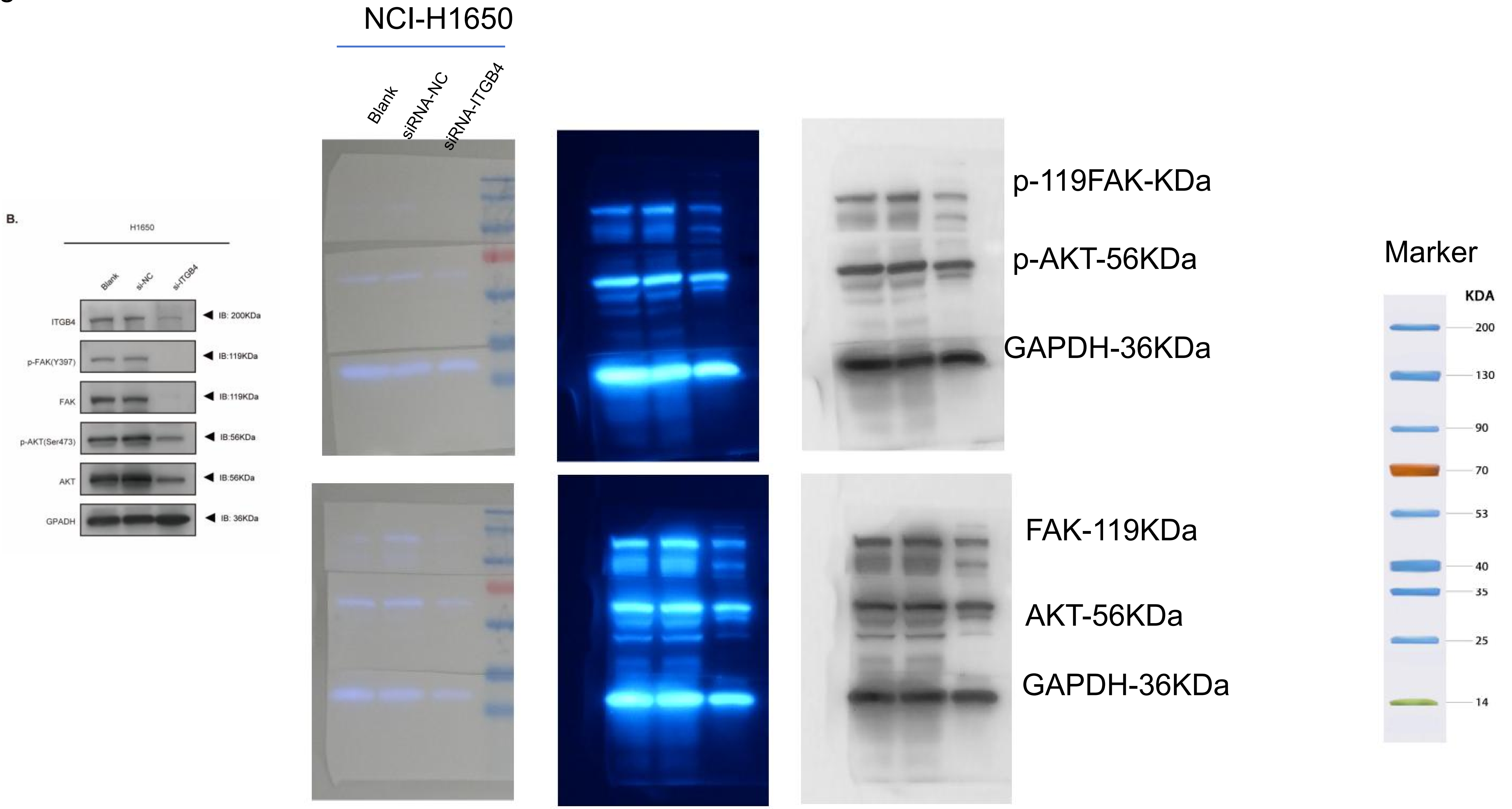

NCI-H1650
Blank
siRNA-NC
siRNA-ITGB4
p-119FAK-KDa
p-AKT-56KDa
GAPDH-36KDa
FAK-119KDa
AKT-56KDa
GAPDH-36KDa
B.
H1650
Blank
si-NC
si-ITGB4
ITGB4
IB: 200KDa
p-FAK(Y397)
IB:119KDa
FAK
IB:119KDa
p-AKT(Ser473)
IB:56KDa
AKT
IB:56KDa
GPADH
IB: 36KDa
Marker
KDA
200
130
90
70
53
40
35
25
14

figure4B-WB

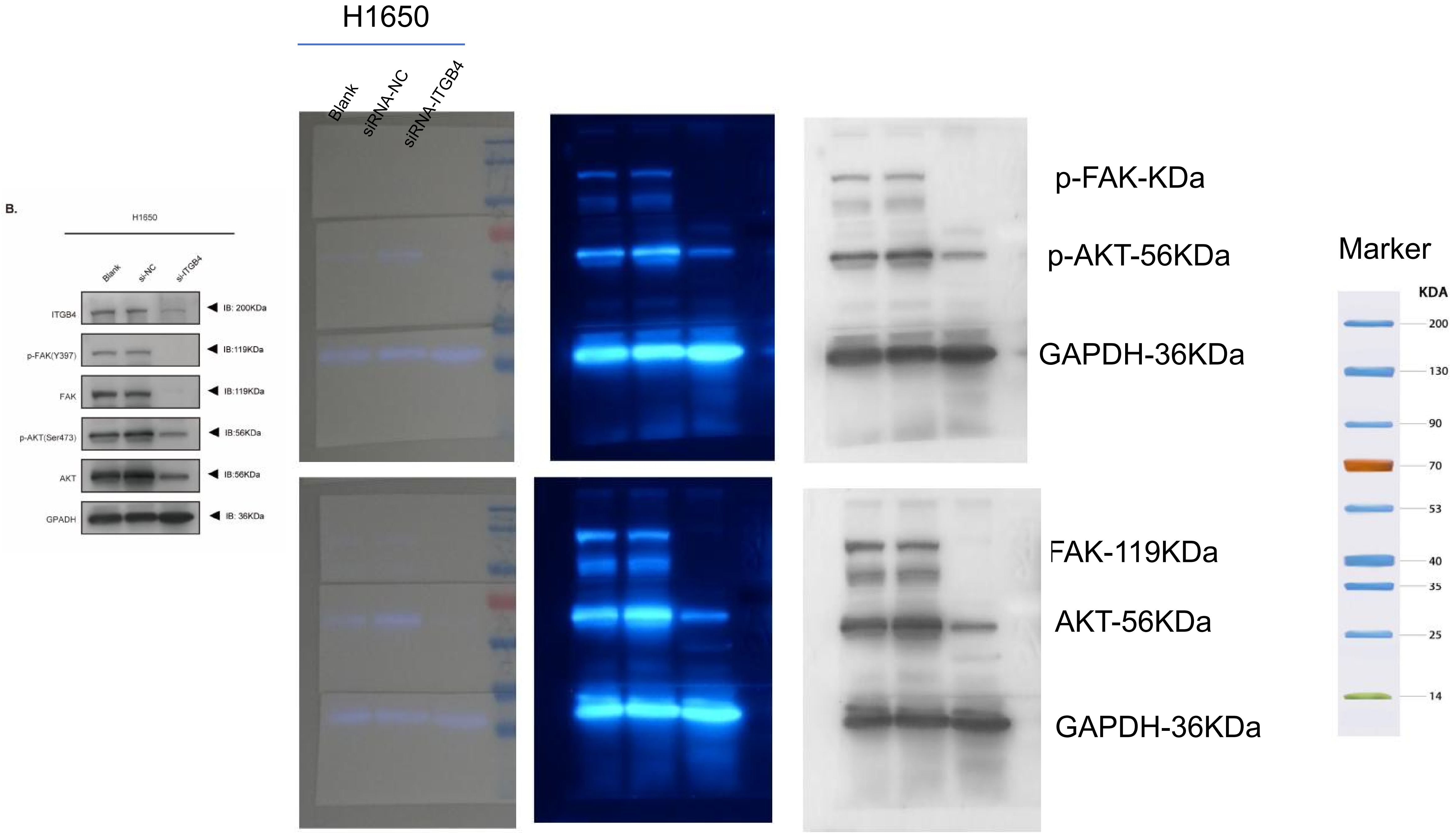
B.
H1650
Blank
si-NC
si-ITGB4
ITGB4
IB: 200KDa
p-FAK(Y397)
IB:119KDa
FAK
IB:119KDa
p-AKT(Ser473)
IB:56KDa
AKT
IB:56KDa
GPADH
IB: 36KDa
H1650
Blank
siRNA-NC
siRNA-ITGB4
p-FAK-KDa
p-AKT-56KDa
GAPDH-36KDa
FAK-119KDa
AKT-56KDa
GAPDH-36KDa
Marker
KDA
200
130
90
70
53
40
35
25
14

figure4B-WB

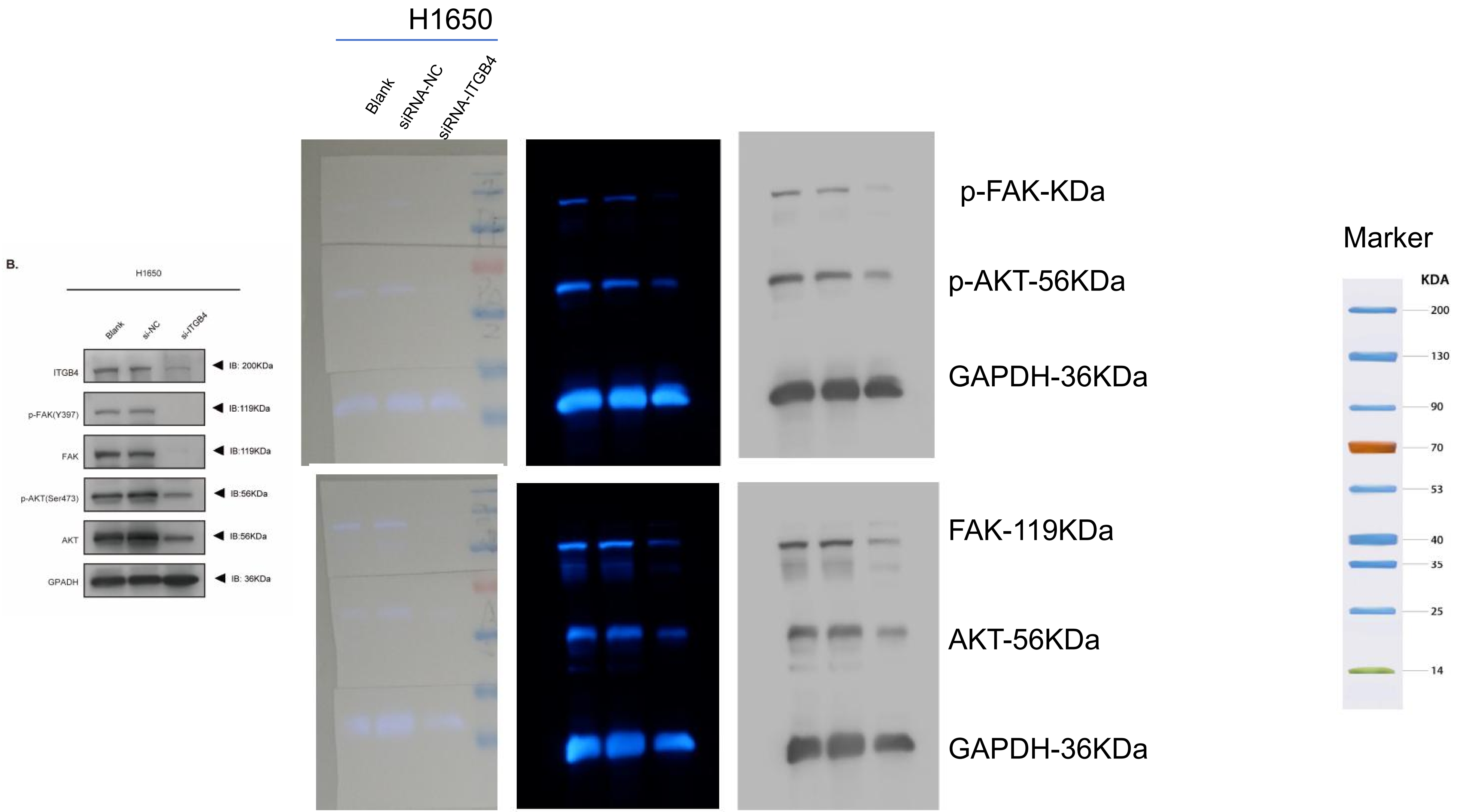
H1650
Blank
siRNA-NC
siRNA-ITGB4
p-FAK-KDa
p-AKT-56KDa
GAPDH-36KDa
FAK-119KDa
AKT-56KDa
GAPDH-36KDa
Marker
KDA
200
130
90
70
53
40
35
25
14
B.
H1650
Blank
si-NC
si-ITGB4
ITGB4
p-FAK(Y397)
FAK
p-AKT(Ser473)
AKT
GPADH
IB: 200KDa
IB:119KDa
IB:119KDa
IB:56KDa
IB:56KDa
IB: 36KDa

figure4C-WB

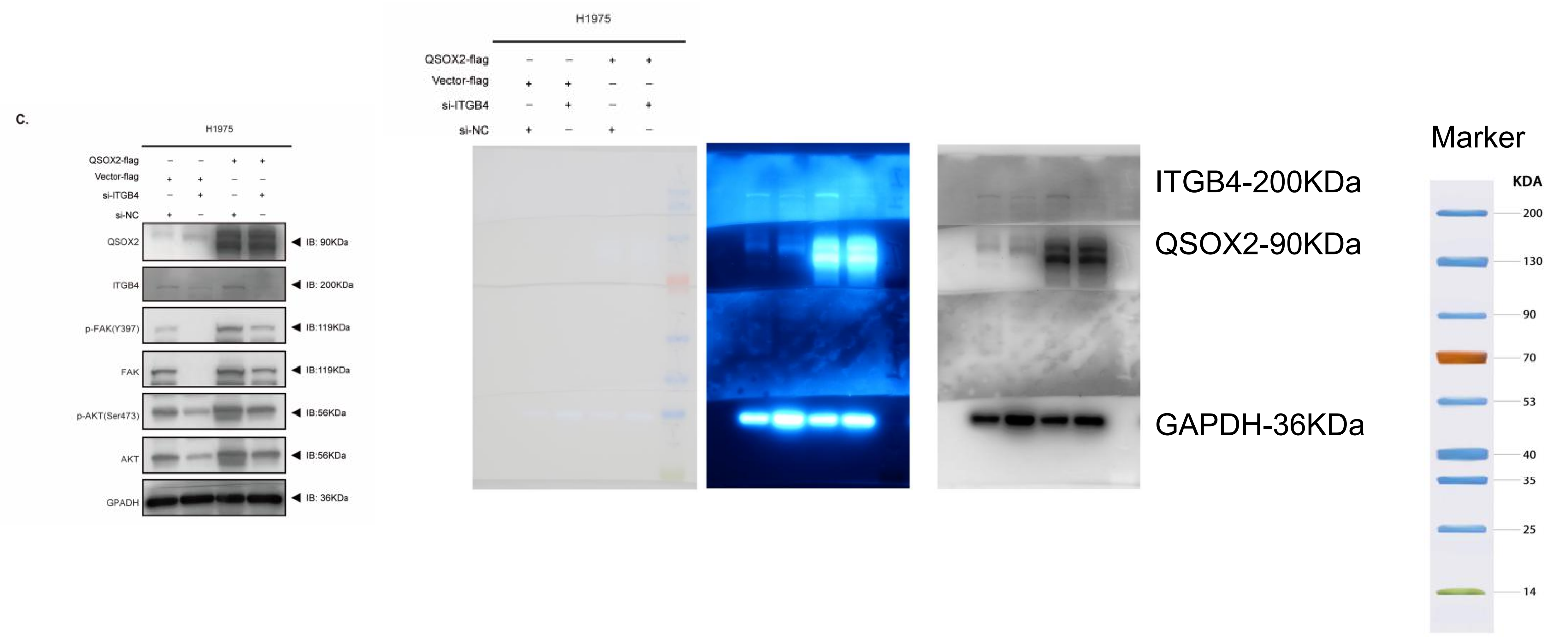
C.
H1975
QSOX2-flag
Vector-flag
si-ITGB4
si-NC
QSOX2
ITGB4
p-FAK(Y397)
FAK
p-AKT(Ser473)
AKT
GPADH
IB: 90KDa
IB: 200KDa
IB:119KDa
IB:119KDa
IB:56KDa
IB:56KDa
IB: 36KDa
H1975
QSOX2-flag
Vector-flag
si-ITGB4
si-NC
ITGB4-200KDa
QSOX2-90KDa
GAPDH-36KDa
Marker
KDA
200
130
90
70
53
40
35
25
14

figure4C-WB

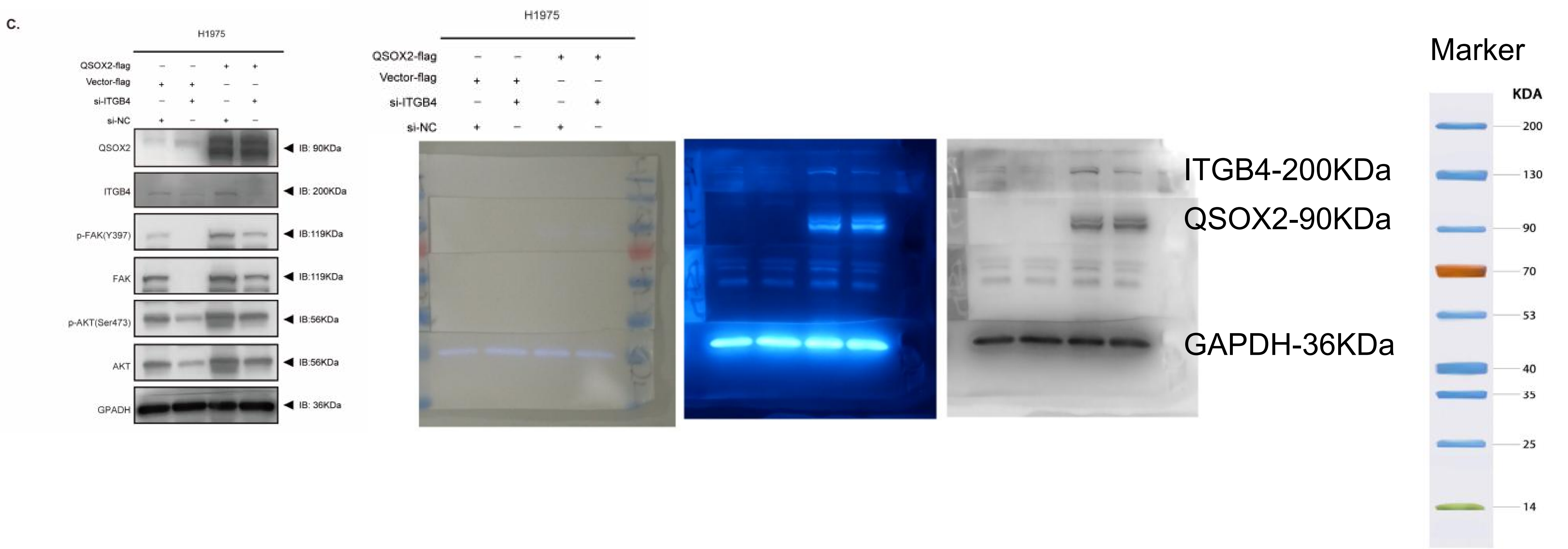
C.
H1975
QSOX2-flag
Vector-flag
si-ITGB4
si-NC
QSOX2
ITGB4
p-FAK(Y397)
FAK
p-AKT(Ser473)
AKT
GPADH
IB: 90KDa
IB: 200KDa
IB:119KDa
IB:119KDa
IB:56KDa
IB:56KDa
IB: 36KDa
ITGB4-200KDa
QSOX2-90KDa
GAPDH-36KDa
Marker
KDA
200
130
90
70
53
40
35
25
14

figure4C-WB

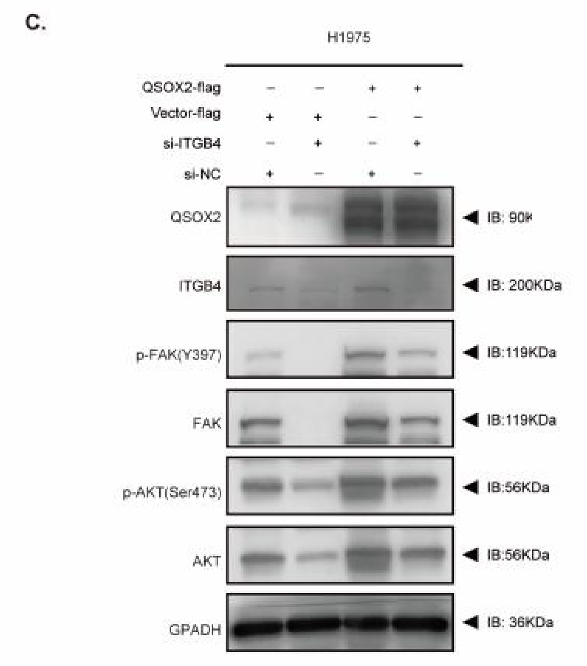

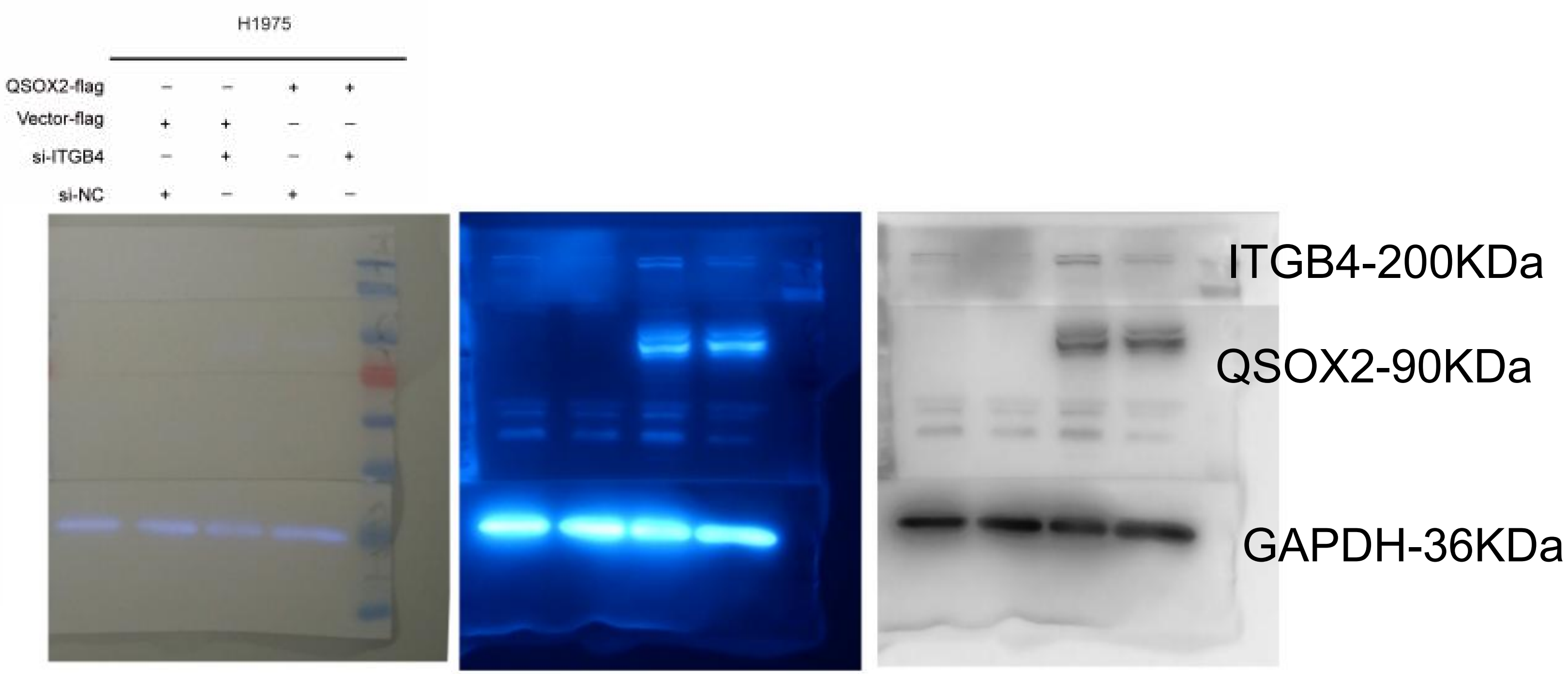

Marker

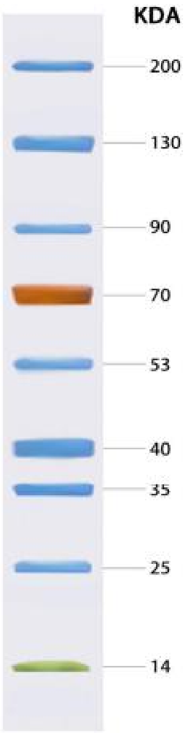

figure4C-WB

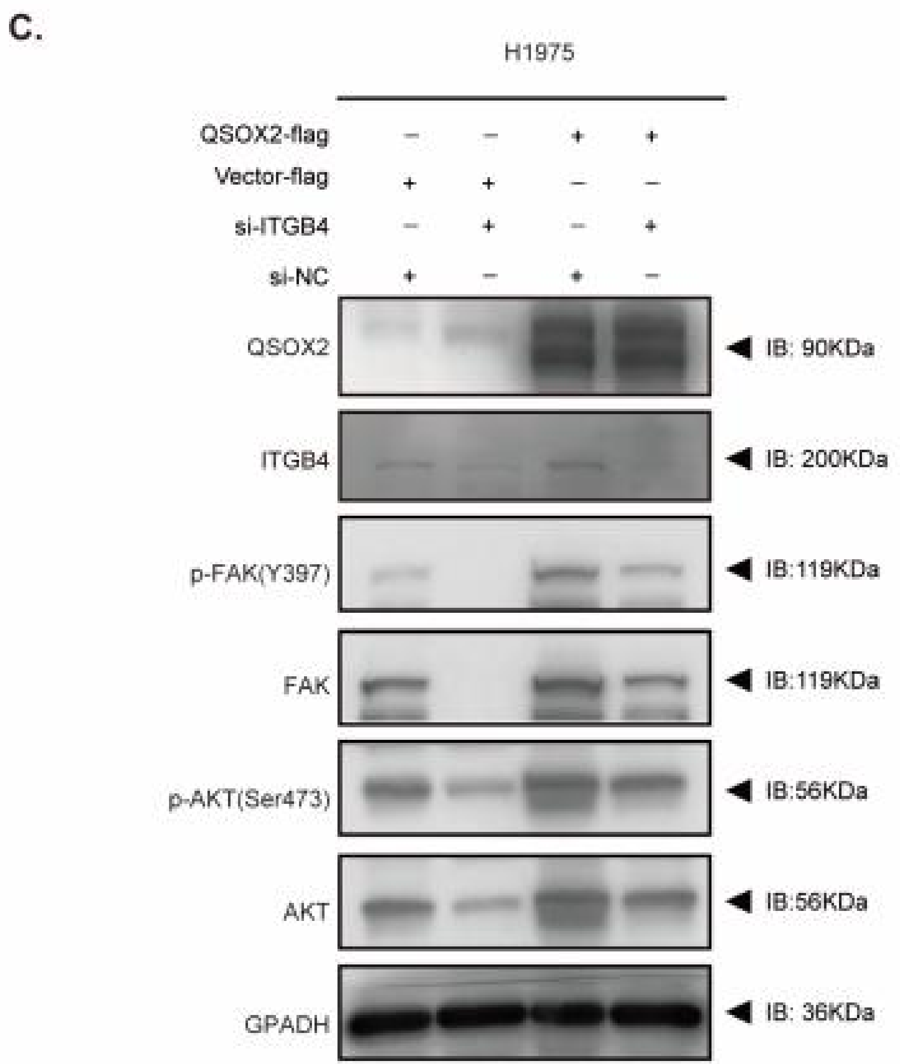

Marker

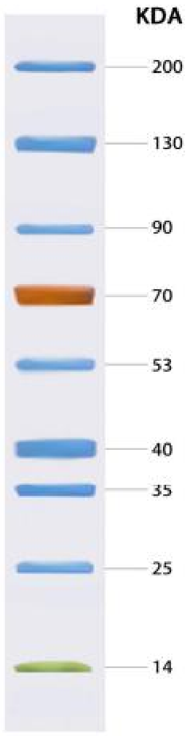

figure4C-WB

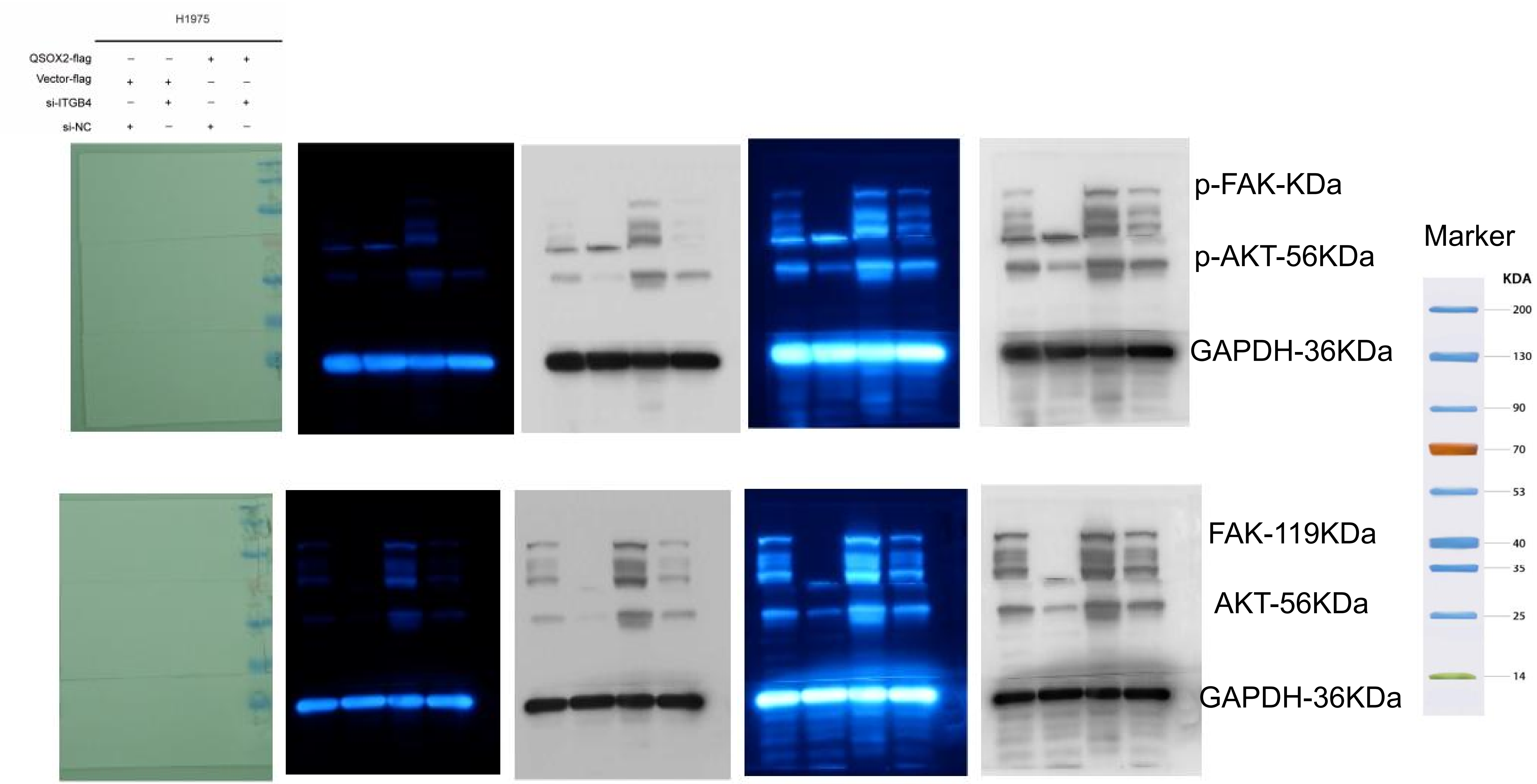
H1975
QSOX2-flag − − + +
Vector-flag + + − −
si-ITGB4 − + − +
si-NC + − + −
p-FAK-KDa
p-AKT-56KDa
GAPDH-36KDa
FAK-119KDa
AKT-56KDa
GAPDH-36KDa
Marker
KDA
200
130
90
70
53
40
35
25
14

figure4C-WB

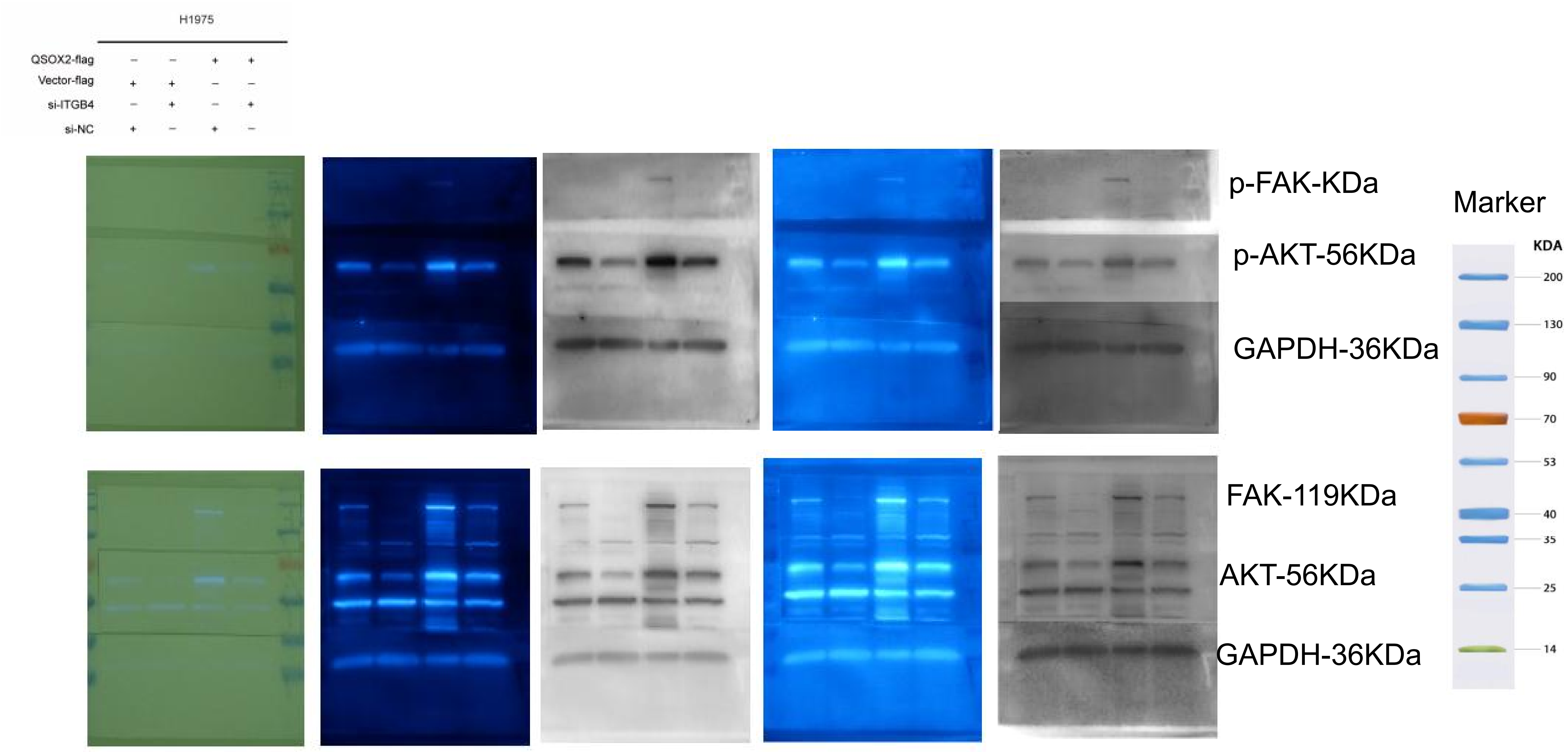
H1975
QSOX2-flag − − + +
Vector-flag + + − −
si-ITGB4 − + − +
si-NC + − + −
p-FAK-KDa
p-AKT-56KDa
GAPDH-36KDa
FAK-119KDa
AKT-56KDa
GAPDH-36KDa
Marker
KDA
200
130
90
70
53
40
35
25
14

figure4C-WB

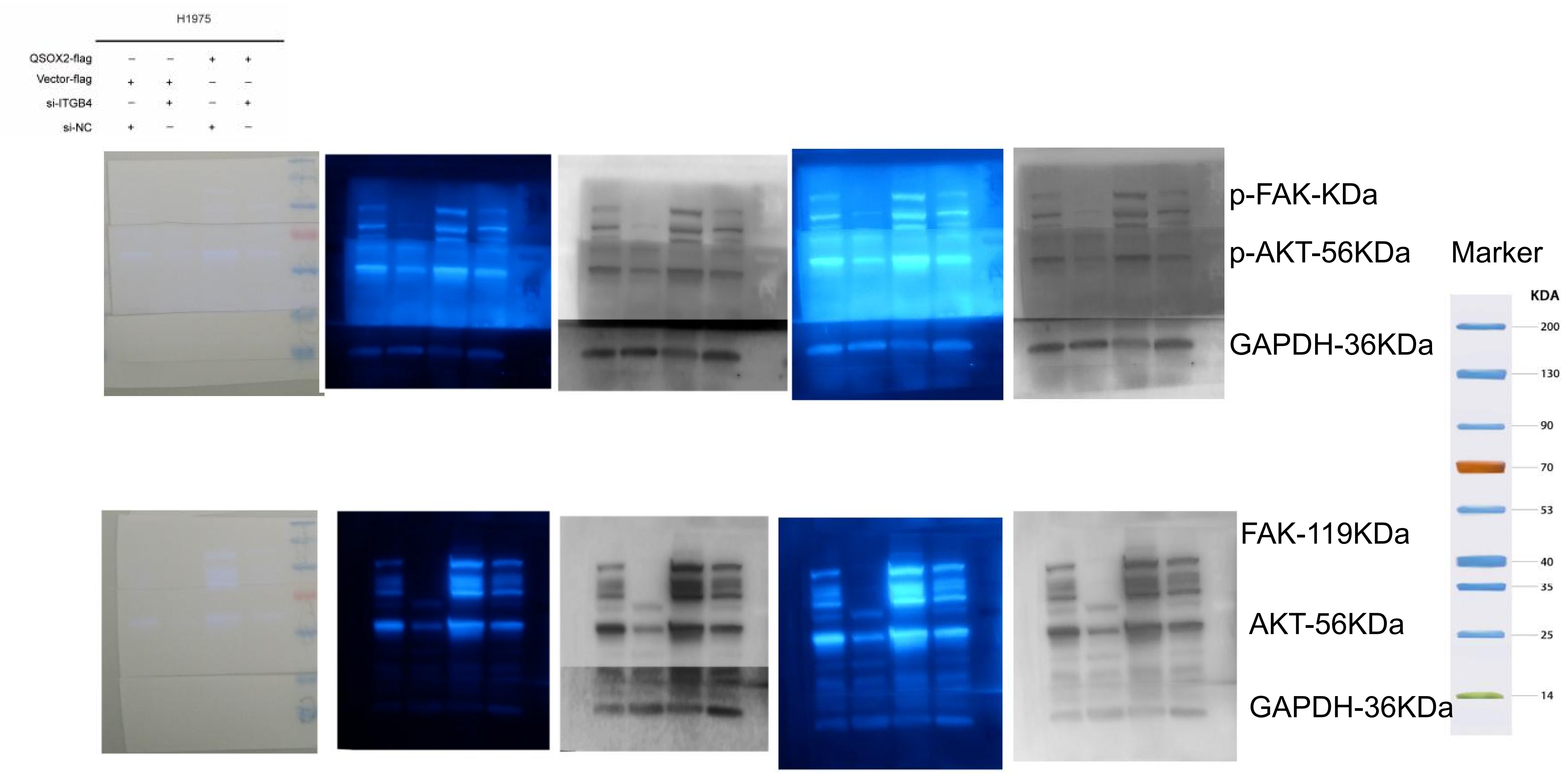
H1975
QSOX2-flag – – + +
Vector-flag + + – –
si-ITGB4 – + – +
si-NC + – + –
p-FAK-KDa
p-AKT-56KDa
GAPDH-36KDa
FAK-119KDa
AKT-56KDa
GAPDH-36KDa
Marker
KDA
200
130
90
70
53
40
35
25
14

fugure5A-Co-IP

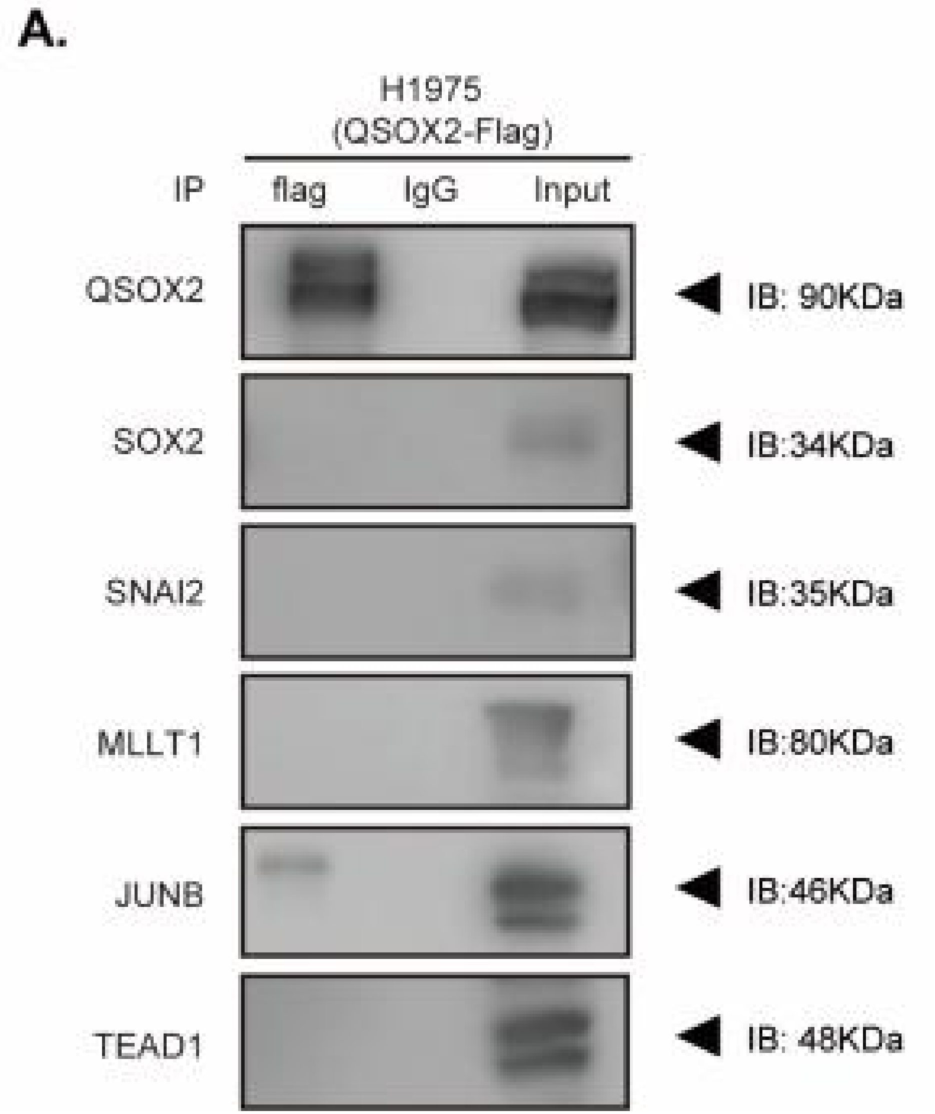

QSOX2	90KDa
JUNB	46KDa
SOX2	34KDa
SNAI2	35KDa
TEAD1	48KDa
MLLT1	80KDa

Marker

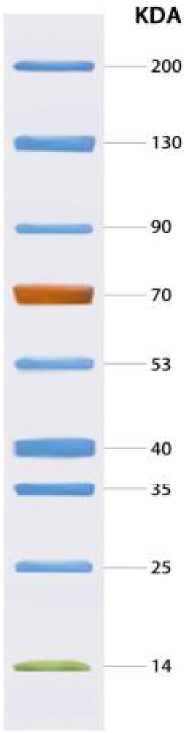

fugure5A-Co-IP

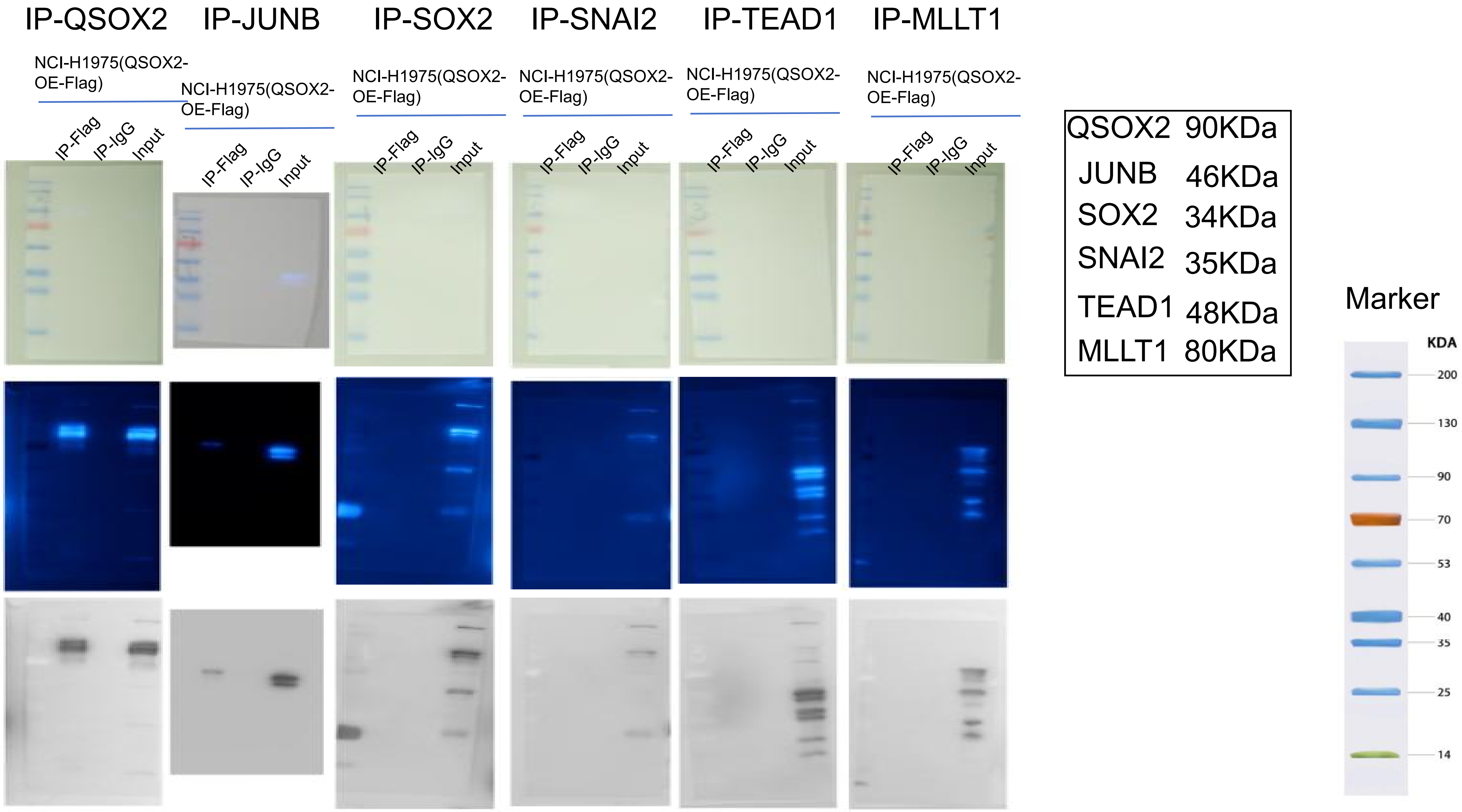

fugure5A-Co-IP

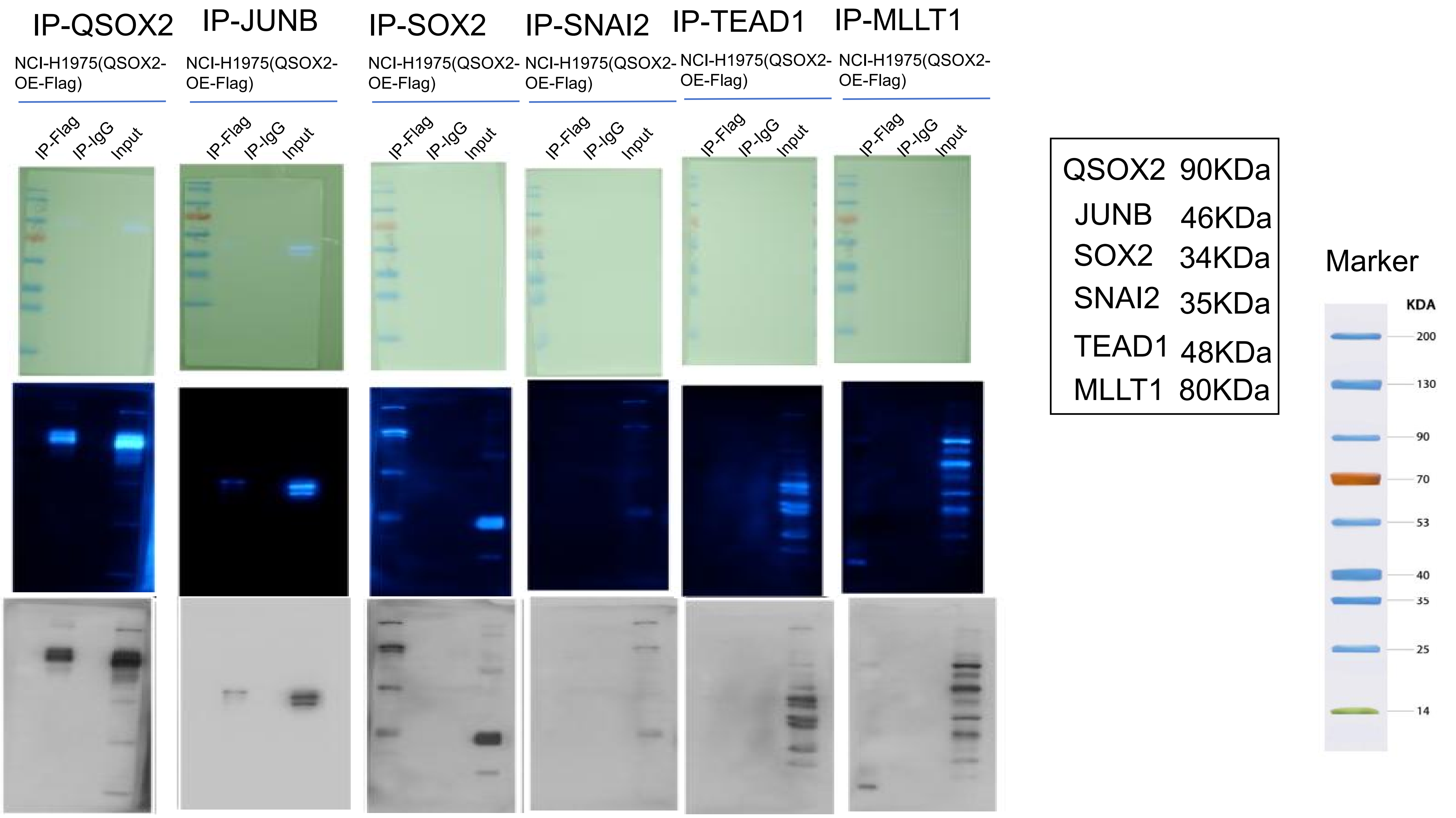

fugure5
A-Co-IP

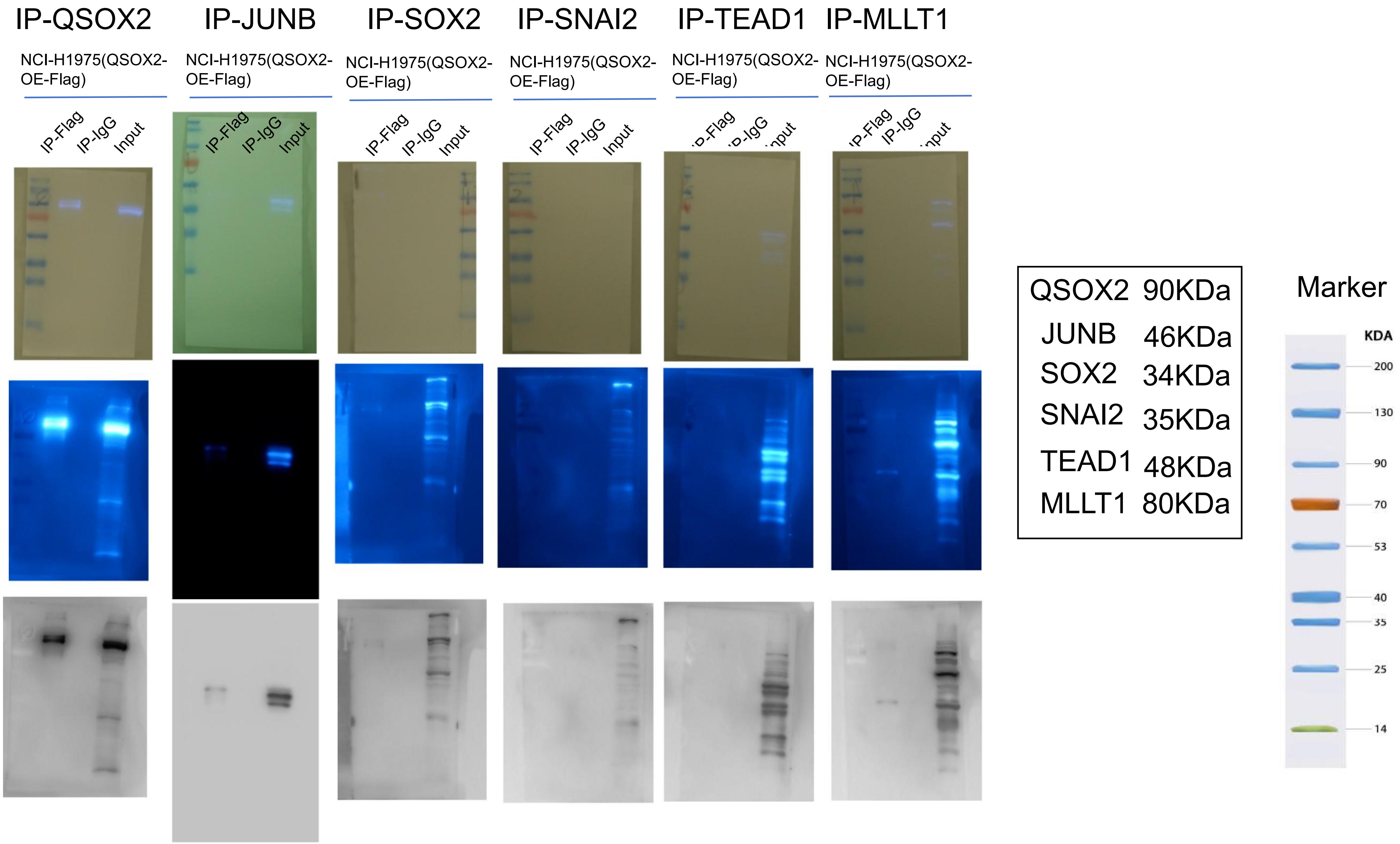

figure5E-ChIP-PCR

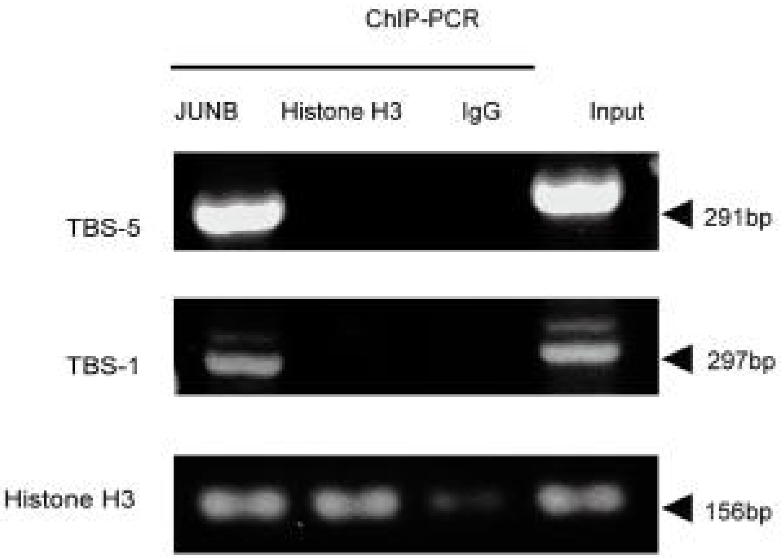

3%的 Agarose 电泳图

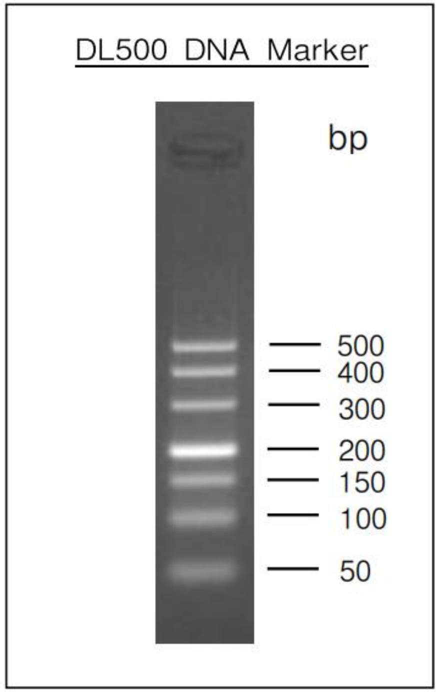

TBS-5:291bp

TBS-1:297bp

Histone H3:157bp

figure5E-ChIP-PCR

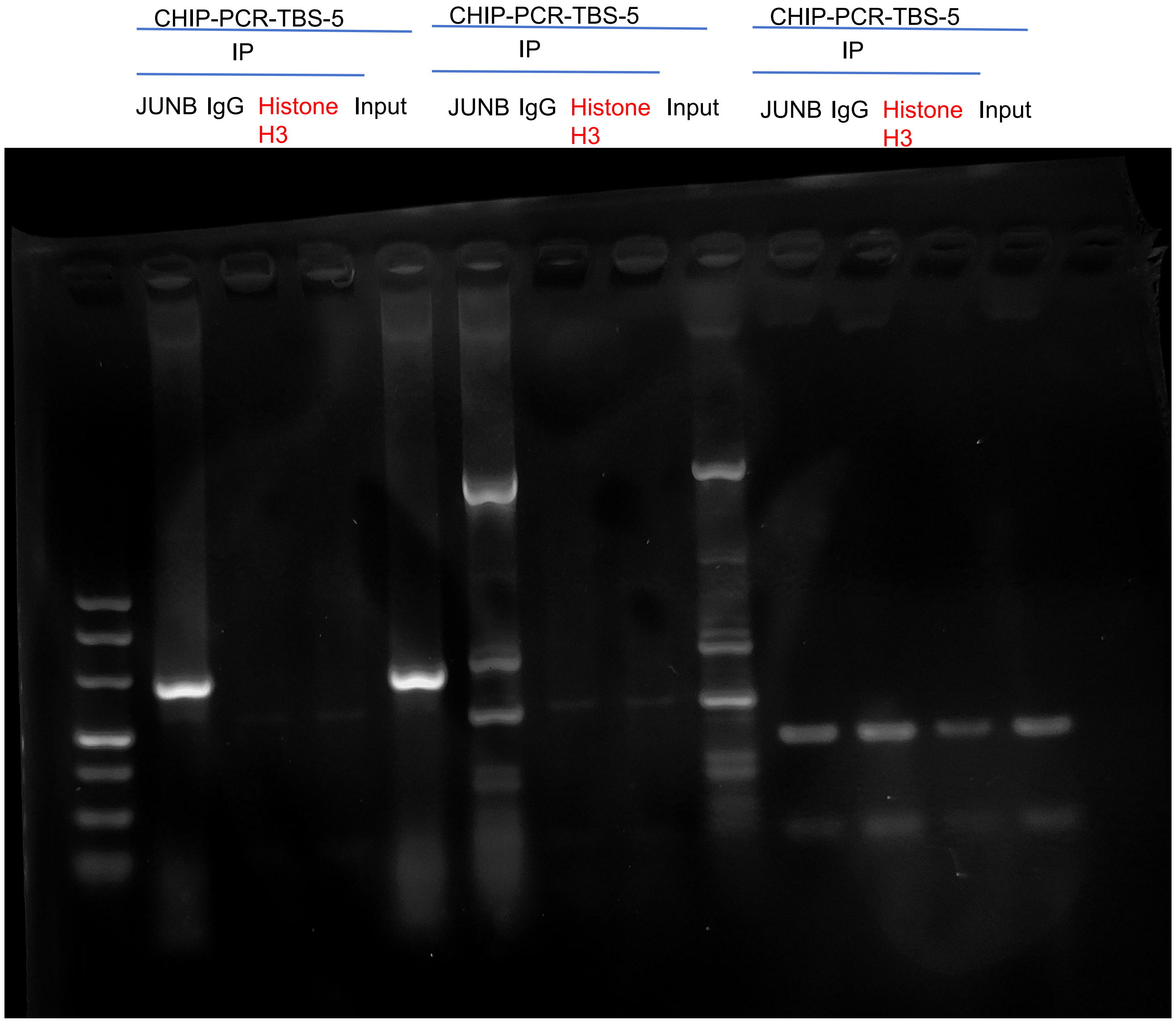

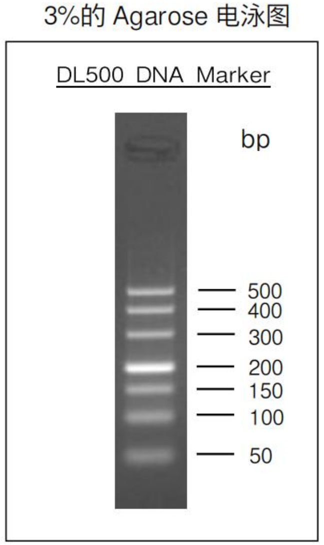

TBS-5:291bp

TBS-1:297bp

Histone H3:157bp

figure5E-ChIP-PCR

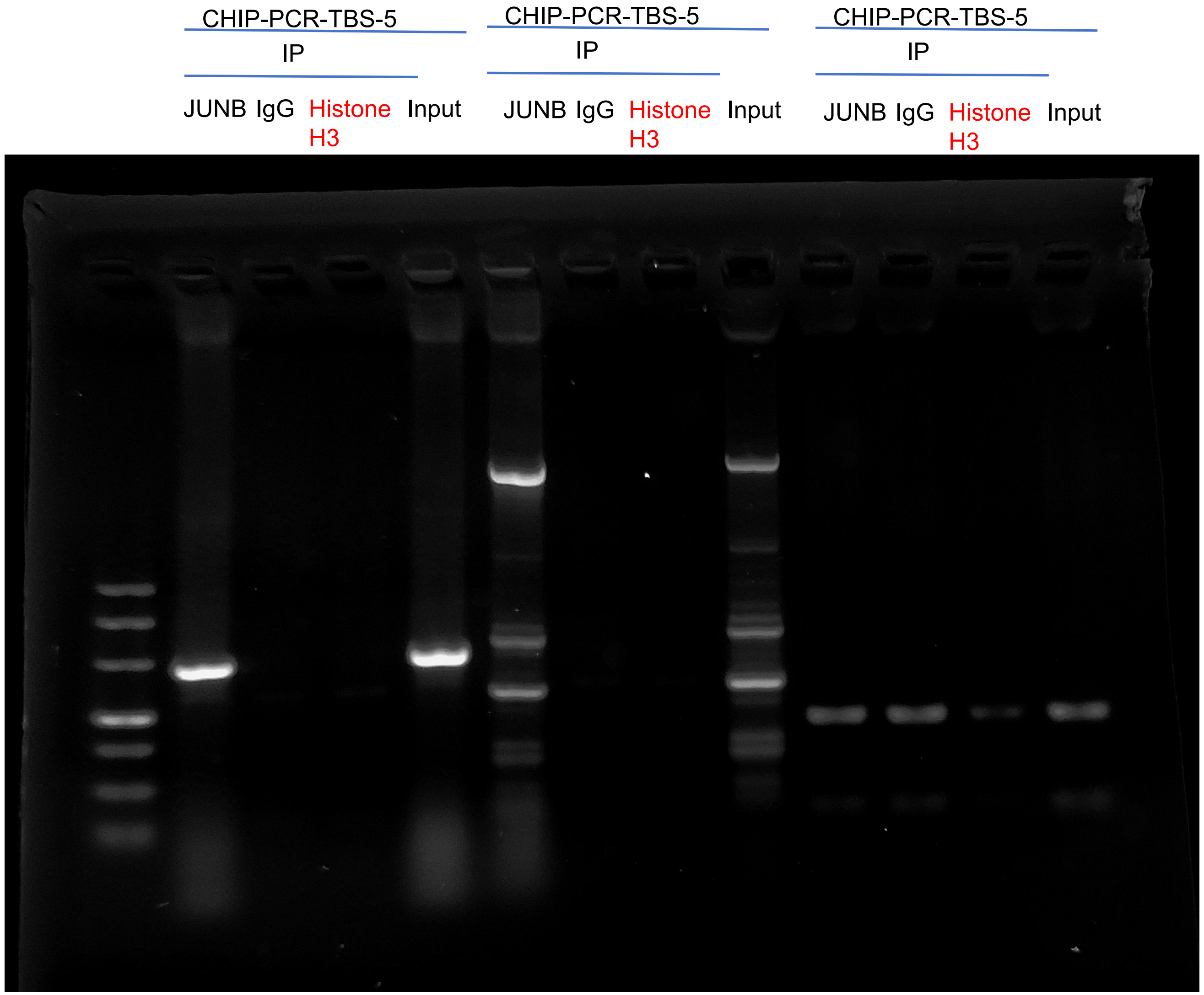

3%的 Agarose 电泳图

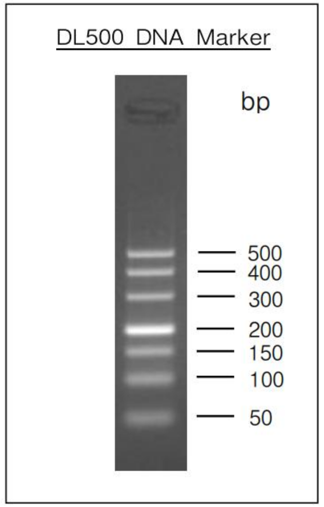

TBS-5:291bp

TBS-1:297bp

Histone H3:157bp

figure5E-ChIP-PCR

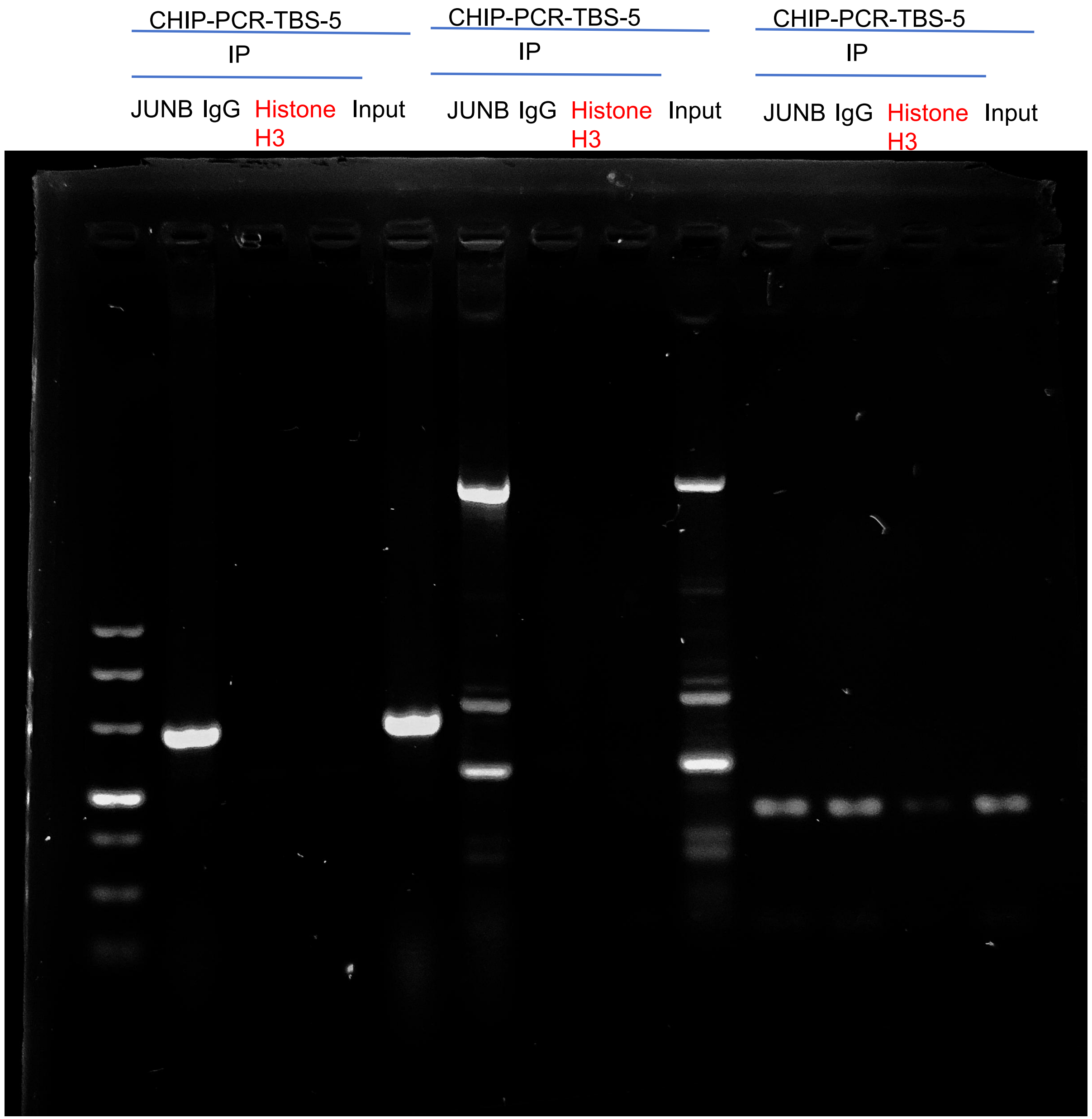

3%的 Agarose 电泳图

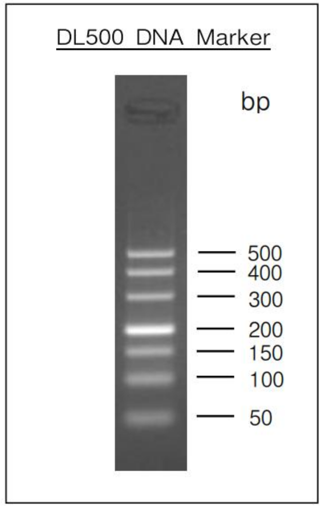

TBS-5:291bp

TBS-1:297bp

Histone H3:157bp

fugure6C-Co-IP

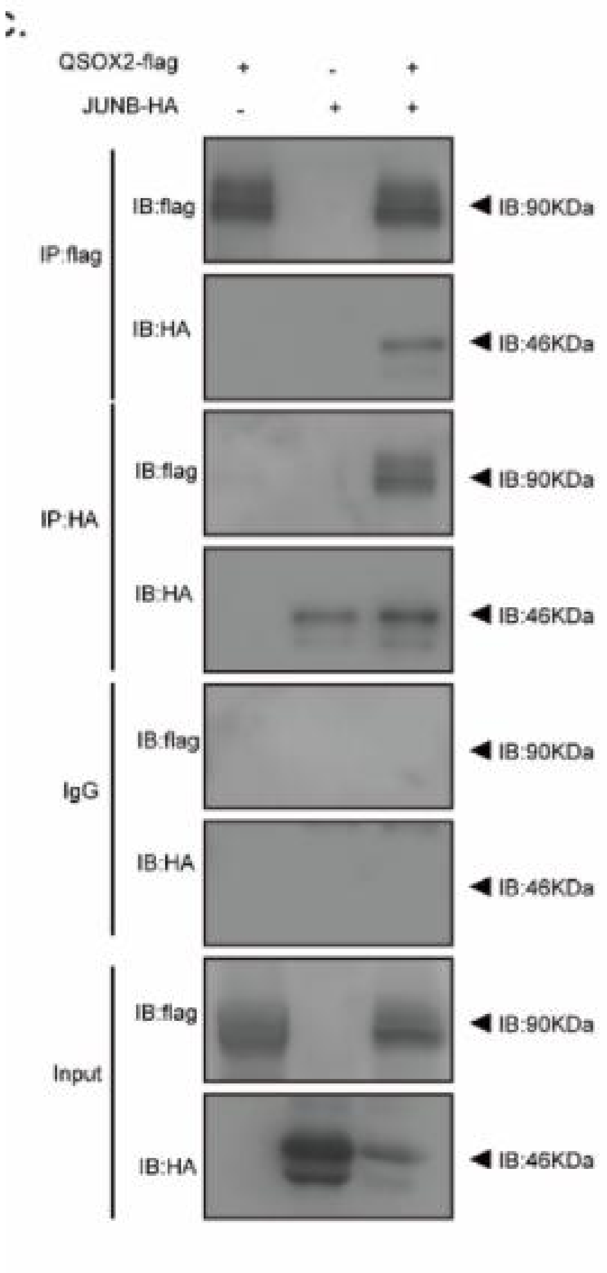

fugure6C-Co-IP

IP-HA

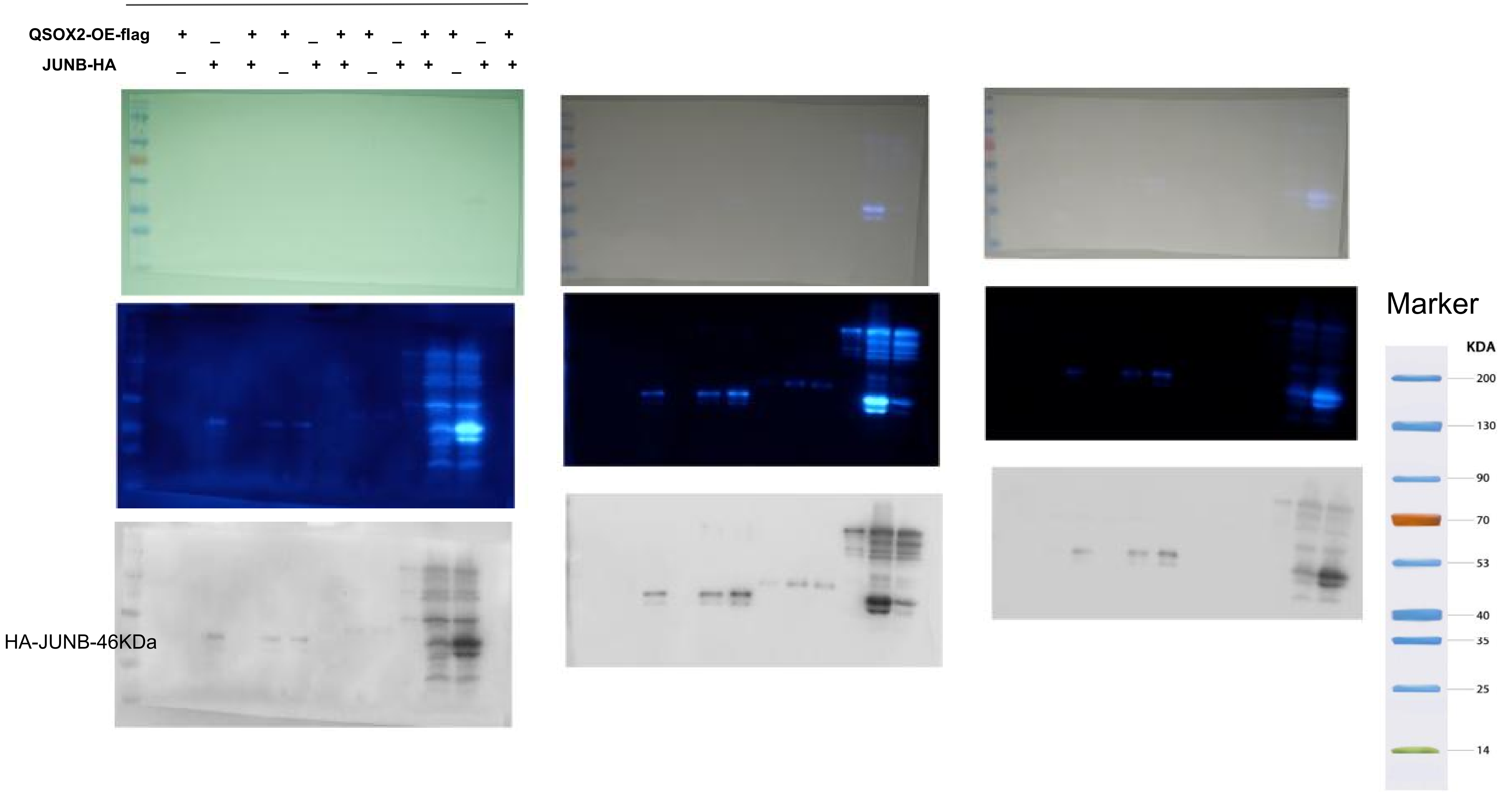

fugure6C-Co-IP

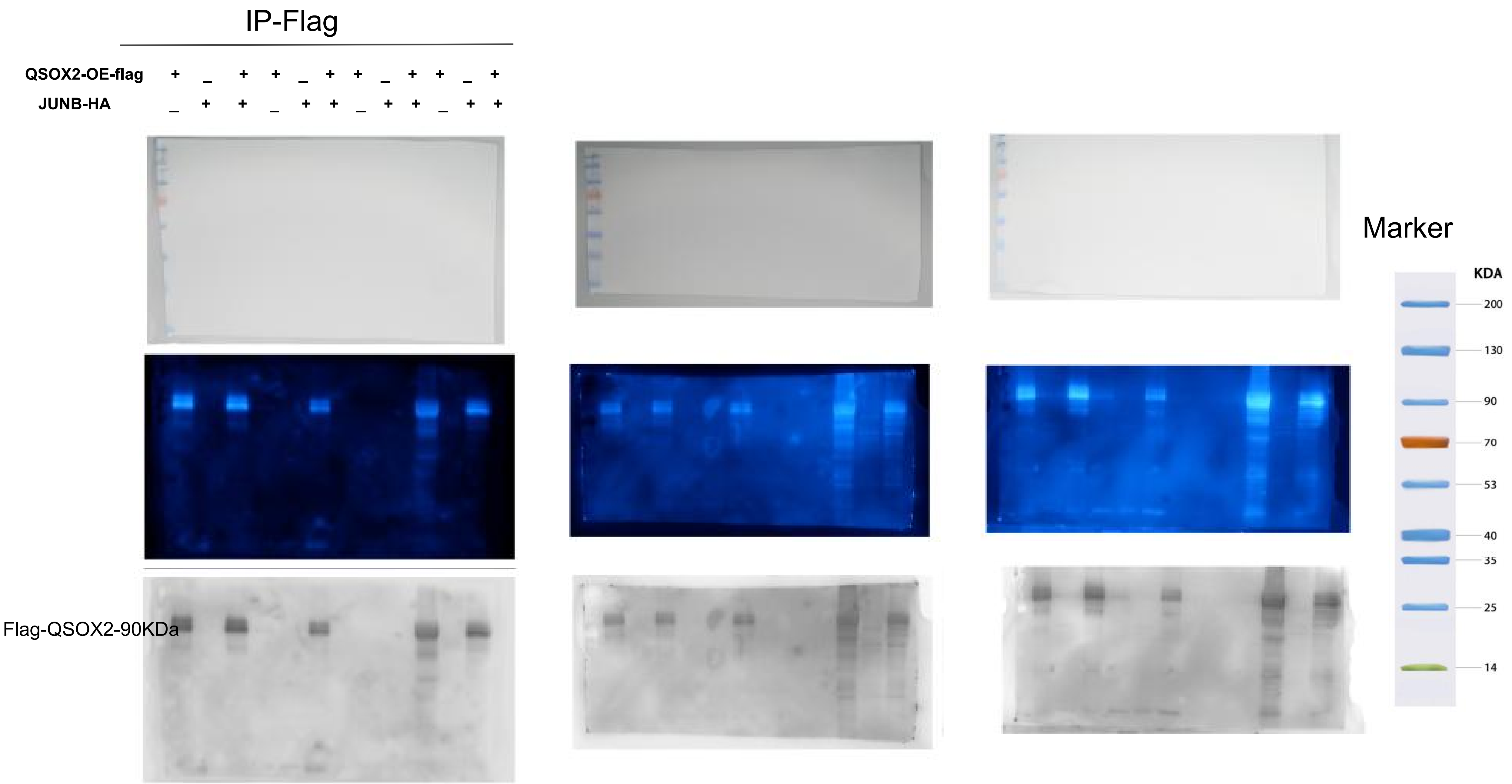

figure6D-WB

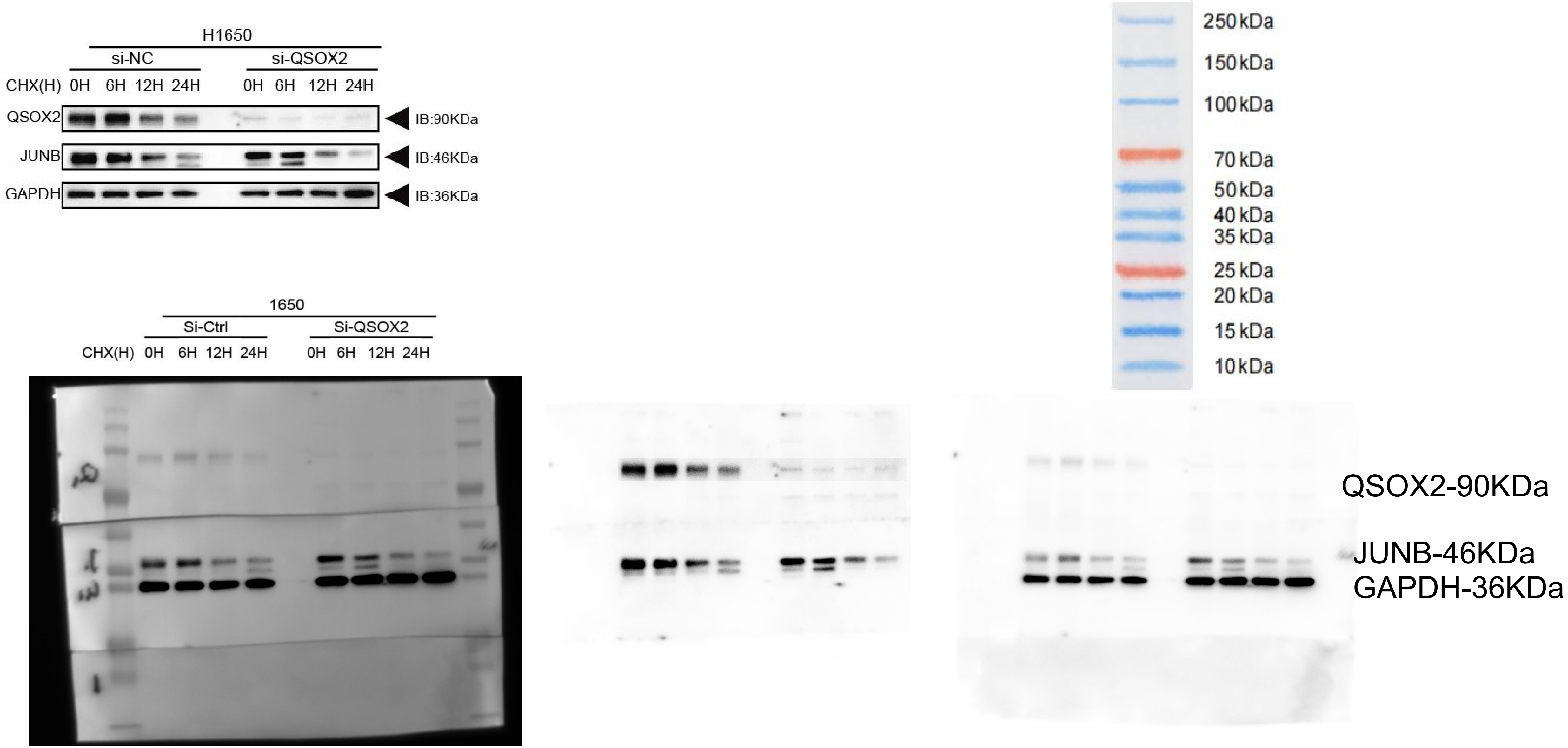
H1650
si-NC
si-QSOX2
CHX(H) 0H 6H 12H 24H 0H 6H 12H 24H
QSOX2
JUNB
GAPDH
IB:90KDa
IB:46KDa
IB:36KDa
1650
Si-Ctrl
Si-QSOX2
CHX(H) 0H 6H 12H 24H 0H 6H 12H 24H
250kDa
150kDa
100kDa
70kDa
50kDa
40kDa
35kDa
25kDa
20kDa
15kDa
10kDa
QSOX2-90KDa
JUNB-46KDa
GAPDH-36KDa

figure6D-WB

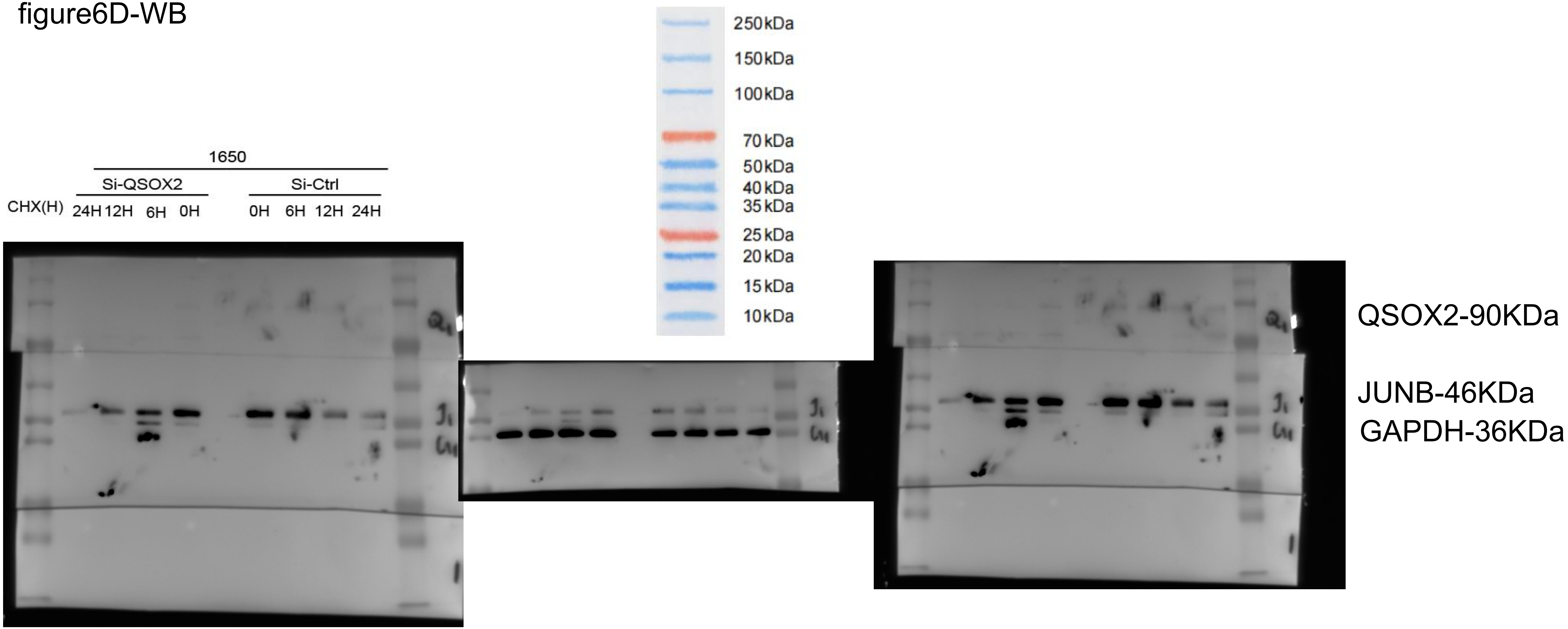

250kDa
150kDa
100kDa
70kDa
50kDa
40kDa
35kDa
25kDa
20kDa
15kDa
10kDa
1650
Si-QSOX2
Si-Ctrl
CHX(H)
24H 12H 6H 0H
0H 6H 12H 24H
QSOX2-90KDa
JUNB-46KDa
GAPDH-36KDa

figure6D-WB

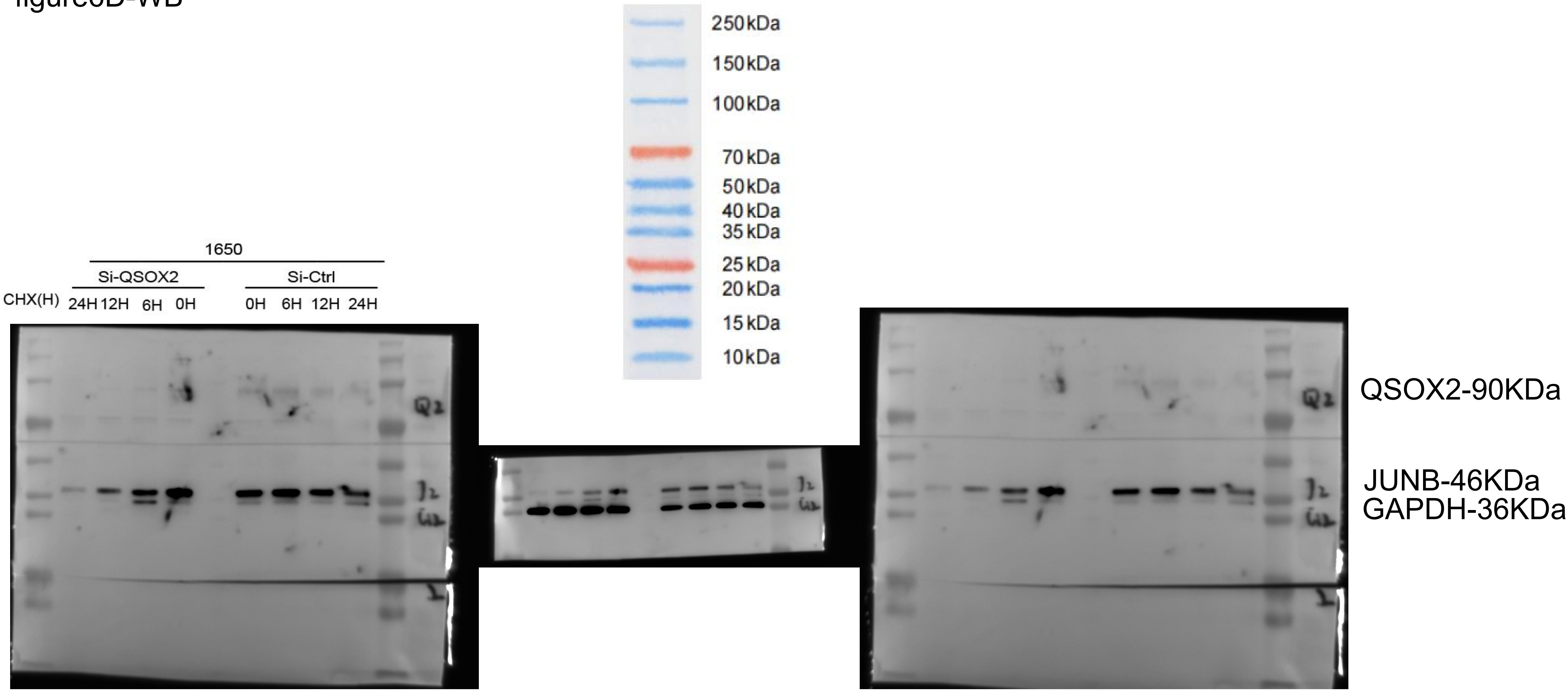
250kDa
150kDa
100kDa
70kDa
50kDa
40kDa
35kDa
25kDa
20kDa
15kDa
10kDa
1650
Si-QSOX2
Si-Ctrl
CHX(H)
24H 12H 6H 0H
0H 6H 12H 24H
QSOX2-90KDa
JUNB-46KDa
GAPDH-36KDa

figure6E-WB

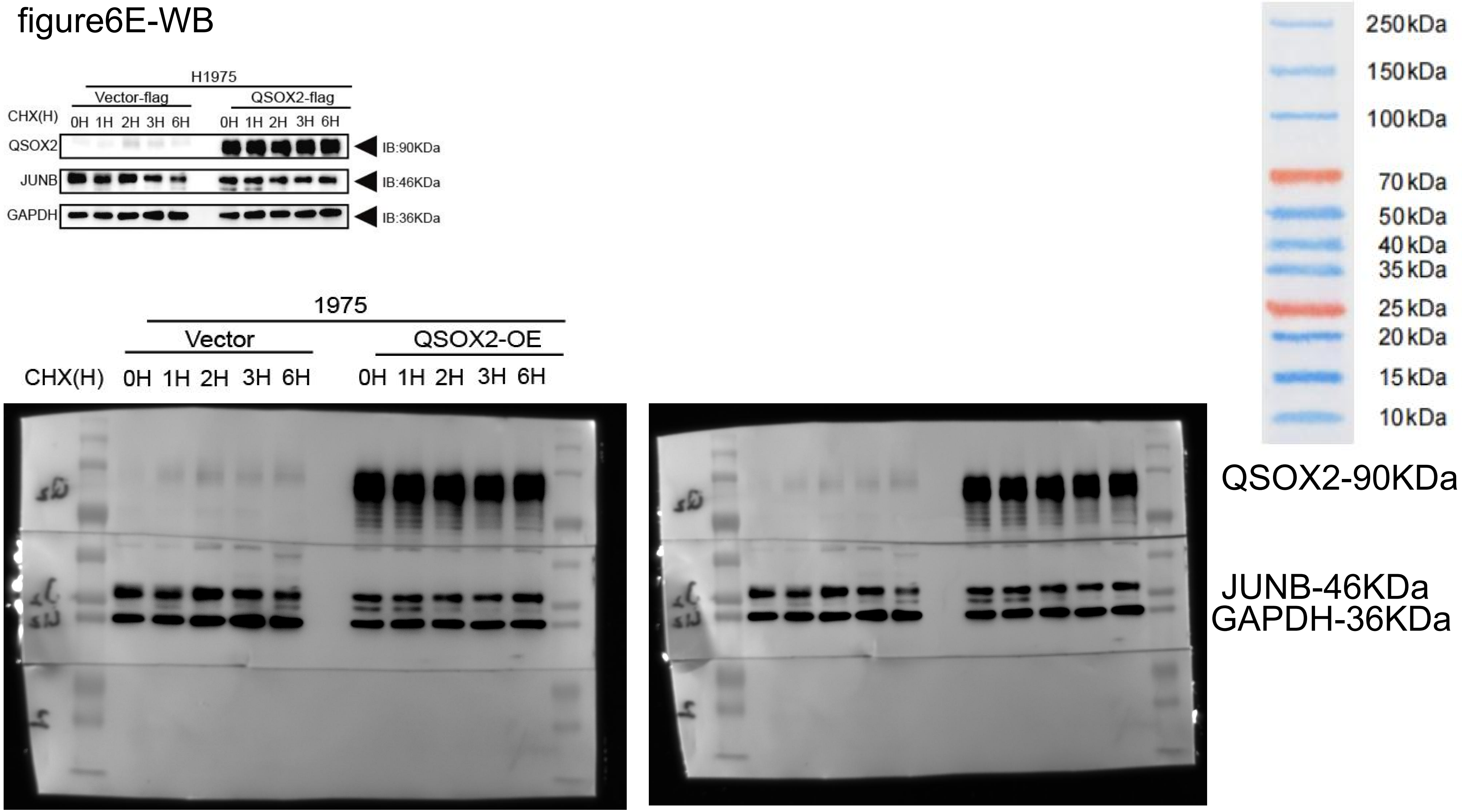
H1975
Vector-flag
QSOX2-flag
CHX(H) 0H 1H 2H 3H 6H 0H 1H 2H 3H 6H
QSOX2
JUNB
GAPDH
IB:90KDa
IB:46KDa
IB:36KDa
1975
Vector
QSOX2-OE
CHX(H) 0H 1H 2H 3H 6H 0H 1H 2H 3H 6H
250kDa
150kDa
100kDa
70kDa
50kDa
40kDa
35kDa
25kDa
20kDa
15kDa
10kDa
QSOX2-90KDa
JUNB-46KDa
GAPDH-36KDa

figure6E-WB

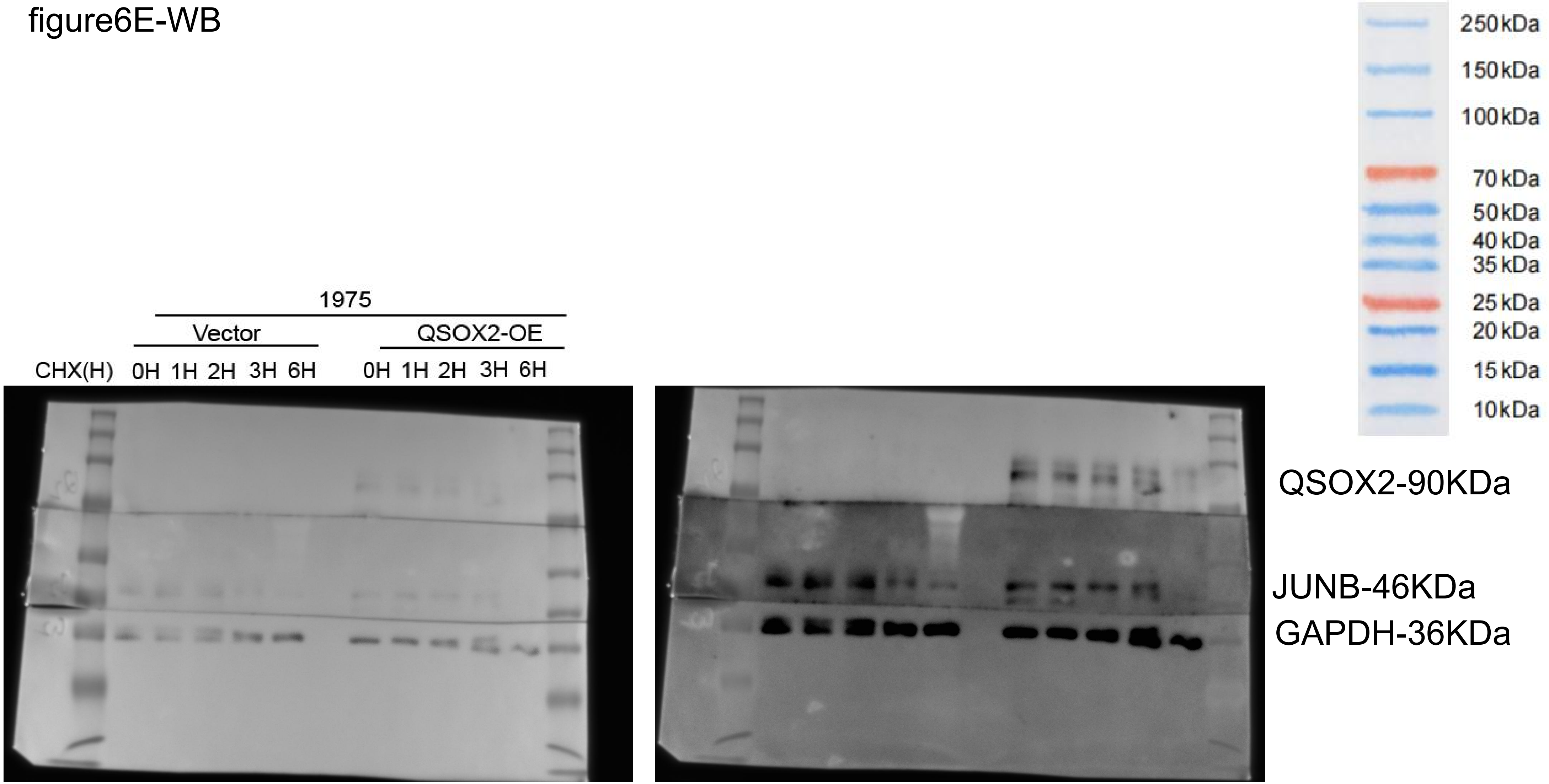

1975
Vector
QSOX2-OE
CHX(H)
0H 1H 2H 3H 6H
0H 1H 2H 3H 6H
QSOX2-90KDa
JUNB-46KDa
GAPDH-36KDa
250kDa
150kDa
100kDa
70kDa
50kDa
40kDa
35kDa
25kDa
20kDa
15kDa
10kDa

figure6E-WB

1975

Vector | QSOX2-OE

CHX(H) 0H 1H 2H 3H 6H 0H 1H 2H 3H 6H

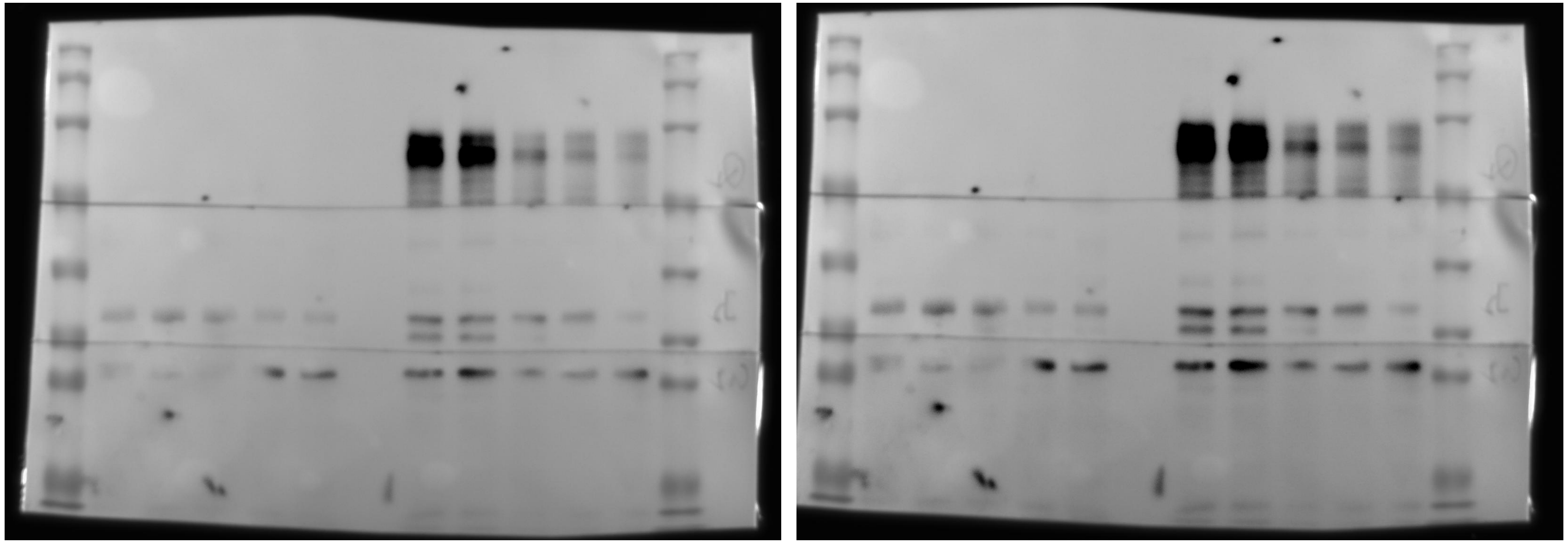

QSOX2-90KDa

JUNB-46KDa

GAPDH-36KDa

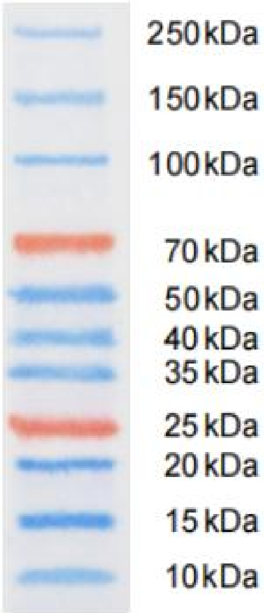

figure6F-WB

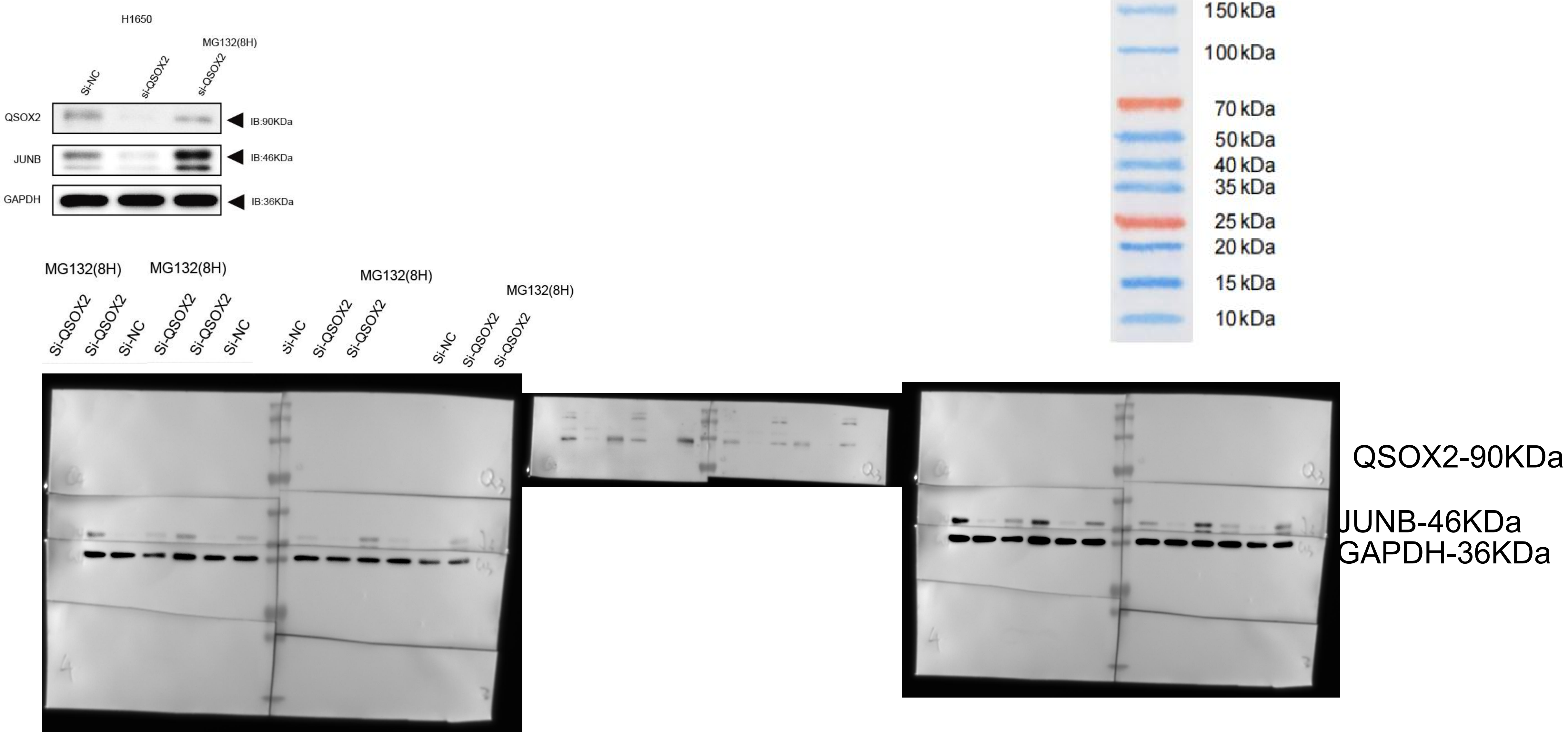
H1650
MG132(8H)
Si-NC
si-QSOX2
si-QSOX2
QSOX2
IB:90KDa
JUNB
IB:46KDa
GAPDH
IB:36KDa
250kDa
150kDa
100kDa
70kDa
50kDa
40kDa
35kDa
25kDa
20kDa
15kDa
10kDa
MG132(8H)
MG132(8H)
Si-QSOX2
Si-QSOX2
Si-NC
Si-QSOX2
Si-QSOX2
Si-NC
MG132(8H)
Si-NC
Si-QSOX2
Si-QSOX2
MG132(8H)
Si-NC
Si-QSOX2
Si-QSOX2
QSOX2-90KDa
JUNB-46KDa
GAPDH-36KDa

figure6G-WB

Marker

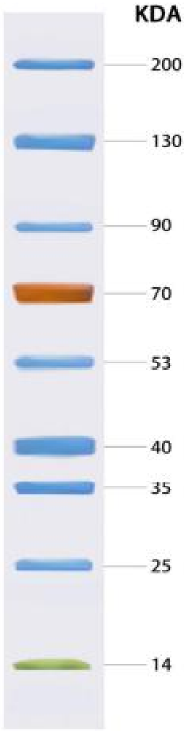

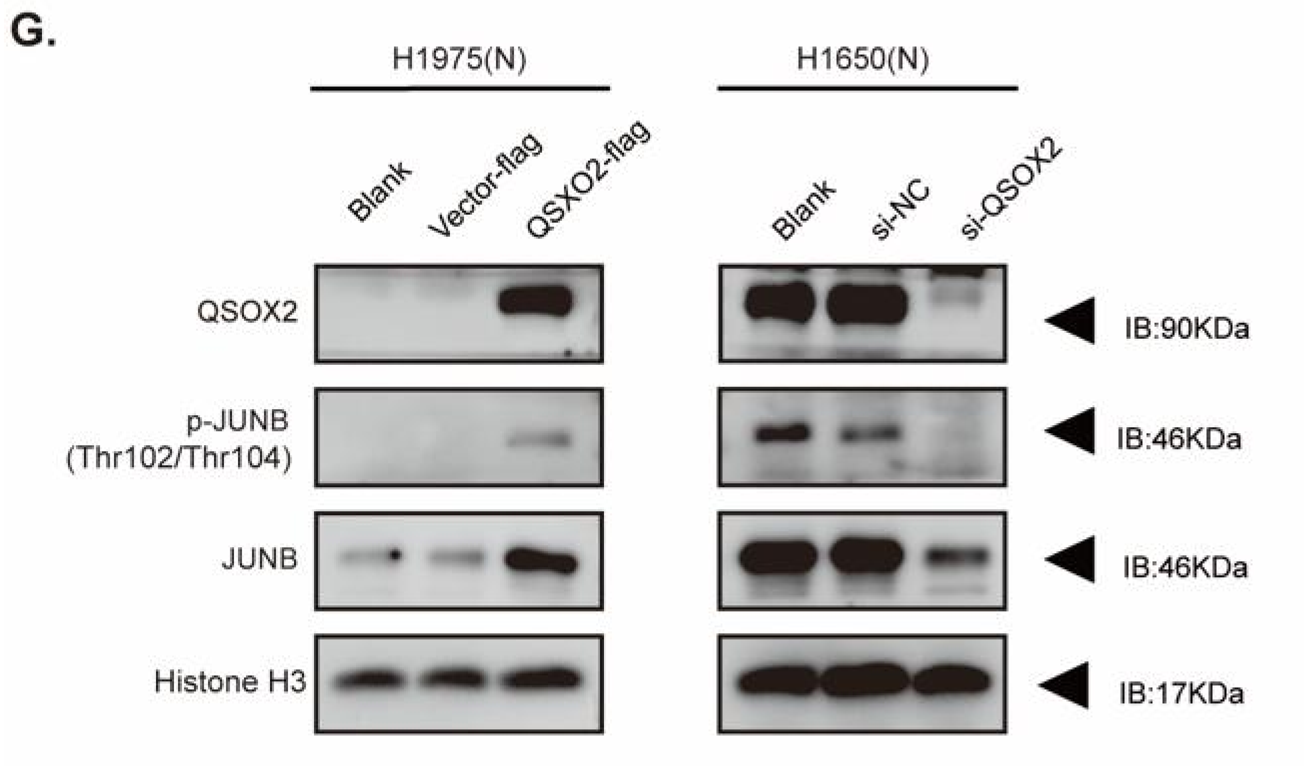

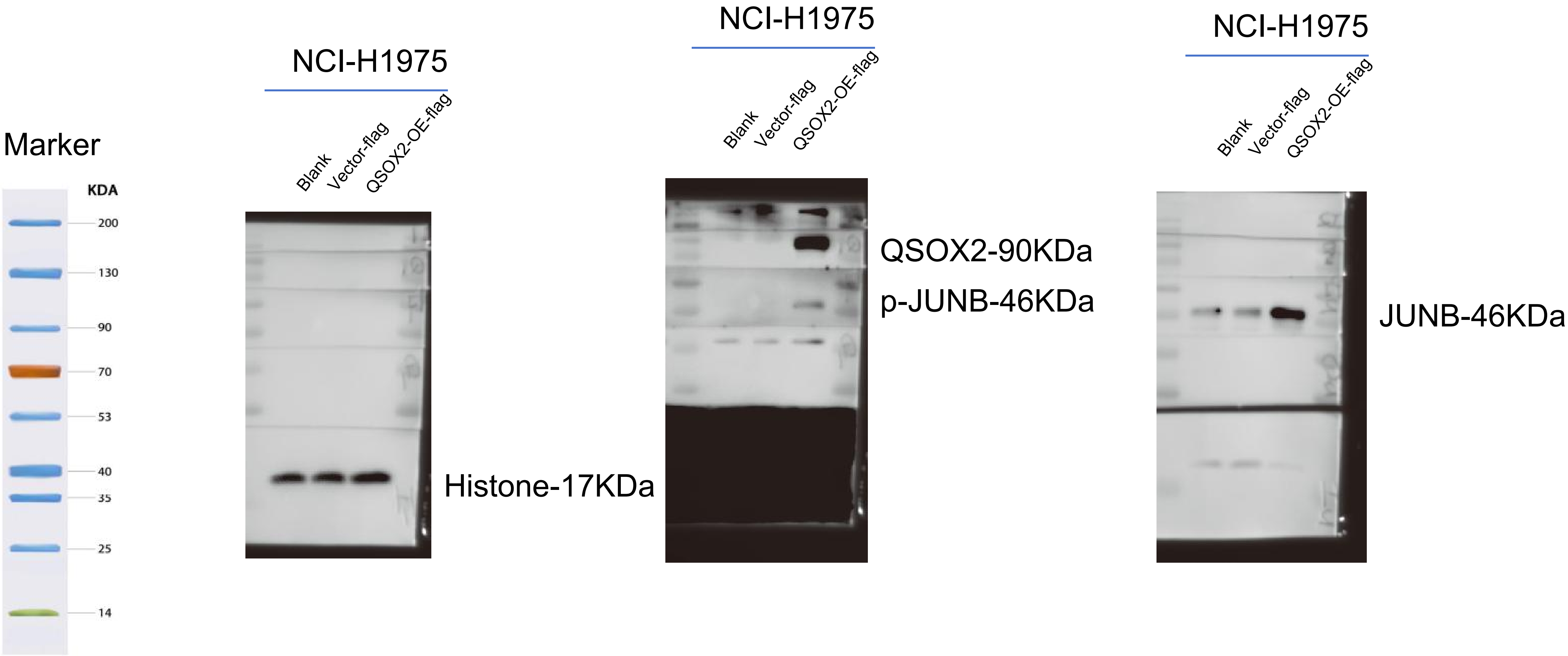
Marker
KDA
200
130
90
70
53
40
35
25
14
NCI-H1975
Blank
Vector-flag
QSOX2-OE-flag
Histone-17KDa
NCI-H1975
Blank
Vector-flag
QSOX2-OE-flag
QSOX2-90KDa
p-JUNB-46KDa
NCI-H1975
Blank
Vector-flag
QSOX2-OE-flag
JUNB-46KDa

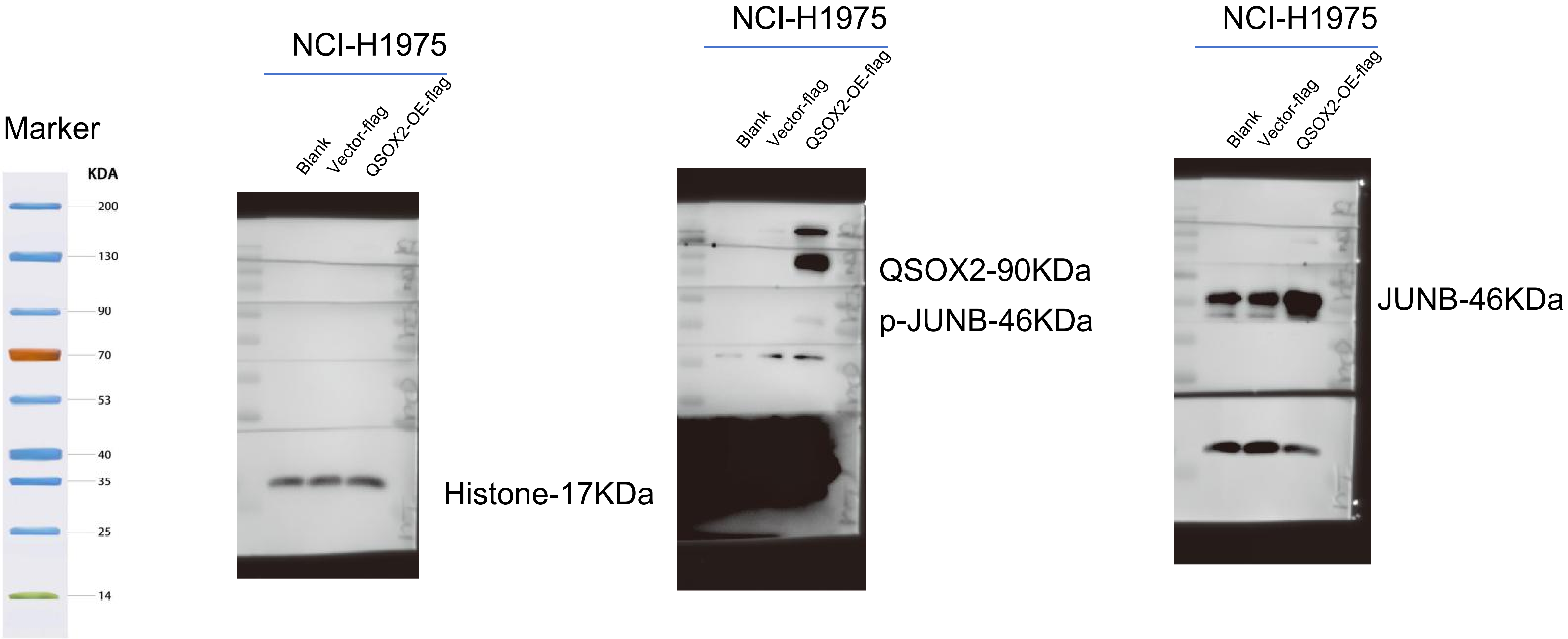

Marker
KDA
200
130
90
70
53
40
35
25
14
NCI-H1975
Blank
Vector-flag
QSOX2-OE-flag
Histone-17KDa
NCI-H1975
Blank
Vector-flag
QSOX2-OE-flag
QSOX2-90KDa
p-JUNB-46KDa
NCI-H1975
Blank
Vector-flag
QSOX2-OE-flag
JUNB-46KDa

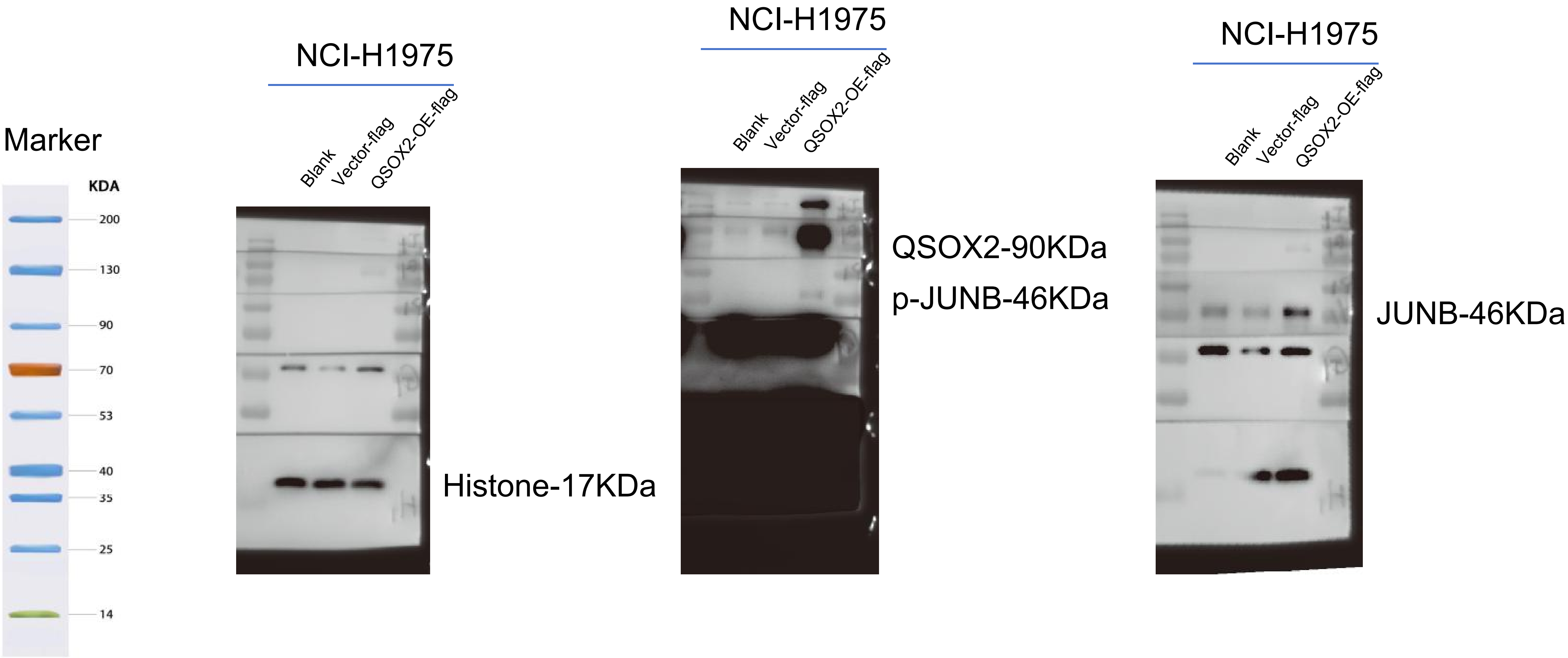
Marker
KDA
200
130
90
70
53
40
35
25
14
NCI-H1975
Blank
Vector-flag
QSOX2-OE-flag
Histone-17KDa
NCI-H1975
Blank
Vector-flag
QSOX2-OE-flag
QSOX2-90KDa
p-JUNB-46KDa
NCI-H1975
Blank
Vector-flag
QSOX2-OE-flag
JUNB-46KDa

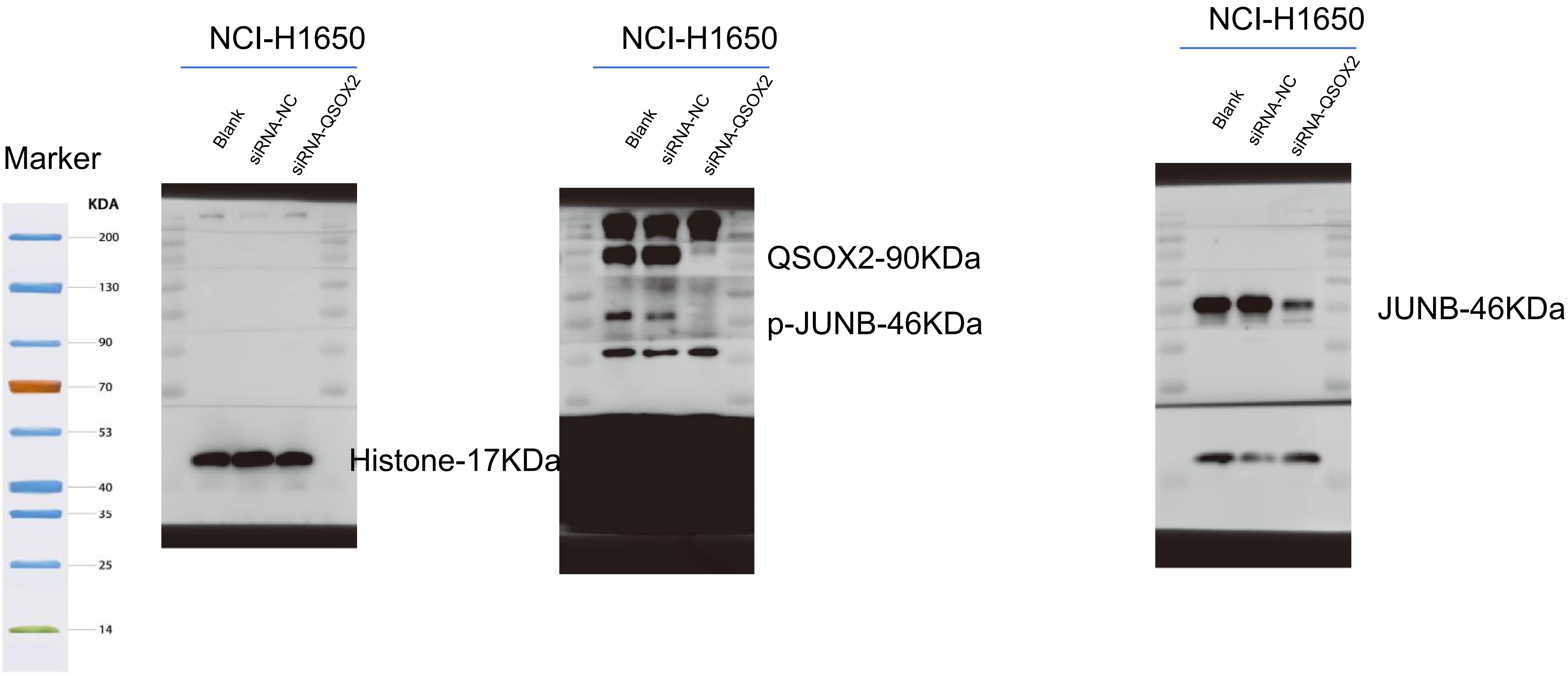
Marker
KDA
200
130
90
70
53
40
35
25
14
NCI-H1650
Blank
siRNA-NC
siRNA-QSOX2
Histone-17KDa
NCI-H1650
Blank
siRNA-NC
siRNA-QSOX2
QSOX2-90KDa
p-JUNB-46KDa
NCI-H1650
Blank
siRNA-NC
siRNA-QSOX2
JUNB-46KDa

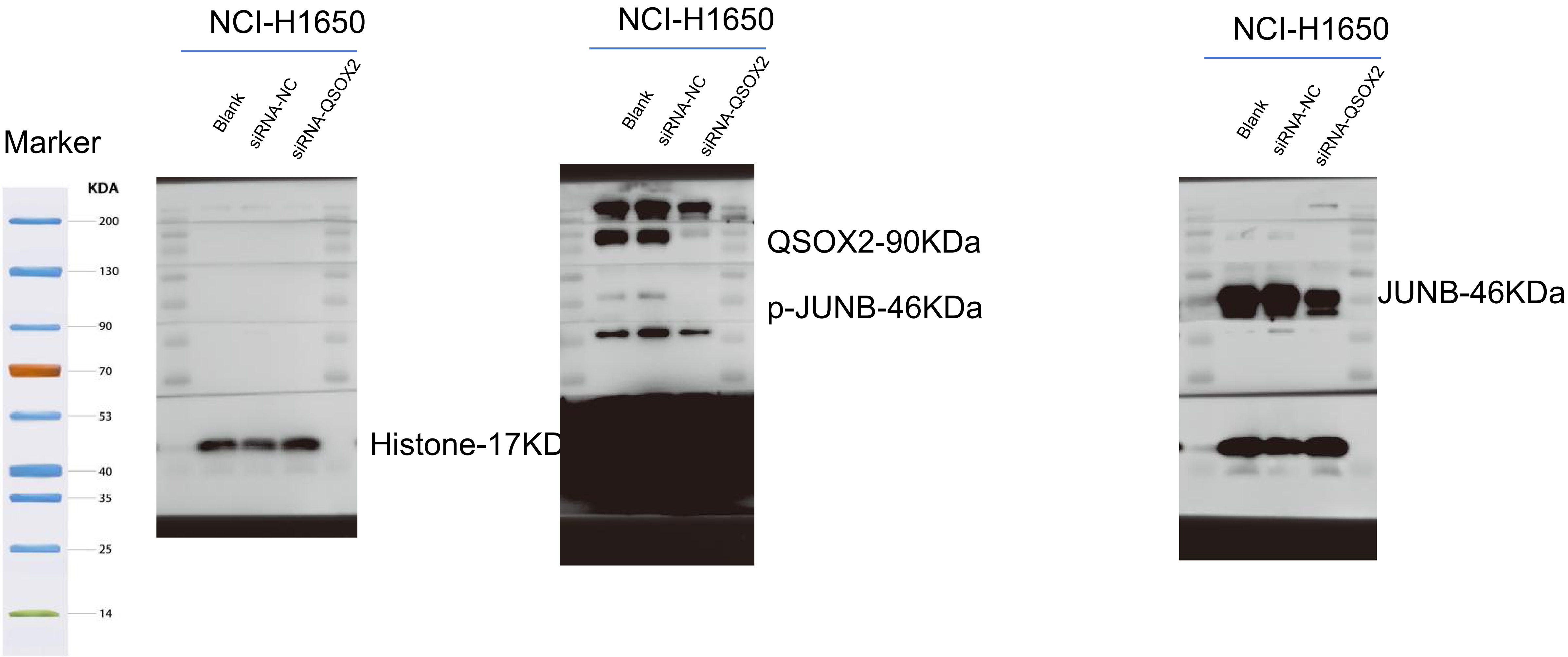

Marker
KDA
200
130
90
70
53
40
35
25
14
NCI-H1650
Blank
siRNA-NC
siRNA-QSOX2
Histone-17KD
NCI-H1650
Blank
siRNA-NC
siRNA-QSOX2
QSOX2-90KDa
p-JUNB-46KDa
NCI-H1650
Blank
siRNA-NC
siRNA-QSOX2
JUNB-46KDa

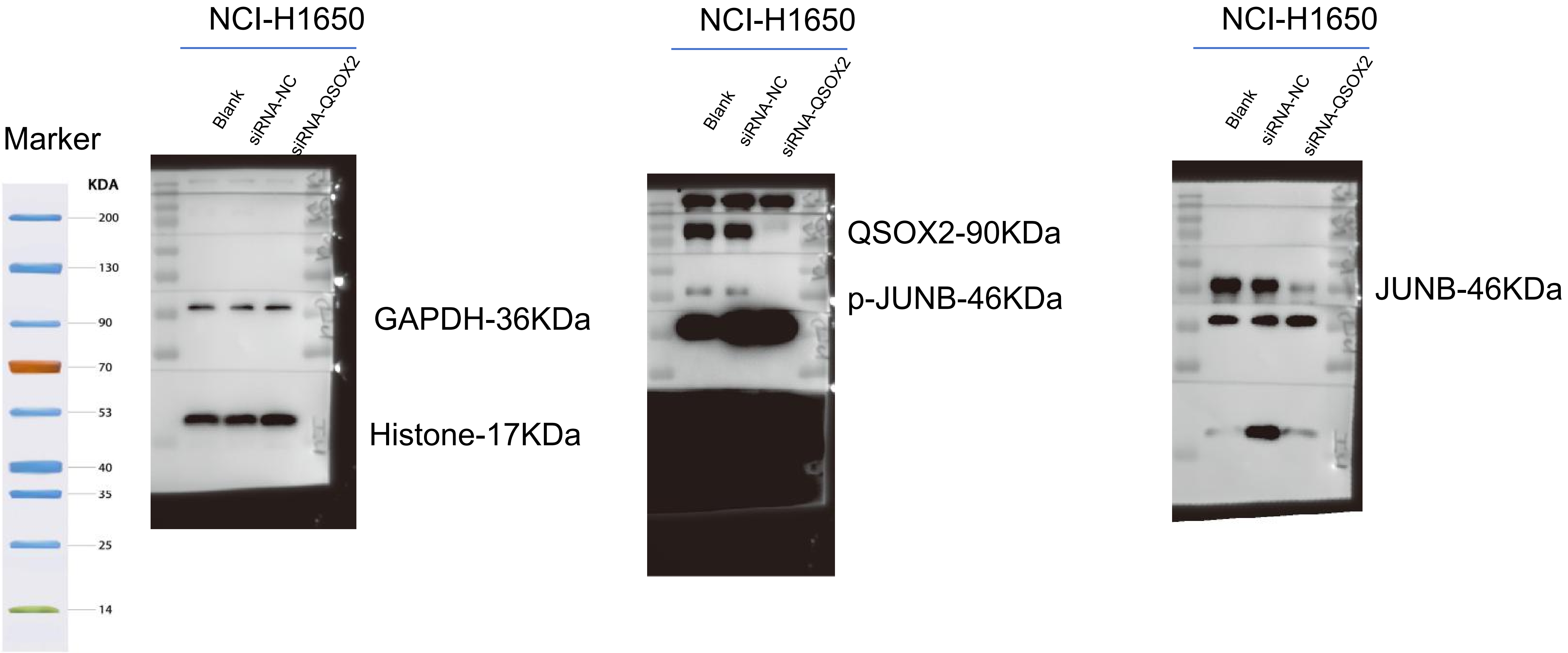
Marker
KDA
200
130
90
70
53
40
35
25
14
NCI-H1650
Blank
siRNA-NC
siRNA-QSOX2
GAPDH-36KDa
Histone-17KDa
NCI-H1650
Blank
siRNA-NC
siRNA-QSOX2
QSOX2-90KDa
p-JUNB-46KDa
NCI-H1650
Blank
siRNA-NC
siRNA-QSOX2
JUNB-46KDa

figure7C-WB

Marker

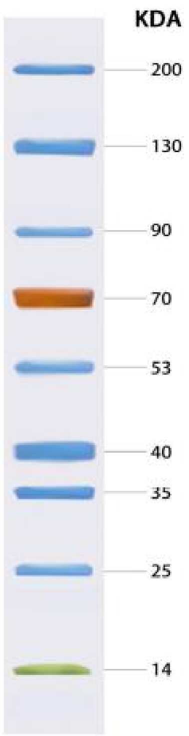

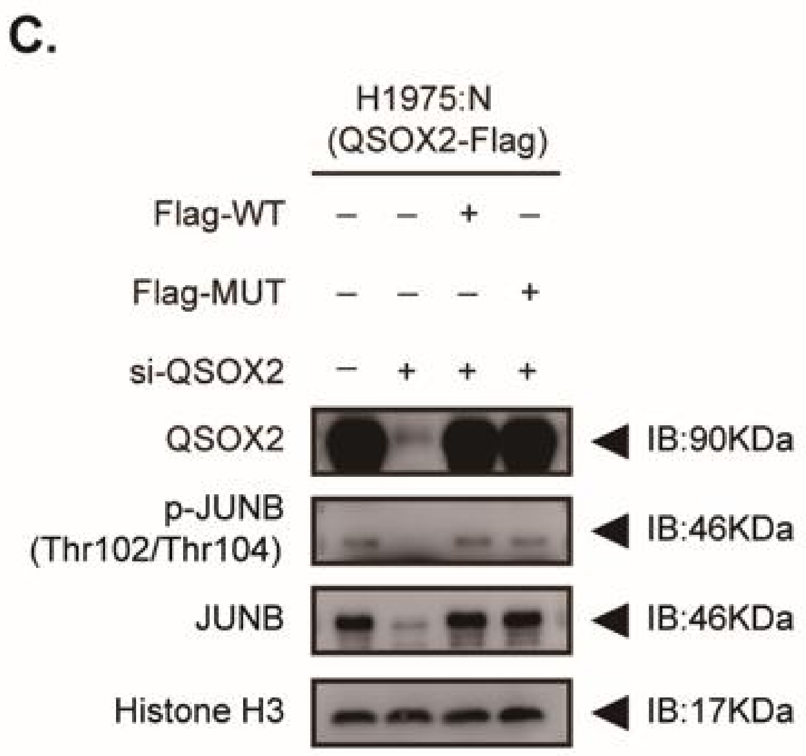

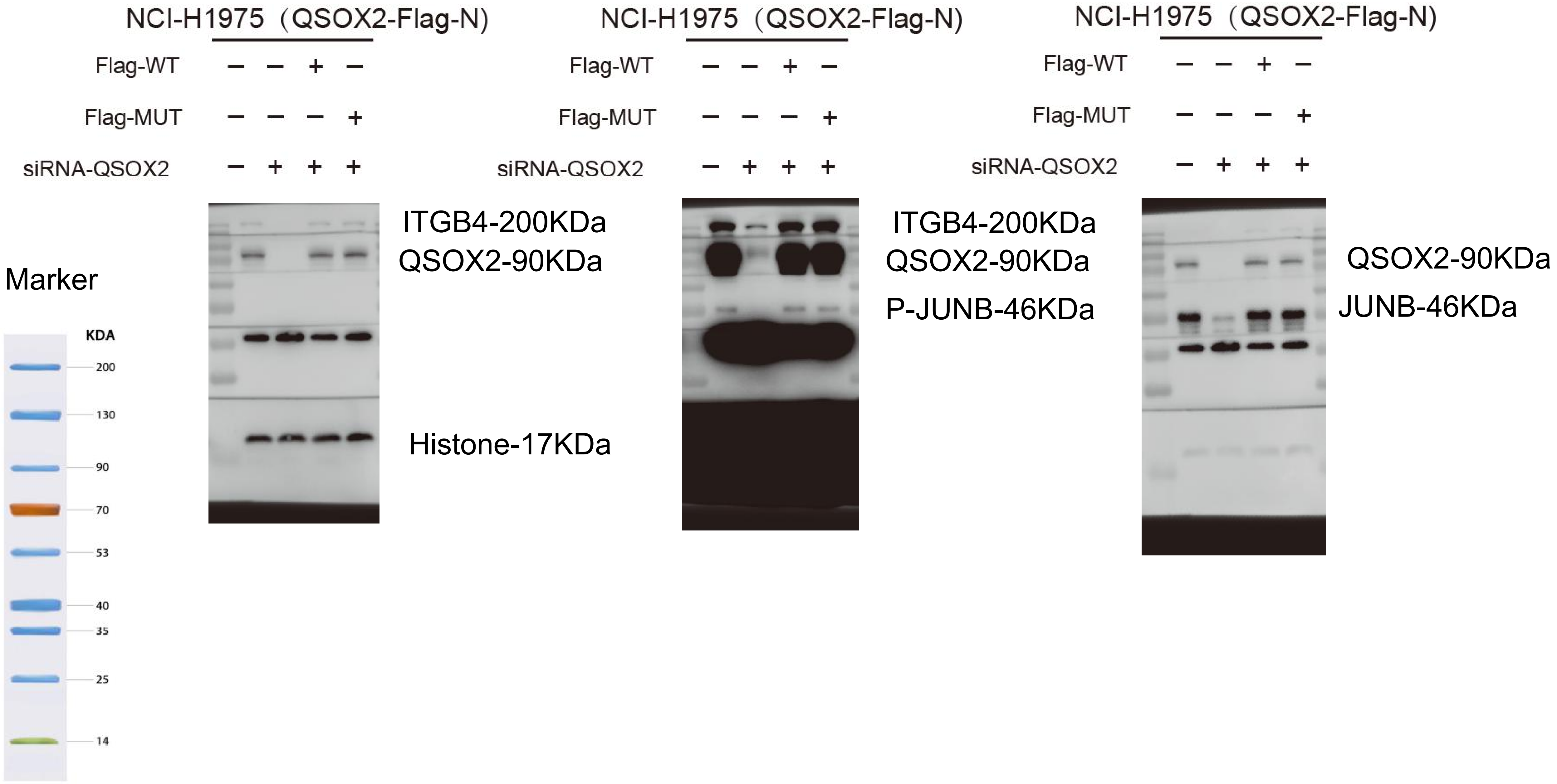
NCI-H1975（QSOX2-Flag-N)
Flag-WT
Flag-MUT
siRNA-QSOX2
ITGB4-200KDa
QSOX2-90KDa
Histone-17KDa
Marker
KDA
200
130
90
70
53
40
35
25
14
NCI-H1975（QSOX2-Flag-N)
Flag-WT
Flag-MUT
siRNA-QSOX2
ITGB4-200KDa
QSOX2-90KDa
P-JUNB-46KDa
NCI-H1975（QSOX2-Flag-N)
Flag-WT
Flag-MUT
siRNA-QSOX2
QSOX2-90KDa
JUNB-46KDa

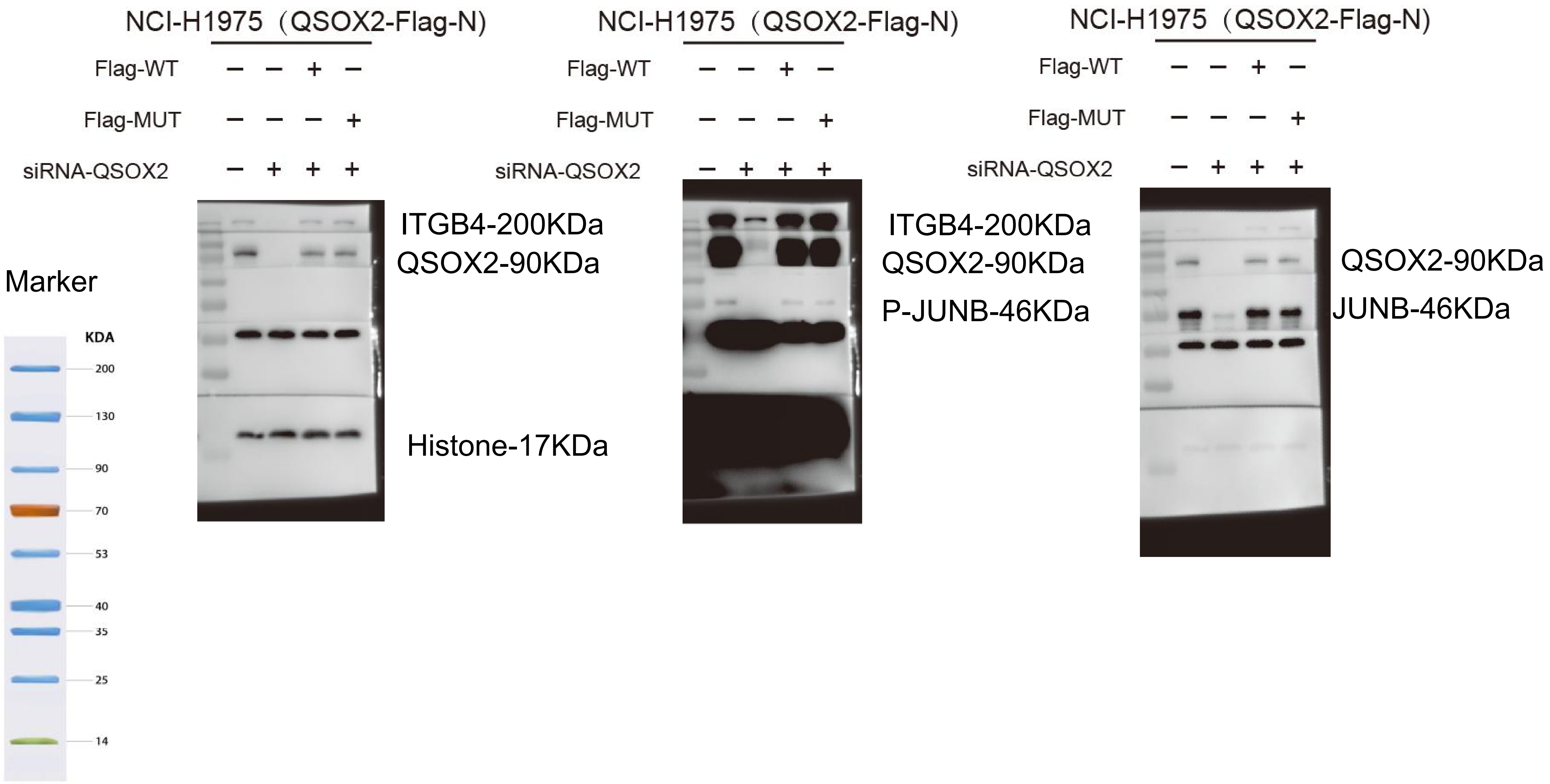
NCI-H1975（QSOX2-Flag-N)
Flag-WT − − + −
Flag-MUT − − − +
siRNA-QSOX2 − + + +
ITGB4-200KDa
QSOX2-90KDa
Histone-17KDa
NCI-H1975（QSOX2-Flag-N)
Flag-WT − − + −
Flag-MUT − − − +
siRNA-QSOX2 − + + +
ITGB4-200KDa
QSOX2-90KDa
P-JUNB-46KDa
NCI-H1975（QSOX2-Flag-N)
Flag-WT − − + −
Flag-MUT − − − +
siRNA-QSOX2 − + + +
QSOX2-90KDa
JUNB-46KDa
Marker
KDA
200
130
90
70
53
40
35
25
14

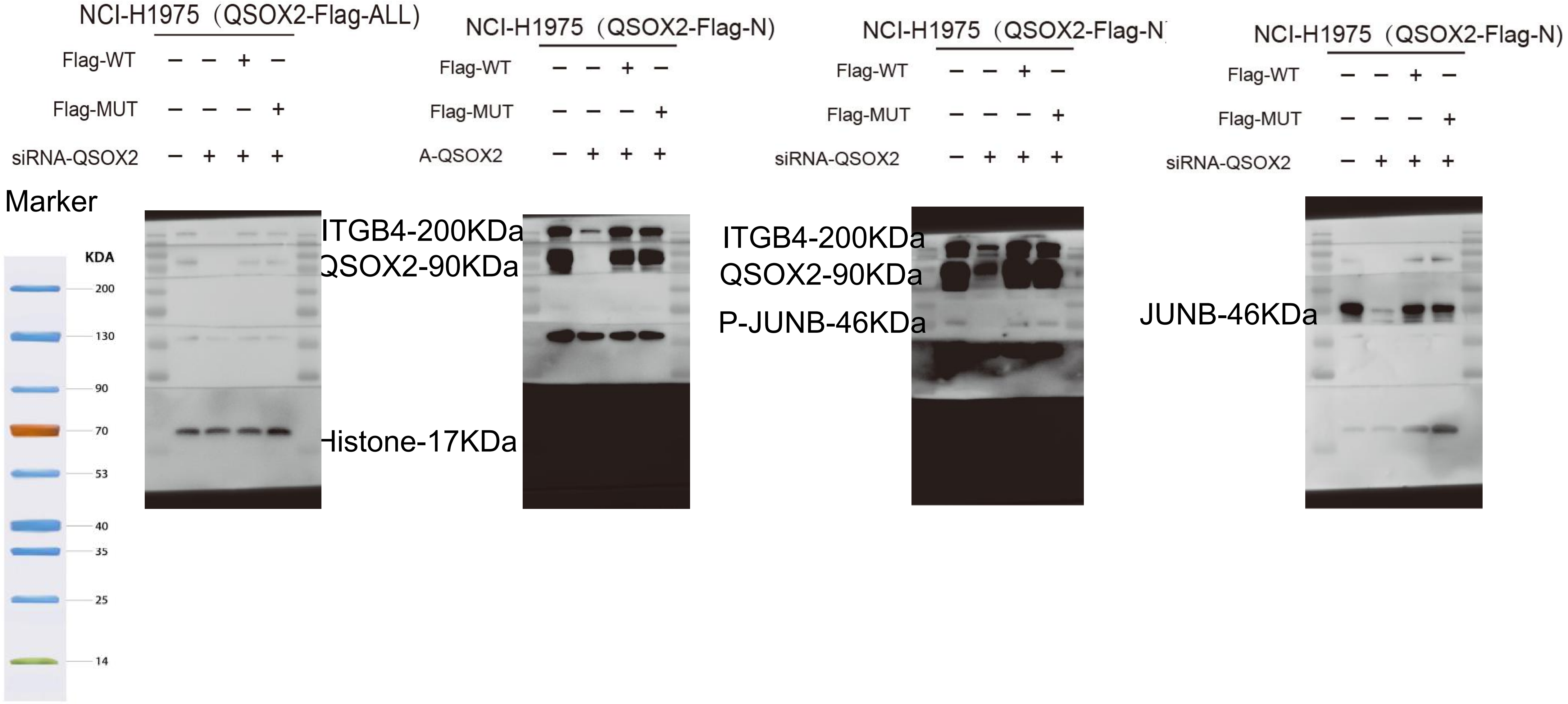
NCI-H1975（QSOX2-Flag-ALL)
Flag-WT − − + −
Flag-MUT − − − +
siRNA-QSOX2 − + + +
Marker
KDA
200
130
90
70
53
40
35
25
14
ITGB4-200KDa
QSOX2-90KDa
Histone-17KDa
NCI-H1975（QSOX2-Flag-N)
Flag-WT − − + −
Flag-MUT − − − +
A-QSOX2 − + + +
NCI-H1975（QSOX2-Flag-N
Flag-WT − − + −
Flag-MUT − − − +
siRNA-QSOX2 − + + +
ITGB4-200KDa
QSOX2-90KDa
P-JUNB-46KDa
NCI-H1975（QSOX2-Flag-N)
Flag-WT − − + −
Flag-MUT − − − +
siRNA-QSOX2 − + + +
JUNB-46KDa

figure7D-WB

Marker

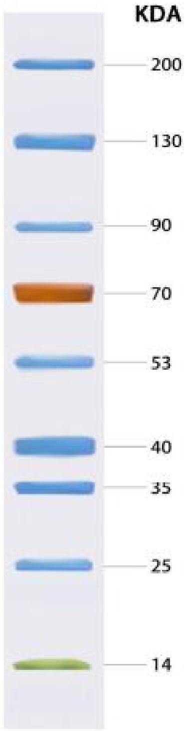

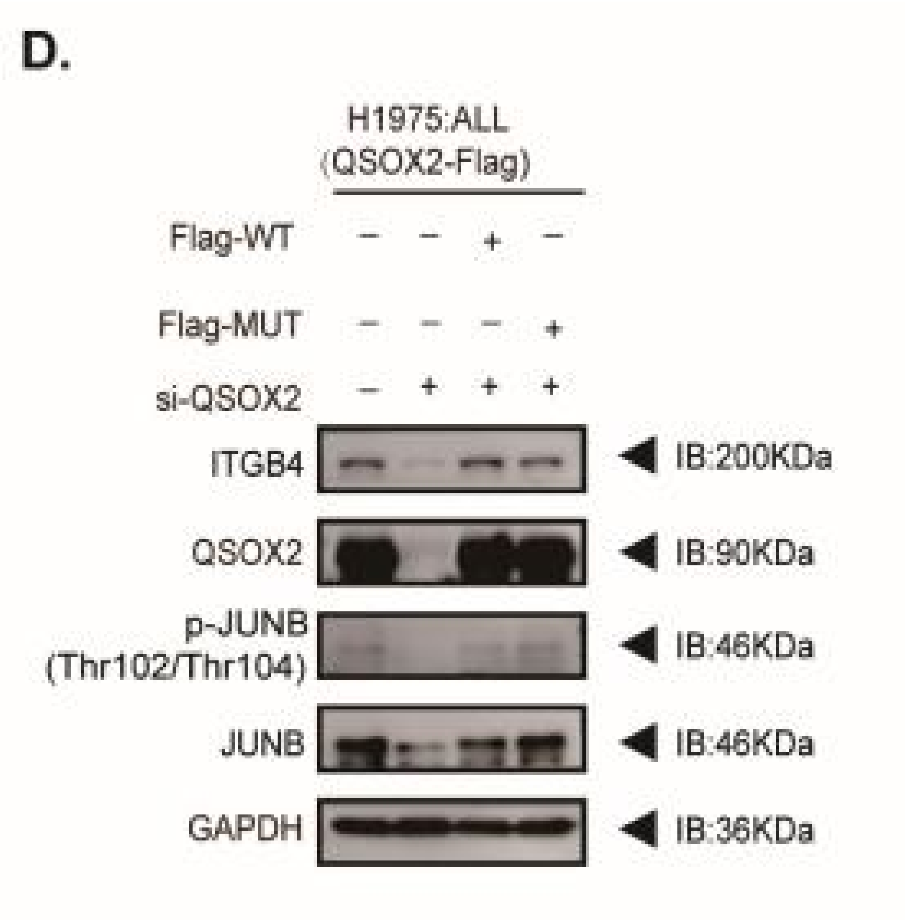

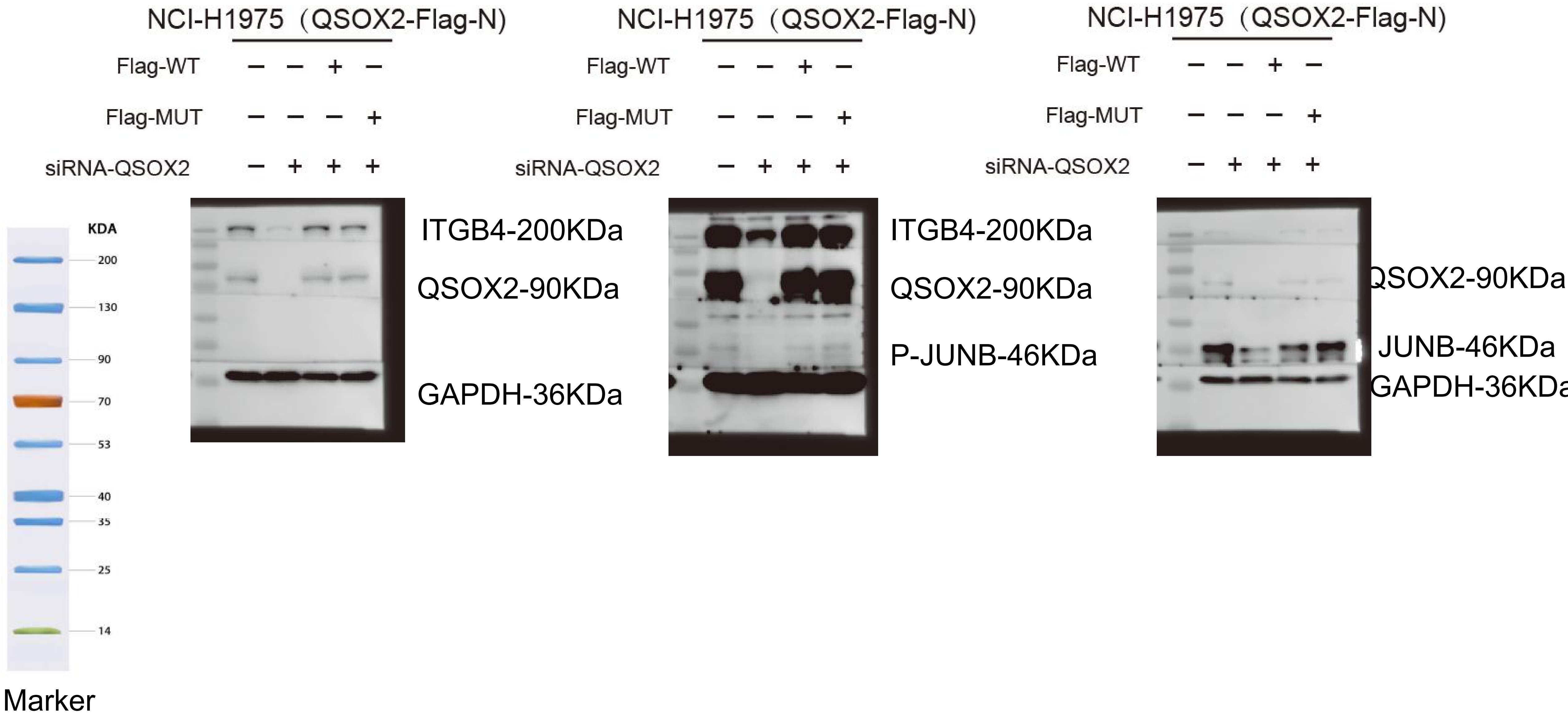
NCI-H1975（QSOX2-Flag-N)
Flag-WT − − + −
Flag-MUT − − − +
siRNA-QSOX2 − + + +
ITGB4-200KDa
QSOX2-90KDa
GAPDH-36KDa
NCI-H1975（QSOX2-Flag-N)
Flag-WT − − + −
Flag-MUT − − − +
siRNA-QSOX2 − + + +
ITGB4-200KDa
QSOX2-90KDa
P-JUNB-46KDa
NCI-H1975（QSOX2-Flag-N)
Flag-WT − − + −
Flag-MUT − − − +
siRNA-QSOX2 − + + +
QSOX2-90KDa
JUNB-46KDa
GAPDH-36KDa
KDA
200
130
90
70
53
40
35
25
14
Marker

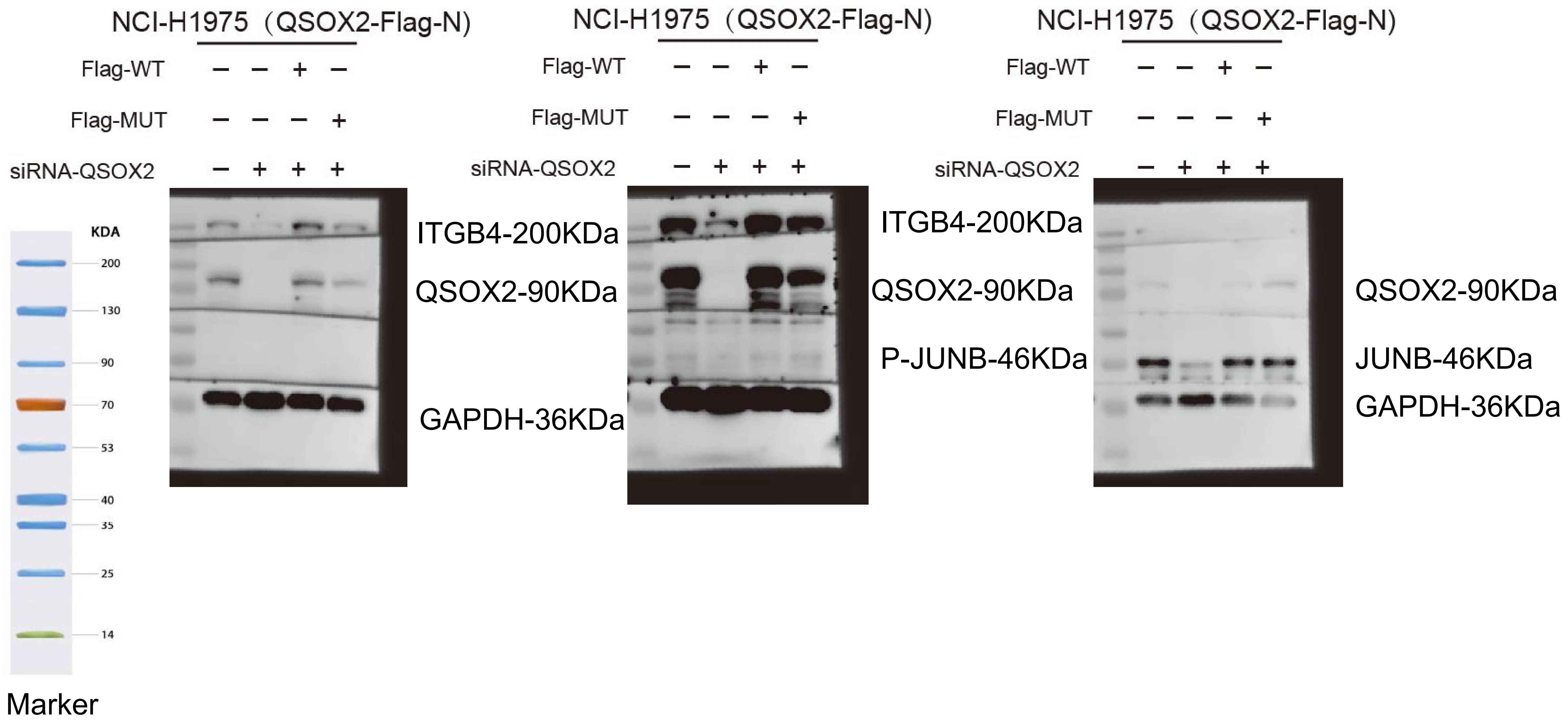
NCI-H1975（QSOX2-Flag-N)
Flag-WT
Flag-MUT
siRNA-QSOX2
KDA
200
130
90
70
53
40
35
25
14
Marker
ITGB4-200KDa
QSOX2-90KDa
GAPDH-36KDa
NCI-H1975（QSOX2-Flag-N)
Flag-WT
Flag-MUT
siRNA-QSOX2
ITGB4-200KDa
QSOX2-90KDa
P-JUNB-46KDa
NCI-H1975（QSOX2-Flag-N)
Flag-WT
Flag-MUT
siRNA-QSOX2
QSOX2-90KDa
JUNB-46KDa
GAPDH-36KDa

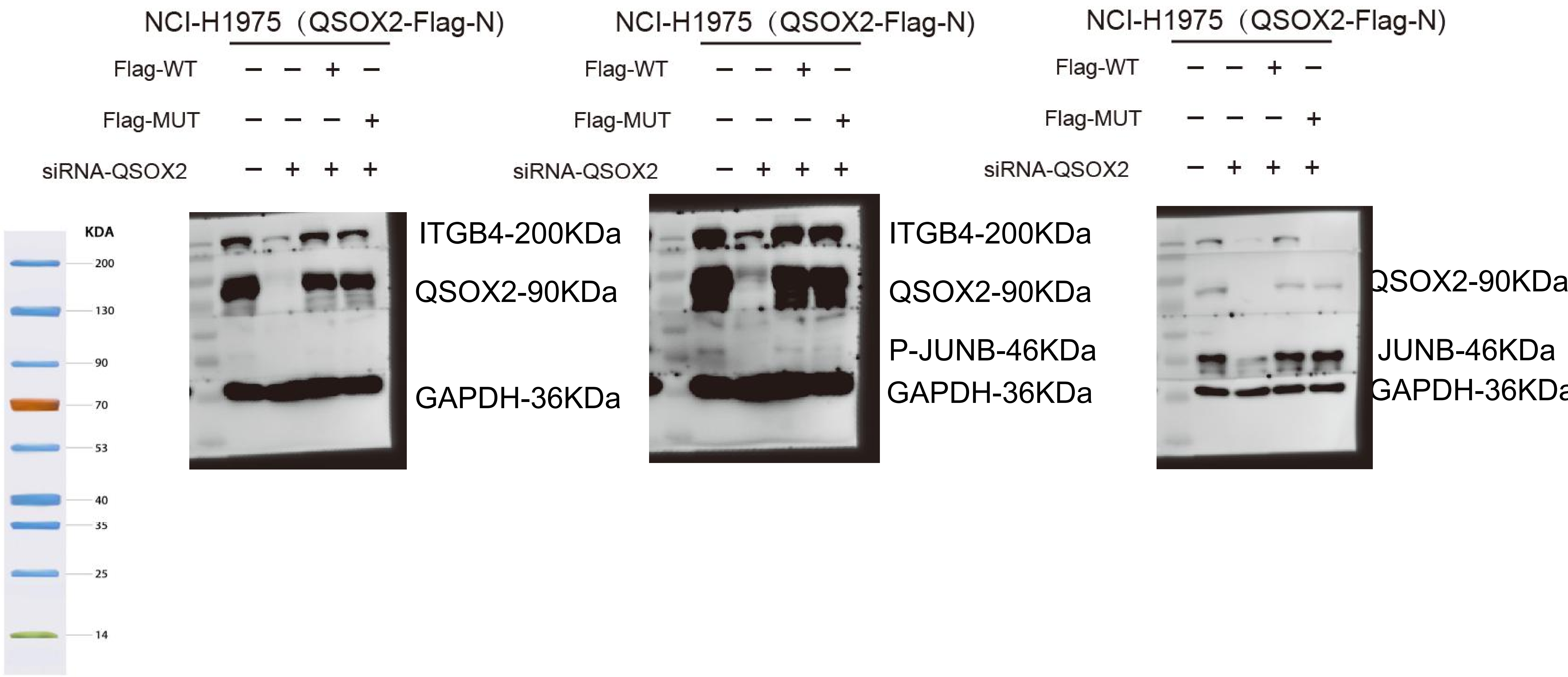

NCI-H1975（QSOX2-Flag-N)
Flag-WT − − + −
Flag-MUT − − − +
siRNA-QSOX2 − + + +
ITGB4-200KDa
QSOX2-90KDa
GAPDH-36KDa
NCI-H1975（QSOX2-Flag-N)
Flag-WT − − + −
Flag-MUT − − − +
siRNA-QSOX2 − + + +
ITGB4-200KDa
QSOX2-90KDa
P-JUNB-46KDa
GAPDH-36KDa
NCI-H1975（QSOX2-Flag-N)
Flag-WT − − + −
Flag-MUT − − − +
siRNA-QSOX2 − + + +
QSOX2-90KDa
JUNB-46KDa
GAPDH-36KDa
KDA
200
130
90
70
53
40
35
25
14

Marker

figure7E-WB

Marker

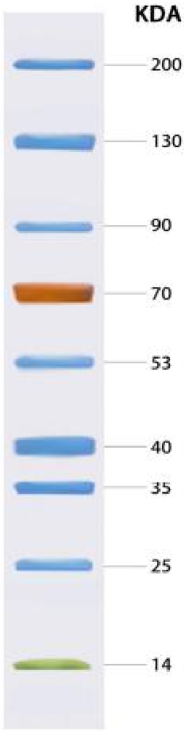

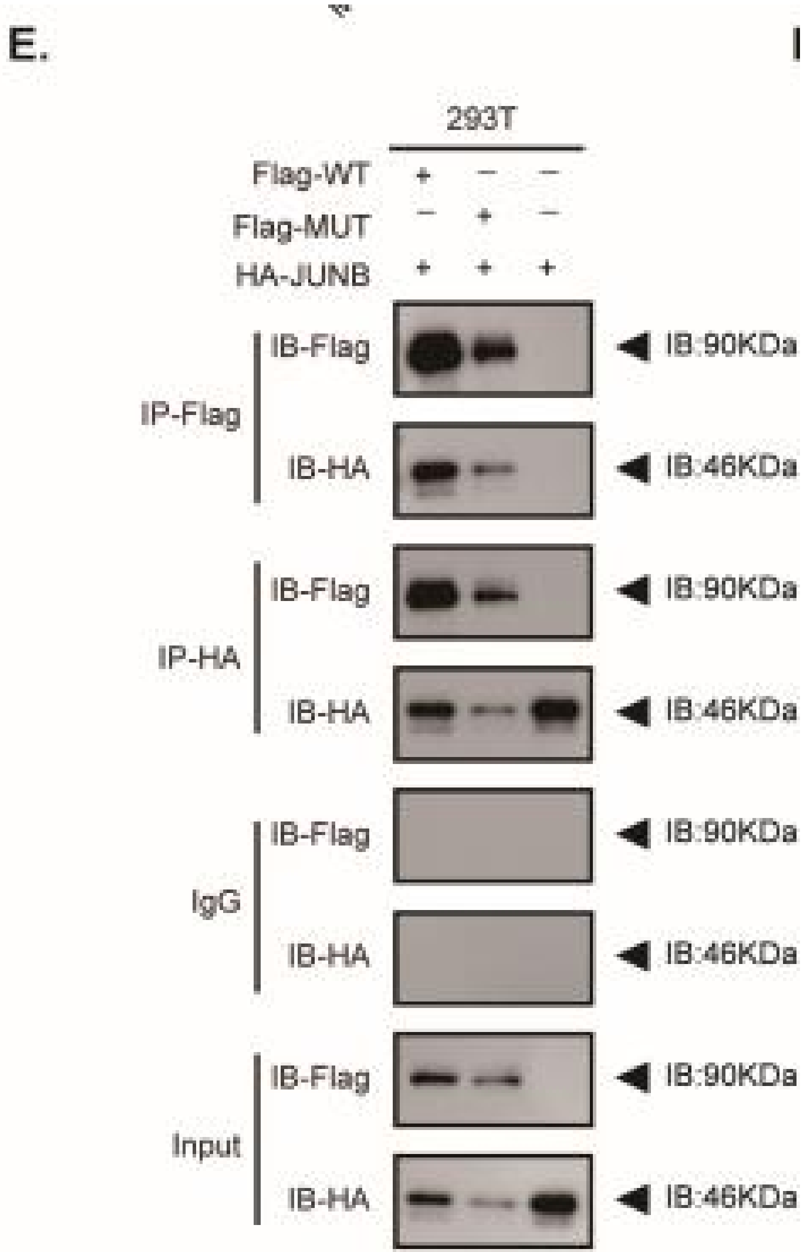

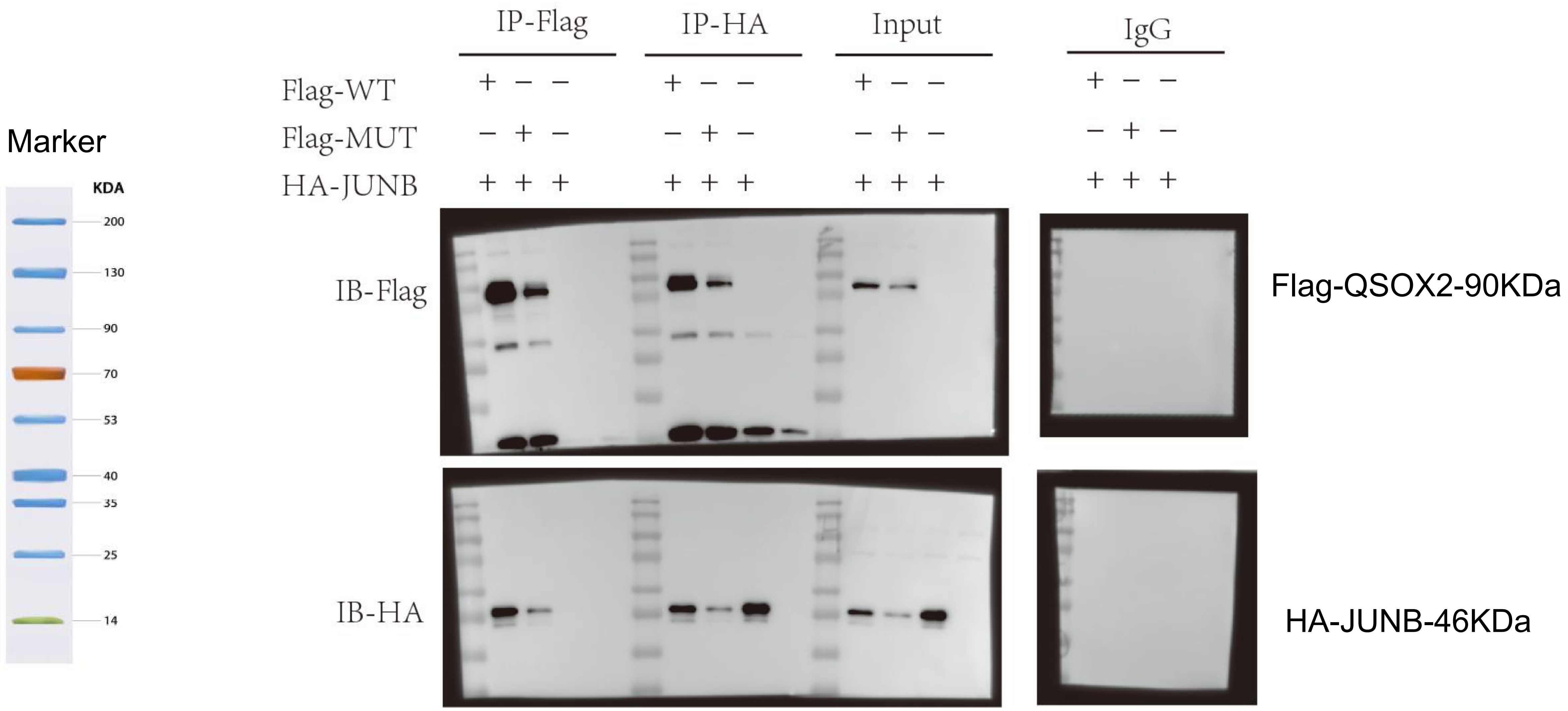

Marker
KDA
200
130
90
70
53
40
35
25
14
IP-Flag
IP-HA
Input
IgG
Flag-WT
Flag-MUT
HA-JUNB
IB-Flag
IB-HA
Flag-QSOX2-90KDa
HA-JUNB-46KDa

Marker

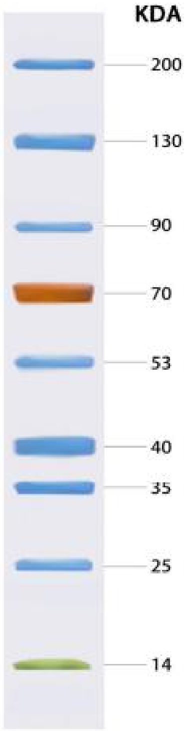
KDA
200
130
90
70
53
40
35
25
14

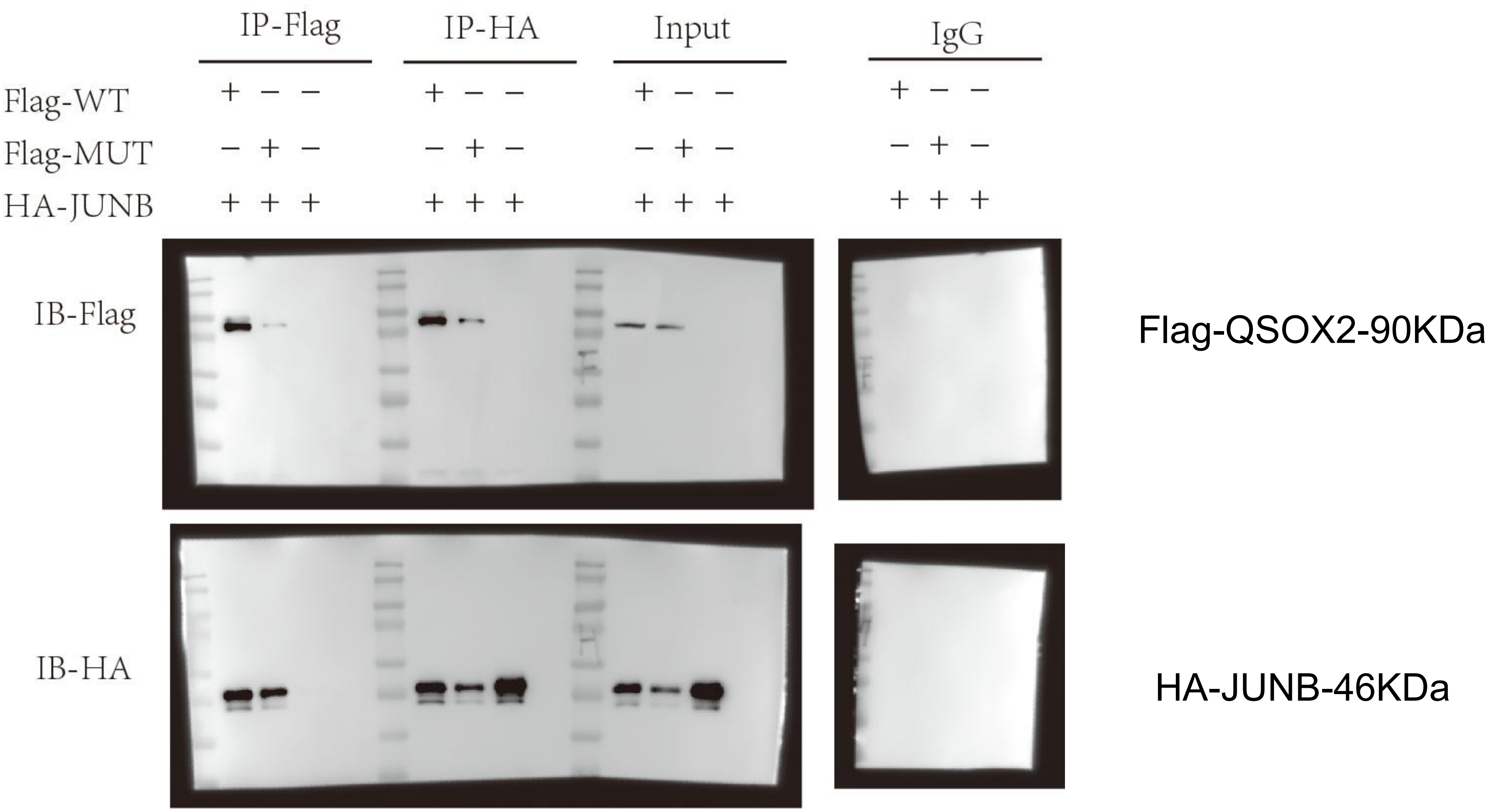
IP-Flag
IP-HA
Input
IgG
Flag-WT
Flag-MUT
HA-JUNB
IB-Flag
IB-HA
Flag-QSOX2-90KDa
HA-JUNB-46KDa

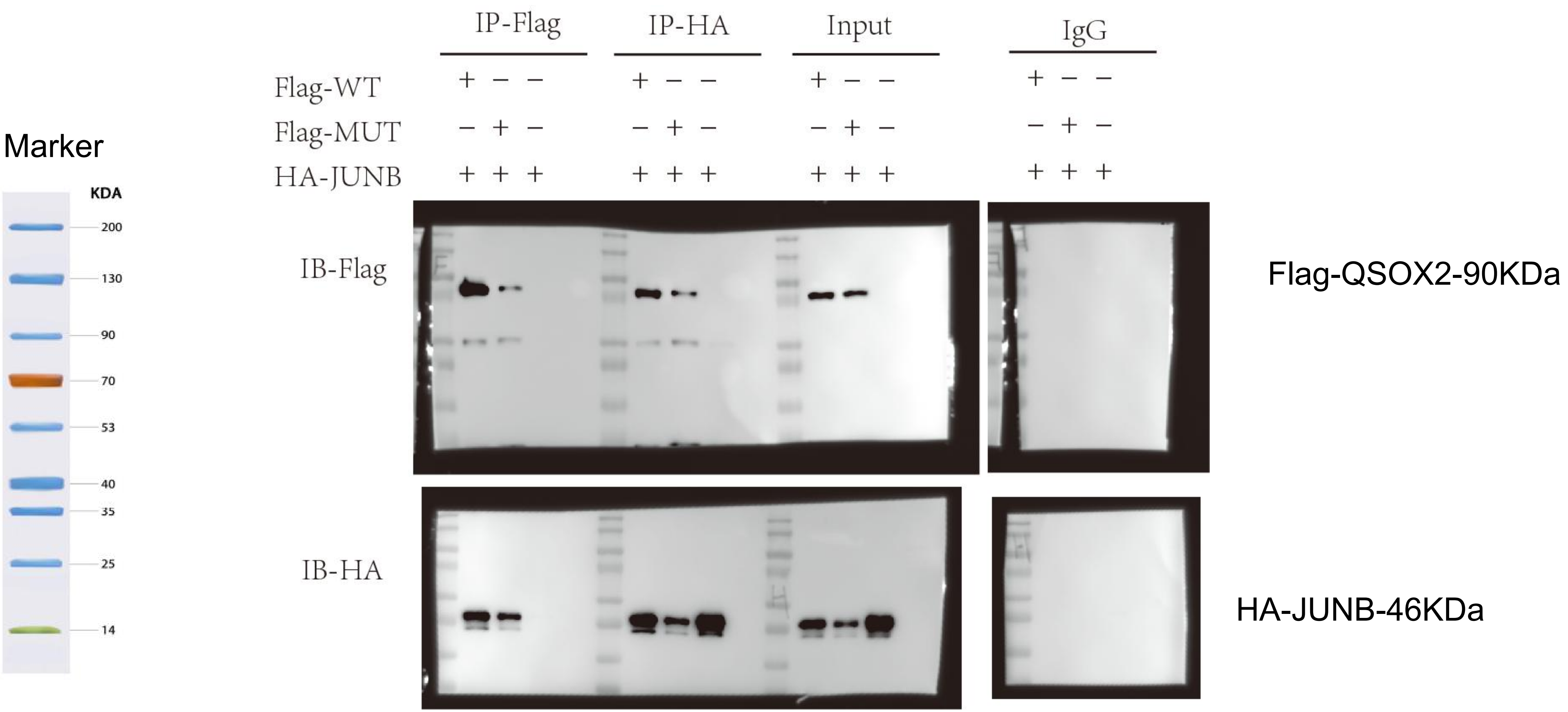
Marker
KDA
200
130
90
70
53
40
35
25
14
IP-Flag
IP-HA
Input
IgG
Flag-WT + − − + − − + − − + − −
Flag-MUT − + − − + − − + − − + −
HA-JUNB + + + + + + + + + + + +
IB-Flag
IB-HA
Flag-QSOX2-90KDa
HA-JUNB-46KDa

figure8C-WB

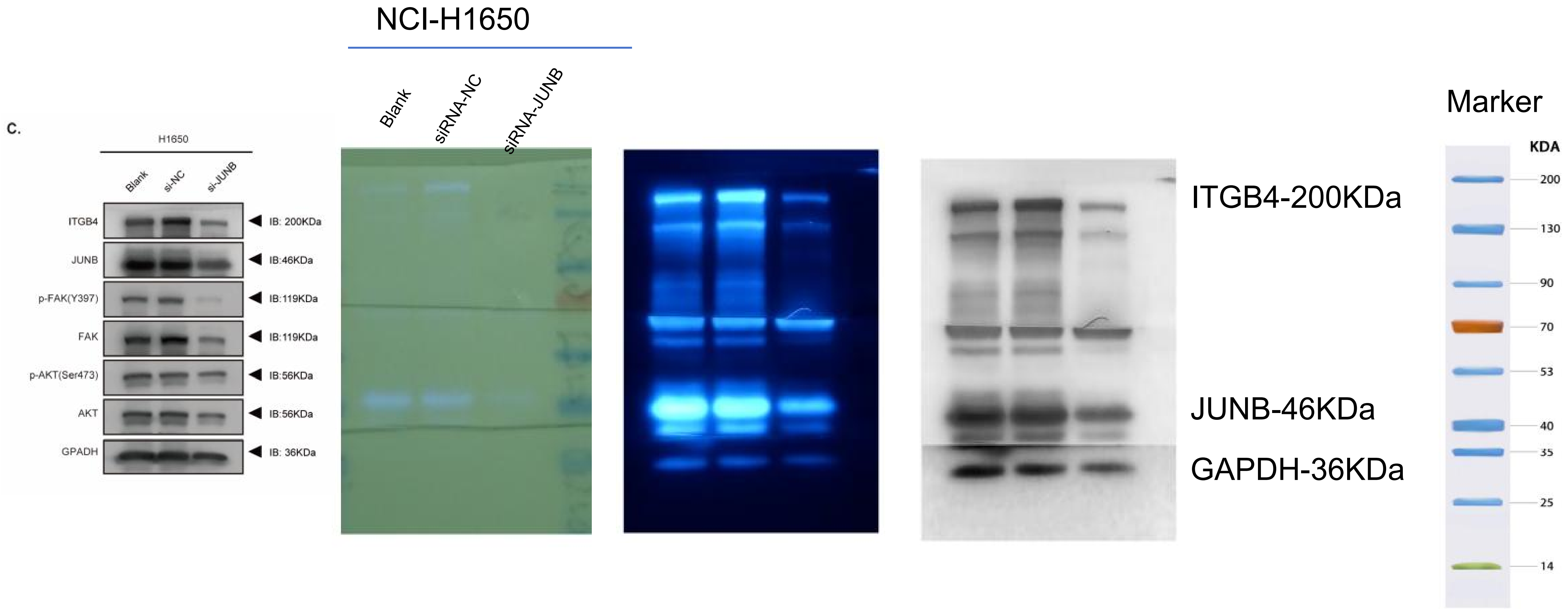
C.
H1650
Blank
si-NC
si-JUNB
ITGB4
JUNB
p-FAK(Y397)
FAK
p-AKT(Ser473)
AKT
GPADH
IB: 200KDa
IB:46KDa
IB:119KDa
IB:119KDa
IB:56KDa
IB:56KDa
IB: 36KDa
NCI-H1650
Blank
siRNA-NC
siRNA-JUNB
ITGB4-200KDa
JUNB-46KDa
GAPDH-36KDa
Marker
KDA
200
130
90
70
53
40
35
25
14

figure8C-WB

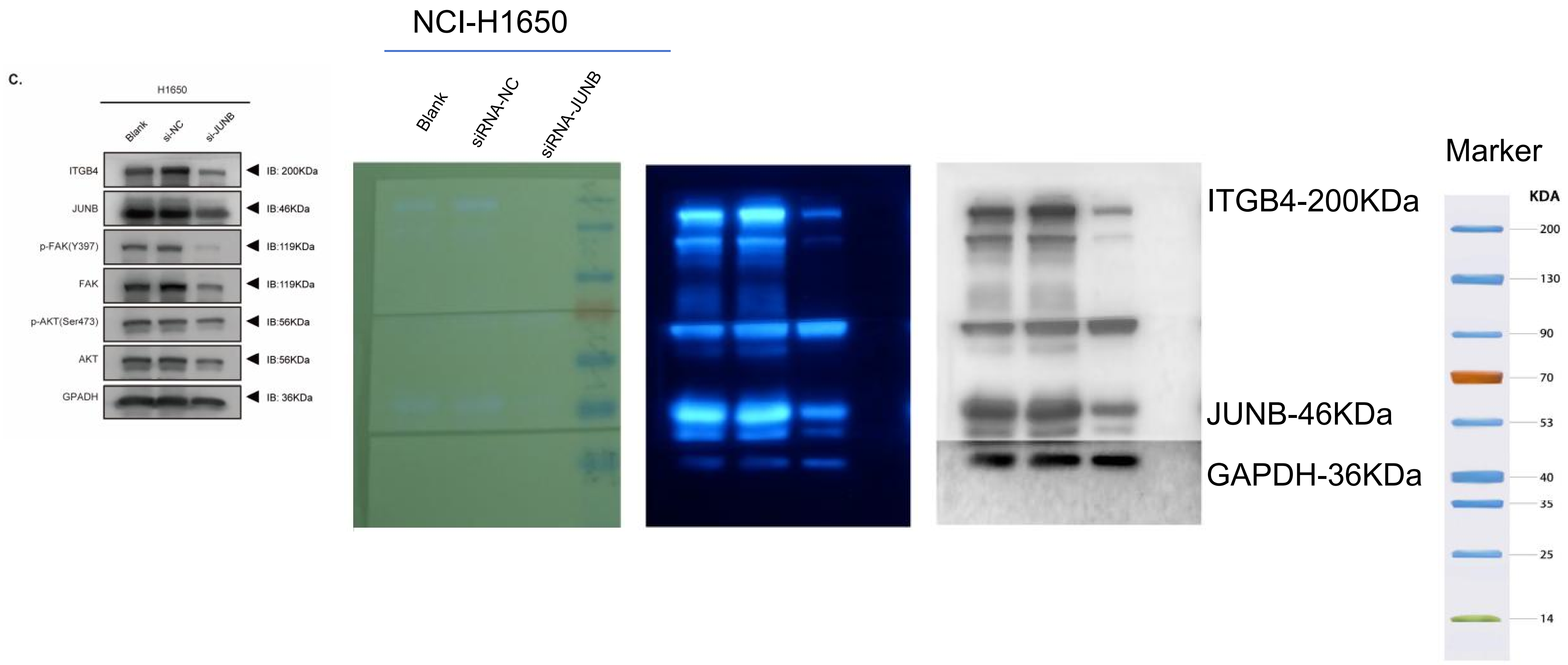

C.
H1650
Blank
si-NC
si-JUNB
ITGB4
JUNB
p-FAK(Y397)
FAK
p-AKT(Ser473)
AKT
GPADH
IB: 200KDa
IB:46KDa
IB:119KDa
IB:119KDa
IB:56KDa
IB:56KDa
IB: 36KDa
NCI-H1650
Blank
siRNA-NC
siRNA-JUNB
ITGB4-200KDa
JUNB-46KDa
GAPDH-36KDa
Marker
KDA
200
130
90
70
53
40
35
25
14

figure8C-WB

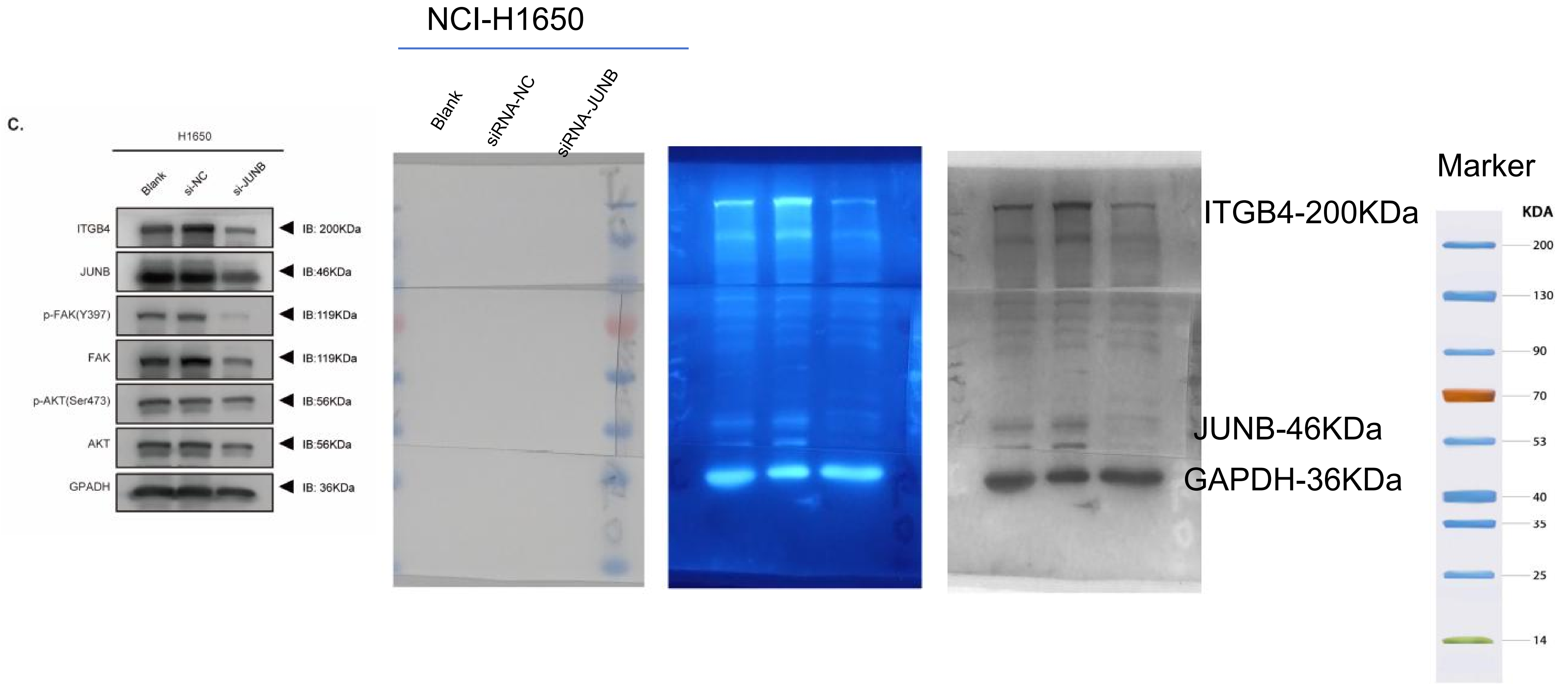
C.
H1650
Blank
si-NC
si-JUNB
ITGB4
IB: 200KDa
JUNB
IB:46KDa
p-FAK(Y397)
IB:119KDa
FAK
IB:119KDa
p-AKT(Ser473)
IB:56KDa
AKT
IB:56KDa
GPADH
IB: 36KDa
NCI-H1650
Blank
siRNA-NC
siRNA-JUNB
ITGB4-200KDa
JUNB-46KDa
GAPDH-36KDa
Marker
KDA
200
130
90
70
53
40
35
25
14

figure8C-WB

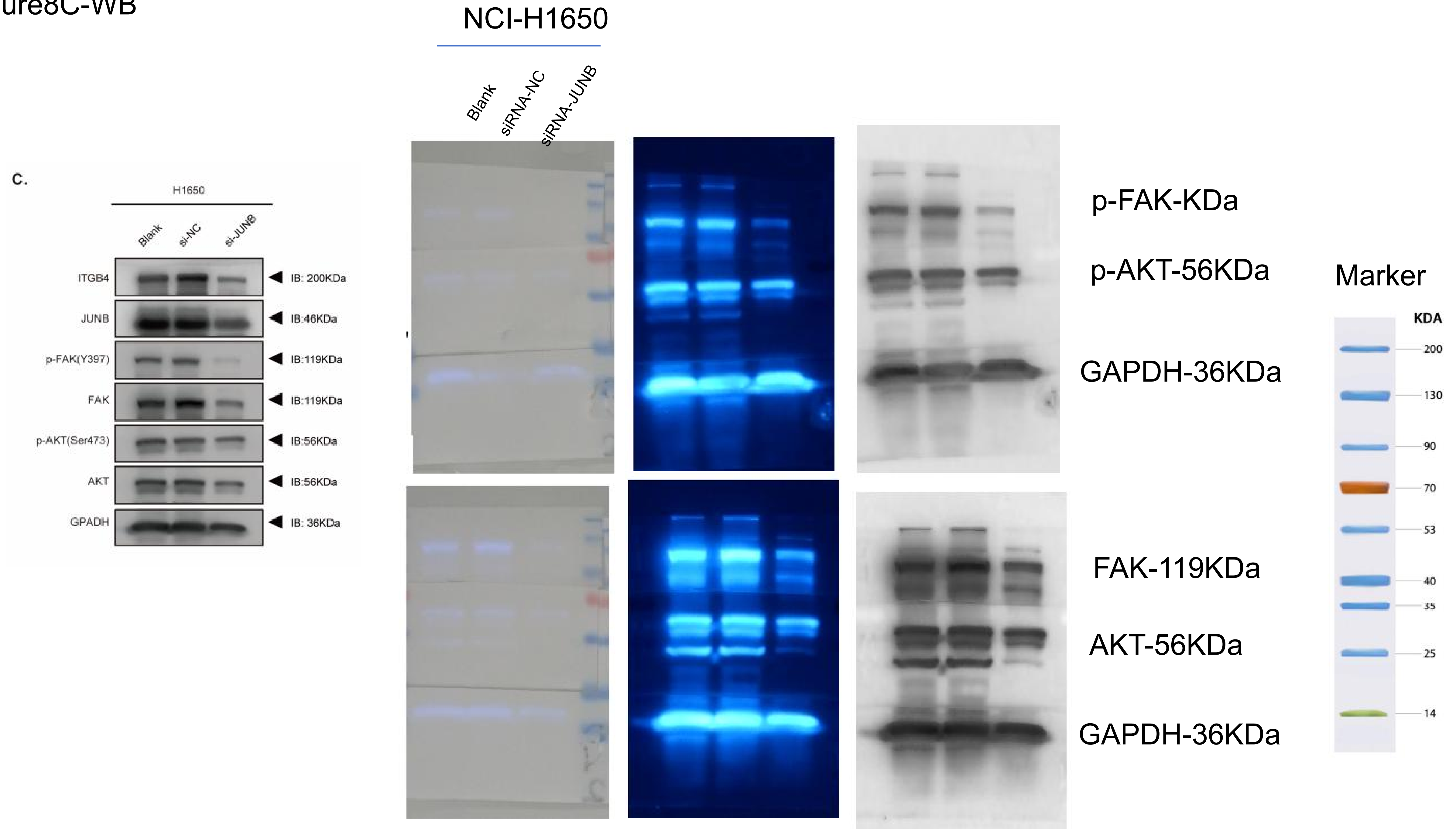
NCI-H1650
Blank
siRNA-NC
siRNA-JUNB
C.
H1650
Blank
si-NC
si-JUNB
ITGB4
JUNB
p-FAK(Y397)
FAK
p-AKT(Ser473)
AKT
GPADH
IB: 200KDa
IB:46KDa
IB:119KDa
IB:119KDa
IB:56KDa
IB:56KDa
IB: 36KDa
p-FAK-KDa
p-AKT-56KDa
GAPDH-36KDa
FAK-119KDa
AKT-56KDa
GAPDH-36KDa
Marker
KDA
200
130
90
70
53
40
35
25
14

figure8C-WB

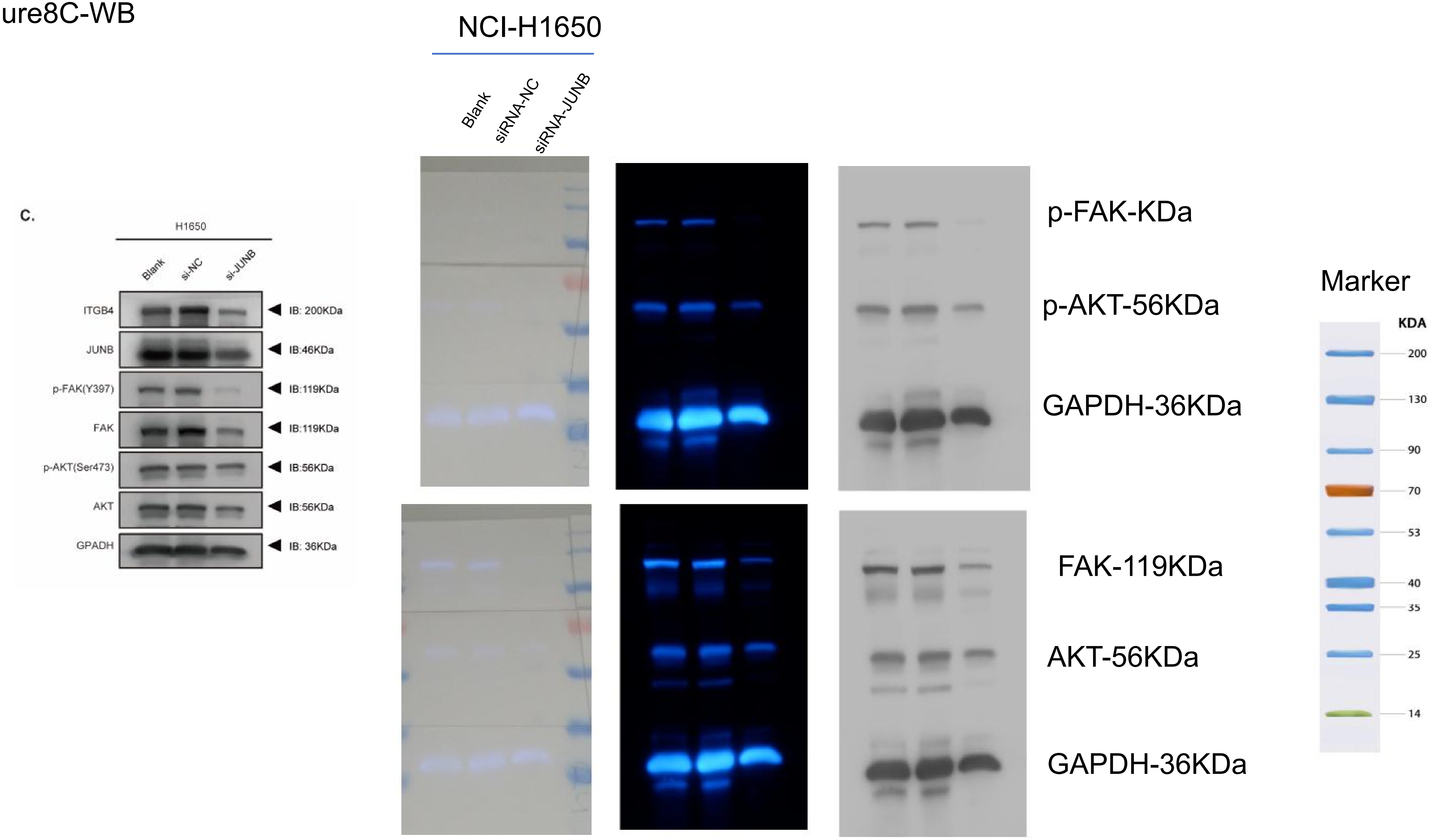
C.
H1650
Blank
si-NC
si-JUNB
ITGB4
IB: 200KDa
JUNB
IB:46KDa
p-FAK(Y397)
IB:119KDa
FAK
IB:119KDa
p-AKT(Ser473)
IB:56KDa
AKT
IB:56KDa
GPADH
IB: 36KDa
NCI-H1650
Blank
siRNA-NC
siRNA-JUNB
p-FAK-KDa
p-AKT-56KDa
GAPDH-36KDa
FAK-119KDa
AKT-56KDa
GAPDH-36KDa
Marker
KDA
200
130
90
70
53
40
35
25
14

figure8C-WB

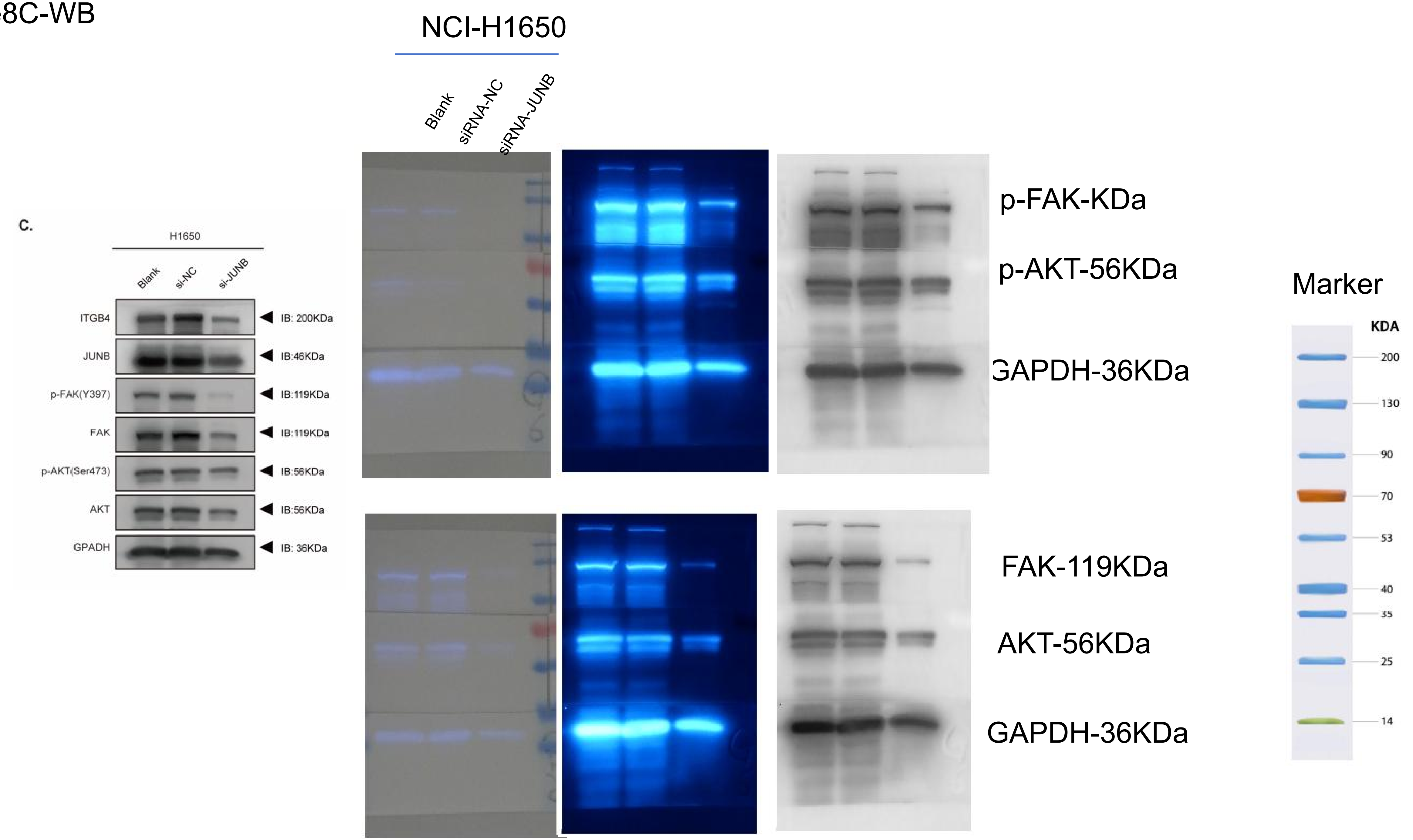
NCI-H1650
Blank
siRNA-NC
siRNA-JUNB
p-FAK-KDa
p-AKT-56KDa
GAPDH-36KDa
FAK-119KDa
AKT-56KDa
GAPDH-36KDa
Marker
KDA
200
130
90
70
53
40
35
25
14
C.
H1650
Blank
si-NC
si-JUNB
ITGB4
IB: 200KDa
JUNB
IB:46KDa
p-FAK(Y397)
IB:119KDa
FAK
IB:119KDa
p-AKT(Ser473)
IB:56KDa
AKT
IB:56KDa
GPADH
IB: 36KDa

figure8D-WB

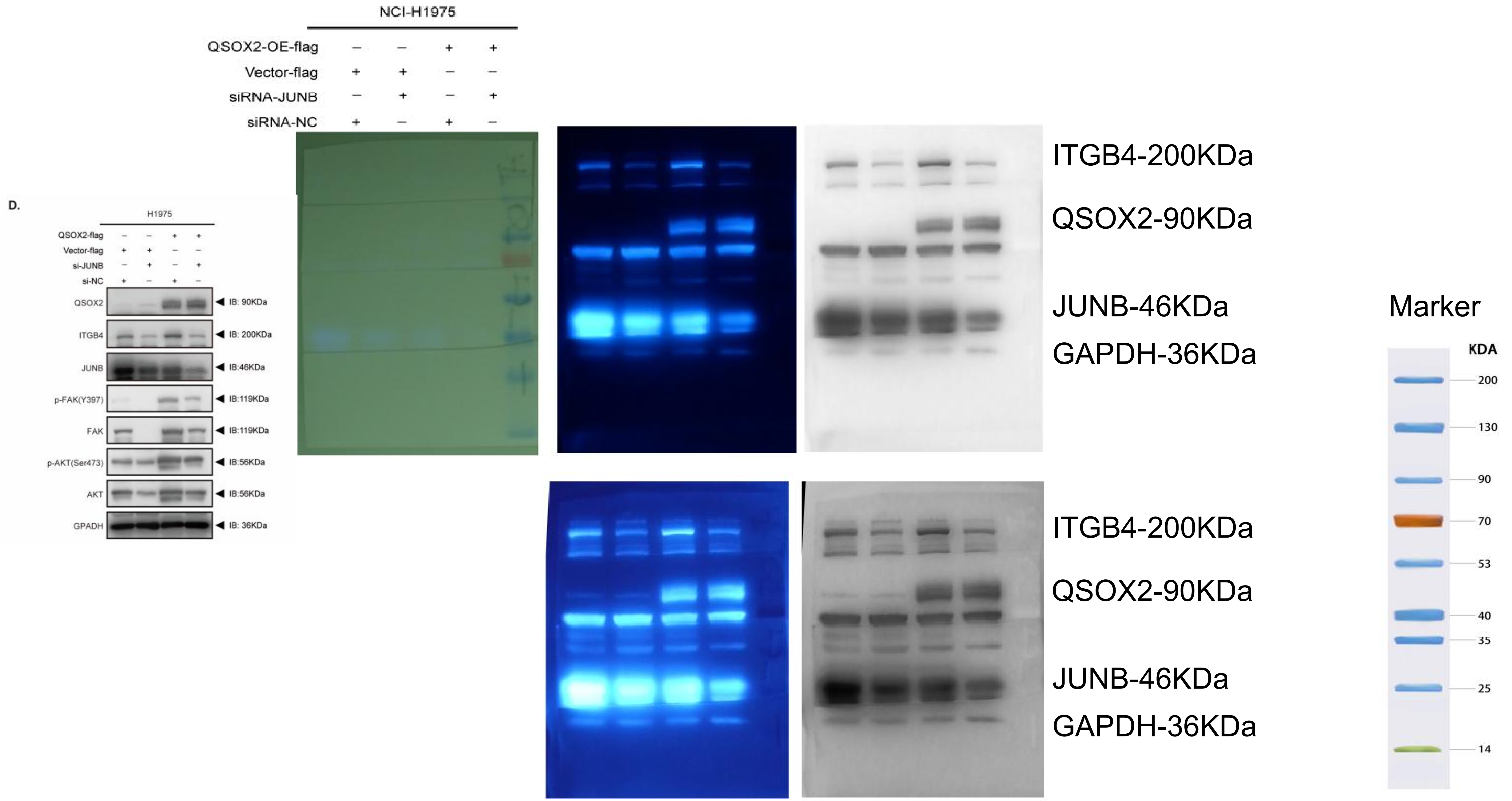
NCI-H1975
QSOX2-OE-flag – – + +
Vector-flag + + – –
siRNA-JUNB – + – +
siRNA-NC + – + –
D.
H1975
QSOX2-flag – – + +
Vector-flag + + – –
si-JUNB – + – +
si-NC + – + –
QSOX2
IB: 90KDa
ITGB4
IB: 200KDa
JUNB
IB:46KDa
p-FAK(Y397)
IB:119KDa
FAK
IB:119KDa
p-AKT(Ser473)
IB:56KDa
AKT
IB:56KDa
GPADH
IB: 36KDa
ITGB4-200KDa
QSOX2-90KDa
JUNB-46KDa
GAPDH-36KDa
ITGB4-200KDa
QSOX2-90KDa
JUNB-46KDa
GAPDH-36KDa
Marker
KDA
200
130
90
70
53
40
35
25
14

figure8D-WB

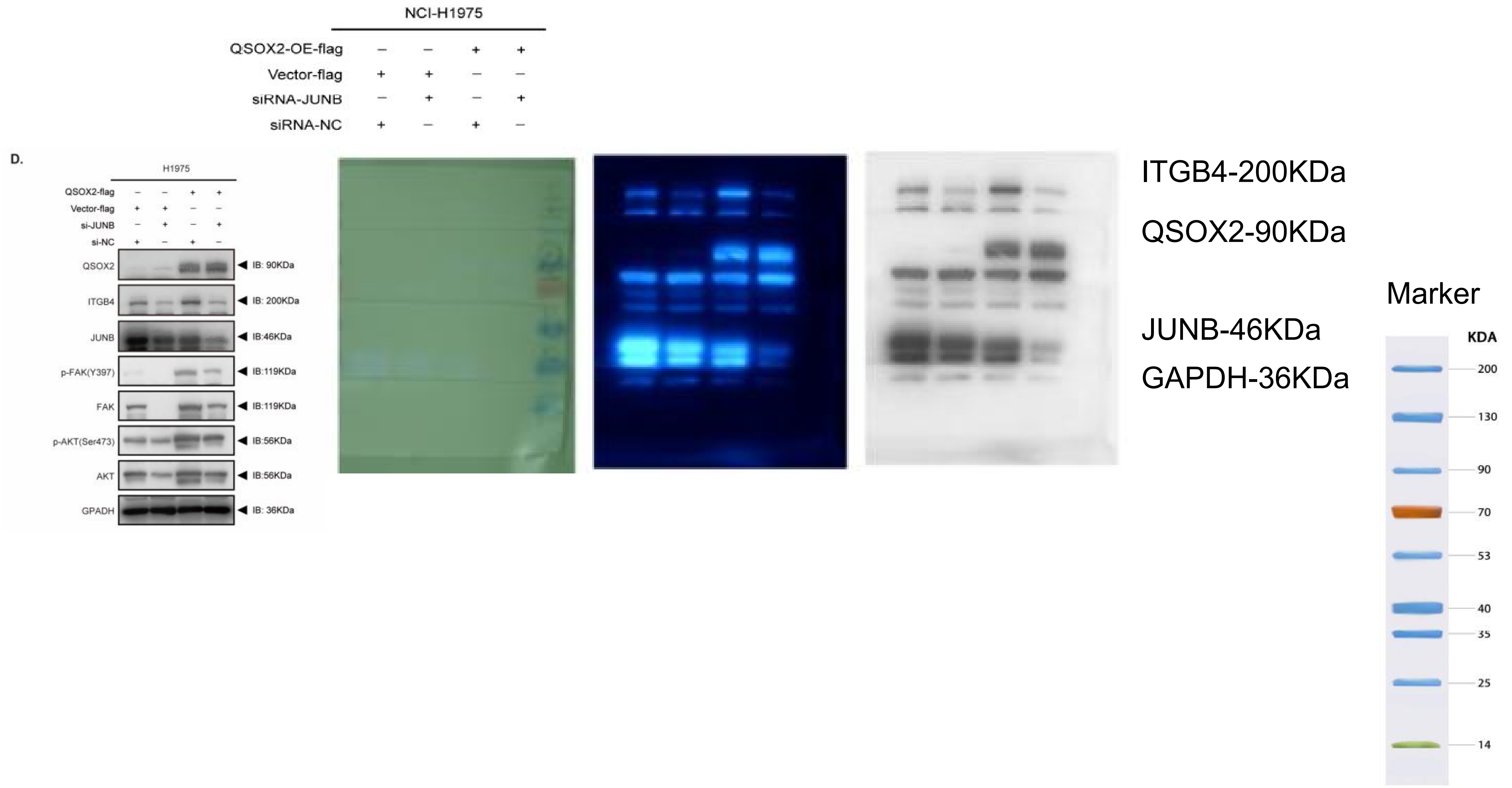
NCI-H1975
QSOX2-OE-flag − − + +
Vector-flag + + − −
siRNA-JUNB − + − +
siRNA-NC + − + −
D.
H1975
QSOX2-flag − − + +
Vector-flag + + − −
si-JUNB − + − +
si-NC + − + −
QSOX2
IB: 90KDa
ITGB4
IB: 200KDa
JUNB
IB:46KDa
p-FAK(Y397)
IB:119KDa
FAK
IB:119KDa
p-AKT(Ser473)
IB:56KDa
AKT
IB:56KDa
GPADH
IB: 36KDa
ITGB4-200KDa
QSOX2-90KDa
JUNB-46KDa
GAPDH-36KDa
Marker
KDA
200
130
90
70
53
40
35
25
14

figure8D-WB

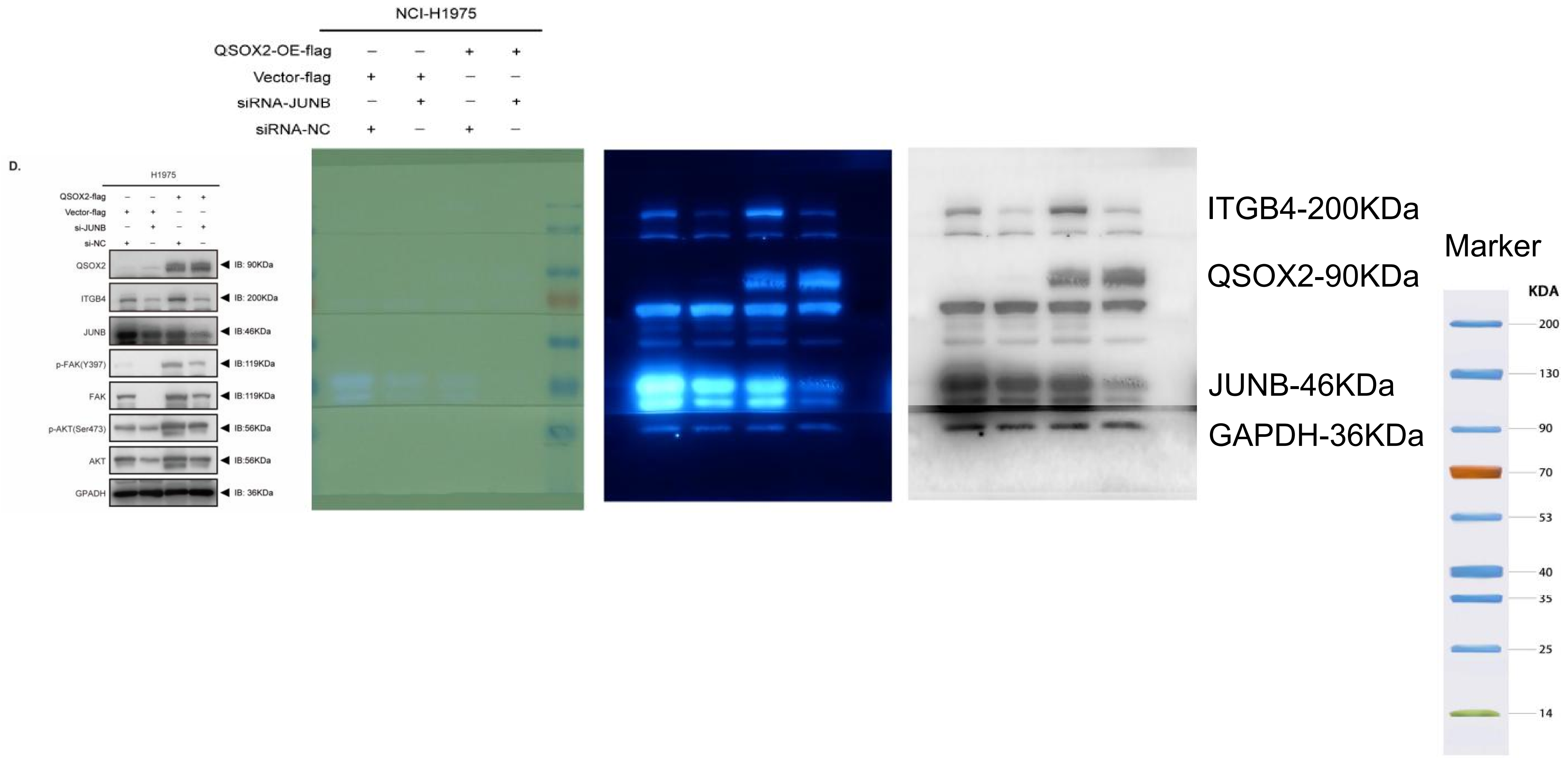
NCI-H1975
QSOX2-OE-flag
Vector-flag
siRNA-JUNB
siRNA-NC
D.
H1975
QSOX2-flag
Vector-flag
si-JUNB
si-NC
QSOX2
IB: 90KDa
ITGB4
IB: 200KDa
JUNB
IB:46KDa
p-FAK(Y397)
IB:119KDa
FAK
IB:119KDa
p-AKT(Ser473)
IB:56KDa
AKT
IB:56KDa
GPADH
IB: 36KDa
ITGB4-200KDa
QSOX2-90KDa
JUNB-46KDa
GAPDH-36KDa
Marker
KDA
200
130
90
70
53
40
35
25
14

figure8D-WB

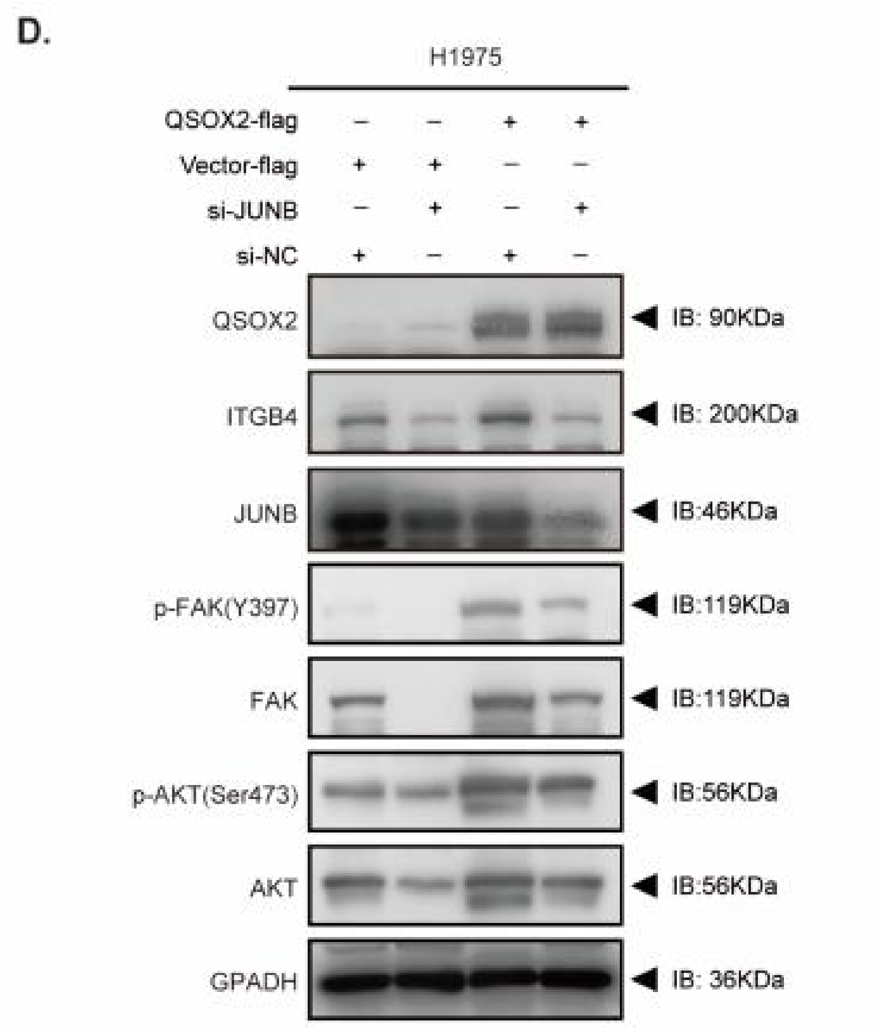

Marker

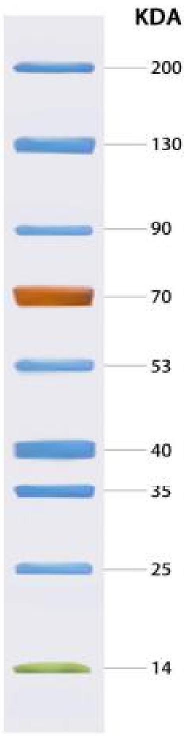

figure8D-WB

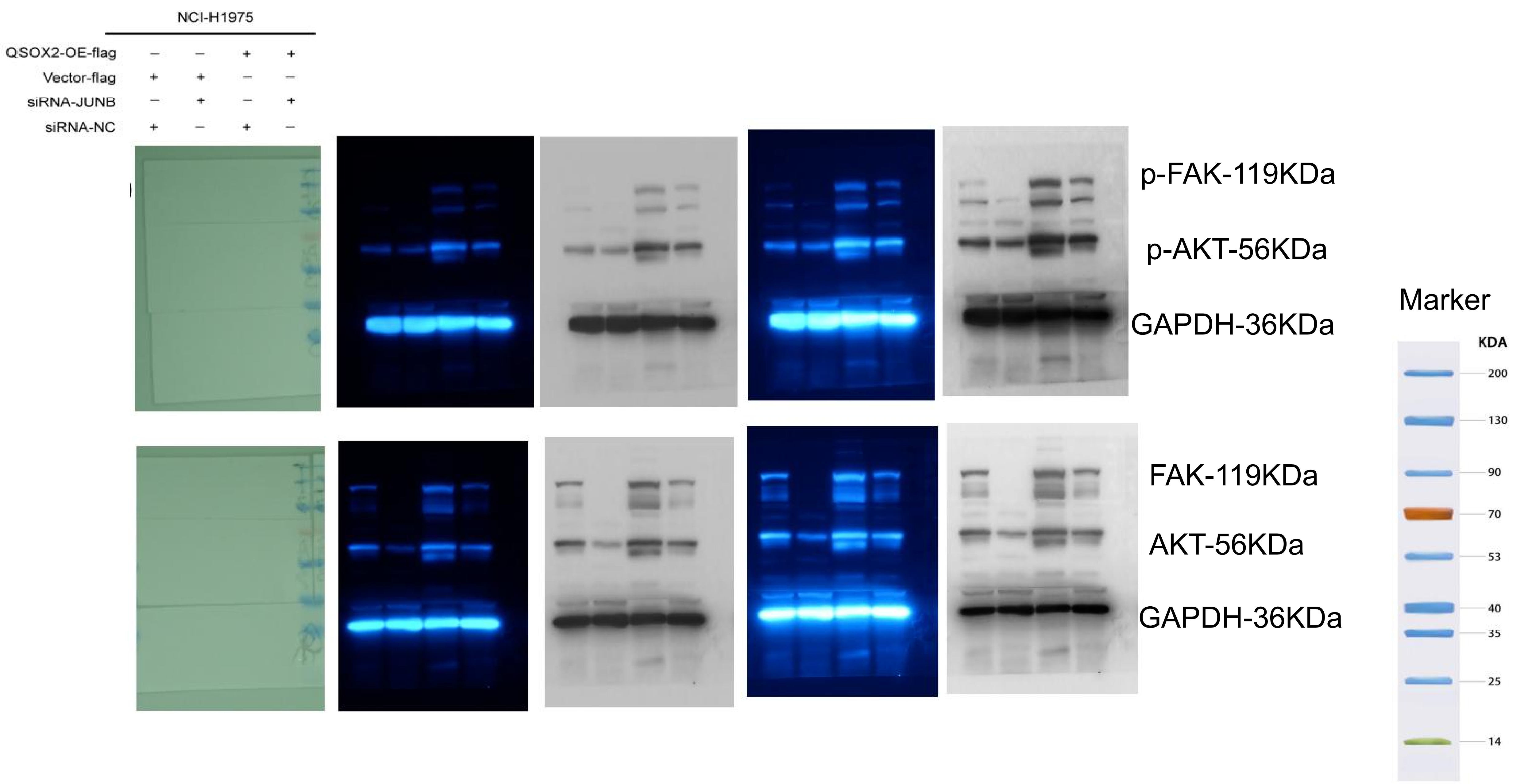
NCI-H1975
QSOX2-OE-flag – – + +
Vector-flag + + – –
siRNA-JUNB – + – +
siRNA-NC + – + –
p-FAK-119KDa
p-AKT-56KDa
GAPDH-36KDa
FAK-119KDa
AKT-56KDa
GAPDH-36KDa
Marker
KDA
200
130
90
70
53
40
35
25
14

figure8D-WB

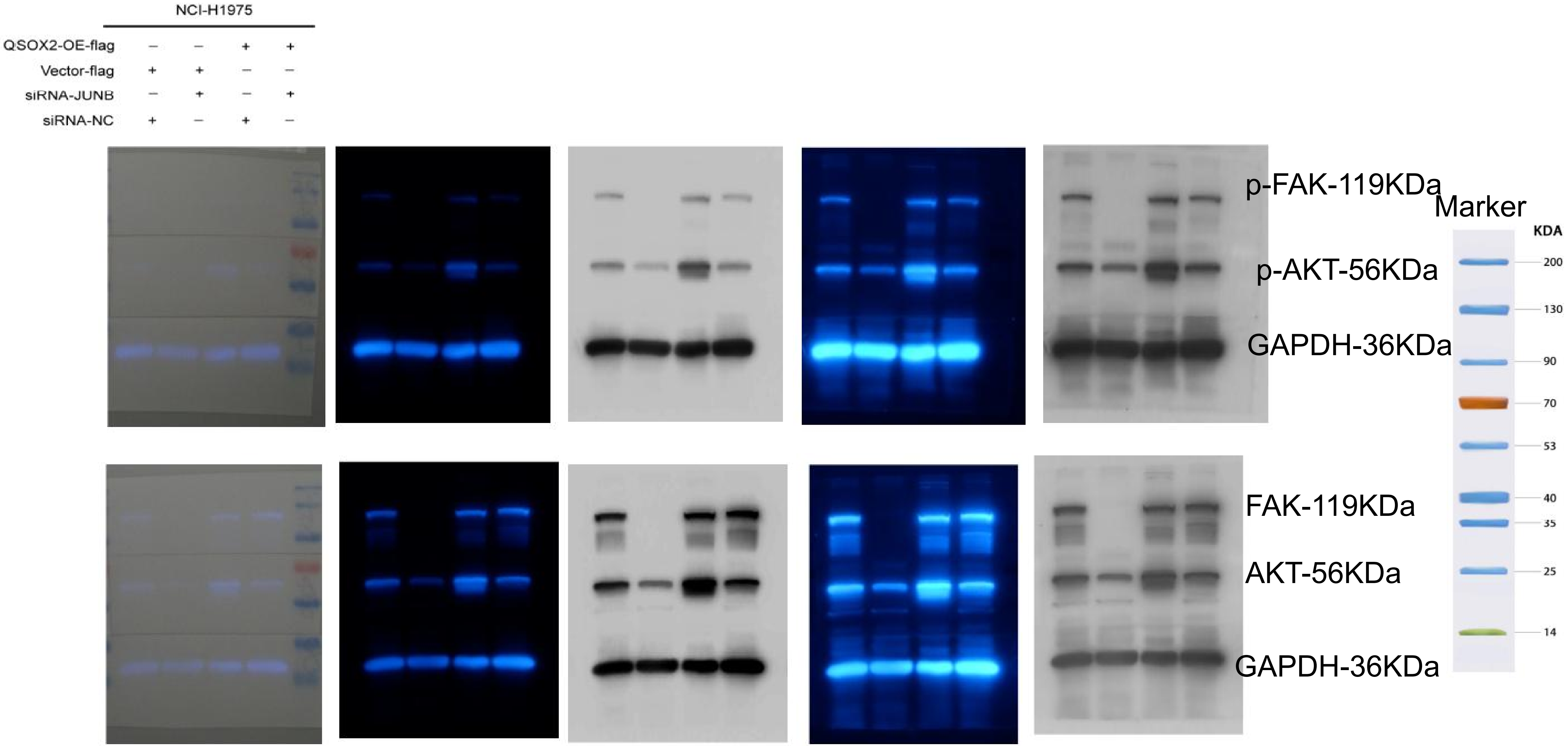
NCI-H1975
QSOX2-OE-flag − − + +
Vector-flag + + − −
siRNA-JUNB − + − +
siRNA-NC + − + −
p-FAK-119KDa
p-AKT-56KDa
GAPDH-36KDa
FAK-119KDa
AKT-56KDa
GAPDH-36KDa
Marker
KDA
200
130
90
70
53
40
35
25
14

figure8D-WB

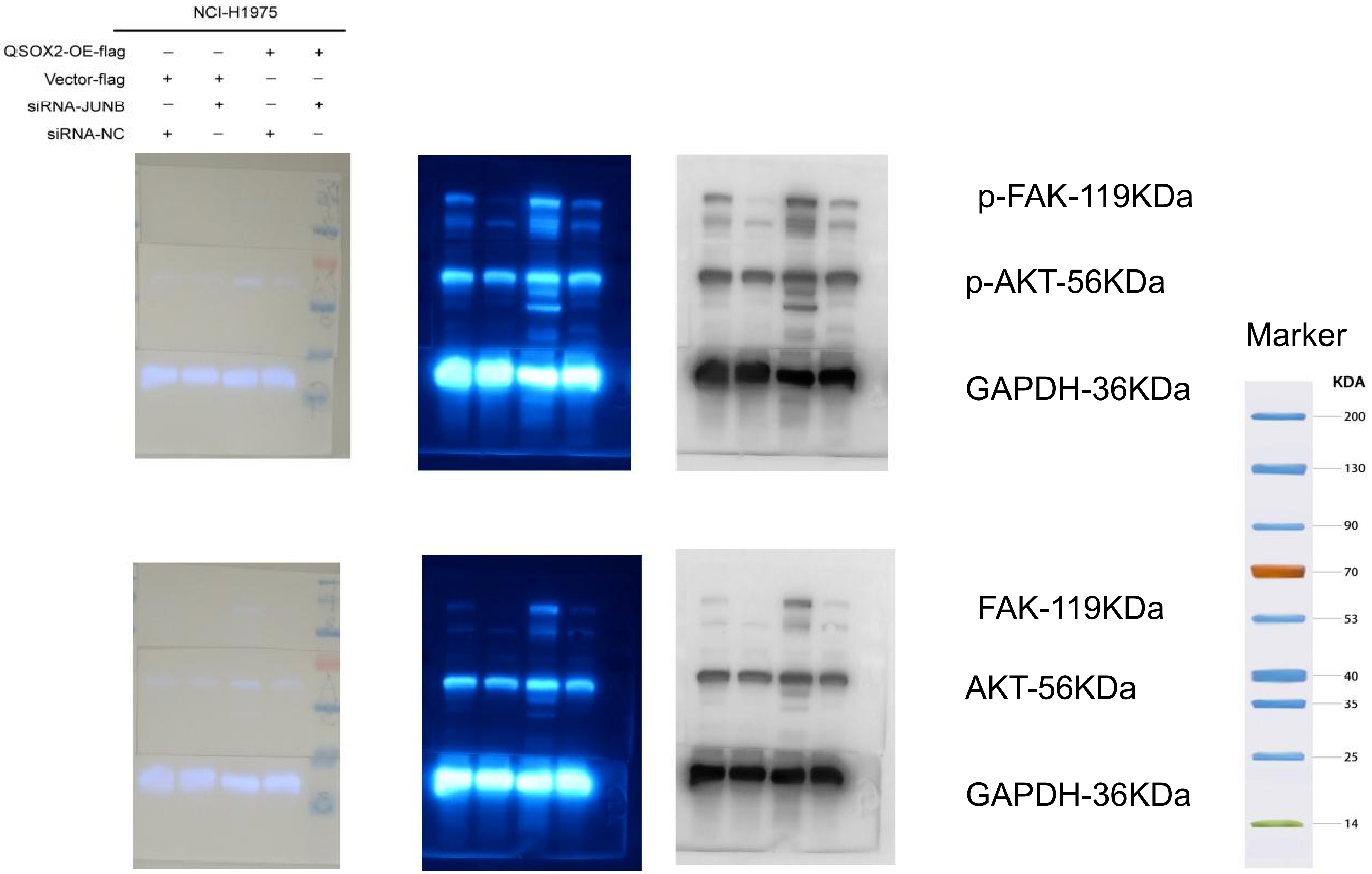
NCI-H1975
QSOX2-OE-flag − − + +
Vector-flag + + − −
siRNA-JUNB − + − +
siRNA-NC + − + −
p-FAK-119KDa
p-AKT-56KDa
GAPDH-36KDa
FAK-119KDa
AKT-56KDa
GAPDH-36KDa
Marker
KDA
200
130
90
70
53
40
35
25
14

figure8E-WB

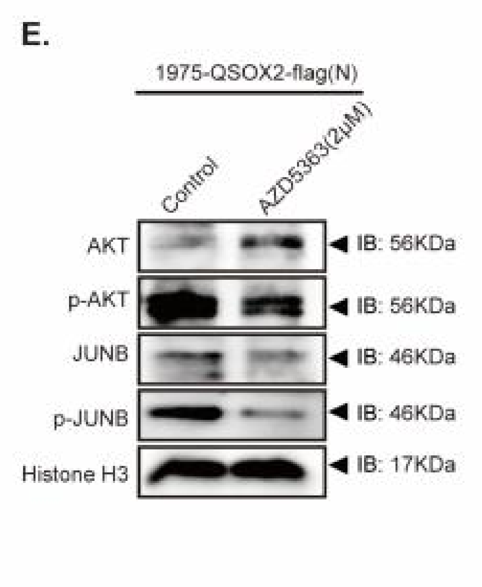
E.
1975-QSOX2-flag(N)
Control
AZD5363(2μM)
AKT
p-AKT
JUNB
p-JUNB
Histone H3
IB: 56KDa
IB: 56KDa
IB: 46KDa
IB: 46KDa
IB: 17KDa

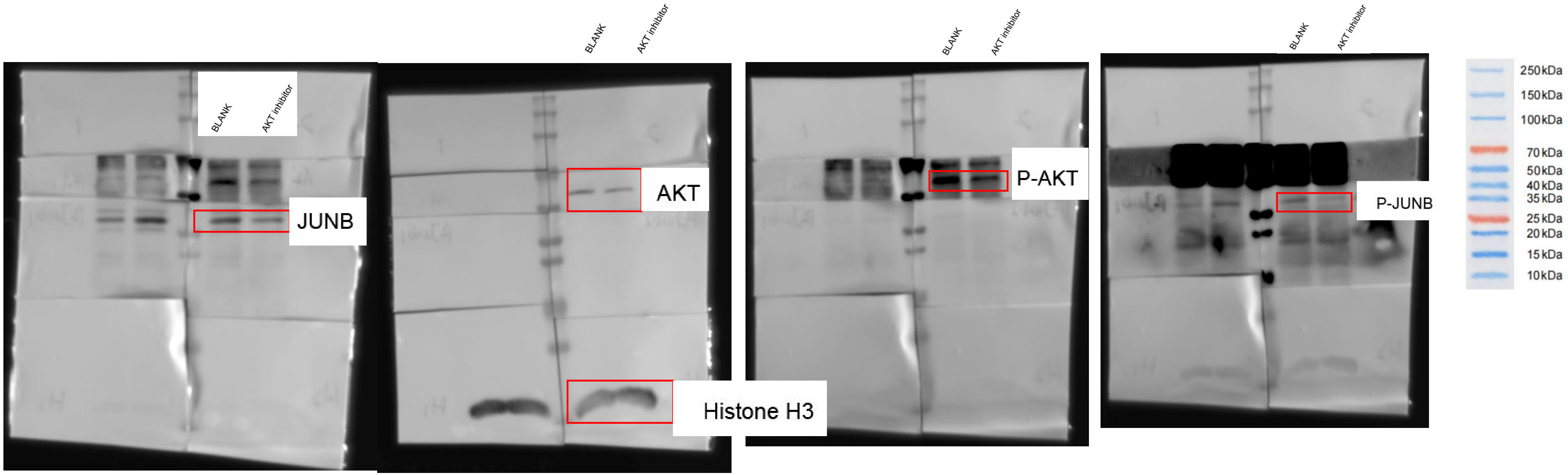
BLANK
AKT inhibitor
JUNB
AKT
Histone H3
P-AKT
P-JUNB
250kDa
150kDa
100kDa
70kDa
50kDa
40kDa
35kDa
25kDa
20kDa
15kDa
10kDa

figure8E-WB

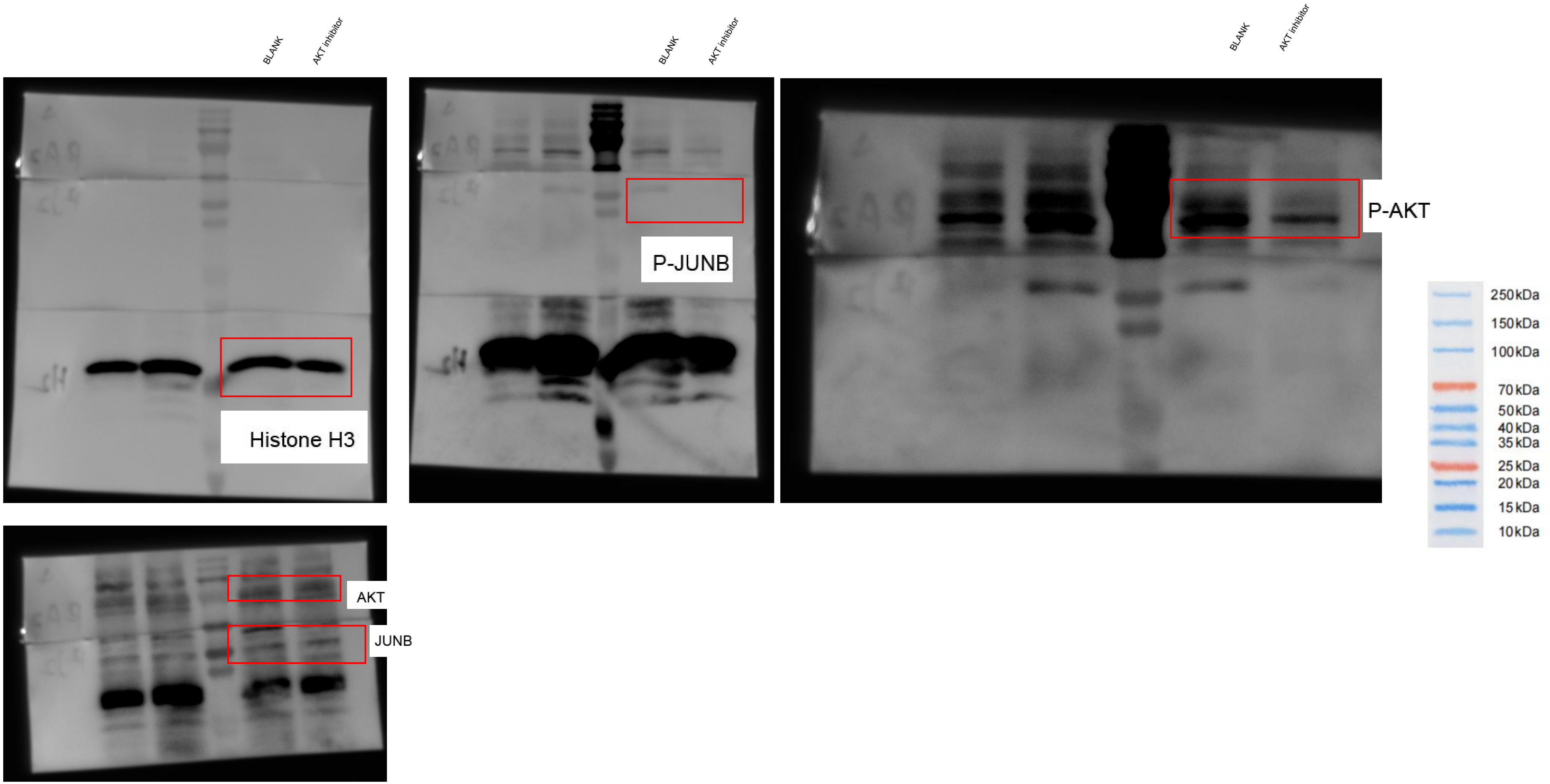
BLANK
AKT inhibitor
Histone H3
BLANK
AKT inhibitor
P-JUNB
BLANK
AKT inhibitor
P-AKT
AKT
JUNB
250kDa
150kDa
100kDa
70kDa
50kDa
40kDa
35kDa
25kDa
20kDa
15kDa
10kDa

figure8E-WB

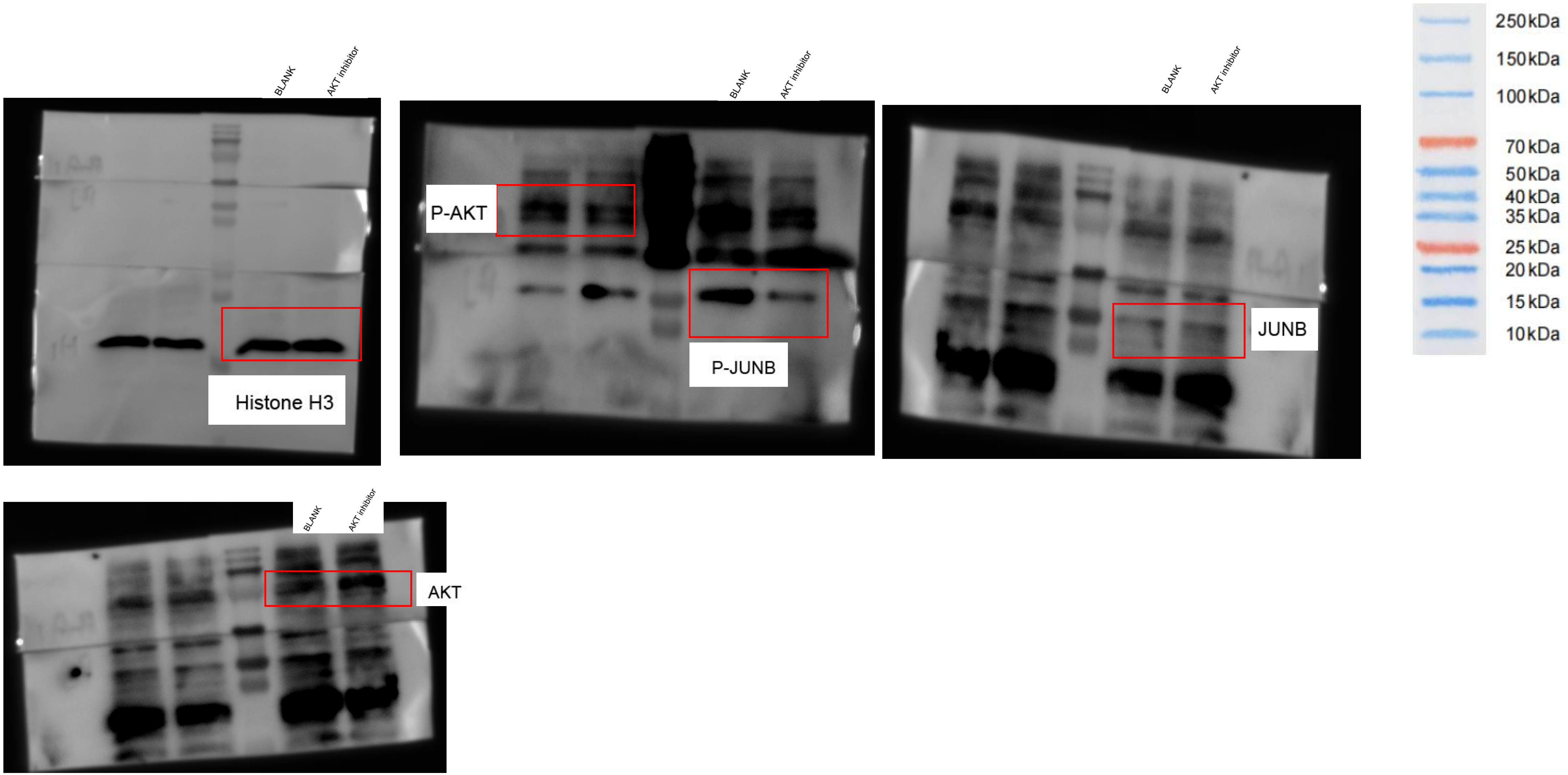
BLANK
AKT inhibitor
Histone H3
BLANK
AKT inhibitor
P-AKT
P-JUNB
BLANK
AKT inhibitor
JUNB
250kDa
150kDa
100kDa
70kDa
50kDa
40kDa
35kDa
25kDa
20kDa
15kDa
10kDa
BLANK
AKT inhibitor
AKT

figureS1C-WB

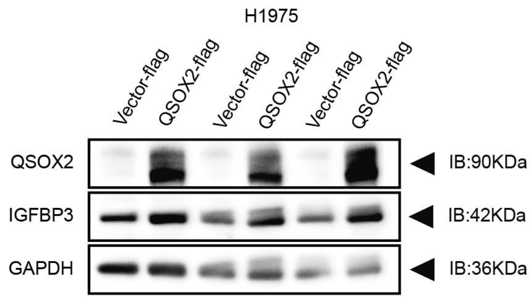

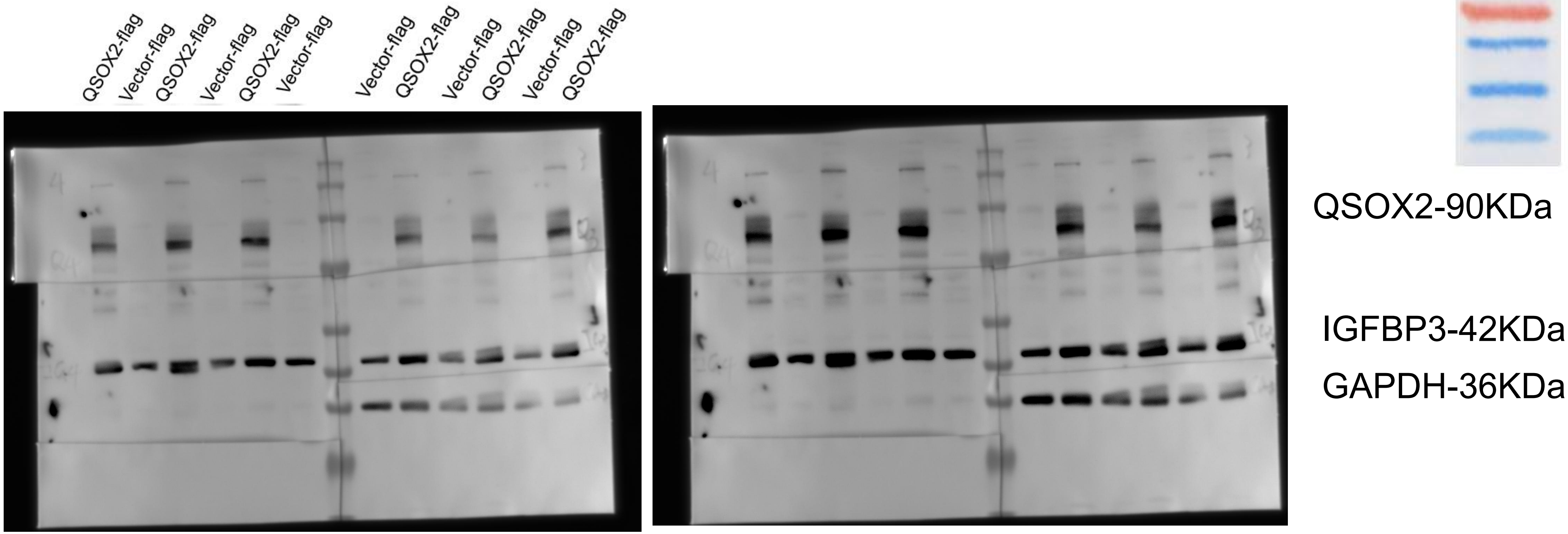

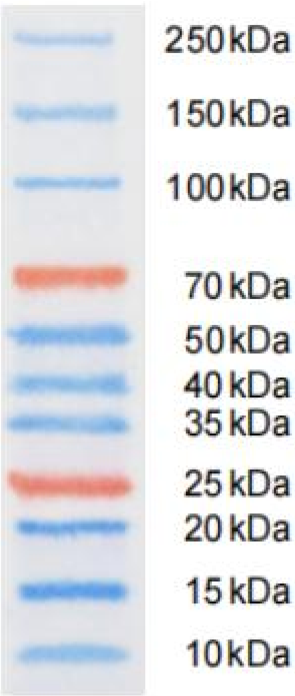

figureS1D-WB

Marker

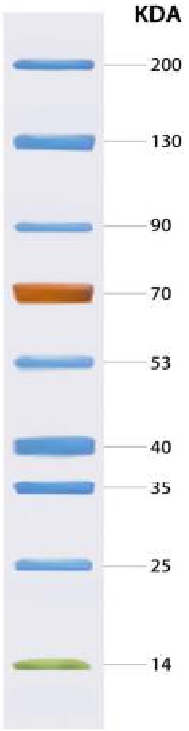

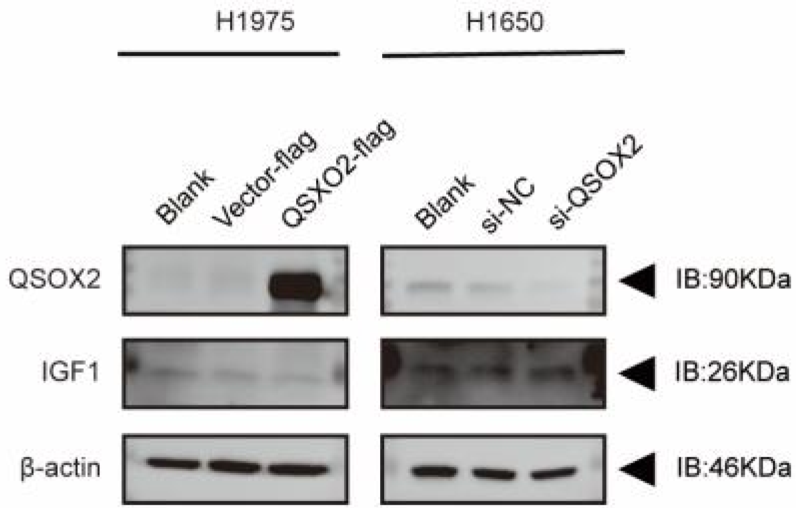

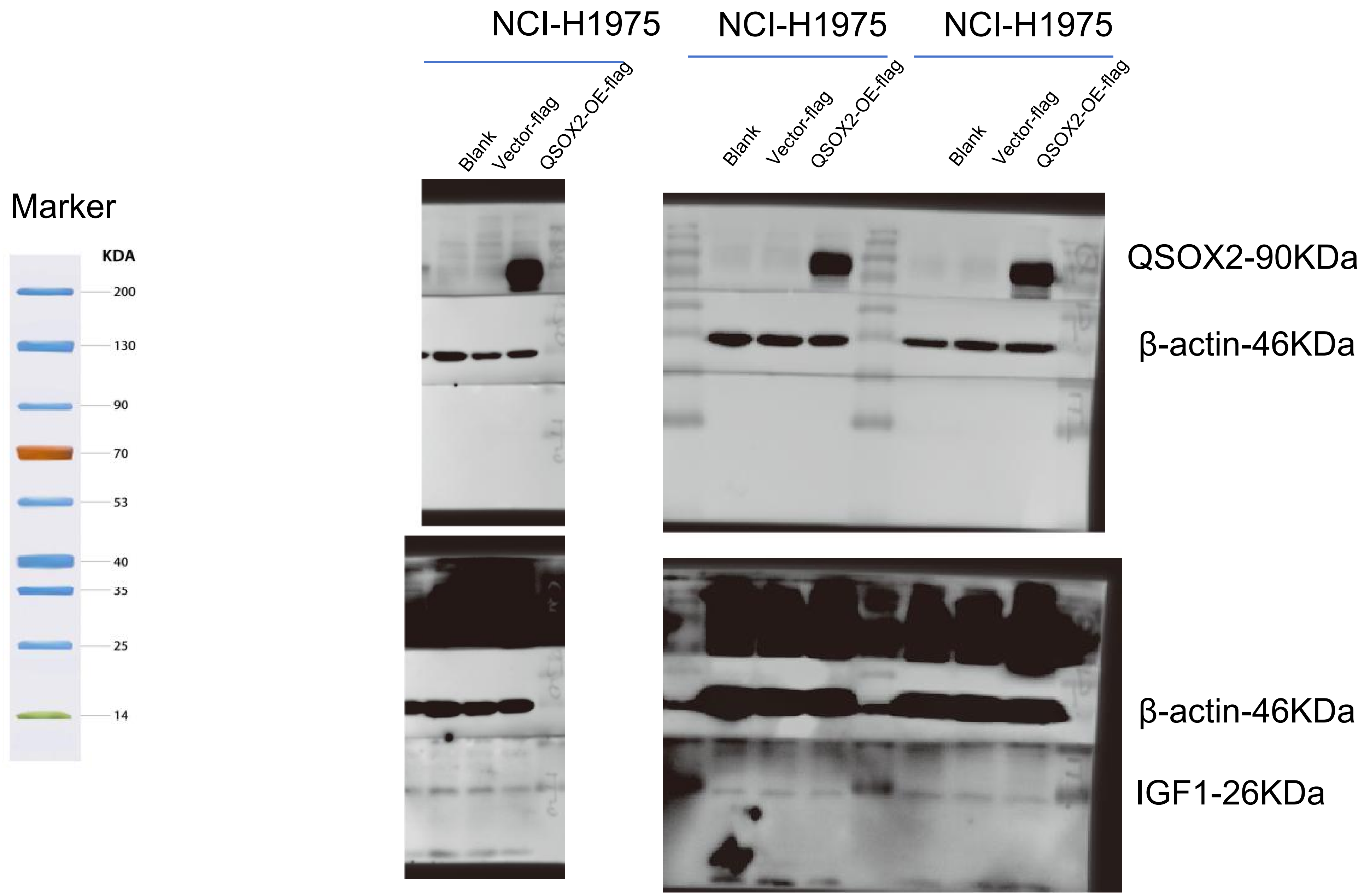

Marker
KDA
200
130
90
70
53
40
35
25
14
NCI-H1975
NCI-H1975
NCI-H1975
Blank
Vector-flag
QSOX2-OE-flag
Blank
Vector-flag
QSOX2-OE-flag
Blank
Vector-flag
QSOX2-OE-flag
QSOX2-90KDa
β-actin-46KDa
β-actin-46KDa
IGF1-26KDa

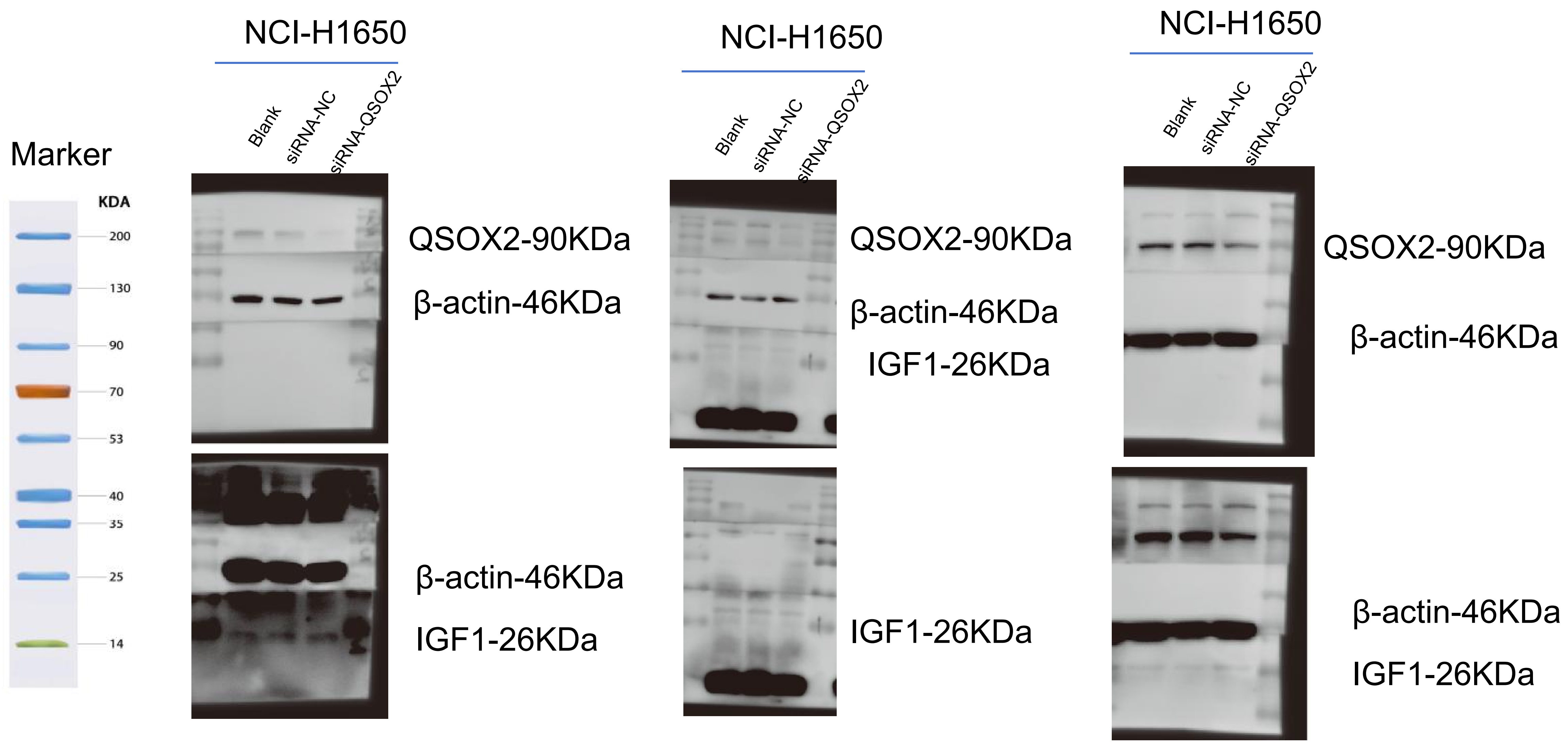
Marker
KDA
200
130
90
70
53
40
35
25
14
NCI-H1650
Blank
siRNA-NC
siRNA-QSOX2
QSOX2-90KDa
β-actin-46KDa
β-actin-46KDa
IGF1-26KDa
NCI-H1650
Blank
siRNA-NC
siRNA-QSOX2
QSOX2-90KDa
β-actin-46KDa
IGF1-26KDa
IGF1-26KDa
NCI-H1650
Blank
siRNA-NC
siRNA-QSOX2
QSOX2-90KDa
β-actin-46KDa
β-actin-46KDa
IGF1-26KDa

figureS3A-WB

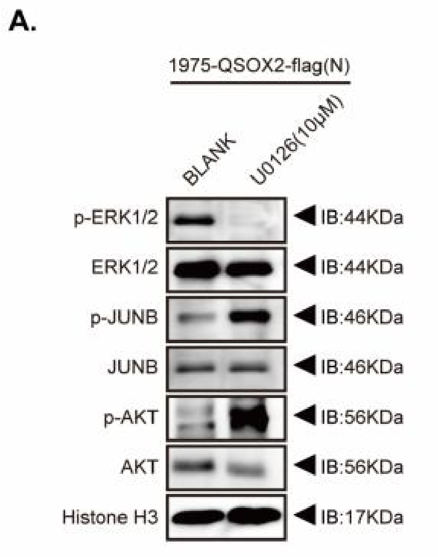

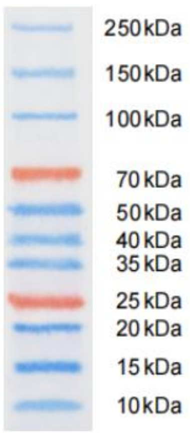

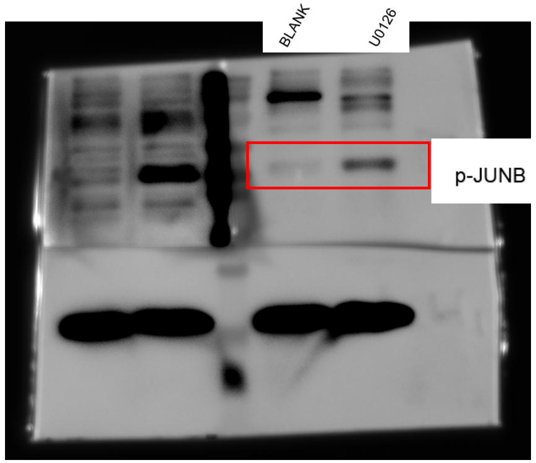

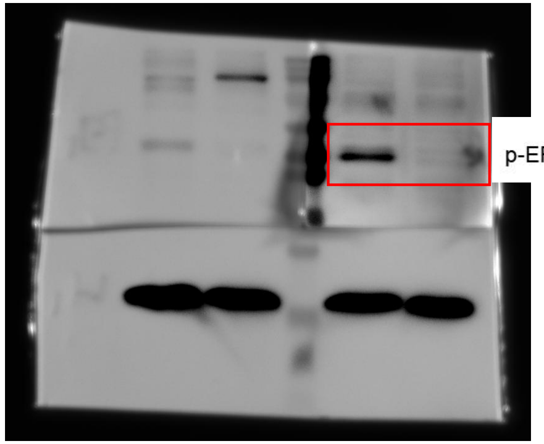

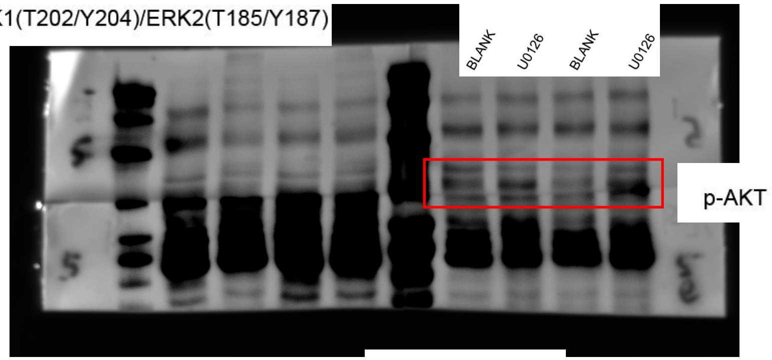

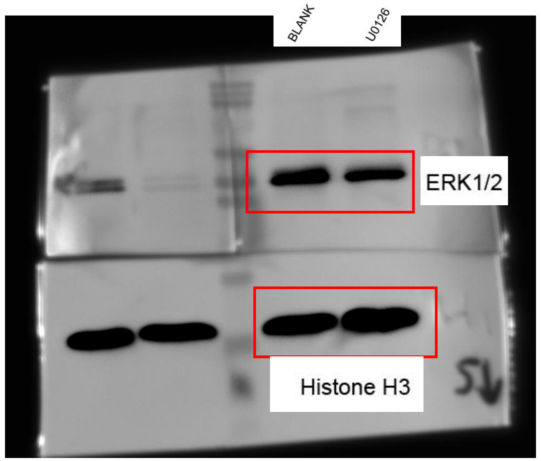

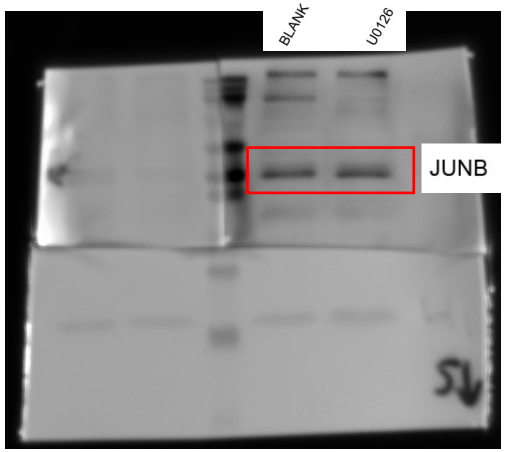

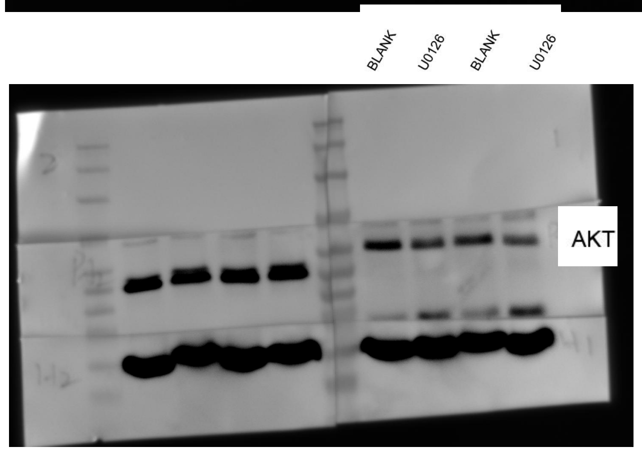

figureS3A-WB

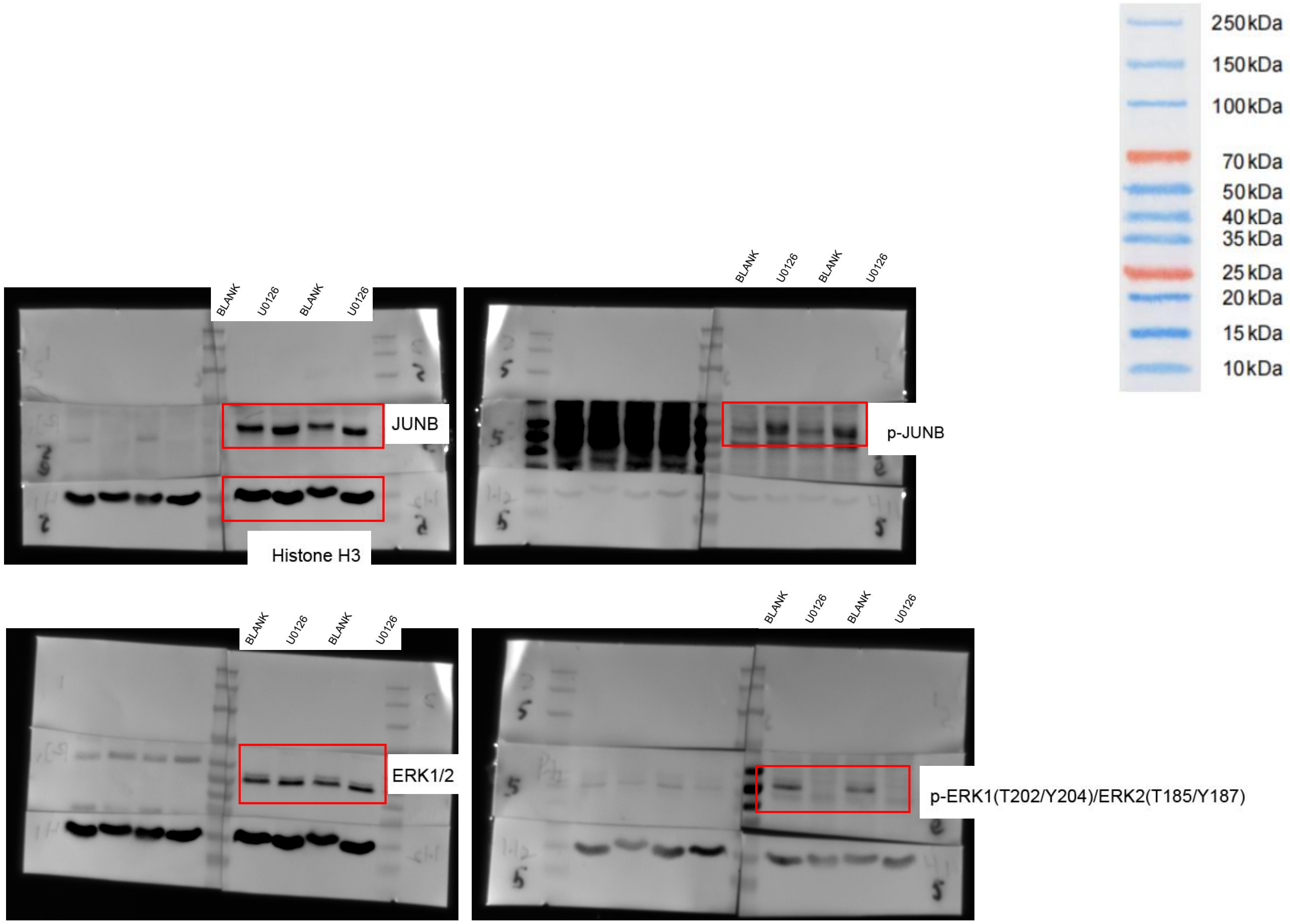

figureS3A-WB

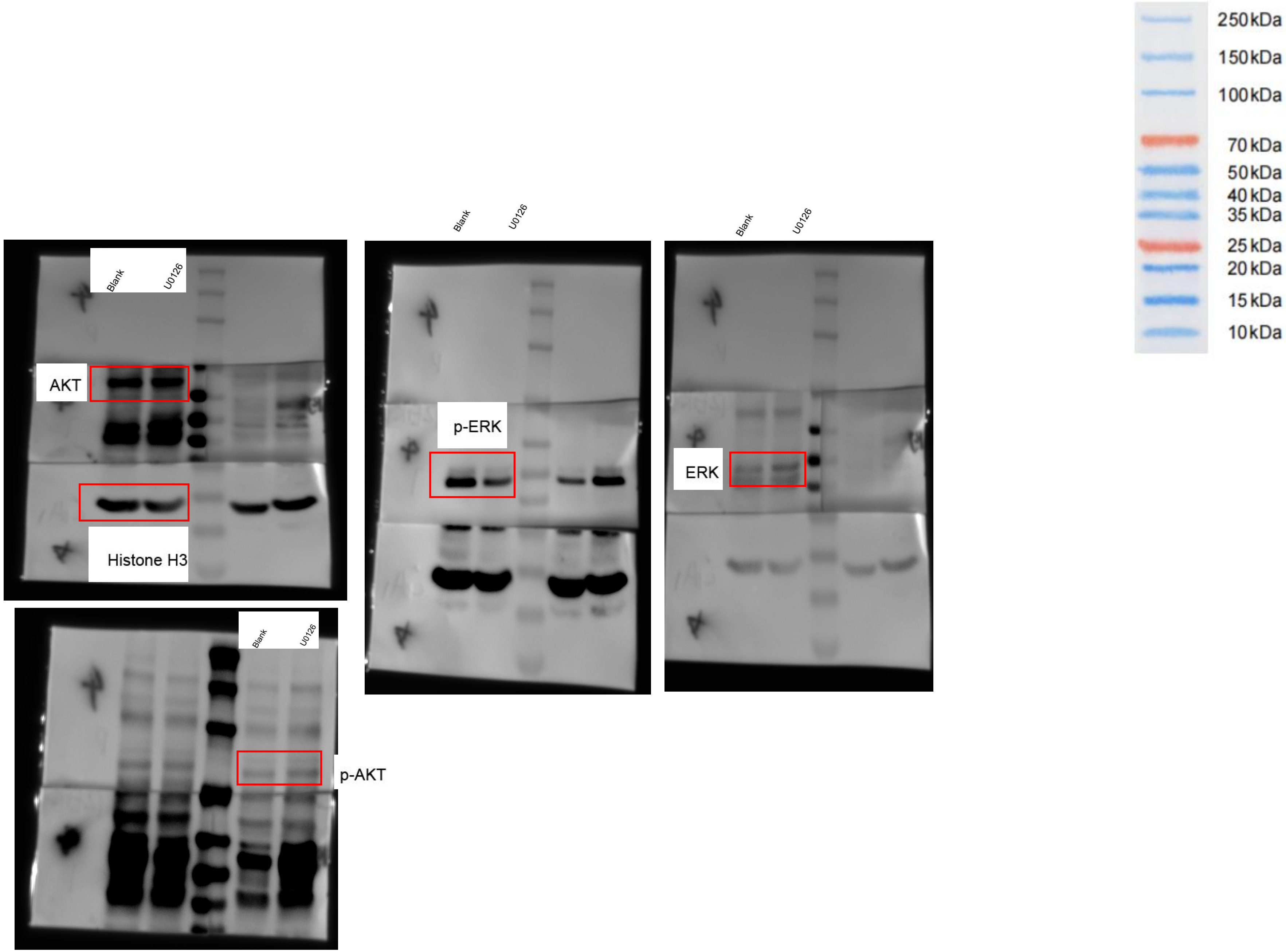

figureS3A-WB

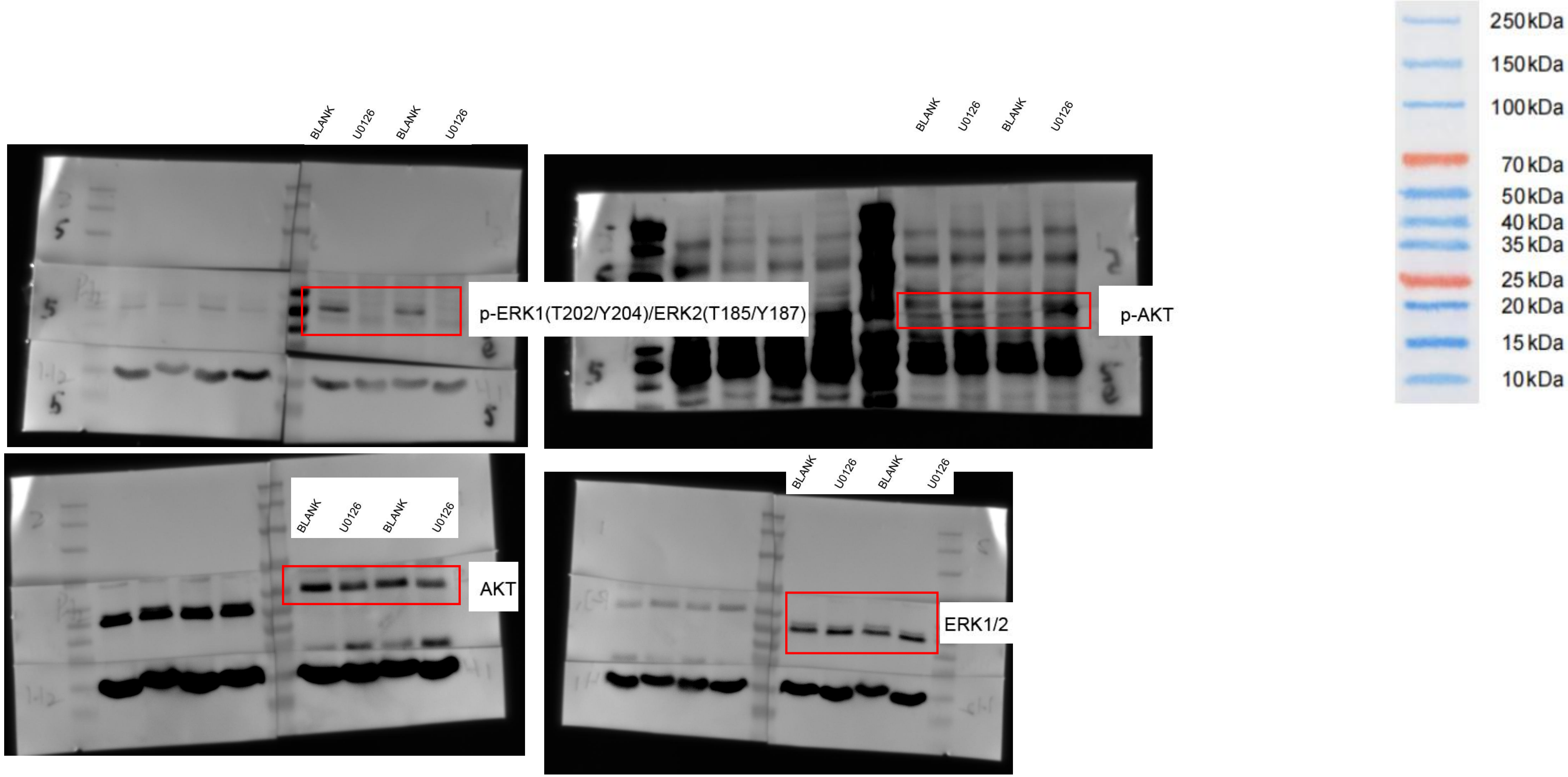

figureS3B-WB

Marker

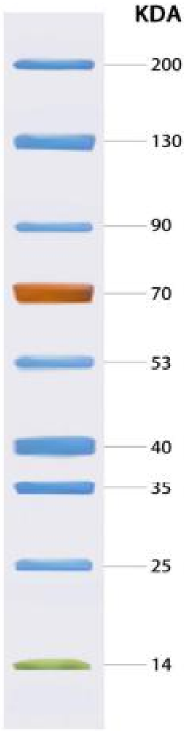

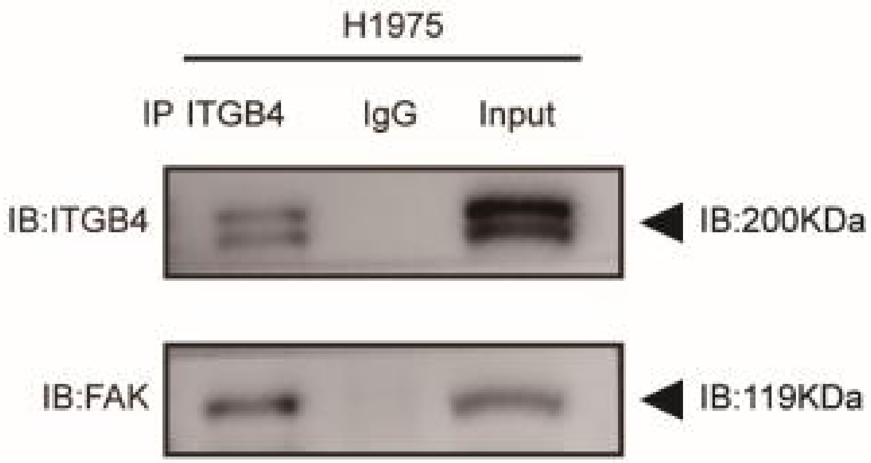

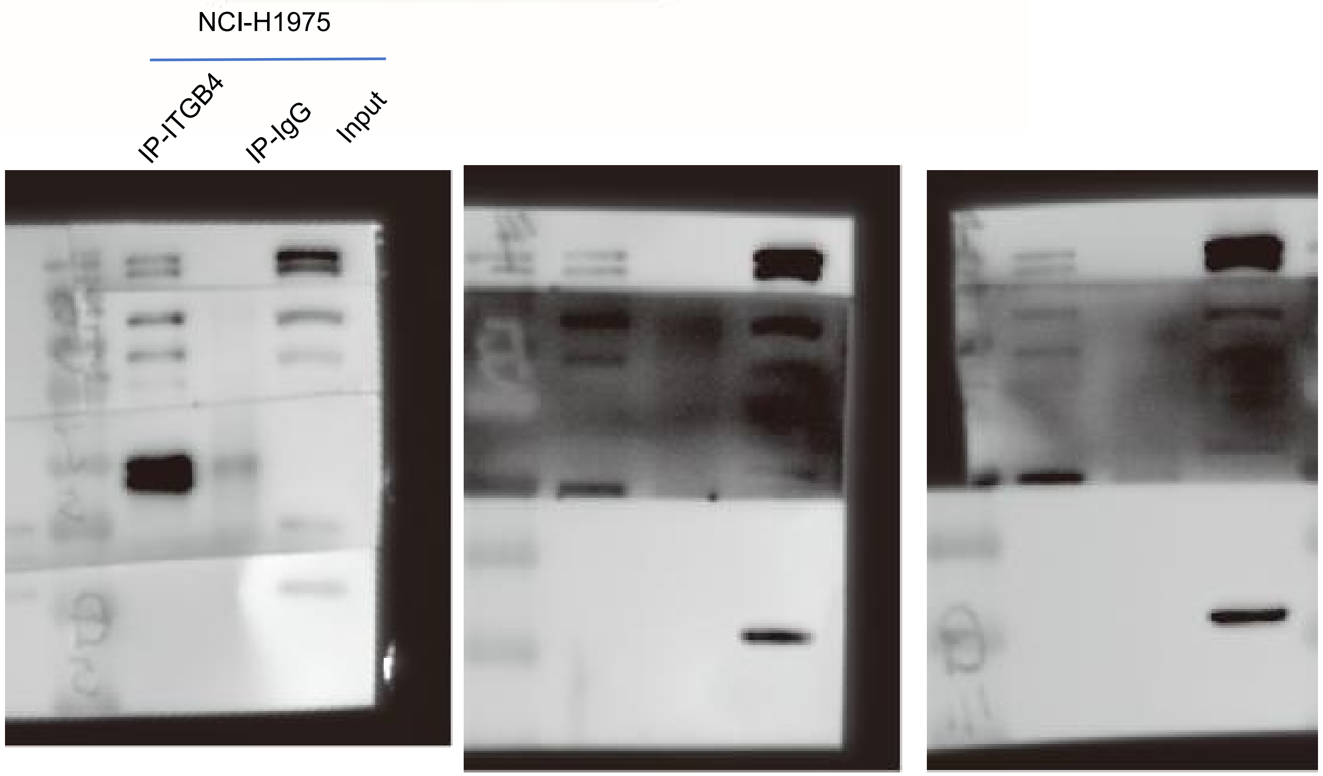

ITGB4-200KDa

FAK-119KDa

figureS3C-WB

Marker

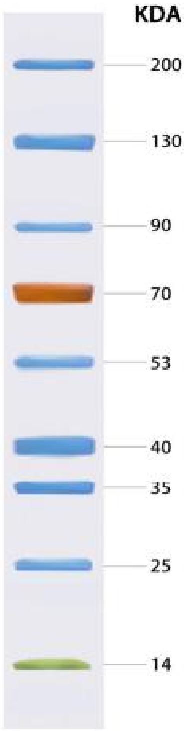

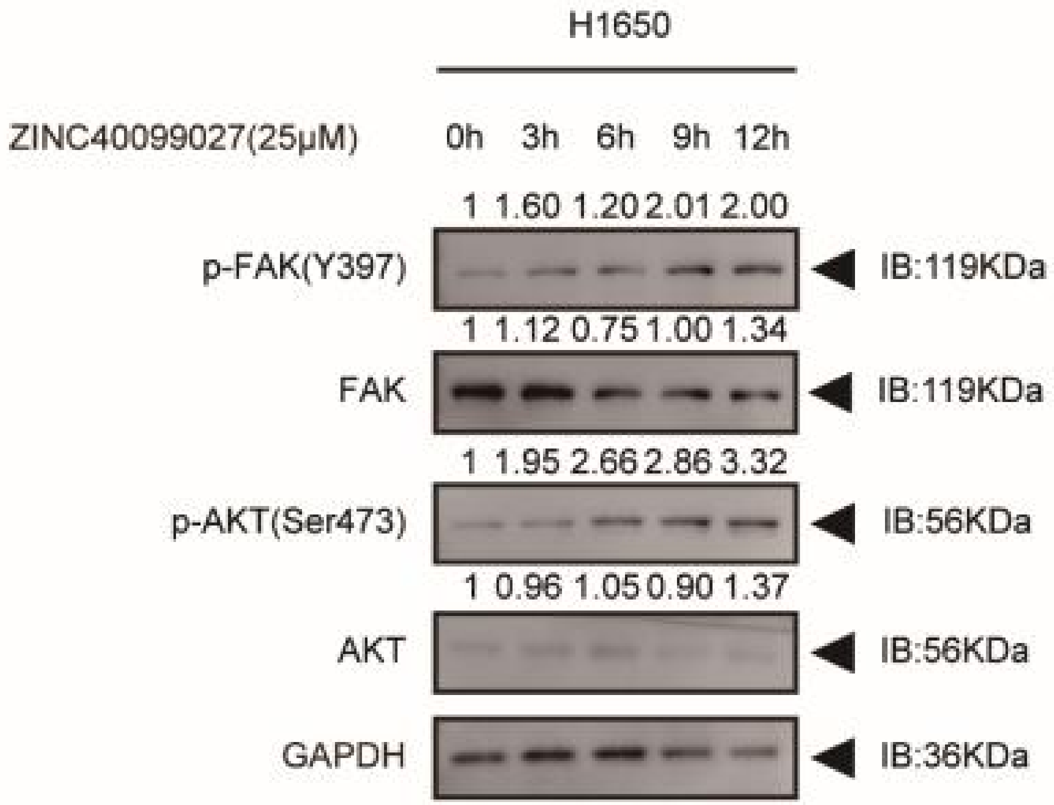

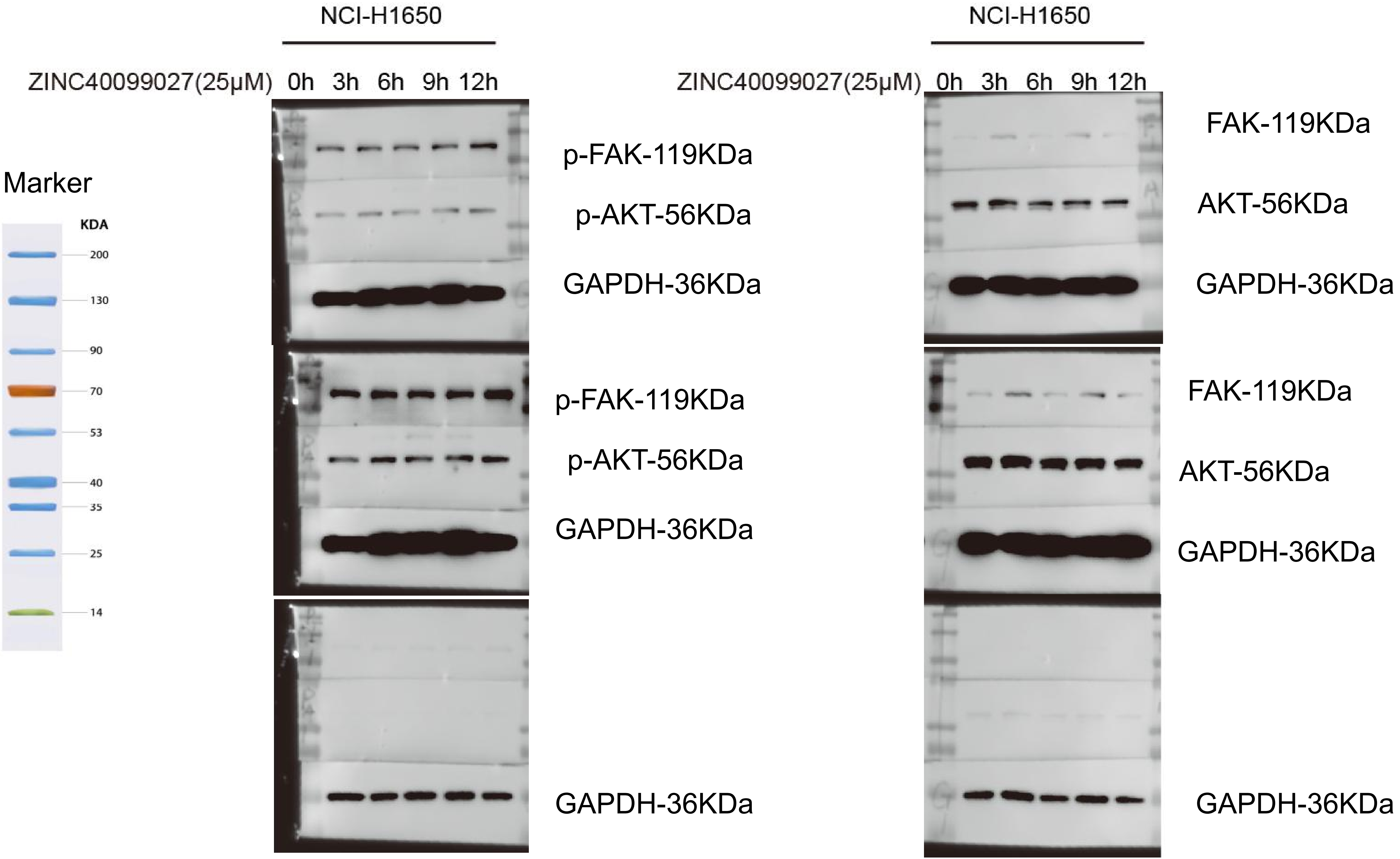
NCI-H1650
ZINC40099027(25μM) 0h 3h 6h 9h 12h
Marker
KDA
200
130
90
70
53
40
35
25
14
p-FAK-119KDa
p-AKT-56KDa
GAPDH-36KDa
p-FAK-119KDa
p-AKT-56KDa
GAPDH-36KDa
GAPDH-36KDa
NCI-H1650
ZINC40099027(25μM) 0h 3h 6h 9h 12h
FAK-119KDa
AKT-56KDa
GAPDH-36KDa
FAK-119KDa
AKT-56KDa
GAPDH-36KDa
GAPDH-36KDa

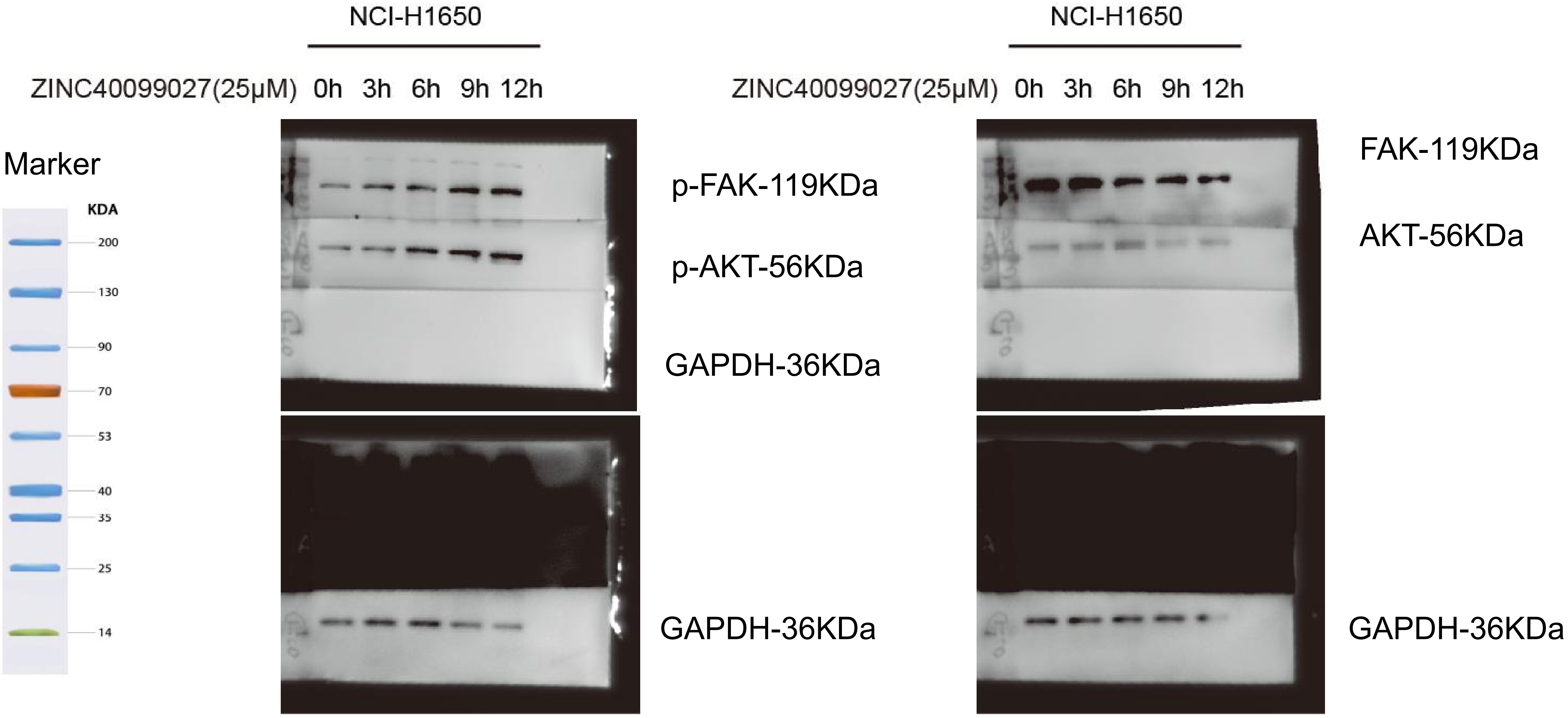

NCI-H1650
ZINC40099027(25μM) 0h 3h 6h 9h 12h
Marker
KDA
200
130
90
70
53
40
35
25
14
p-FAK-119KDa
p-AKT-56KDa
GAPDH-36KDa
GAPDH-36KDa
NCI-H1650
ZINC40099027(25μM) 0h 3h 6h 9h 12h
FAK-119KDa
AKT-56KDa
GAPDH-36KDa

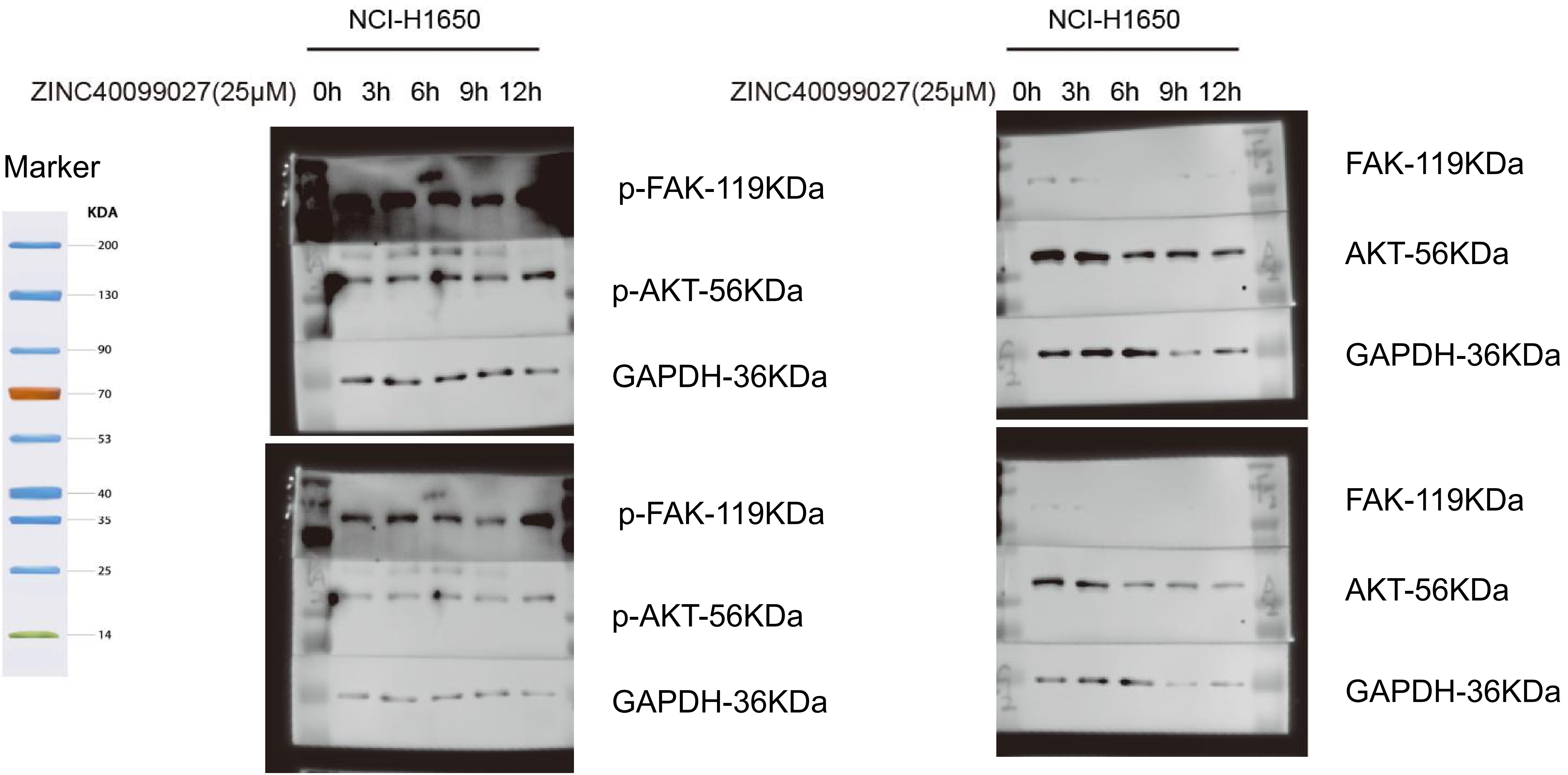
NCI-H1650
ZINC40099027(25μM) 0h 3h 6h 9h 12h
Marker
KDA
200
130
90
70
53
40
35
25
14
p-FAK-119KDa
p-AKT-56KDa
GAPDH-36KDa
p-FAK-119KDa
p-AKT-56KDa
GAPDH-36KDa
NCI-H1650
ZINC40099027(25μM) 0h 3h 6h 9h 12h
FAK-119KDa
AKT-56KDa
GAPDH-36KDa
FAK-119KDa
AKT-56KDa
GAPDH-36KDa

figureS3D-WB

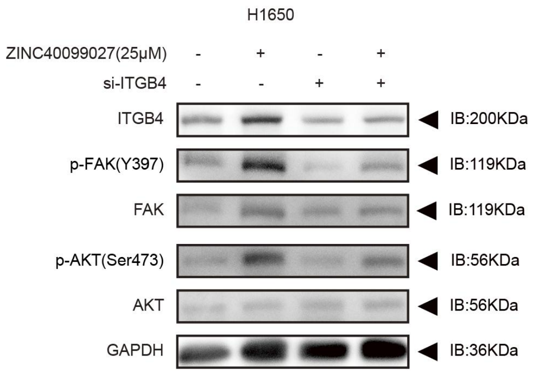

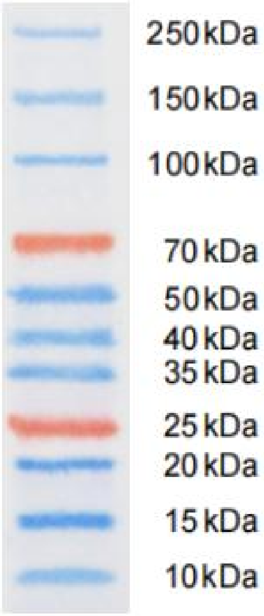

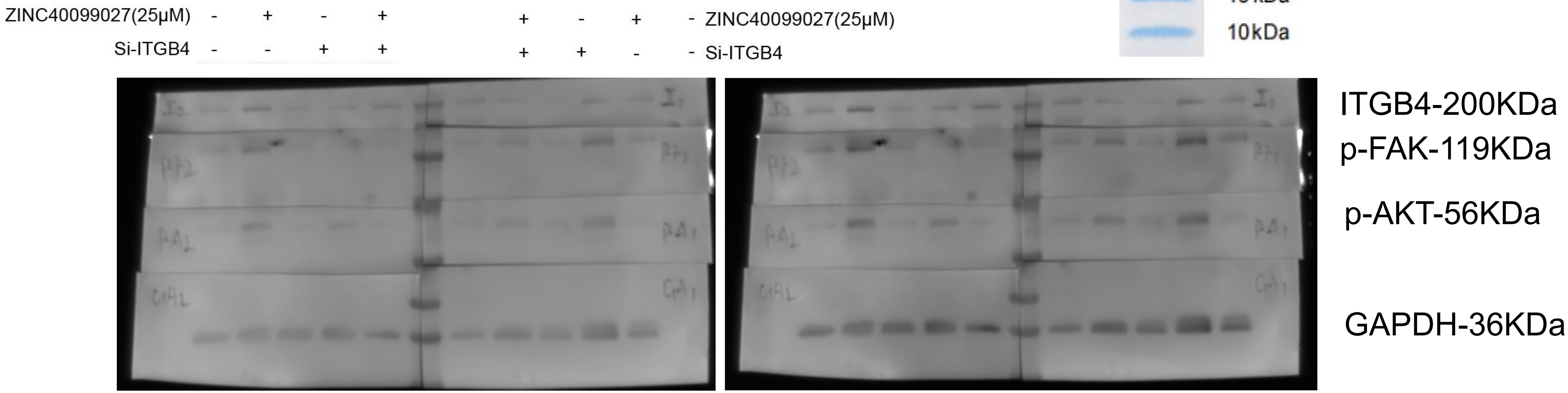

figureS3D-WB

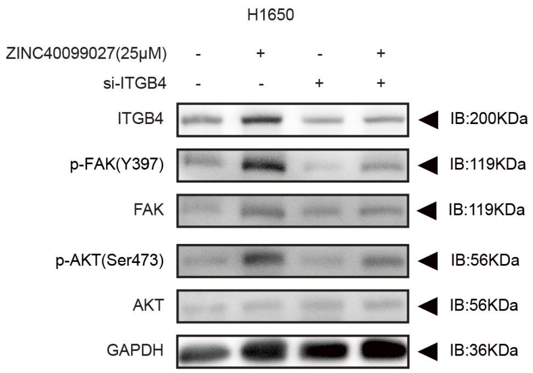

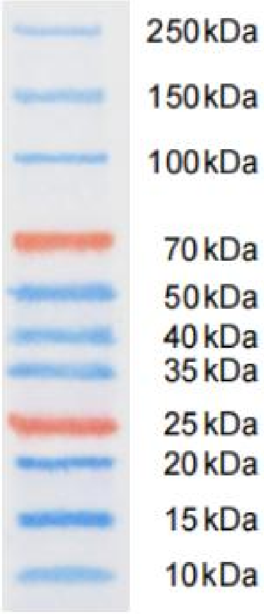

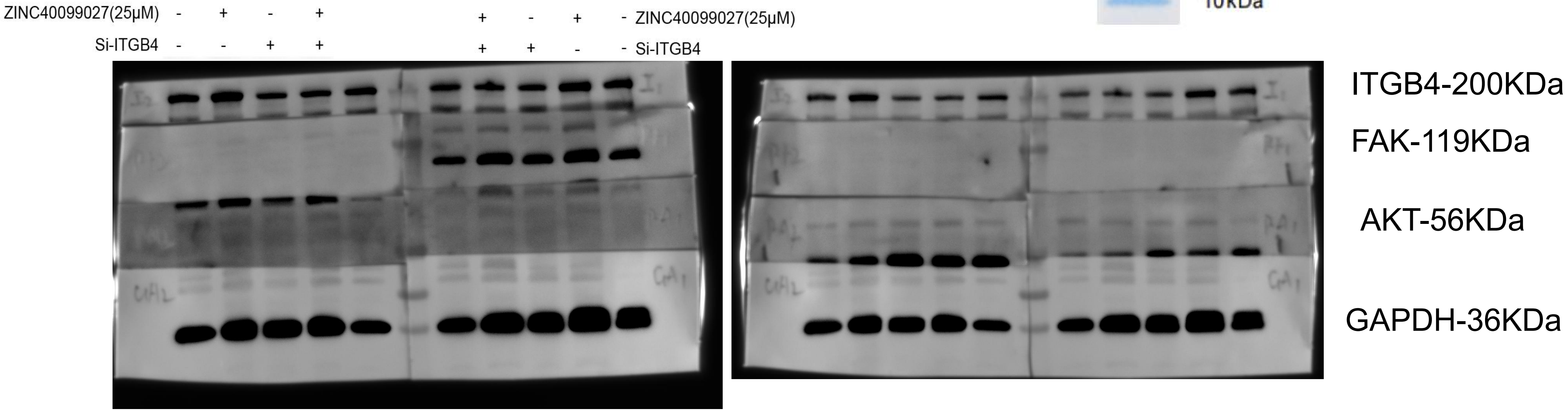

figureS3D-WB

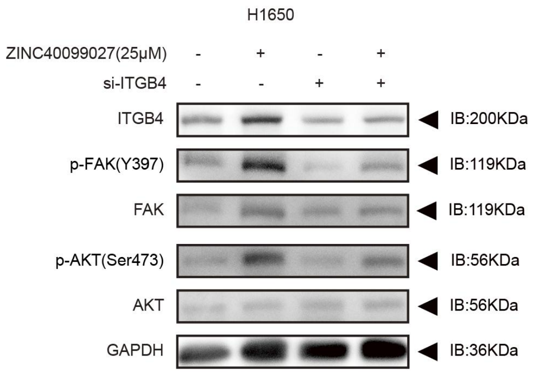

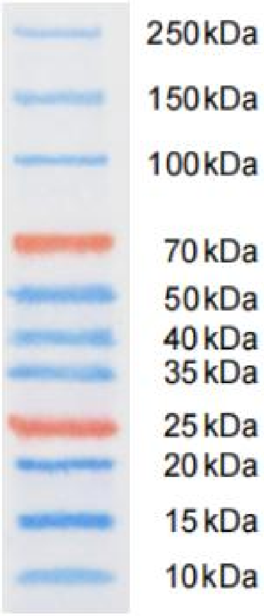

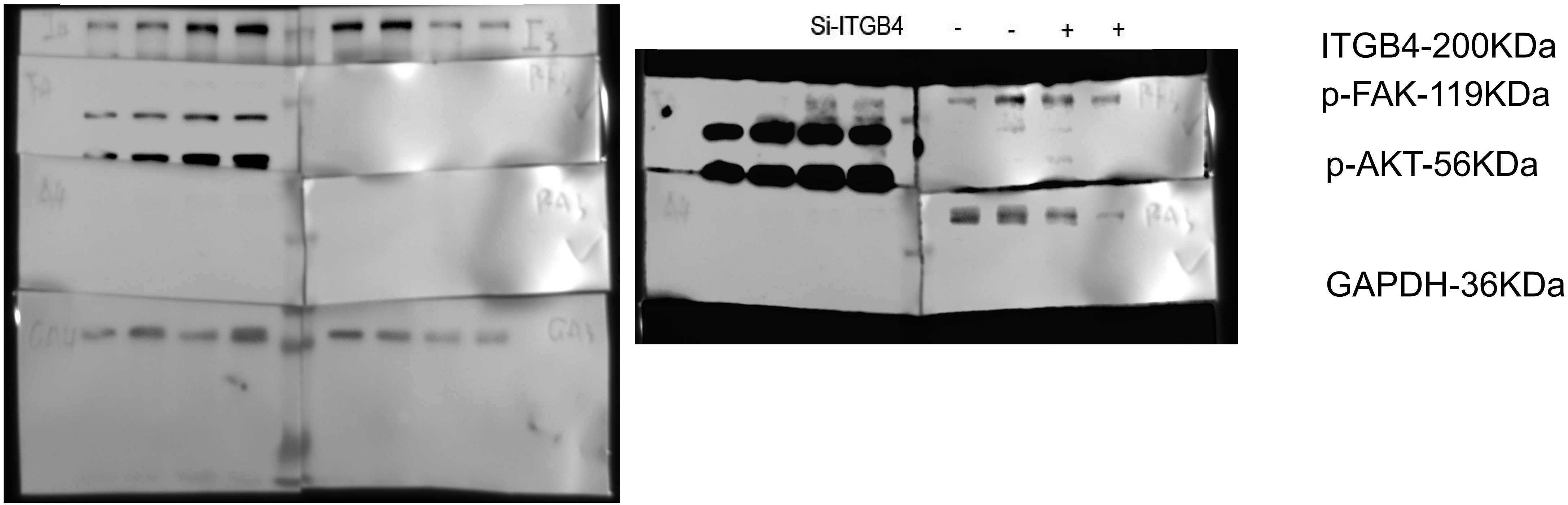

figureS3D-WB

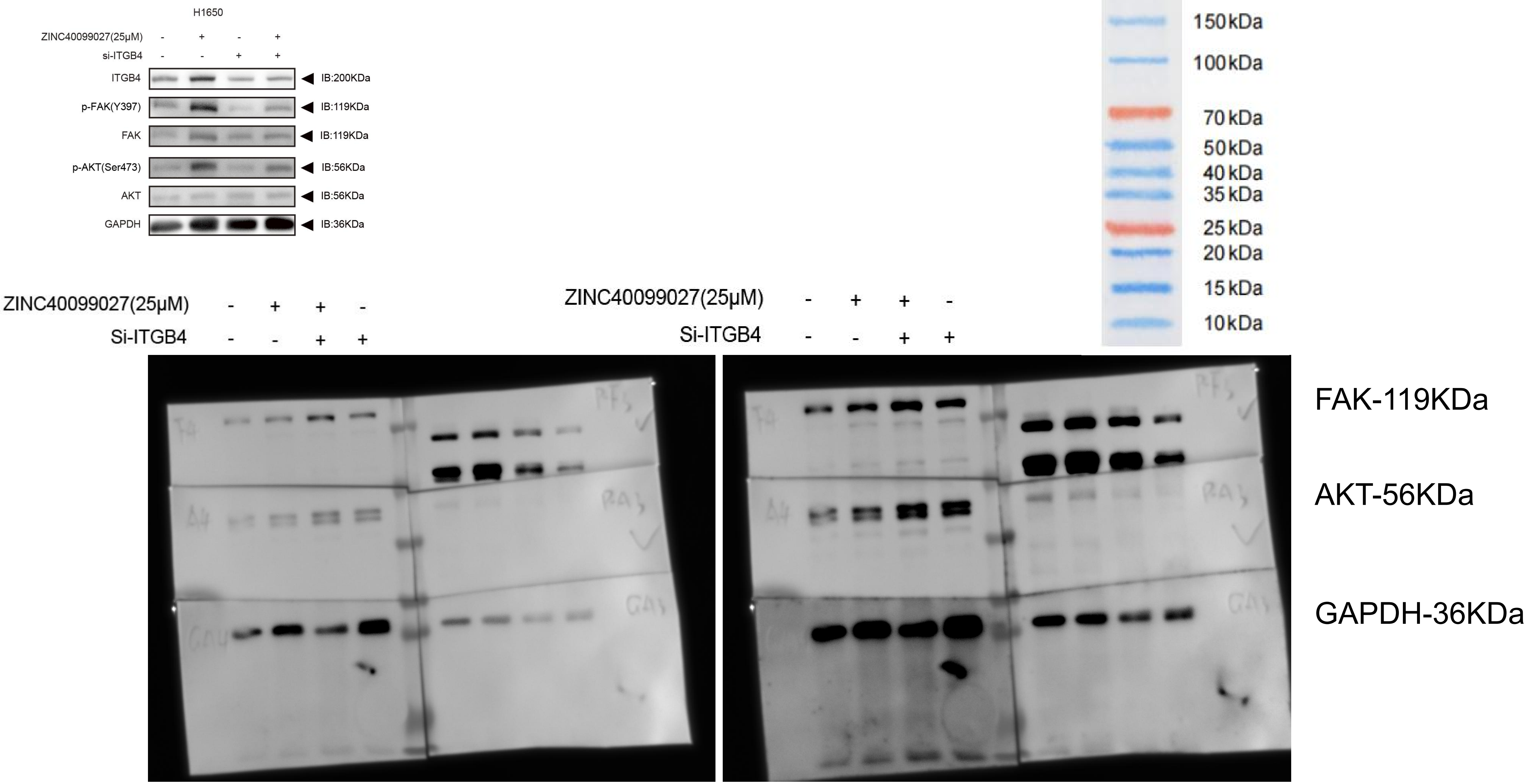

figureS4A-WB

Marker

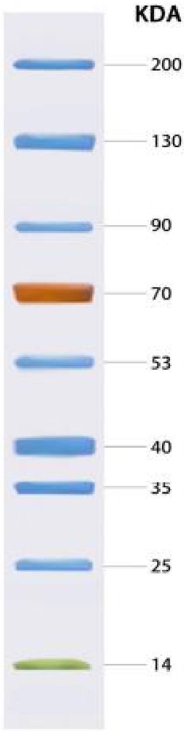

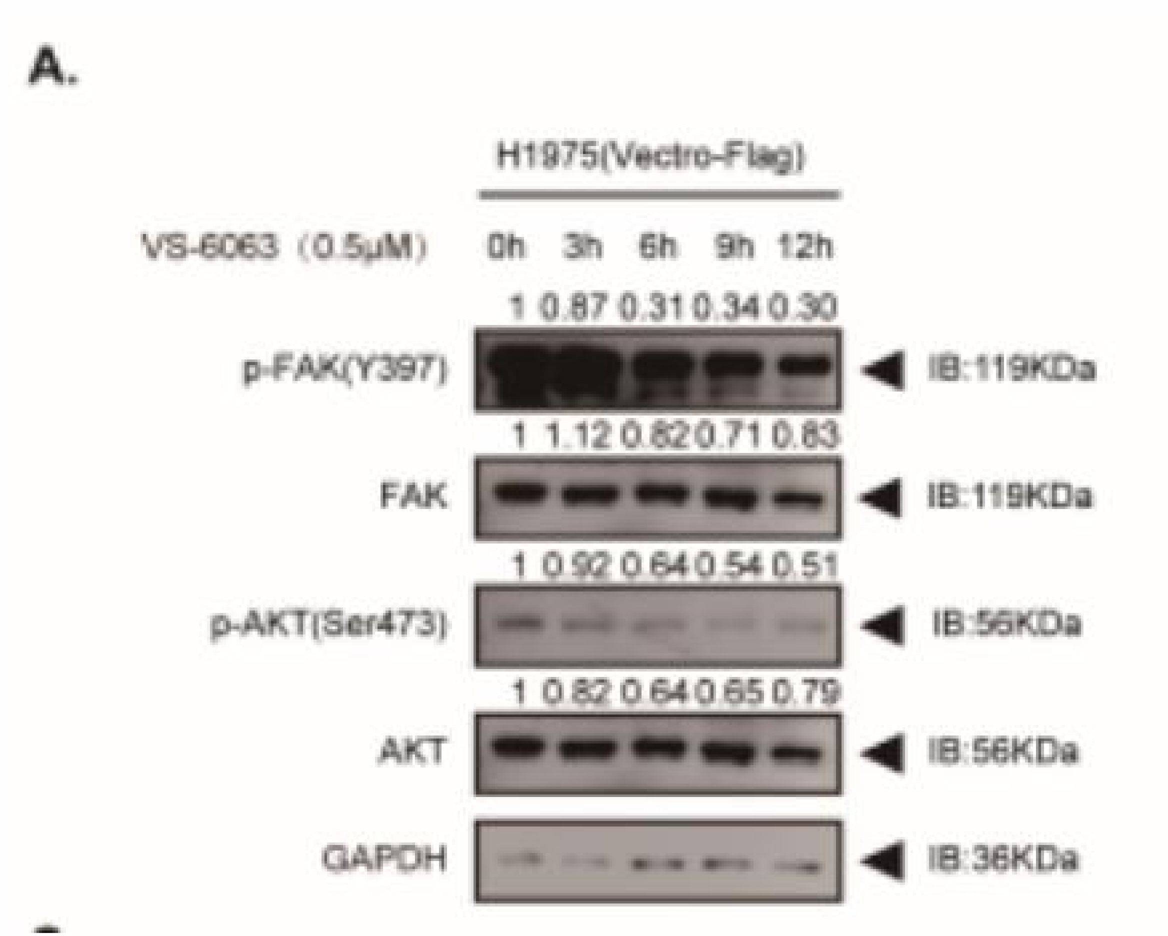

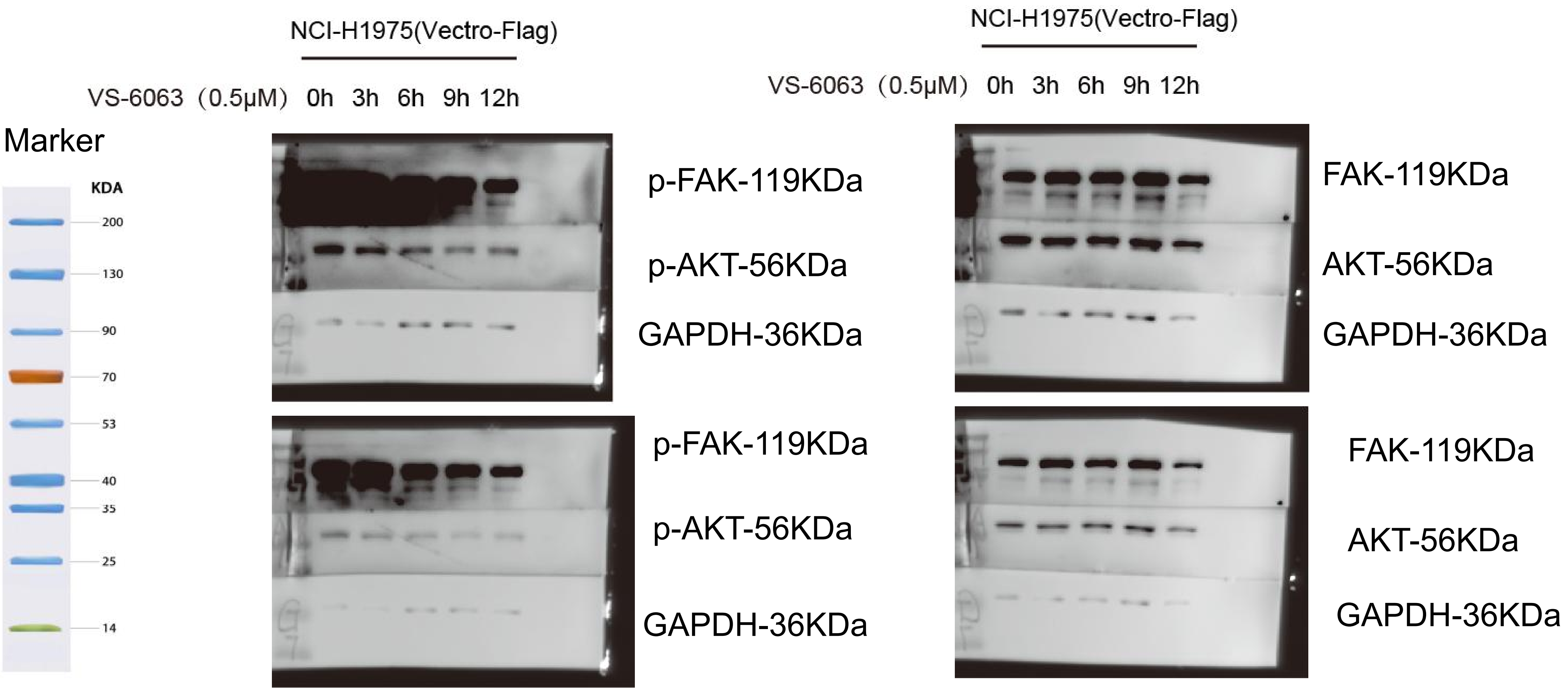
NCI-H1975(Vectro-Flag)
VS-6063（0.5μM） 0h 3h 6h 9h 12h
Marker
KDA
200
130
90
70
53
40
35
25
14
p-FAK-119KDa
p-AKT-56KDa
GAPDH-36KDa
p-FAK-119KDa
p-AKT-56KDa
GAPDH-36KDa
NCI-H1975(Vectro-Flag)
VS-6063（0.5μM） 0h 3h 6h 9h 12h
FAK-119KDa
AKT-56KDa
GAPDH-36KDa
FAK-119KDa
AKT-56KDa
GAPDH-36KDa

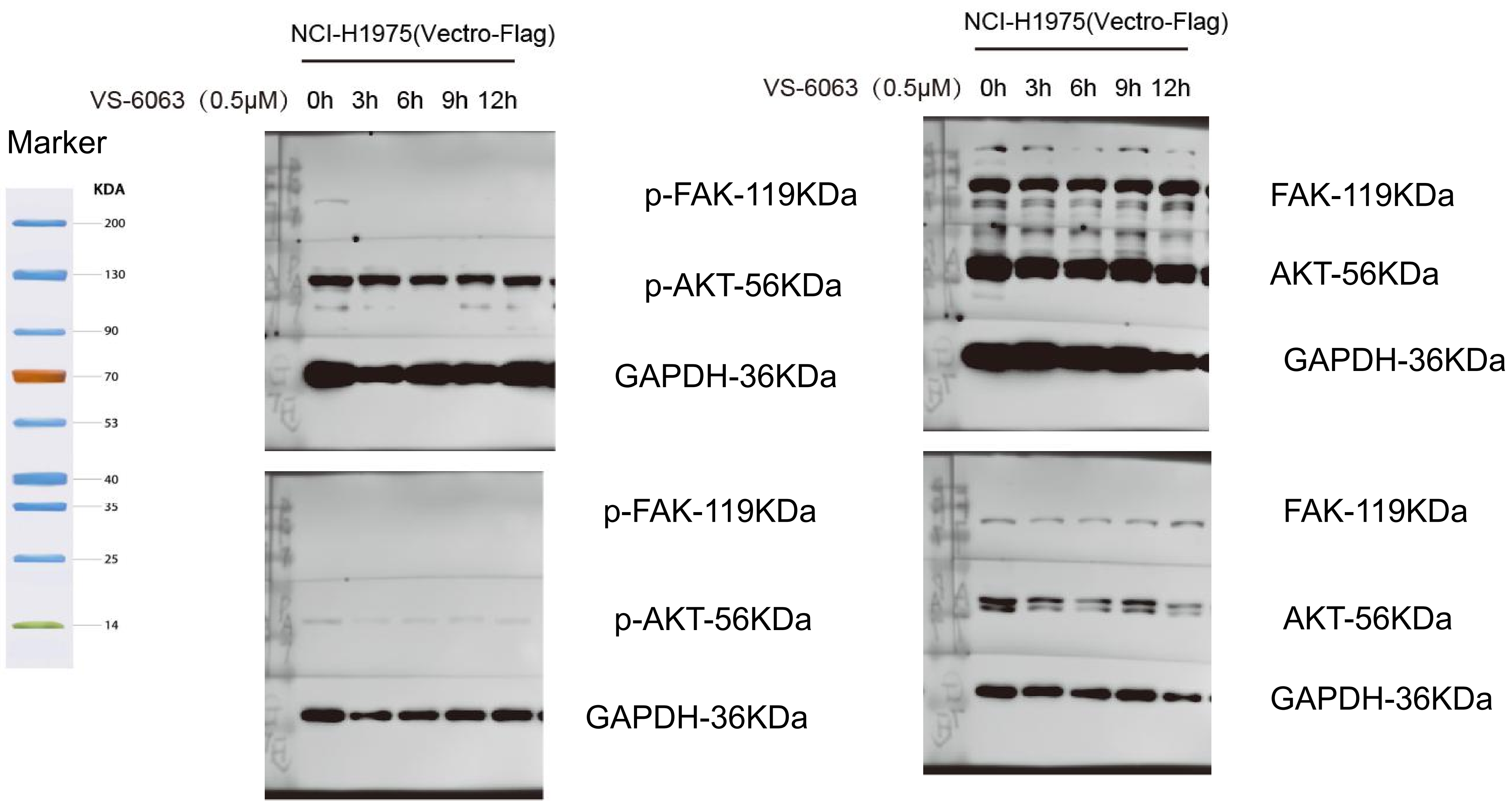
NCI-H1975(Vectro-Flag)
VS-6063（0.5μM） 0h 3h 6h 9h 12h
Marker
KDA
200
130
90
70
53
40
35
25
14
p-FAK-119KDa
p-AKT-56KDa
GAPDH-36KDa
p-FAK-119KDa
p-AKT-56KDa
GAPDH-36KDa
NCI-H1975(Vectro-Flag)
VS-6063（0.5μM） 0h 3h 6h 9h 12h
FAK-119KDa
AKT-56KDa
GAPDH-36KDa
FAK-119KDa
AKT-56KDa
GAPDH-36KDa

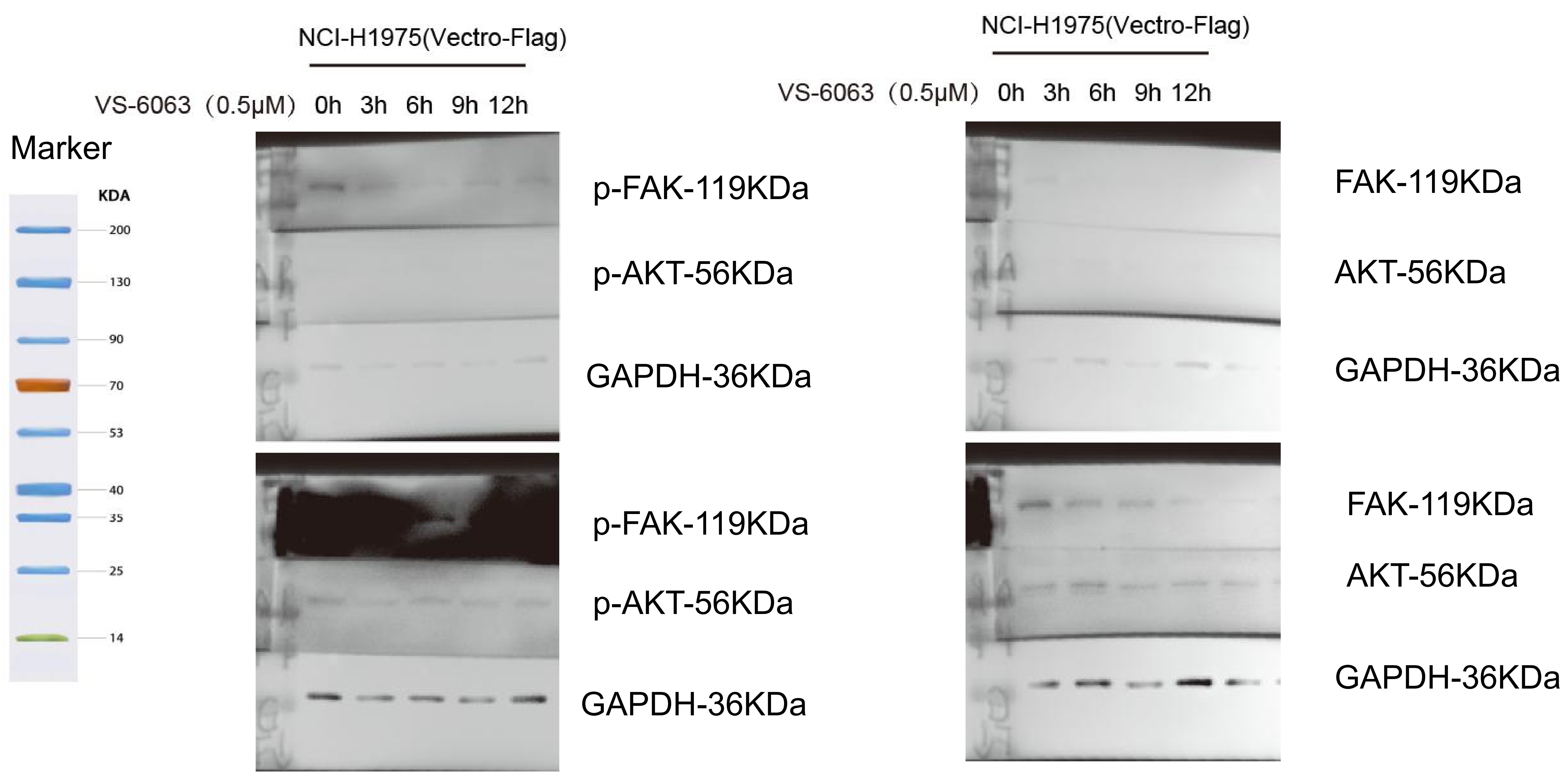
NCI-H1975(Vectro-Flag)
VS-6063（0.5μM） 0h 3h 6h 9h 12h
Marker
KDA
200
130
90
70
53
40
35
25
14
p-FAK-119KDa
p-AKT-56KDa
GAPDH-36KDa
p-FAK-119KDa
p-AKT-56KDa
GAPDH-36KDa
NCI-H1975(Vectro-Flag)
VS-6063（0.5μM） 0h 3h 6h 9h 12h
FAK-119KDa
AKT-56KDa
GAPDH-36KDa
FAK-119KDa
AKT-56KDa
GAPDH-36KDa

figureS4B-WB

Marker

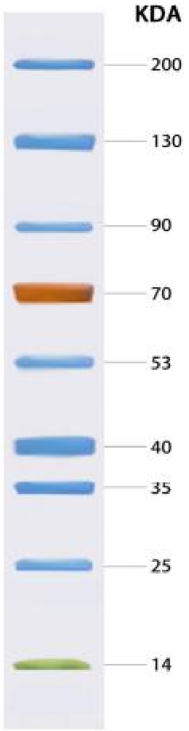

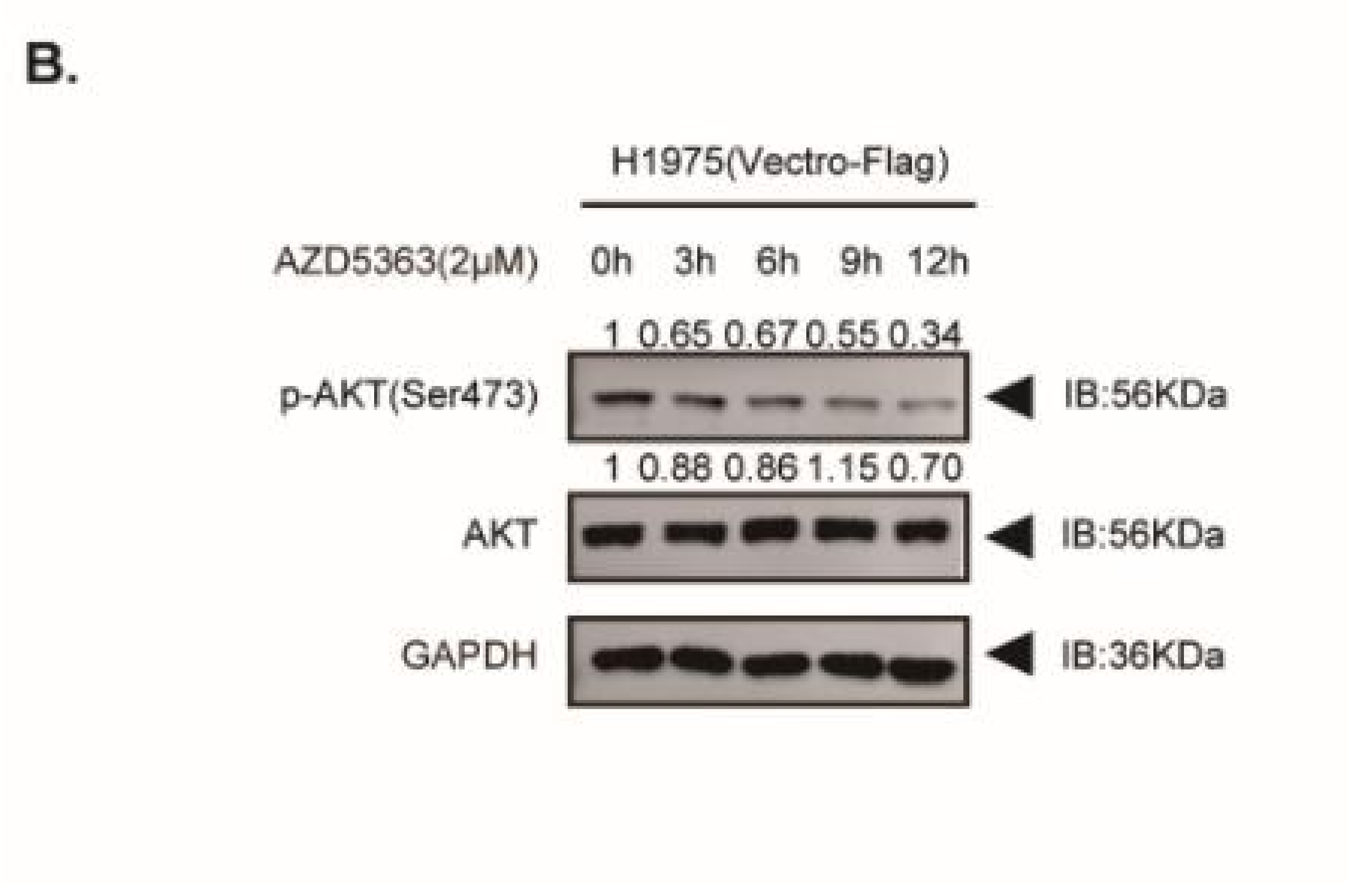

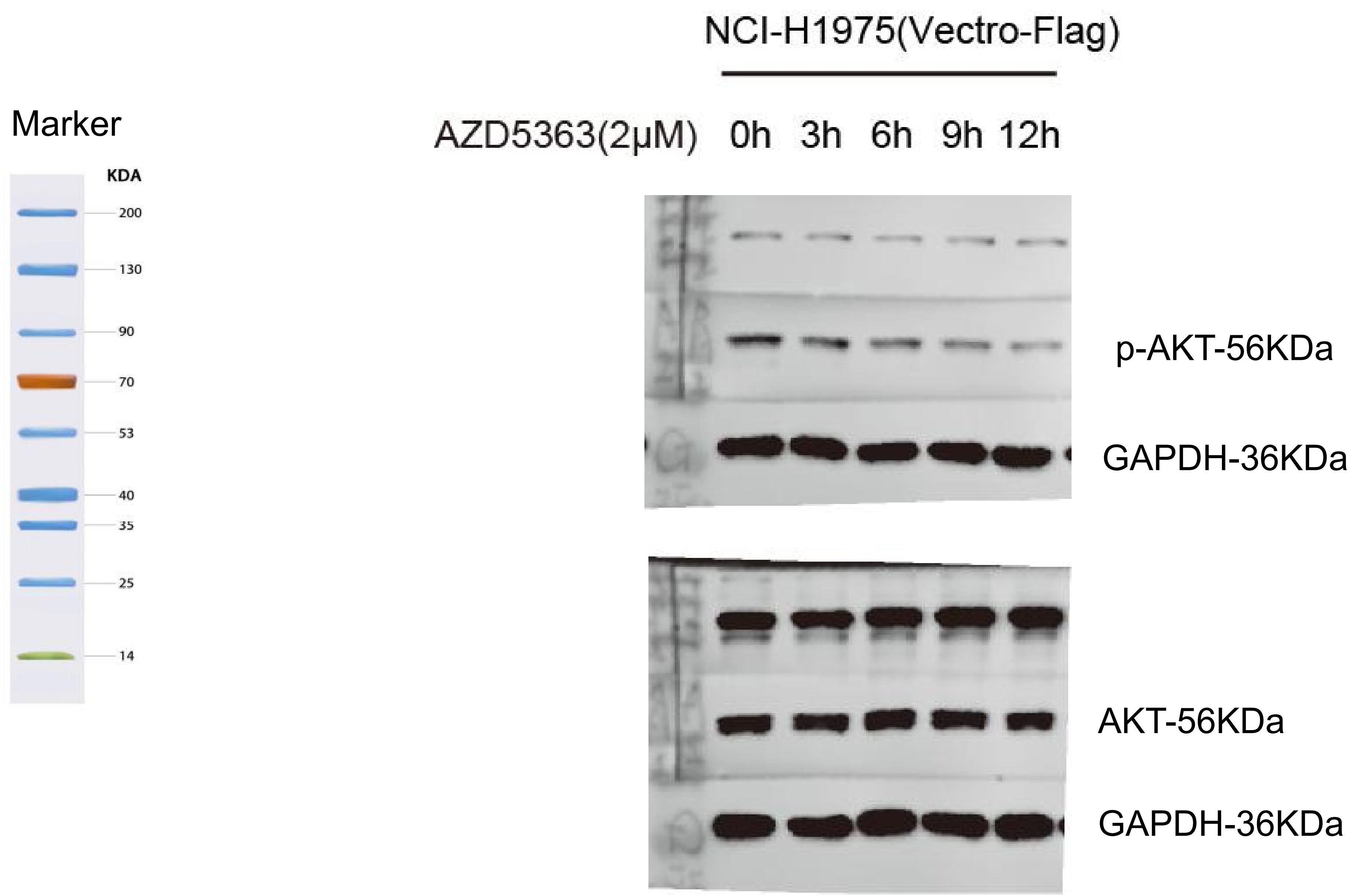

Marker
KDA
200
130
90
70
53
40
35
25
14
NCI-H1975(Vectro-Flag)
AZD5363(2μM)
0h
3h
6h
9h
12h
p-AKT-56KDa
GAPDH-36KDa
AKT-56KDa
GAPDH-36KDa

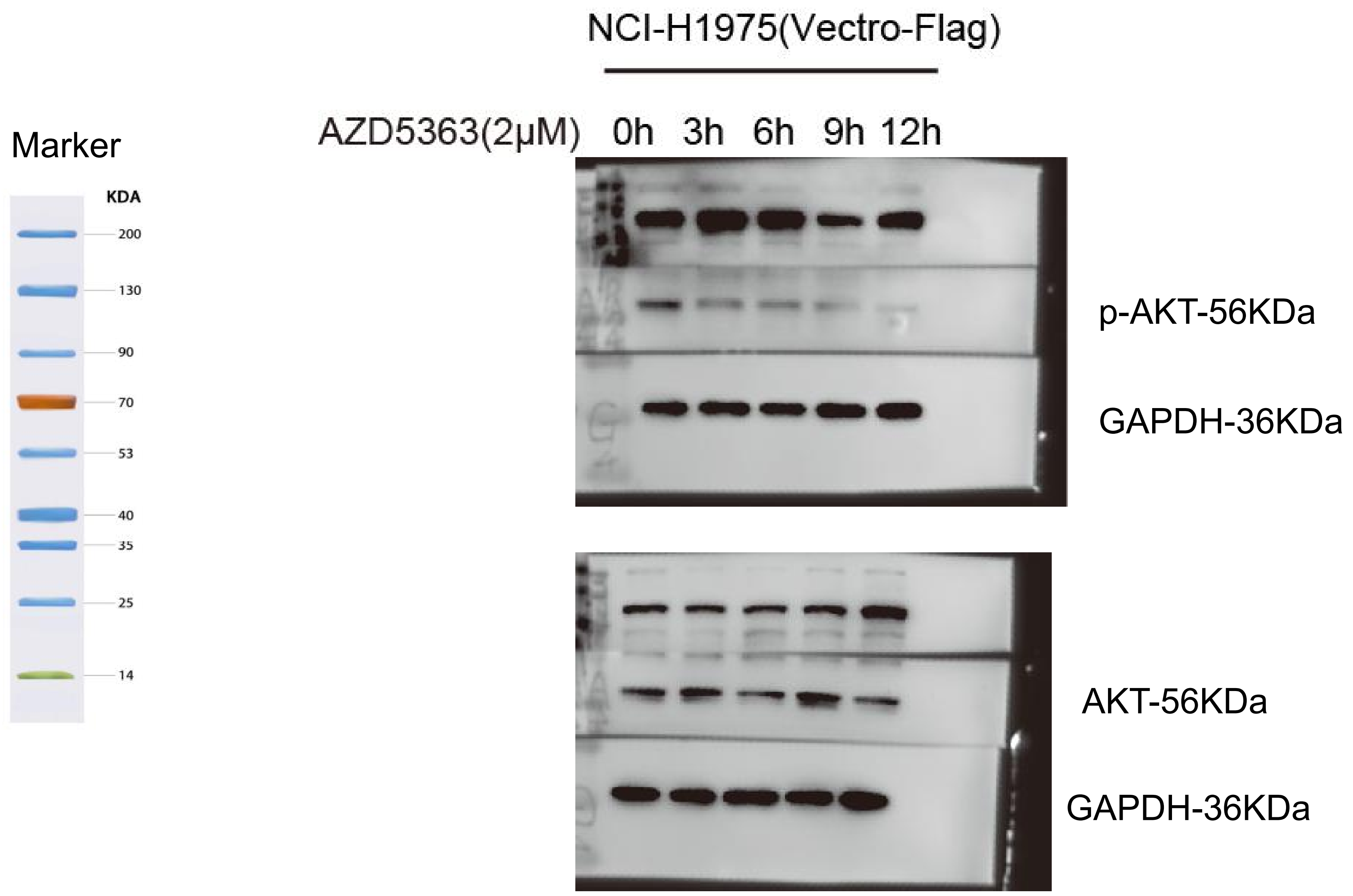
NCI-H1975(Vectro-Flag)
Marker
KDA
200
130
90
70
53
40
35
25
14
AZD5363(2μM) 0h 3h 6h 9h 12h
p-AKT-56KDa
GAPDH-36KDa
AKT-56KDa
GAPDH-36KDa

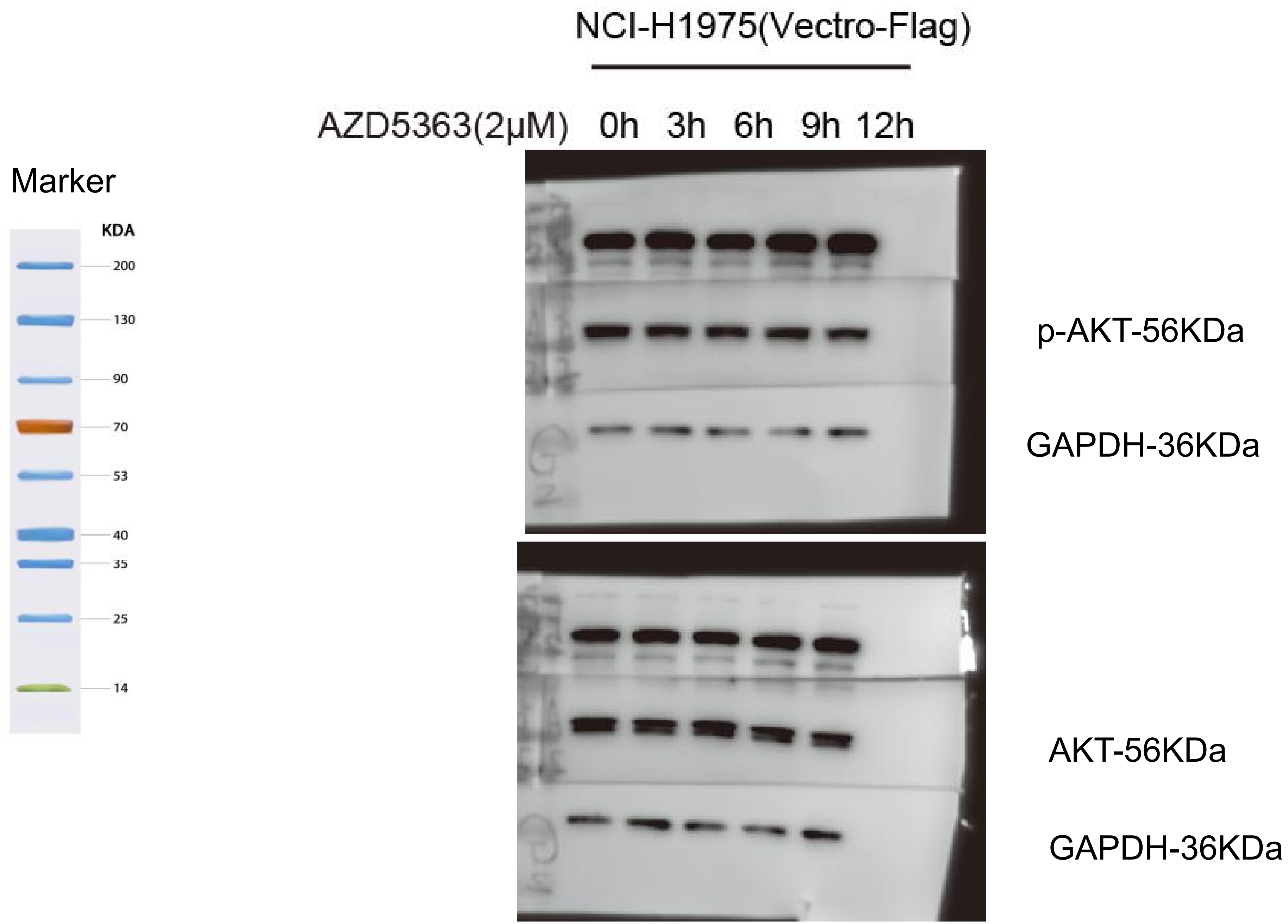
NCI-H1975(Vectro-Flag)
AZD5363(2μM) 0h 3h 6h 9h 12h
Marker
KDA
200
130
90
70
53
40
35
25
14
p-AKT-56KDa
GAPDH-36KDa
AKT-56KDa
GAPDH-36KDa

figureS3E-WB

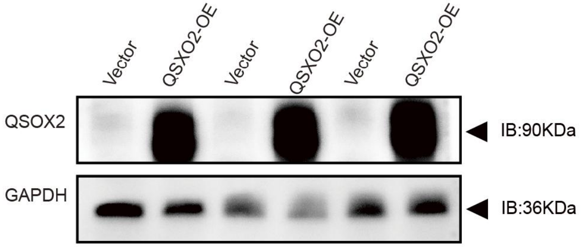

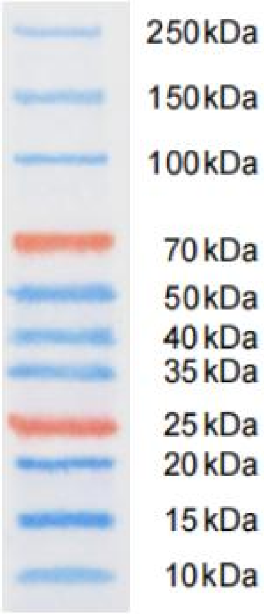

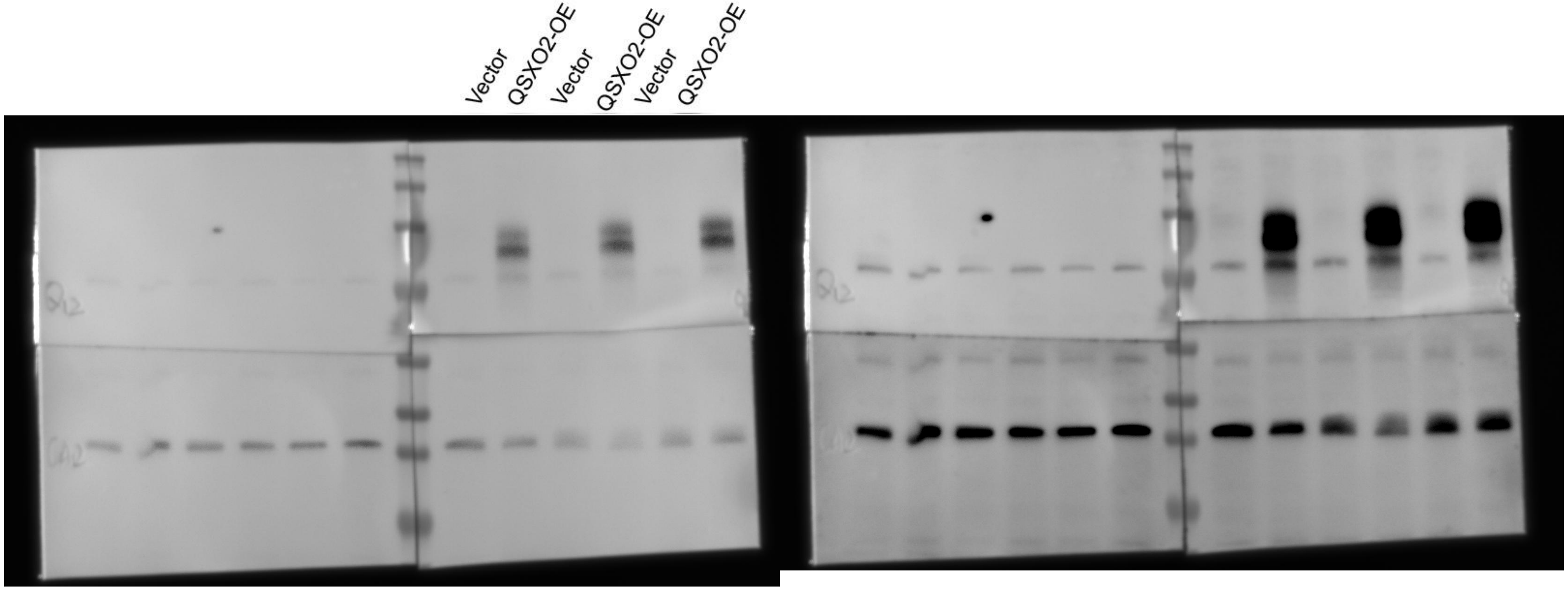

QSOX2-90KDa

GAPDH-36KDa

figureS5E-WB

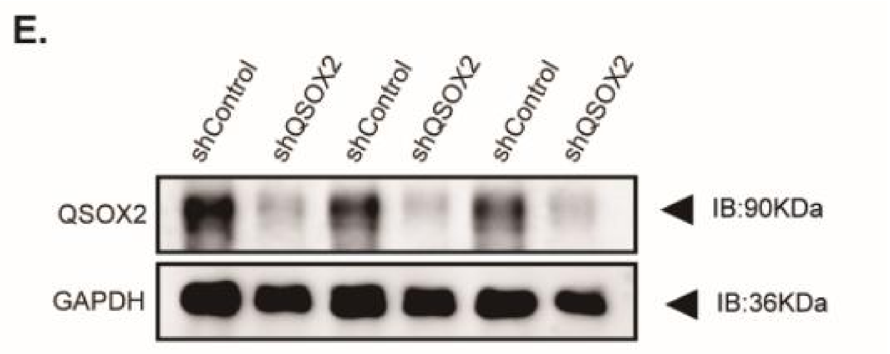

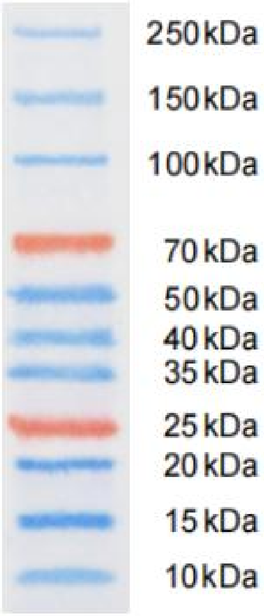

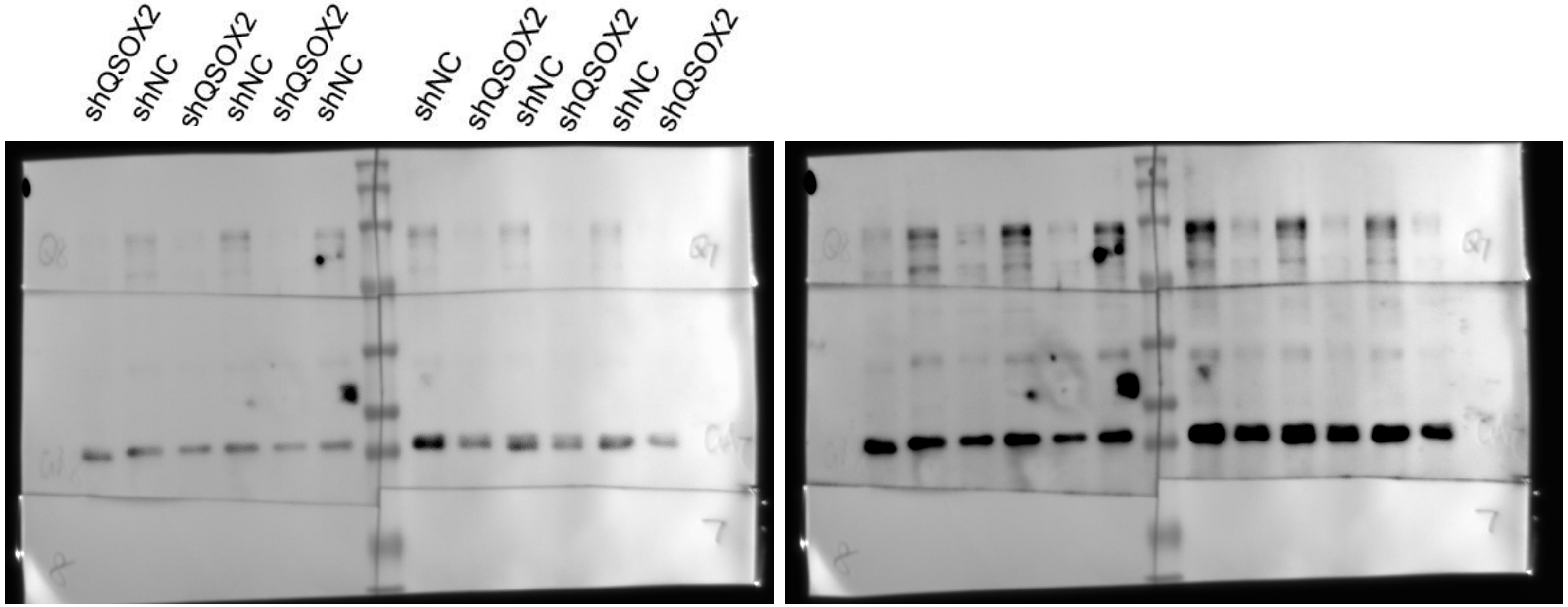

QSOX2-90KDa

GAPDH-36KDa
